# Supplementary material for: Interplay of diruthenium catalyst in controlling enantioselective propargylic substitution reactions with visible light-generated alkyl radicals
Source: Nat Commun. 2023 Feb 23;14:859. doi: 10.1038/s41467-023-36453-9 (PMC9950057; doi:10.1038/s41467-023-36453-9)
Supplement: Supplementary file 1 — Supplementary Information [file 41467_2023_36453_MOESM1_ESM.pdf]

Supplementary Information

Interplay of diruthenium catalyst in controlling enantioselective propargylic substitution reactions with visible light-generated alkyl radicals

Yulin Zhang[1], Yoshiaki Tanabe[1]*, Shogo Kuriyama[1], Ken Sakata[2]* & Yoshiaki Nishibayashi[1]*

[1] Department of Applied Chemistry, School of Engineering, The University of Tokyo, Hongo, Bunkyo-ku, Tokyo, 113-8656 Japan

[2] Faculty of Pharmaceutical Sciences, Toho University, Miyama, Funabashi, Chiba, 274-8510 Japan

Table of Contents

1. Supplementary Methods **S2**
- **1.1. General methods** **S2**
- **1.2. General procedure for the preparation of 3 and 8** **S2**
- **1.3. General procedure for the preparation of 4** **S4**
- **1.4. General procedure for the preparation of 5, 7, and 9** **S6**
- **1.5. Large-Scale Preparation of 7aa** **S20**
- **1.6. Preparation of 10** **S21**
- **1.7. Preparation of 11** **S21**
- **1.8. Preparation of 12** **S22**
- **1.9. Preparation of 13** **S22**
- **1.10 Preparation of 14** **S23**
- **1.11. Stoichiometric reaction of 14 with 4a** **S23**
- **1.12. Catalytic reaction of 3a with 4a by using 14 as a catalyst** **S24**
- **1.13. Reactions in the presence of TEMPO** **S24**
- **1.14. Deuterium labeling reaction** **S24**
- **1.15. Stern–Volmer analysis** **S25**
- **1.16. Light on/off experiment** **S25**
- **1.17. Cyclic voltammetric studies** **S25**
- **1.18. Time profile experiment** **S27**
- **1.19. Determination of quantum yields** **S27**
- **1.20. X-ray crystallographic study** **S28**
- **1.21. DFT calculations** **S31**
- **1.22. NMR Charts** **S35**
- **1.23: HPLC Charts** **S87**

2. Supplementary References **S129**

1. Supplementary Methods

1.1. General methods

All reactions were carried out under a dry nitrogen atmosphere by using standard Schlenk techniques. **1**,[1] **2**,[2] **3a**,[3] **3b**,[4] **3d**,[5] **3e**,[6] **3f**,[6] **3g**,[7] **3i**,[8] **3j**,[6] **3n**,[6] **4a**,[9] **4b**,[9] **4c**,[9] **4d**,[S9] **4e**,[10] **4f**,[9] **4g**,[11] **4j**,[12] **4k**,[13] **4l**,[12] **4m**,[10] **4n**,[14] **4p**,[12] **4q**,[9] **4r**,[15] **4t**,[16] **4u**,[17] **4v**,[11] **14**,[5] **16**,[18] **[Ru]-1**,[19] and **[Ru]-2**[19] were prepared according to the literature procedures. Other reagents include starting materials for the preparation of **1**, **2**, **3a**–**3n**, **4a**–**4v**, **5**, **8**, **14**, **16**, **[Ru]-1**, and **[Ru]-2**, *fac*-[Ir(ppy)$_3$] (ppy = 2-(pyridyl)phenyl), *fac*-[Ir(Fppy)$_3$] (Fppy = 3,5-difluoro-2-(pyridyl)phenyl), [Ir(ppy)$_2$(dtbbpy)]PF$_6$ (dtbbpy = 4,4′-di-tert-butyl-2,2′-bipyridine), NH_4BF_4, Lewis acids ($BF_3 \cdot Et_2O$, $B(OH)_3$, BCl_3, $Sc(OTf)_3$ (OTf = trifluoromethanesulfonate)), TEMPO, and solvents were obtained from commercial sources. Solvents were dried by general methods and degassed before use. Flash column chromatography was carried out on a Yamazen YFLC-AI-580 system. Gas chromatography–mass spectroscopy (GC–MS) was performed on a Shimadzu GCMS-QP2010 PLUS instrument. HPLC analyses were performed on Hitachi L-7100 and GL-7410 apparatuses equipped with a UV detector using 25 cm x 4.6 mm DAICEL Chiralpak columns. Specific rotations were measured on a JASCO DIP-1000 polarimeter. X-ray analysis was performed by a Rigaku XtaLAB Synergy-S diffractometer. Photoluminescence spectra were measured on a Shimadzu RF-5300PC spectrophotometer. Melting points were measured by using a Stanford Research Systems OptiMelt MPA100. High-resolution FAB mass spectra were measured on a JEOL JMS-700 mass spectrometer. Specific rotations were measured on a JASCO DIP-1000 polarimeter. ^{1}H NMR (400 MHz), ^{13}C{^{1}H} NMR (100 MHz), and ^{19}F NMR (376 MHz, referenced to $CF_3C_6H_5$ in $CDCl_3$ at δ –64.0) spectra were measured in $CDCl_3$, $(CD_3)_2CO$ or CD_3CN on a JEOL ECS-400 spectrometer with δ values in ^{1}H and ^{13}C{^{1}H} NMR calibrated by using residual peaks of $CHCl_3$ (^{1}H: 7.26; ^{13}C: 77.0), $(CD_2H)_2CO$ (^{1}H, 2.09; ^{13}C: 206.0), CD_2HCN (^{1}H, 1.93; ^{13}C: 117.7) respectively.

1.2. General procedure for the preparation of propargylic alcohol substrates (3 and 8) (taking 1,1,1-trifluoro-2-(4-ethylphenyl)but-3-yn-2-ol (3c) as a typical example)

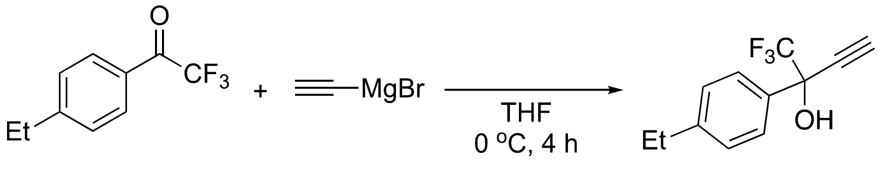

3c

To a solution of 2,2,2-trifluoro-1-(4-ethylphenyl)ethan-1-one (3.00 mmol, 607 mg) in anhydrous THF (15 mL) was added ethynylmagnesium bromide (0.5 M in THF, 9.0 mL, 4.5 mmol) at 0 °C. After stirring for 4 h, the mixture was quenched by saturated NH_4Cl aq. (4 mL), and the solution was extracted with Et_2O (10 ml × 2). The combined organic layers were dried over anhydrous Na_2SO_4 and concentrated under reduced pressure. The residue was purified by column chromatography with hexane/EtOAc (95:5-90:10) to give 1,1,1-trifluoro-2-(4-ethylphenyl)but-3-yn-2-ol as a pale yellow oil (588.8 mg, 2.58 mmol, 86% isolated yield based on the amount of 2,2,2-trifluoro-1-(4-ethylphenyl)ethan-1-one). ^{1}H NMR (400 MHz, $(CD_3)_2CO$): δ 7.76 (d, J = 8.4 Hz, 2H), 7.33 (d, J = 8.4 Hz, 2H), 6.67 (s, 1H), 3.49 (s, 1H), 2.71 (q, J = 7.6 Hz, 2H), 1.27 (t, J = 7.6 Hz, 3H); ^{13}C{^{1}H} NMR (100 MHz, $(CD_3)_2CO$): δ 145.7, 133.8, 127.7, 127.4, 124.1 (q, $^1J_{CF}$ = 283.7 Hz), 80.3, 77.1, 72.4 (q, $^2J_{CF}$ = 31.6 Hz), 28.4, 15.2; ^{19}F NMR (376 MHz, $(CD_3)_2CO$): δ –82.4 (s); HRMS (FAB+) (m/z): $[M]^+$ calcd. for $C_{12}H_{11}F_3O$: 228.0762; found: 228.0763.

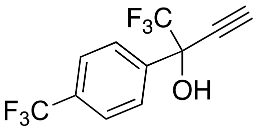

3h

1,1,1-Trifluoro-2-(4-(trifluoromethyl)phenyl)but-3-yn-2-ol (**3h**). A pale yellow oil (683.8 mg, 2.55 mmol, 85% isolated yield based on the amount of 2,2,2-trifluoro-1-(4-(trifluoromethyl)phenyl)ethan-1-one). ^{1}H NMR (400 MHz, $CDCl_3$): δ 7.88 (d, J = 8.4 Hz, 2H), 7.69 (t, J = 8.4 Hz, 2H), 3.37 (s, 1H), 2.87 (s, 1H); ^{13}C{^{1}H} NMR (100 MHz, $(CD_3)_2CO$)): δ 141.3, 131.7 (q, $^{1}J_{CF}$ = 32.2 Hz), 128.8, 125.9 (q, $^{3}J_{CF}$ = 3.5 Hz), 124.9 (q, $^{2}J_{CF}$ = 270.2 Hz), 124.2 (q, $^{3}J_{CF}$ = 284.0 Hz), 79.7, 78.7, 72.6 (q, $^{2}J_{CF}$ = 31.9 Hz) (^{13}C NMR spectrum of this compound was measured in acetone-d_6 because some peaks of this compound overlapped with those of $CDCl_3$); ^{19}F NMR (376 MHz, $CDCl_3$): δ –62.9 (s), –80.4 (s); HRMS (FAB+) (m/z): $[M+H]^+$ calcd. for $C_{11}H_7F_6O$, 269.0401; found, 269.0401.

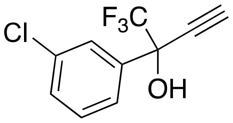

3k

1,1,1-Trifluoro-2-(3-chloromophenyl)but-3-yn-2-ol (**3k**). A pale yellow oil (667.1 mg, 2.49 mmol, 83% isolated yield based on the amount of 1-(3-chlorophenyl)-2,2,2-trifluoroethan-1-one). ^{1}H NMR (400 MHz, $(CD_3)_2CO$): δ 7.75 (br, 1H), 7.63 (d, J = 7.6 Hz, 1H), 7.41 (dq, J = 8.1, 1.1 Hz, 1H), 7.36 (pseudo t, J = 7.8 Hz, 1H), 3.26 (s, 1H), 2.85 (s, 1H); ^{13}C{^{1}H} NMR (100 MHz, $(CD_3)_2CO$): δ 136.4, 134.3, 129.9, 129.5, 127.4, 125.3, 122.8 (q, $^{1}J_{CF}$ = 284.3 Hz), 78.8, 77.2, 72.3 (q, $^{2}J_{CF}$ = 32.6 Hz); ^{19}F NMR (376 MHz, $(CD_3)_2CO$): δ –82.0 (s); HRMS (FAB+) (m/z): $[M]^+$ calcd. for $C_{10}H_6ClF_3O$, 234.0059; found: 234.0068.

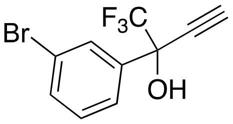

3l

1,1,1-Trifluoro-2-(3-bromophenyl)but-3-yn-2-ol (**3l**). A pale yellow oil (667.1 mg, 2.40 mmol, 80% isolated yield based on the amount of 1-(3-bromophenyl)-2,2,2-trifluoroethan-1-one). ^{1}H NMR (400 MHz, $(CD_3)_2CO$): δ 8.09 (br, 1H), 7.92 (d, J = 8.0 Hz, 1H), 7.76 (dq, J = 8.0, 1.0 Hz, 1H), 7.54 (t, J = 8.0 Hz, 1H), 7.09 (s, 1H), 3.68 (s, 1H); ^{13}C{^{1}H} NMR (100 MHz, $(CD_3)_2CO$): δ 138.6, 132.3, 130.0, 129.9, 126.1, 123.4 (q, $^{1}J_{CF}$ = 284.0 Hz) 121.6, 79.0, 77.6, 71.6 (q, $^{2}J_{CF}$ = 31.9 Hz); ^{19}F NMR (376 MHz, $(CD_3)_2CO$, 376 MHz): δ –80.7 (s); HRMS (FAB+) (m/z): $[M]^+$ calcd. for $C_{10}H_6BrF_3O$, 277.9554; found: 277.9565.

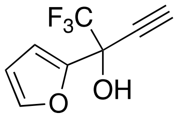

3m

1,1,1-Trifluoro-2-(furan-2-yl)but-3-yn-2-ol (3m). A pale yellow oil (427.7 mg, 2.25 mmol, 75% isolated yield based on the mount of 2,2,2-trifluoro-1-(furan-2-yl)ethan-1-one). ^{1}H NMR (400 MHz, $CDCl_3$): δ 7.48 (d, J = 2.0, 1H), 6.69 (dd, J = 3.3, 0.9 Hz, 1H), 6.42 (dd, J = 3.3, 2.0 Hz, 1H), 3.52 (br, 1H), 2.77 (s, 1H); ^{13}C{^{1}H} NMR (100 MHz, $CDCl_3$): δ 146.8, 144.2, 122.4 (q, $^{1}J_{CF}$ = 284.6 Hz), 111.1, 110.8, 76.7, 76.3, 69.0 (q, $^{2}J_{CF}$ = 34.5 Hz); ^{19}F NMR (376 MHz, $CDCl_3$): δ –81.5 (s); HRMS (FAB+) (*m*/*z*): $[M+H]^+$ calcd. for $C_8H_6F_3O_2$, 191.0320; found: 191.0318.

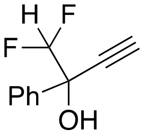

8

1,1-Difluoro-2-phenylbut-3-yn-2-ol (8). A pale yellow oil (477.3 mg, 2.61 mmol, 87% isolated yield based on the amount of 2,2-difluoro-1-phenylethan-1-one). ^{1}H NMR (400 MHz, $CDCl_3$): δ 7.69 (dd, J = 7.6, 1.6 Hz, 2H), 7.47–7.38 (m, 3H), 5.72 (t, J = 56.0 Hz, 1H), 3.02(s, 1H), 2.80 (s, 1H); ^{13}C{^{1}H} NMR (100 MHz, $(CD_3)_2CO$): δ 137.8, 128.8, 128.1, 127.0, 115.4 (t, $^{1}J_{CF}$ = 249.6 Hz), 81.2, 76.9, 72.3 (t, $^{2}J_{CF}$ = 23.5 Hz) (^{13}C NMR spectrum of this compound was measured in acetone-d_6 because some peaks of this compound overlapped with those of $CDCl_3$); ^{19}F NMR (376 MHz, $CDCl_3$): δ –129.3 (dd, $^{2}J_{FF}$ = 276 Hz, $^{2}J_{HF}$ = 60 Hz), –129.2 (dd, $^{2}J_{FF}$ = 276 Hz, $^{2}J_{HF}$ = 60 Hz); HRMS (FAB+) (*m*/*z*): $[M]^+$ calcd. for $C_{10}H_8F_2O$, 182.0543; found, 182.0548.

1.3. General procedure for the preparation of 4-alkyl-1,4-dihydropyridine derivatives (4) (taking diethyl 2,6-dimethyl-4-(4-(trifluoromethyl)benzyl)-1,4-dihydropyridine-3,5-dicarboxylate (4h) as a typical example)

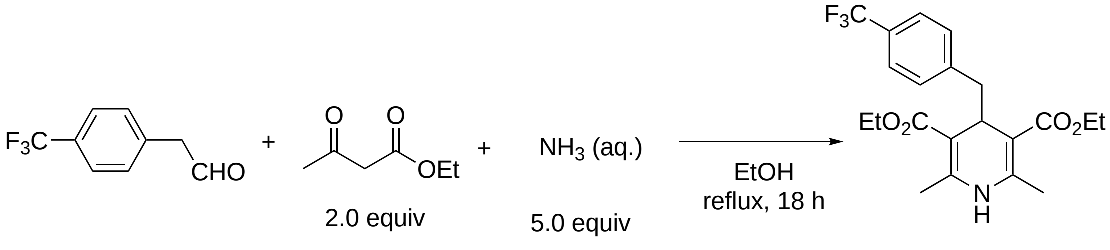

4h

In a 50 mL Schlenk flask were placed 2-(4-(trifluoromethyl)phenyl)acetaldehyde (940.8 mg, 5.00 mmol), ethyl acetoacetate (1301.4 mg, 10.00 mmol), and ethanol (20 mL) at room temperature under N_2, where an aqueous solution of 28 *wt* % NH_3 (1.5 mL, *ca.* 25 mmol) was added. The reaction mixture was stirred and refluxed for 18 h by using an oil bath, then was dried in vacuo. The residue was further purified by column chromatography (SiO_2) with a mixture of *n*-hexane/ethyl acetate (7:3) as an eluent to afford diethyl 2,6-dimethyl-4-(4-(trifluoromethyl)benzyl)-1,4-dihydropyridine-3,5-dicarboxylate (**4h**) as a white solid (1070.1 mg, 2.60 mmol, 52% yield based on the amount of 2-(4-(trifluoromethyl)phenyl)acetaldehyde). mp: 129.0–130.2 °C; ^{1}H NMR (400 MHz, $CDCl_3$): δ 7.40 (d, J = 7.8 Hz, 2H,), 7.11 (d, J = 7.8 Hz, 2H,), 6.02 (s, 1H), 4.18 (t, J = 5.7 Hz, 1H), 4.08–3.92 (m, 4H), 2.60 (d, J = 5.7 Hz, 2H), 2.16 (s, 6H), 1.17 (t, J = 7.2 Hz, 6H); ^{13}C{^{1}H} NMR (100 MHz, $CDCl_3$): δ

167.6, 145.9, 143.6, 130.1, 127.8 (q, $^2J_{CF}$ = 31.9 Hz), 124.4 (q, $^1J_{CF}$ = 270.2 Hz), 124.0 (q, $^3J_{CF}$ = 2.9 Hz), 101.2, 59.6, 42.1, 35.3, 18.9, 14.1; ^{19}F NMR (376 MHz, $CDCl_3$): δ –63.7 (s); HRMS (FAB+) (*m*/*z*): $[M]^+$ calcd. for $C_{21}H_{24}F_3NO_4$, 411.1657; found, 411.1653.

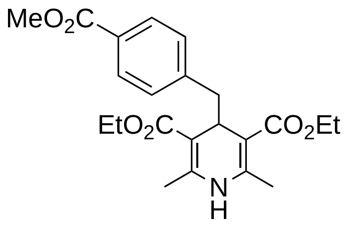

4i

Diethyl 4-(4-(methoxycarbonyl)benzyl)-2,6-dimethyl-1,4-dihydropyridine-3,5-dicarboxylate (**4i**). A white solid (762.2 mg, 1.90 mmol, 38% isolated yield, based on the amount of methyl 4-(2-oxoethyl)benzoate). mp: 86.5–88.0 °C; ^{1}H NMR (400 MHz, $CDCl_3$): δ 7.84 (d, *J* = 8.4 Hz, 2H), 7.08 (d, *J* = 8.4 Hz, 2H), 5.20 (s, 1H), 4.24 (t, *J* = 5.5 Hz, 1H), 4.15–4.02 (m, 4H), 3.89 (s, 1H), 2.65 (d, *J* = 5.5 Hz, 2H), 2.15 (s, 6H), 1.24 (t, *J* = 7.4 Hz, 6H); $^{13}C\{^1H\}$ NMR (100 MHz, $CDCl_3$): δ 167.6, 167.4, 145.5, 145.2, 130.1, 128.5, 127.6, 101.4, 59.7, 51.9, 42.2, 35.4, 19.2, 14.3; HRMS (FAB+) (*m*/*z*): $[M+H]^+$ calcd. for $C_{22}H_{28}NO_6$, 402.1917; found, 402.1936.

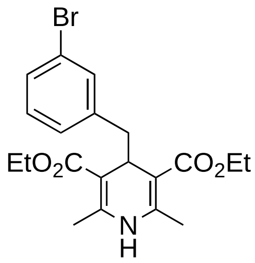

4o

Diethyl 4-(3-bromobenzyl)-2,6-dimethyl-1,4-dihydropyridine-3,5-dicarboxylate (**4o**). A white solid (971.3 mg, 2.30 mmol, 46% isolated yield, based on the amount of 2-(3-bromobenzyl)acetaldehyde). mp: 88.3–89.5 °C; ^{1}H NMR (400 MHz, $CDCl_3$): δ 7.27 (ddd, *J* = 7.8, 2.0, 0.9 Hz1H,), 7.20 (t, *J* = 2.0 Hz, 1H,), 7.03 (t, *J* = 7.8 Hz, 1H), 6.91 (d of pseudo t, *J* = 7.8, 1.6 Hz, 1H), 5.36 (s, 1H), 4.19 (t, *J* = 5.6 Hz, 1H), 4.15–4.04 (m, 4H), 2.54 (d, *J* = 5.6 Hz, 2H), 2.18 (s, 6H), 1.26 (t, *J* = 7.2 Hz, 6H); $^{13}C\{^1H\}$ NMR (100 MHz, $CDCl_3$): δ 167.6, 145.7, 141.7, 133.0, 128.8, 128.7, 128.6, 121.3, 101.4, 59.7, 41.9, 35.4, 19.2, 14.3; HRMS (FAB+) (*m*/*z*): $[M+H]^+$ calcd. for $C_{20}H_{25}BrNO_4$, 422.0967; found, 422.0946.

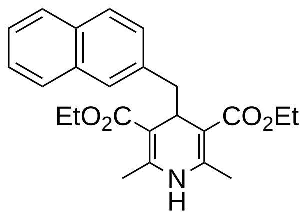

4s

Diethyl 2,6-dimethyl-4-(naphthalen-2-ylmethyl)-1,4-dihydropyridine-3,5-dicarboxylate (**4s**). A white solid (846.0 mg, 2.15 mmol, 43% isolated yield, based on the amount of 2-(4-(naphthalen-2-ylmethyl))acetaldehyde). mp: 119.0–120.6 °C; ^{1}H NMR (400 MHz, $CDCl_3$): δ 7.78-7.71 (m, 2H),

7.65 (d, $J = 8.8$ Hz, 1H,), 7.43 (s, 1H), 7.41-7.36 (m, 2H), 7.20 (dd, $J = 8.0$ Hz, 1.2Hz, 1H), 5.09 (s, 1H), 4.28 (t, $J = 5.4$ Hz, 1H), 4.10-3.95 (m, 4H), 2.75 (d, $J = 5.2$ Hz, 2H), 2.12 (s, 6H), 1.19 (t, $J = 7.2$ Hz, 6H); $^{13}C\{^1H\}$ NMR (100 MHz, $CDCl_3$): δ 167.8, 145.4, 137.0, 133.2, 131.9, 129.1, 128.2, 127.4 (overlapping), 126.3, 125.5, 124.9, 101.8, 59.6, 42.4, 35.6, 19.2, 14.3; HRMS (FAB+) (*m/z*): $[M]^+$ calcd. for $C_{24}H_{27}NO_4$, 393.1940; found, 393.1951.

1.4. General procedure for the preparation of chiral propargylic alkylation products (5, 7, and 9) (taking (*R*)-(2-(trifluoromethyl)but-3-yne-1,2-diyl)dibenzene (7aa) as a typical example)

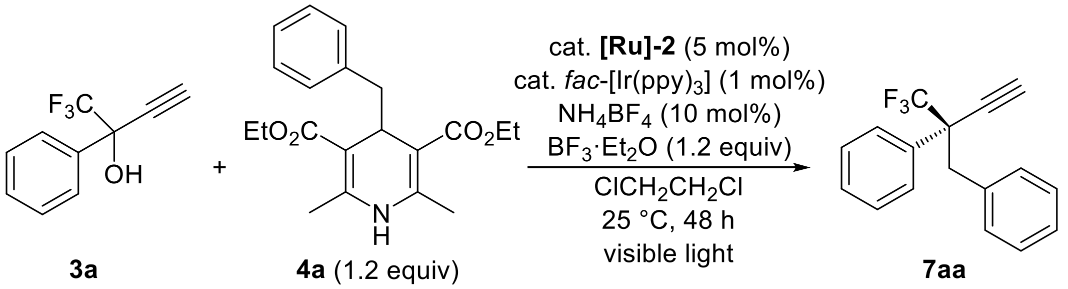

In an oven dried 20 mL Schlenk flask were placed **[Ru]-2** (6.3 mg, 0.0050 mmol) and NH_4BF_4 (1.1 mg, 0.010 mmol) under N_2. Anhydrous $ClCH_2CCH_2Cl$ (2.0 mL) was added, and then the mixture was magnetically stirred at room temperature for 30 min. Then, 1,1,1-trifluoro-2-phenylbut-3-yn-2-ol (**3a**) (20.0 mg. 0.10 mmol), diethyl 4-benzyl-2,6-dimethyl-1,4-dihydropyridine-3,5-dicarboxylate (**4a**) (41.2 mg, 0.12 mmol), *fac*-[Ir(ppy)$_3$] (0.7 mg, 0.0011 mmol), and $BF_3{\cdot}Et_2O$ (15 µL, 17 mg, 0.12 mmol) were added under N_2 at room temperature. The reaction flask was placed in an As One LTB-125 constant low temperature water bath set at 25 °C, and was illuminated from the bottom of the bath with an Aitech System TMN100×120–22WD 12 W white LED lamp (400 nm to 750 nm) at a distance of approximately 2 cm from the light source for 48 h. The volatiles were removed *in vacuo*, and the residue was purified by column chromatography (SiO_2) with *n*-hexane as an eluent to afford (*R*)-(2-(trifluoromethyl)but-3-yne-1,2-diyl)dibenzene (**7aa**) as a colorless oil (22.2 mg, 0.081 mmol, 81% yield based on the amount of **3a**). $[\alpha]_D^{20} = -12.2$ (0.5 M in $CHCl_3$); ^1H NMR (400 MHz, $CDCl_3$): δ 7.68–7.64 (m, 2H), 7.39–7.33 (m, 3H), 7.22–7.09 (m, 3H), 7.00–6.97 (m, 2H), 3.52 (d, $J = 13.2$ Hz, 1H), 3.42 (d, $J = 13.2$ Hz, 1H), 2.66 (s, 1H); $^{13}C\{^1H\}$ NMR (100 MHz, $CDCl_3$): δ 134.4, 133.3, 130.8, 128.5, 128.5, 128.3, 127.6, 127.0, 125.7 (q, $^1J_{CF} = 282.7$ Hz), 79.0 (q, $^3J_{CF} = 1.9$ Hz), 78.1, 53.0 (q, $^2J_{CF} = 26.5$ Hz), 40.7; ^{19}F NMR (376 MHz, $CDCl_3$): δ –73.5 (s); HRMS (FAB+) (*m/z*): $[M]^+$ calcd. for $C_{17}H_{13}F_3$, 274.0969; found: 274.0964. The enantiomeric excess of **7aa** was determined by HPLC analysis; DAICEL Chiralpak OJ-H, hexane/^{i}PrOH = 99/1, flow rate = 0.5 mL/min, λ = 220 nm, retention time: 14.6 min (major) and 19.9 min (minor), 94% ee.

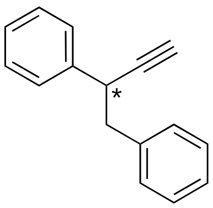

5

But-3-yne-1,2-diyldibenzene (5). A colorless oil (15.1 mg, 0.073 mmol, 73% yield based on the amount of **1**). $[\alpha]_D^{20} = -14.8$ (0.5 M in $CHCl_3$); ^1H NMR (400 MHz, $CDCl_3$) δ 7.33–7.19 (m, 8H), 7.13 (d, $J = 7.2$ Hz, 2H), 3.88 (ddd, $J = 7.8, 6.8, 2.5$ Hz, 1H), 3.06 (dd, $J = 13.2, 7.8$ Hz, 1H), 3.04 (dd, $J = 13.2, 6.8$ Hz, 1H), 2.28 (d, $J = 2.5$ Hz, 1H); $^{13}C\{^1H\}$ NMR (100 MHz, $CDCl_3$) δ 140.7, 138.6,

129.4, 128.4, 128.1, 127.6, 126.9, 126.5, 85.3, 71.9, 44.7, 39.8; HRMS (FAB+) (*m*/*z*): $[M+H]^+$ calcd. for $C_{16}H_{15}$, 207.1174; found: 207.1173. The enantiomeric excess of **5** was determined by HPLC analysis; DAICEL Chiralpak OJ-H, hexane/^{i}PrOH = 99/1, flow rate = 0.5 mL/min, λ = 220 nm, retention time: 21.8 min (minor) and 44.7 min (major), 48% ee.

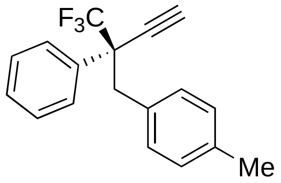

7ab

(*R*)-1-Methyl-4-(2-phenyl-2-(trifluoromethyl)but-3-yn-1-yl)benzene (7ab). A colorless oil (22.5 mg, 0.078 mmol, 78% yield based on the amount of **3a**). $[\alpha]_D^{20}$ = +21.9 (0.5 M in $CHCl_3$); ^1H NMR (400 MHz, $CDCl_3$): δ 7.68–7.64 (m, 2H), 7.39–7.33 (m, 3H), 6.92 (d, *J* = 8.0 Hz, 2H), 6.86 (d, *J* = 8.0 Hz, 2H), 3.48 (d, *J* = 13.6 Hz, 1H), 3.38 (d, *J* = 13.6 Hz, 1H), 2.65 (s, 1H), 2.24 (s, 3H); $^{13}C\{^1H\}$ NMR (100 MHz, $CDCl_3$): δ 136.5, 133.4, 131.2, 130.6, 128.5 (overlapping), 128.3, 128.2, 125.7 (q, $^1J_{CF}$ = 282.7 Hz), 79.1, 78.0, 53.0 (q, $^2J_{CF}$ = 26.2 Hz), 40.3, 21.0; ^{19}F NMR (376 MHz, $CDCl_3$): δ –73.5 (s); HRMS (FAB+) (*m*/*z*): $[M]^+$ calcd. for $C_{18}H_{15}F_3$, 288.1126; found: 288.1128. The enantiomeric excess of **7ab** was determined by HPLC analysis; DAICEL Chiralpak OJ-H, hexane/^{i}PrOH = 99/1, flow rate = 0.5 mL/min, λ = 220 nm, retention time: 13.7 min (major) and 18.9 min (minor), 94% ee.

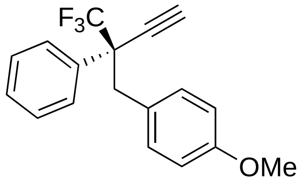

7ac

(*R*)-1-Methyl-4-(2-phenyl-2-(trifluoromethyl)but-3-yn-1-yl)benzene (7ac). A colorless oil (24.0 mg, 0.079 mmol, 79% yield based on the amount of **3a**). $[\alpha]_D^{20}$ = +66.9 (0.5 M in $CHCl_3$); ^1H NMR (400 MHz, $CDCl_3$): δ 7.68–7.64 (m, 2H), 7.40–7.33 (m, 3H), 6.89 (d, *J* = 8.8 Hz, 2H), 6.66 (d, *J* = 8.8 Hz, 2H), 3.72 (s, 3H), 3.46 (d, *J* = 13.6 Hz, 1H), 3.37 (d, *J* = 13.6 Hz, 1H), 2.65 (s, 1H); $^{13}C\{^1H\}$ NMR (100 MHz, $CDCl_3$): δ 158.5, 133.4, 131.7, 128.5 (overlapping), 128.3, 126.3, 125.7 (q, $^1J_{CF}$ = 282.7 Hz), 113.0, 79.1 (q, $^3J_{CF}$ = 1.9 Hz), 78.0, 55.0, 53.1 (q, $^2J_{CF}$ = 26.2 Hz), 39.9; ^{19}F NMR (376 MHz, $CDCl_3$, 376 MHz): δ –71.7 (s); HRMS (FAB+) (*m*/*z*): $[M]^+$ calcd. for $C_{18}H_{15}F_3O$, 304.1075; found, 304.1088. The enantiomeric excess of **7ac** was determined by HPLC analysis; DAICEL Chiralpak OJ-H, hexane/^{i}PrOH = 90/10, flow rate = 0.5 mL/min, λ = 220 nm, retention time: 20.1 min (major) and 23.7 min (minor), 92% ee.

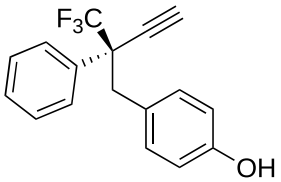

7ad

(*R*)-4-(2-Phenyl-2-(trifluoromethyl)but-3-yn-1-yl)phenol (7ad). A colorless oil (20.3 mg, 0.070 mmol, 70% yield based on the amount of **3a**). $[\alpha]_D^{20}$ = –40.9 (0.5 M in $CHCl_3$); ^1H NMR (400 MHz,

CDCl$_3$): δ 7.66–7.62 (m, 2H), 7.39–7.33 (m, 3H), 6.84 (d, J = 8.8 Hz, 2H), 6.57 (d, J = 8.8 Hz, 2H), 3.44 (d, J = 13.8 Hz, 1H), 3.35 (d, J = 13.8 Hz, 1H), 2.65 (s, 1H), 2.1–1.3 (br, 1H); ^{13}C{^{1}H} NMR (100 MHz, CDCl$_3$): δ 154.5, 133.4, 131.9, 128.5, 128.5, 128.3, 126.6, 125.7 (q, $^1J_{CF}$ = 282.7 Hz), 114.5, 79.1 (q, $^3J_{CF}$ = 1.9 Hz), 78.0, 53.2 (q, $^2J_{CF}$ = 26.2 Hz), 40.0; ^{19}F NMR (376 MHz, CDCl$_3$): δ –73.3 (s); HRMS (FAB+) (m/z): [M]$^+$ calcd. for $C_{17}H_{13}F_3O$, 290.0918; found, 290.0912. The enantiomeric excess of **7ad** was determined by HPLC analysis; DAICEL Chiralpak OJ-H, hexane/^{i}PrOH = 80/20, flow rate = 0.5 mL/min, λ = 220 nm, retention time: 19.8 min (major) and 31.3 min (minor), 92% ee.

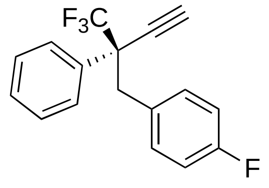

7ae

(*R*)-1-Fluoro-4-(2-phenyl-2-(trifluoromethyl)but-3-yn-1-yl)benzene (7ae). A colorless oil (23.4 mg, 0.080 mmol, 80% yield based on the amount of **3a**). [α]$_D^{20}$ = –17.1 (0.5 M in CHCl$_3$); ^{1}H NMR (400 MHz, CDCl$_3$): δ 7.65–7.61 (m, 2H), 7.39–7.34 (m, 3H), 6.93 (dd, J = 8.8, 5.6 Hz, 2H), 6.80 (pseudo t, J = 8.8 Hz, 2H), 3.47 (d, J = 13.2 Hz, 1H), 3.39 (d, J = 13.2 Hz, 1H), 2.66 (s, 1H); ^{13}C{^{1}H} NMR (100 MHz, CDCl$_3$): δ 162.0 (d, $^1J_{CF}$ = 244.4 Hz), 133.2, 132.2 (d, $^3J_{CF}$ = 7.6 Hz), 130.0 (d, $^4J_{CF}$ = 2.8 Hz), 128.7, 128.4, 128.4, 125.6 (q, $^1J_{CF}$ = 279.5 Hz), 114.5 (d, $^2J_{CF}$ = 21.0 Hz), 78.8 (q, $^3J_{CF}$ = 1.9 Hz), 78.2, 53.0 (q, $^2J_{CF}$ = 26.8 Hz), 40.0; ^{19}F NMR (376 MHz, CDCl$_3$): δ –73.4 (s), –117.3 (s); HRMS (FAB+) (m/z): [M+H]$^+$ calcd. for $C_{17}H_{13}F_4$, 293.0953; found, 293.0962. The enantiomeric excess of **7ae** was determined by HPLC analysis; DAICEL Chiralpak OJ-H, hexane/^{i}PrOH = 99/1, flow rate = 0.3 mL/min, λ = 220 nm, retention time: 32.2 min (major) and 35.0 min (minor), 92% ee.

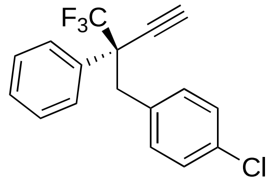

7af

(*R*)-1-Chloro-4-(2-phenyl-2-(trifluoromethyl)but-3-yn-1-yl)benzene (7af). A white solid (22.8 mg, 0.074 mmol, 74% yield based on the amount of **3a**). mp: 56.4–57.8 °C; [α]$_D^{20}$ = –19.5 (0.5 M in CHCl$_3$); ^{1}H NMR (400 MHz, CDCl$_3$): δ 7.65-7.61 (m, 2H), 7.40–7.34 (m, 3H), 7.08 (d, J = 8.2 Hz, 2H), 6.90 (d, J = 8.2 Hz, 2H), 3.47 (d, J = 13.6 Hz, 1H), 3.38 (d, J = 13.6 Hz, 1H), 2.67 (s, 1H); ^{13}C{^{1}H} NMR (100 MHz, CDCl$_3$): δ 133.0, 132.8, 132.0, 128.7, 128.4 (overlapping), 127.8, 125.6 (q, $^1J_{CF}$ = 282.7 Hz), 78.7 (q, $^3J_{CF}$ = 1.9 Hz), 78.3, 52.9 (q, $^2J_{CF}$ = 26.2 Hz), 40.1; ^{19}F NMR (376 MHz, CDCl$_3$): δ –73.5 (s); HRMS (FAB+) [M]$^+$ (m/z): calcd. for $C_{17}H_{12}ClF_3$, 308.0580; found: 308.0570. The enantiomeric excess of **7af** was determined by HPLC analysis; DAICEL Chiralpak OJ-H, hexane/^{i}PrOH = 99/1, flow rate = 0.5 mL/min, λ = 220 nm, retention time: 17.3 min (major) and 25.4 min (minor), 90% ee.

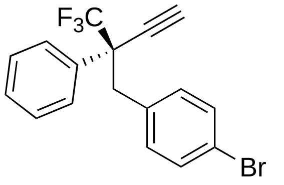

7ag

(*R*)-1-Bromo-4-(2-phenyl-2-(trifluoromethyl)but-3-yn-1-yl)benzene (7ag). A colorless oil (24.0 mg, 0.068 mmol, 68% yield based on the amount of **3a**). $[\alpha]_D^{20}$ = –49.5 (0.5 M in $CHCl_3$); ^{1}H NMR (400 MHz, $CDCl_3$): δ 7.65–7.61 (m, 2H), 7.38–7.34 (m, 3H), 7.23 (d, *J* = 8.8 Hz, 2H), 6.83 (d, *J* = 8.8 Hz, 2H), 3.45 (d, *J* = 13.6 Hz, 1H), 3.36 (d, *J* = 13.6 Hz, 1H), 2.67 (s, 1H); ^{13}C{^{1}H} NMR (100 MHz, $CDCl_3$): δ 133.3, 133.0, 132.4, 130.7, 128.7, 128.4 (overlapping), 125.6 (q, $^1J_{CF}$ = 282.7 Hz), 121.2, 78.7 (q, $^3J_{CF}$ = 2.4 Hz), 78.4, 52.8 (q, $^2J_{CF}$ = 26.8 Hz), 40.2; ^{19}F NMR (376 MHz, $CDCl_3$): δ –73.5; HRMS (FAB+) (*m/z*): $[M]^+$ calcd. for $C_{17}H_{12}BrF_3$, 352.0074; found, 352.0083. The enantiomeric excess of **7ag** was determined by HPLC analysis; DAICEL Chiralpak OJ-H, hexane/^{i}PrOH = 99/1, flow rate = 0.5 mL/min, λ = 220 nm, retention time: 15.4 min (major) and 23.7 min (minor), 94% ee.

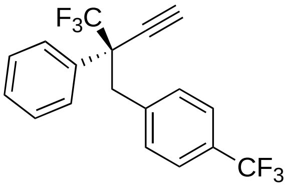

7ah

(*R*)-1-(2-Phenyl-2-(trifluoromethyl)but-3-yn-1-yl)-4-(trifluoromethyl)benzene (7ah). A white solid (25.3 mg, 0.074 mmol, 74% yield based on the amount of **3a**). mp: 54.5–55.5 °C; $[\alpha]_D^{20}$ = –49.1 (0.5 M in $CHCl_3$); ^{1}H NMR (400 MHz, $CDCl_3$): δ 7.66–7.62 (m, 2H), 7.39–7.35 (m, 5H), 7.08 (d, 2H, *J* = 8.4 Hz), 3.56 (d, *J* = 13.4 Hz, 1H), 3.46 (d, *J* = 13.4 Hz, 1H), 2.68 (s, 1H); ^{13}C{^{1}H} NMR (100 MHz, $(CD_3)_2CO$): δ 140.1, 133.6, 132.3, 129.6, 129.5 (q, $^2J_{CF}$ = 31.6 Hz), 129.4, 129.2, 126.6 (q, $^1J_{CF}$ = 282.4 Hz), 125.2 (q, $^1J_{CF}$ = 269.8 Hz), 125.0 (q, $^3J_{CF}$ = 2.8 Hz), 80.8, 79.1, 53.4 (q, $^2J_{CF}$ = 27.2 Hz), 40.2 (^{13}C NMR spectrum of this compound was measured in acetone-d_6 because some peaks of this compound overlapped with those of $CDCl_3$); ^{19}F NMR (376 MHz, $CDCl_3$): δ –64.1 (s), –73.6 (s); HRMS (FAB+) (*m/z*): $[M]^+$ calcd. for $C_{18}H_{12}F_6$, 342.0843; found, 342.0856. The enantiomeric excess of **7ah** was determined by HPLC analysis; DAICEL Chiralpak OJ-H, hexane/^{i}PrOH = 99/1, flow rate = 0.5 mL/min, λ = 220 nm, retention time: 11.4 min (major) and 15.6 min (minor), 91% ee.

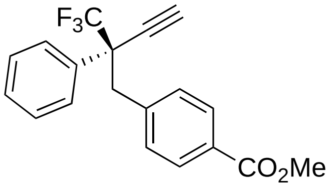

7ai

Methyl (*R*)-4-(2-phenyl-2-(trifluoromethyl)but-3-yn-1-yl)benzoate (7ai). A pale yellow solid (24.9 mg, 0.075 mmol, 75% yield based on the amount of **3a**). mp: 74.0–75.5 °C; $[\alpha]_D^{20}$ = –45.6 (0.5 M in $CHCl_3$); ^{1}H NMR (400 MHz, $CDCl_3$): δ 7.79 (d, *J* = 8.4 Hz, 2H), 7.65–7.62 (m, 2H), 7.38–7.33 (m, 3H), 7.05 (d, *J* = 8.4 Hz, 2H), 3.86 (s, 3H), 3.56 (d, *J* = 14.0 Hz, 1H), 3.47 (d, *J* = 14.0 Hz, 1H), 2.67 (s, 1H); ^{13}C{^{1}H} NMR (100 MHz, $CDCl_3$): δ 166.9, 139.7, 133.0, 130.8, 128.9, 128.8, 128.7, 128.4, 125.6 (q, $^1J_{CF}$ = 282.7 Hz), 78.6 (q, $^3J_{CF}$ = 1.9 Hz), 78.4, 52.8 (q, $^2J_{CF}$ = 26.8 Hz), 52.0, 40.7; ^{19}F

NMR (376 MHz, $CDCl_3$): δ –73.5 (s); HRMS (FAB+) (*m*/*z*): $[M+H]^+$ calcd. for $C_{19}H_{16}F_3O_2$, 333.1102; found, 333.1107. The enantiomeric excess of **7ai** was determined by HPLC analysis; DAICEL Chiralpak OJ-H, hexane/^{i}PrOH = 90/10, flow rate = 0.5 mL/min, λ = 220 nm, retention time: 18.9 min (major) and 31.1 min (minor), 96% ee.

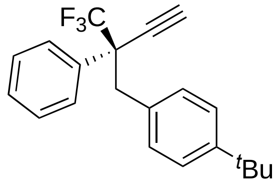

7aj

(*R*)-1-(*tert*-Butyl)-4-(2-phenyl-2-(trifluoromethyl)but-3-yn-1-yl)benzene (7aj). A white solid (23.1 mg, 0.070 mmol, 70% yield based on the amount of **3a**). mp: 88.7–89.9 °C; $[\alpha]_D^{20}$ = –13.2 (0.5 M in $CHCl_3$); ^1H NMR (400 MHz, $CDCl_3$): δ 7.69–7.66 (m, 2H), 7.39–7.33 (m, 3H,), 7.13 (d, *J* = 8.4 Hz, 2H), 6.92 (d, *J* = 8.4 Hz, 2H), 3.50 (d, *J* = 13.6 Hz, 1H), 3.39 (d, *J* = 13.6 Hz, 1H), 2.65 (s, 1H), 1.24 (s, 9H); $^{13}C\{^1H\}$ NMR (100 MHz, $CDCl_3$): δ 149.6, 133.5, 131.3, 130.3, 128.5, 128.5, 128.2, 125.7 (q, $^1J_{CF}$ = 282.7 Hz), 124.5, 79.2 (q, $^3J_{CF}$ = 1.9 Hz), 77.8, 52.9 (q, $^2J_{CF}$ = 26.2 Hz), 40.0, 34.3, 31.3; ^{19}F NMR (376 MHz, $CDCl_3$): δ –72.0 (s); HRMS (FAB+) (*m*/*z*): $[M]^+$ calcd. for $C_{21}H_{21}F_3$, 330.1595; found, 330.1594. The enantiomeric excess of **7aj** was determined by HPLC analysis; DAICEL Chiralpak OJ-H, hexane/^{i}PrOH = 99/1, flow rate = 0.5 mL/min, λ = 220 nm, retention time: 9.9 min (major) and 17.9 min (minor), 84% ee.

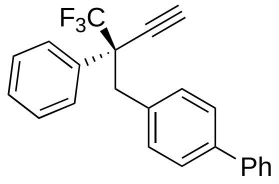

7ak

(*R*)-4-(2-Phenyl-2-(trifluoromethyl)but-3-yn-1-yl)-1,1'-biphenyl (7ak). A white solid (24.9 mg, 0.071 mmol, 71% yield based on the amount of **3a**). mp: 102.6–103.7 °C; $[\alpha]_D^{20}$ = –46.4 (0.5 M in $CHCl_3$); ^1H NMR (400 MHz, $CDCl_3$): δ 7.71–7.67 (m, 2H), 7.52 (dd, *J* = 7.2, 1.2 Hz, 2H), 7.42–7.34 (m, 7H), 7.30 (tt, *J* = 7.4, 1.6 Hz,1H), 7.05 (d, *J* = 8.0 Hz, 2H), 3.56 (d, *J* = 13.2 Hz, 1H), 3.47 (d, *J* = 13.2 Hz, 1H), 2.69 (s, 1H); $^{13}C\{^1H\}$ NMR (100 MHz, $CDCl_3$): δ 140.6, 139.7, 133.4, 133.3, 131.1, 128.7, 128.6, 128.5, 128.3, 127.2, 126.9, 126.2, 125.7 (q, $^1J_{CF}$ = 283.7 Hz), 79.0, 78.1, 53.0 (q, $^2J_{CF}$ = 26.5 Hz), 40.3; ^{19}F NMR (376 MHz, $CDCl_3$): δ –71.8 (s); HRMS (FAB+) (*m*/*z*): $[M]^+$ calcd. for $C_{23}H_{17}F_3$, 350.1282; found: 350.1270. The enantiomeric excess of **7ak** was determined by HPLC analysis; DAICEL Chiralpak OD, hexane/^{i}PrOH = 99/1, flow rate = 0.5 mL/min, λ = 220 nm, retention time: 11.4 min (minor) and 12.4 min (major), 83% ee.

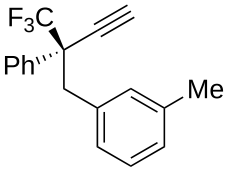

7al

(*R*)-1-Methyl-3-(2-phenyl-2-(trifluoromethyl)but-3-yn-1-yl)benzene (7al). A colorless oil (21.0 mg, 0.073 mmol, 73% yield based on the amount of **3a**). $[\alpha]_D^{20}$ = –14.9 (0.5 M in $CHCl_3$); ^{1}H NMR (400 MHz, $CDCl_3$): δ 7.67–7.64 (m, 2H), 7.38–7.33 (m, 3H), 7.00 (pseudo t, *J* = 7.4 Hz, 1H), 6.96 (d, *J* = 8.0 Hz, 1H), 6.79 (br, 1H), 6.76 (d, *J* = 6.8 Hz, 1H), 3.47 (d, *J* = 13.4 Hz, 1H), 3.38 (d, *J* = 13.4 Hz, 1H), 2.65 (s, 1H), 2.19 (s, 3H); ^{13}C{^{1}H} NMR (100 MHz, $CDCl_3$): δ 137.0, 134.2, 133.4, 131.7, 128.5, 128.2, 127.7, 127.7, 127.4, 127.2, 125.7 (q, $^1J_{CF}$ = 282.7 Hz), 79.0 (q, $^3J_{CF}$ = 1.9 Hz), 78.0, 53.0 (q, $^2J_{CF}$ = 26.2 Hz), 40.7, 21.3; ^{19}F NMR (376 MHz, $CDCl_3$): δ –72.0 (s); HRMS (FAB+) (*m*/*z*): $[M]^+$ calcd. for $C_{18}H_{15}F_3$, 288.1126; found, 288.1137. The enantiomeric excess of **7al** was determined by HPLC analysis; DAICEL Chiralpak OJ-H, hexane/^{i}PrOH = 99/1, flow rate = 0.5 mL/min, λ = 220 nm, retention time: 13.6 min (major) and 17.7 min (minor), 90% ee.

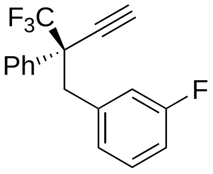

7am

(*R*)-1-Fluoro-3-(2-phenyl-2-(trifluoromethyl)but-3-yn-1-yl)benzene (7am). A colorless oil (23.4 mg, 0.080 mmol, 80% yield based on the amount of **3a**). $[\alpha]_D^{20}$ = –48.0 (0.5 M in $CHCl_3$); ^{1}H NMR (400 MHz, $CDCl_3$): δ 7.67–7.62 (m, 2H), 7.42–7.34 (m, 3H), 7.07 (dd, *J* = 14.0, 8.6 Hz, 1H), 6.85 (ddd, *J* = 8.6, 7.6, 2.8 Hz, 1H), 6.73 (d, *J* = 8.0 Hz, 1H), 6.72 (dd, *J* = 7.6, 2.4 Hz, 1H), 3.50 (d, *J* = 13.2 Hz, 1H), 3.41 (d, *J* = 13.2 Hz, 1H), 2.69 (s, 1H); ^{13}C{^{1}H} NMR (100 MHz, $CDCl_3$): δ 162.1 (d, $^1J_{CF}$ = 243.4 Hz), 136.8 (d, $^3J_{CF}$ = 7.7 Hz), 133.0, 128.9 (d, ^3J = 8.6 Hz), 128.7, 128.4 (overlapping), 126.5 (d, $^4J_{CF}$ = 1.9 Hz), 125.5 (q, $^1J_{CF}$ = 283.0 Hz), 117.6 (d, $^2J_{CF}$ = 22.0 Hz), 114.0 (d, $^2J_{CF}$ = 20.1 Hz), 78.7 (q, $^3J_{CF}$ = 1.9 Hz), 78.3, 52.8 (q, $^2J_{CF}$ = 26.8 Hz), 40.4; ^{19}F NMR (376 MHz, $CDCl_3$): δ –73.6 (s), –115.7 (s); HRMS (FAB+) (*m*/*z*): $[M]^+$ calcd. for $C_{17}H_{12}F_4$, 292.0875; found, 292.0872. The enantiomeric excess of **7am** was determined by HPLC analysis; DAICEL Chiralpak OJ-H, hexane/^{i}PrOH = 99/1, flow rate = 0.5 mL/min, λ = 220 nm, retention time: 11.9 min (major) and 16.0 min (minor), 90% ee.

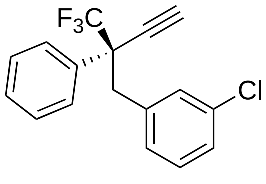

7an

(*R*)-1-Chloro-3-(2-phenyl-2-(trifluoromethyl)but-3-yn-1-yl)benzene (7an). A colorless oil (23.8 mg, 0.077 mmol, 77% yield based on the amount of **3a**). $[\alpha]_D^{20}$ = –13.5 (0.5 M in $CHCl_3$); ^{1}H NMR (400 MHz, $CDCl_3$): δ 7.66–7.61 (m, 2H), 7.40–7.34 (m, 3H), 7.13 (ddd, *J* = 8.0, 2.1, 0.9 Hz, 1H), 7.03 (t, *J* = 8.0, Hz, 1H), 7.00 (pseudo t, *J* = 2.0 Hz, 1H), 6.82 (d of pseudo t, *J* = 8.0, 1.4 Hz, 1H), 3.47 (d,

J = 13.8 Hz, 1H), 3.38 (d, J = 13.8 Hz, 1H), 2.70 (s, 1H); $^{13}C\{^1H\}$ NMR (100 MHz, $CDCl_3$): δ 136.3, 133.3, 133.0, 130.9, 128.8, 128.8, 128.4 (overlapping), 127.2, 125.5 (q, $^1J_{CF}$ = 282.7 Hz), 78.6 (q, $^3J_{CF}$ = 1.9 Hz), 78.4, 52.8 (q, $^2J_{CF}$ = 26.8 Hz), 40.4; ^{19}F NMR (376 MHz, $CDCl_3$): δ –73.5 (s); HRMS (FAB+) (m/z): $[M]^+$ calcd. for $C_{17}H_{12}ClF_3$, 308.0580; found, 308.0578. The enantiomeric excess of **7an** was determined by HPLC analysis; DAICEL Chiralpak OJ-H, hexane/^{i}PrOH = 99/1, flow rate = 0.5 mL/min, λ = 220 nm, retention time: 22.4 min (major) and 33.0 min (minor), 91% ee.

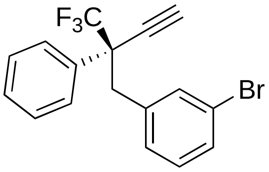

7ao

(*R*)-1-Bromo-3-(2-phenyl-2-(trifluoromethyl)but-3-yn-1-yl)benzene (7ao). A colorless oil (25.4 mg, 0.072 mmol, 72% yield based on the amount of **3a**). $[\alpha]_D^{20}$ = –44.5 (0.5 M in $CHCl_3$); ^1H NMR (400 MHz, $CDCl_3$): δ 7.65–7.61 (m, 2H), 7.39–7.35 (m, 3H,), 7.29 (ddd, J = 8.0, 2.0, 0.9 Hz, 1H), 7.16 (t, J = 2.0 Hz, 1H), 6.97 (t, J = 8.0 Hz, 1H), 6.86 (d of pseudo t, J = 8.0, 1.4 Hz, 1H), 3.46 (d, J = 13.6 Hz, 1H), 3.37 (d, J = 13.6 Hz, 1H), 2.70 (s, 1H). $^{13}C\{^1H\}$ NMR (100 MHz, $CDCl_3$) : δ 136.6, 133.8, 133.0, 130.1, 129.3, 129.1, 128.8, 128.4, 128.4, 125.5 (q, $^1J_{C\text{-}F}$ = 282.7 Hz), 121.5, 78.6 (q, $^3J_{CF}$ = 1.9 Hz), 78.5, 52.9 (q, $^2J_{CF}$ = 26.8 Hz), 40.4; ^{19}F NMR (376 MHz, $CDCl_3$): δ –73.4 (s); HRMS (FAB+) (m/z): $[M]^+$ calcd. for $C_{17}H_{12}BrF_3$, 352.0074; found, 352.0073. The enantiomeric excess of **7ao** was determined by HPLC analysis; DAICEL Chiralpak OJ-H, hexane/^{i}PrOH = 99/1, flow rate = 0.5 mL/min, λ = 220 nm, retention time: 19.5 min (major) and 27.8 min (minor), 93% ee.

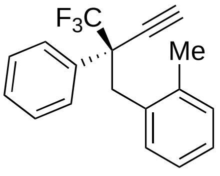

7ap

(*R*)-1-Methyl-2-(2-phenyl-2-(trifluoromethyl)but-3-yn-1-yl)benzene (7ap). A colorless oil (21.3 mg, 0.074 mmol, 74% yield based on the amount of **3a**). $[\alpha]_D^{20}$ = –50.8 (0.5 M in $CHCl_3$); ^1H NMR (400 MHz, $CDCl_3$): δ 7.68–7.64 (m, 2H), 7.39–7.35 (m, 3H), 7.10 (dd, J = 8.0, 2.0 Hz,1H), 7.07 (pseudo t of d J = 7.8, 1.5 Hz, 1H), 6.88 (pseudo t of d, J = 7.2, 2.0 Hz, 1H), 6.68 (d, J = 7.6 Hz, 1H), 3.61 (d, J = 14.4 Hz, 1H), 3.44 (d, J = 14.4 Hz, 1H), 2.55 (s, 1H), 2.25 (s, 3H). $^{13}C\{^1H\}$ NMR (100 MHz, $(CD_3)_2CO$): δ 138.3, 134.6, 133.6, 130.7, 130.6, 129.2, 129.1, 128.9, 127.3, 126.7 (q, $^1J_{CF}$ = 282.1 Hz), 125.4, 79.4, 79.2 (q, $^3J_{CF}$ = 1.9 Hz), 52.9 (q, $^2J_{CF}$ = 26.9 Hz), 36.7, 19.9 (^{13}C NMR spectrum of this compound was measured in acetone-d_6 because some peaks of this compound overlapped with those of $CDCl_3$); ^{19}F NMR (376 MHz, $CDCl_3$): δ –73.5 (s); HRMS (FAB+) (m/z): $[M]^+$ calcd. for $C_{18}H_{15}F_3$, 288.1126; found, 288.1112. The enantiomeric excess of **7ap** was determined by HPLC analysis; DAICEL Chiralpak OJ-H, hexane/^{i}PrOH = 99/1, flow rate = 0.5 mL/min, λ = 220 nm, retention time: 14.4 min (major) and 20.0 min (minor), 90% ee.

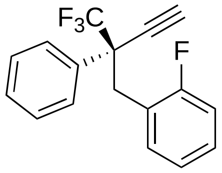

7aq

(*R*)-1-Fluoro-2-(2-phenyl-2-(trifluoromethyl)but-3-yn-1-yl)benzene (7aq). A colorless oil (22.8 mg, 0.078 mmol, 78% yield based on the amount of **3a**). $[\alpha]_D^{20}$ = –47.0 (0.5 M in $CHCl_3$); ^{1}H NMR (400 MHz, $CDCl_3$): δ 7.71–7.67 (m, 2H), 7.38–7.33 (m, 3H), 7.18–7.11 (m, 1H), 7.02–6.85 (m, 3H), 3.62 (d, *J* = 13.6 Hz, 1H), 3.51 (d, *J* = 13.6 Hz, 1H), 2.64 (s, 1H); $^{13}C\{^1H\}$ NMR (100 MHz, $CDCl_3$): δ 161.4 (d, $^1J_{CF}$ = 245.3 Hz), 133.4, 132.0 (d, $^3J_{CF}$ = 3.8 Hz), 128.8 (d, $^3J_{CF}$ = 8.6 Hz), 128.6, 128.4, 128.2, 125.6 (q, $^1J_{CF}$ = 282.7 Hz), 123.2 (d, $^4J_{CF}$ = 3.8 Hz), 121.8 (d, $^2J_{CF}$ = 14.4 Hz), 115.0 (d, $^2J_{CF}$ = 23.0 Hz), 78.8 (q, $^3J_{CF}$ = 1.9 Hz), 77.6, 52.3 (q, $^2J_{CF}$ = 26.8 Hz), 33.1; ^{19}F NMR (376 MHz, $CDCl_3$): δ –73.4 (s), –116.8 (s); HRMS (FAB+) (*m*/*z*): $[M]^+$ calcd. for $C_{17}H_{12}F_4$, 292.0875; found, 292.0865. The enantiomeric excess of **7aq** was determined by HPLC analysis; DAICEL Chiralpak OJ-H, hexane/^{i}PrOH = 99/1, flow rate = 0.5 mL/min, λ = 220 nm, retention time: 15.6 min (major) and 18.7 min (minor), 93% ee.

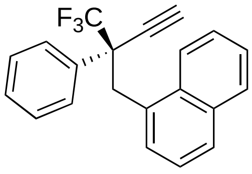

7ar

(*R*)-1-(2-Phenyl-2-(trifluoromethyl)but-3-yn-1-yl)naphthalene (7ar). A white solid (22.4 mg, 0.069 mmol, 69% yield based on the amount of **3a**). mp: 83.6–84.2 °C; $[\alpha]_D^{20}$ = +26.9 (0.5 M in $CHCl_3$); ^{1}H NMR (400 MHz, $CDCl_3$): δ 8.11–8.06 (m, 1H), 7.82–7.77 (m, 1H), 7.70–7.66 (m, 3H), 7.47–7.41 (m, 2H), 7.37–7.33 (m, 2H), 7.17 (pseudo t, *J* = 7.8 Hz, 1H), 6.94 (d, *J* = 6,8 Hz, 1H), 4.16 (d, *J* = 14.4 Hz, 1H), 3.89 (d, *J* = 14.4 Hz, 1H), 2.41 (s, 1H); $^{13}C\{^1H\}$ NMR (100 MHz, $CDCl_3$): δ 134.0, 133.5, 133.0, 130.5, 128.6, 128.5, 128.4 (overlapping), 128.3, 127.6, 125.9 (q, $^1J_{CF}$ = 283.0 Hz), 125.5, 125.3, 124.7, 124.2, 79.2 (q, $^3J_{CF}$ = 2.0 Hz), 78.0, 52.4 (q, $^2J_{CF}$ = 26.5 Hz), 36.1; ^{19}F NMR (376 MHz, $CDCl_3$): δ –71.9 (s); HRMS (FAB+) (*m*/*z*): $[M]^+$ calcd. for $C_{21}H_{15}F_3$, 324.1126; found, 324.1132. The enantiomeric excess of **7ar** was determined by HPLC analysis; DAICEL Chiralpak OD, hexane/^{i}PrOH = 99/1, flow rate = 0.5 mL/min, λ = 220 nm, retention time: 10.4 min (minor) and 12.9 min (major), 91% ee.

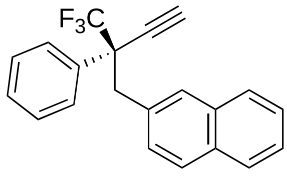

7as

(*R*)-2-(2-Phenyl-2-(trifluoromethyl)but-3-yn-1-yl)naphthalene (7as). A colorless oil (23.0 mg, 0.071 mmol, 71% yield based on the amount of **3a**). $[\alpha]_D^{20}$ = +37.9 (0.5 M in $CHCl_3$); ^{1}H NMR (400 MHz, $CDCl_3$): δ 7.74 (dd, J = 5.6, 3.3 Hz, 1H), 7.71–7.67 (m, 2H), 7.65 (dd, J = 6.3, 3.3 Hz, 1H), 7.75–7.62 (m, 4H), 7.58 (d, *J* = 8.3 Hz, 1H), 7.47 (br, 1H), 7.41 (d, *J* = 5.6 H, 1H), 7.40 (d, *J* = 6.3 Hz,

1H), 7.38–7.33 (m, 3H), 7.07 (dd, J = 8.3, 1.8 Hz, 1H), 3.69 (d, J = 13.4 Hz, 1H), 3.60 (d, J = 13.4 Hz, 1H), 2.66 (s, 1H); $^{13}C\{^1H\}$ NMR (100 MHz, $CDCl_3$): δ 133.4, 132.9, 132.4, 131.9, 129.9, 128.7, 128.6, 128.5, 128.3, 127.7, 127.4, 126.9, 125.7, 125.7 (q, $^1J_{CF}$ = 282.7 Hz), 125.7, 79.0 (q, $^3J_{CF}$ = 1.9 Hz), 78.2, 53.1 (q, $^2J_{CF}$ = 26.5 Hz), 40.9; ^{19}F NMR (376 MHz, $CDCl_3$): δ –73.4 (s); HRMS (FAB+) (m/z): $[M]^+$ calcd. for $C_{21}H_{15}F_3$, 324.1126; found: 324.1115. The enantiomeric excess of **7as** was determined by HPLC analysis; DAICEL Chiralpak OJ-H, hexane/^{i}PrOH = 99/1, flow rate = 0.5 mL/min, λ = 220 nm, retention time: 22.3 min (minor) and 26.3 min (major), 95% ee.

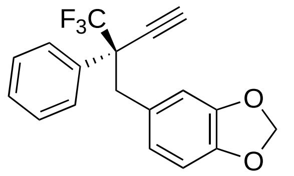

7at

(*R*)-5-(2-Phenyl-2-(trifluoromethyl)but-3-yn-1-yl)benzo[d][1,3]dioxole (7at). A colorless oil (21.0 mg, 0.066 mmol, 66% yield based on the amount of **3a**). $[\alpha]_D^{20}$ = +52.5 (0.5 M in $CHCl_3$); ^1H NMR (400 MHz, $CDCl_3$): δ 7.67–7.64 (m, 2H), 7.40–7.34 (m, 3H), 6.57 (d, J = 8.1 Hz, 1H), 6.49 (d, J = 1.3 Hz, 1H), 6.44 (dd, J = 8.1, 2.2 Hz, 1H), 5.86 (dd, J = 2.2, 1.3 Hz, 1H), 3.43 (d, J = 13.6 Hz, 1H), 3.34 (d, J = 13.6 Hz, 1H), 2.68 (s, 1H); $^{13}C\{^1H\}$ NMR (100 MHz, $CDCl_3$): δ 146.8, 146.5, 133.3, 128.6, 128.4, 128.3, 127.9, 125.6 (q, $^1J_{CF}$ = 283.0 Hz), 124.2, 111.0, 107.5, 100.8, 79.0, 78.1, 53.1 (q, $^2J_{CF}$ = 26.2 Hz), 40.5; ^{19}F NMR (376 MHz, $CDCl_3$): δ –73.4 (s); HRMS (FAB+) (m/z): $[M]^+$ calcd. for $C_{18}H_{13}F_3O_2$, 318.0868; found, 318.0865. The enantiomeric excess of **7at** was determined by HPLC analysis; DAICEL Chiralpak OJ-H, hexane/^{i}PrOH = 90/10, flow rate = 1.0 mL/min, λ = 220 nm, retention time: 22.7 min (minor) and 48.1 min (major), 91% ee.

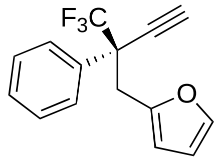

7au

(*R*)-2-(2-Phenyl-2-(trifluoromethyl)but-3-yn-1-yl)furan (7au). A colorless oil (19.7 mg, 0.080 mmol, 80% yield based on the amount of **3a**). $[\alpha]_D^{20}$ = –16.2 (0.5 M in $CHCl_3$); ^1H NMR (400 MHz, $CDCl_3$): δ 7.64–7.60 (m, 2H), 7.44–7.35 (m, 4H,), 6.42 (d, J = 3.2 Hz, 1H), 6.00-5.99 (m, 1H), 6.73-6.72 (m, 1H), 6.42 (d, J = 3.2 Hz, 1H), 6.00 (ddd, J = 3.2, 2.4, 0.8Hz, 1H), 2.74 (s, 1H), 2.29 (d, J = 0.8 Hz, 2H); $^{13}C\{^1H\}$ NMR (100 MHz, $CDCl_3$): δ 153.3, 146.0, 134.2, 128.8, 128.5, 128.3, 124.1 (q, $^1J_{CF}$ = 284.6 Hz), 111.0, 106.4, 78.2 (q, $^3J_{CF}$ = 1.9 Hz), 75.7, 52.3 (q, $^2J_{CF}$ = 29.7 Hz), 13.6; ^{19}F NMR (376 MHz, $CDCl_3$): δ –72.2 (s); HRMS (FAB+) (m/z): $[M]^+$ calcd. for $C_{15}H_{11}F_3O$, 264.0762; found, 264.0752. The enantiomeric excess of **7au** was determined by HPLC analysis; DAICEL Chiralpak OJ-H, hexane/^{i}PrOH = 99/1, flow rate = 0.5 mL/min, λ = 220 nm, retention time: 21.0 min (major) and 27.2 min (minor), 90% ee.

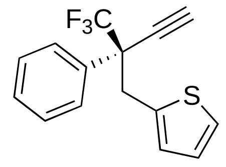

7av

(*R*)-2-(2-Phenyl-2-(trifluoromethyl)but-3-yn-1-yl)thiophene (7av). A colorless oil (21.0 mg, 0.075 mmol, 75% yield based on the amount of **3a**). $[\alpha]_D^{20}$ = –12.5 (0.5 M in $CHCl_3$); ^{1}H NMR (400 MHz, $CDCl_3$): δ 7.71–7.67 (m, 2H), 7.42–7.36 (m, 3H,), 7.03 (dd, *J* = 5.1, 1.2 Hz, 1H), 6.80 (dd, *J* = 5.1, 3.7 Hz, 1H), 6.72 (d, *J* = 3.7 Hz, 1H), 3.80 (d, *J* = 14.8 Hz, 1H), 3.68 (d, *J* = 14.8 Hz, 1H), 2.66 (s, 1H); ^{13}C{^{1}H} NMR (100 MHz, $CDCl_3$): δ 135.8, 132.8, 128.8, 128.6, 128.4, 128.2, 126.0, 125.3 (q, $^1J_{CF}$ = 282.7 Hz), 124.9, 78.9 (q, $^3J_{CF}$ = 1.9 Hz), 77.7, 52.9 (q, $^2J_{CF}$ = 27.2 Hz), 35.4; ^{19}F NMR (376 MHz, $CDCl_3$): δ –74.1 (s); HRMS (FAB+) (*m*/*z*): $[M]^+$ calcd. for $C_{15}H_{11}F_3S$, 280.0534; found, 280.0522. The enantiomeric excess of **7av** was determined by HPLC analysis; DAICEL Chiralpak OJ-H, hexane/^{i}PrOH = 99/1, flow rate = 0.5 mL/min, λ = 220 nm, retention time: 15.4 min (major) and 22.9 min (minor), 94% ee.

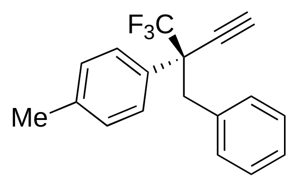

7ba

(*R*)-1-(2-Benzyl-1,1,1-trifluorobut-3-yn-2-yl)-4-methylbenzene (7ba). A colorless oil (23.4 mg, 0.081 mmol, 81% yield based on the amount of **3b**). $[\alpha]_D^{20}$ = –44.6 (0.5 M in $CHCl_3$); ^{1}H NMR (400 MHz, $CDCl_3$): δ 7.53 (d, *J* = 8.0 Hz, 2H), 7.18–7.10 (m, 5H), 7.00 (d, *J* = 8.0, 1.6 Hz, 2H), 3.50 (d, *J* = 13.4 Hz, 1H), 3.40 (d, *J* = 13.4 Hz, 1H), 2.63 (s, 1H), 2.35 (s, 3H); ^{13}C{^{1}H} NMR (100 MHz, $CDCl_3$): δ 138.4, 134.5, 130.8, 130.3, 129.0, 128.3, 127.5, 126.9, 125.7 (q, $^1J_{CF}$ = 282.7 Hz), 79.1 (q, $^3J_{CF}$ = 2.0 Hz), 77.8, 52.6 (q, $^2J_{CF}$ = 26.5 Hz), 40.6, 21.0; ^{19}F NMR (376 MHz, $CDCl_3$): δ –72.1 (s); HRMS (FAB+) (*m*/*z*): $[M]^+$ calcd. for $C_{18}H_{15}F_3$, 288.1126; found, 288.1123. The enantiomeric excess of **7ba** was determined by HPLC analysis; DAICEL Chiralpak OJ-H, hexane/^{i}PrOH = 99/1, flow rate = 0.5 mL/min, λ = 220 nm, retention time: 15.1 min (major) and 22.2 min (minor), 90% ee.

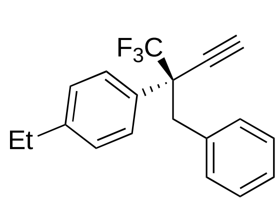

7ca

(*R*)-1-(2-Benzyl-1,1,1-trifluorobut-3-yn-2-yl)-4-ethylbenzene (7ca). A colorless oil (23.0 mg, 0.076 mmol, 76% yield based on the amount of **3c**). $[\alpha]_D^{20}$ = –47.4 (0.5 M in $CHCl_3$); ^{1}H NMR (400 MHz, $CDCl_3$): δ 7.56 (d, *J* = 7.6 Hz, 2H), 7.21–7.10 (m, 5H), 7.01 (dd, *J* = 8.0, 1.6 Hz, 2H), 3.51 (d, *J* = 13.2 Hz, 1H), 3.41 (d, *J* = 13.2 Hz, 1H), 2.66 (q, *J* = 7.7 Hz, 2H), 2.63 (s, 1H), 1.25 (t, *J* = 7.7 Hz, 3H); ^{13}C{^{1}H} NMR (100 MHz, $CDCl_3$): δ 144.6, 134.6, 130.8, 130.6, 128.4, 127.7, 127.5, 126.9, 125.7 (q, $^1J_{CF}$ = 282.7 Hz), 79.2 (q, $^3J_{CF}$ = 2.9 Hz), 77.8, 52.6 (q, $^2J_{CF}$ = 26.5 Hz), 40.7, 28.3, 15.2; ^{19}F NMR (376 MHz, $CDCl_3$): δ –73.6 (s); HRMS (FAB+) (*m*/*z*): $[M]^+$ calcd. for $C_{19}H_{17}F_3$, 302.1282; found, 302.1280. The enantiomeric excess of **7ca** was determined by HPLC analysis; DAICEL Chiralpak

OJ-H, hexane/^{i}PrOH = 99/1, flow rate = 0.5 mL/min, λ = 220 nm, retention time: 11.7 min (major) and 14.6 min (minor), 90% ee.

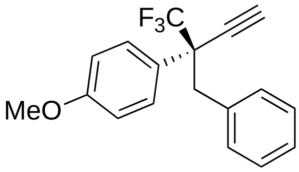

7da

(*R*)-1-(2-Benzyl-1,1,1-trifluorobut-3-yn-2-yl)-4-methylbenzene (7da). A colorless oil (21.6 mg, 0.071 mmol, 71% yield based on the amount of **3d**). $[\alpha]_D^{20}$ = –43.6 (0.5 M in $CHCl_3$); ^{1}H NMR (400 MHz, $CDCl_3$): δ 7.58 (d, *J* = 9.0 Hz, 2H), 7.20–7.11 (m, 3H), 7.01 (dd, *J* = 7.6, 1.6 Hz, 2H), 6.89 (d, *J* = 9.0 Hz, 2H), 3.82 (s, 3H), 3.50 (d, *J* = 13.4 Hz, 1H), 3.40 (d, *J* = 13.4 Hz, 1H), 2.63 (s, 1H); ^{13}C{^{1}H} NMR (100 MHz, $CDCl_3$): δ 159.6, 134.5, 130.8, 129.7, 127.6, 126.9, 125.7 (q, $^1J_{CF}$ = 282.7 Hz), 125.2, 113.5, 79.2 (q, $^3J_{CF}$ = 2.0 Hz), 77.8, 55.2, 52.3 (q, $^2J_{CF}$ = 26.5 Hz), 40.6; ^{19}F NMR (376 MHz, $CDCl_3$): δ –74.0 (s); HRMS (FAB+) (*m*/*z*): $[M+H]^+$ calcd. for $C_{18}H_{16}F_3O$, 304.1153; found: 305.1140. The enantiomeric excess of **7da** was determined by HPLC analysis; DAICEL Chiralpak OJ-H, hexane/^{i}PrOH = 90/10, flow rate = 0.5 mL/min, λ = 220 nm, retention time: 23.2 min (major) and 34.6 min (minor), 91% ee.

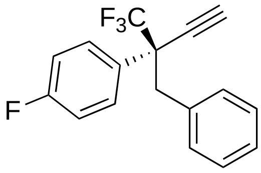

7ea

(*R*)-1-(2-Benzyl-1,1,1-trifluorobut-3-yn-2-yl)-4-fluorobenzene (7ea). A colorless oil (22.5 mg, 0.077 mmol, 77% yield based on the amount of **3e**). $[\alpha]_D^{20}$ = –58.8 (0.5 M in $CHCl_3$); ^{1}H NMR (400 MHz, $CDCl_3$): δ 7.62 (dd, *J* = 8.6, 5.0 Hz, 2H), 7.20–7.10 (m, 3H), 7.04 (pseudo t, *J* = 8.4 Hz, 2H), 6.98 (dd, *J* = 8.0, 1.6 Hz, 2H), 3.46 (d, *J* = 13.2 Hz, 1H), 3.41 (d, *J* = 13.2 Hz, 1H), 2.67 (s, 1H); ^{13}C{^{1}H} NMR (100 MHz, $CDCl_3$): δ 162.7 (d, $^1J_{CF}$ = 247.3 Hz), 134.1, 130.7, 130.4 (d, $^3J_{CF}$ = 7.7 Hz), 129.1 (d, $^4J_{CF}$ = 2.9 Hz), 127.7, 127.1, 125.6 (q, $^1J_{CF}$ = 282.7 Hz), 115.2 (d, $^2J_{CF}$ = 21.1 Hz), 78.8 (q, $^3J_{CF}$ = 2.9 Hz), 78.2, 52.5 (q, $^2J_{CF}$ = 26.7 Hz), 40.8; ^{19}F NMR (376 MHz, $CDCl_3$): δ –73.8 (s), –115.1 (s); HRMS (FAB+) (*m*/*z*): $[M+H]^+$ calcd. for $C_{17}H_{13}F_4$, 293.0953; found, 293.0945. The enantiomeric excess of **7ea** was determined by HPLC analysis; DAICEL Chiralpak OD, hexane/^{i}PrOH = 99/1, flow rate = 0.5 mL/min, λ = 220 nm, retention time: 9.0 min (major) and 10.0 min (minor), 94% ee.

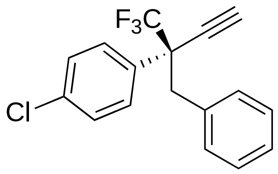

7fa

(*R*)-1-(2-Benzyl-1,1,1-trifluorobut-3-yn-2-yl)-4-chlorobenzene (7fa). A colorless oil (22.8 mg, 0.074 mmol, 74% yield based on the amount of **3f**). $[\alpha]_D^{20}$ = –54.2 (0.5 M in $CHCl_3$); ^{1}H NMR (400 MHz, $CDCl_3$): δ 7.58 (d, *J* = 8.8 Hz, 2H), 7.33 (d, *J* = 8.8 Hz, 2H), 7.21–7.11 (m, 3H), 6.99 (dd, *J* = 8.0, 1.6 Hz, 2H), 3.47 (d, *J* = 13.6 Hz, 1H), 3.42 (d, *J* = 13.6 Hz, 1H), 2.68 (s, 1H); ^{13}C{^{1}H} NMR

(100 MHz, $CDCl_3$): δ 134.7, 134.0, 131.9, 130.7, 129.9, 128.5, 127.7, 127.1, 125.5 (q, $^{1}J_{CF}$ = 282.7 Hz), 78.6 (q, $^{3}J_{CF}$ = 1.9 Hz), 78.4, 52.6 (q, $^{2}J_{CF}$ = 26.8 Hz), 40.6; ^{19}F NMR (376 MHz, $CDCl_3$): δ –72.0 (s); HRMS (FAB+) (*m*/*z*): $[M]^+$ calcd. for $C_{17}H_{12}ClF_3$, 308.0580; found: 308.0590. The enantiomeric excess of **7fa** was determined by HPLC analysis; DAICEL Chiralpak OJ-H, hexane/^{i}PrOH = 99/1, flow rate = 0.5 mL/min, λ = 220 nm, retention time: 15.2 min (major) and 16.6 min (minor), 90% ee.

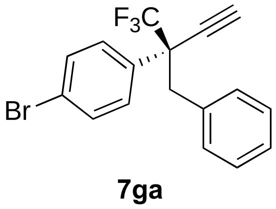

7ga

(*R*)-1-(2-Benzyl-1,1,1-trifluorobut-3-yn-2-yl)-4-bromobenzene (7ga). A white solid (24.7 mg, 0.070 mmol, 70% yield based on the amount of **3g**). mp: 49.2–59.3 °C; $[\alpha]_D^{20}$ = –20.3 (0.5 M in $CHCl_3$); ^{1}H NMR (400 MHz, $CDCl_3$): δ 7.53–7.46 (m, 4H), 7.20–7.11 (m, 3H), 6.98 (dd, *J* = 8.0, 1.6 Hz, 2H), 3.46 (d, *J* = 13.2 Hz, 1H), 3.41 (d, *J* = 13.2 Hz, 1H), 2.67 (s, 1H); $^{13}C\{^{1}H\}$ NMR (100 MHz, $CDCl_3$): δ 133.9, 132.5, 131.4, 130.7, 130.3, 127.7, 127.2, 125.4 (q, $^{1}J_{CF}$ = 283.0 Hz), 123.0, 78.5 (q, $^{3}J_{CF}$ = 1.9 Hz), 78.4, 52.7 (q, $^{2}J_{CF}$ = 26.8 Hz), 40.6; ^{19}F NMR (376 MHz, $CDCl_3$): δ –73.7 (s); HRMS (FAB+) (*m*/*z*): $[M]^+$ calcd. for $C_{17}H_{12}BrF_3$, 352.0074; found: 352.0083. The enantiomeric excess of **7ga** was determined by HPLC analysis; DAICEL Chiralpak OD, hexane/^{i}PrOH = 99/1, flow rate = 0.5 mL/min, λ = 220 nm, retention time: 8.8 min (major) and 9.8 min (minor), 92% ee.

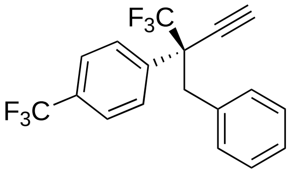

7ha

(*R*)-1-(2-Benzyl-1,1,1-trifluorobut-3-yn-2-yl)-4-(trifluoromethyl)benzene (7ha). A colorless oil (28.4 mg, 0.083 mmol, 83% yield based on the amount of **3h**). $[\alpha]_D^{20}$ = –50.2 (0.5 M in $CHCl_3$); ^{1}H NMR (400 MHz, $CDCl_3$): δ 7.78 (d, *J* = 8.0 Hz, 2H), 7.61 (d, *J* = 8.0 Hz, 2H), 7.21–7.10 (m, 3H), 6.97 (dd, *J* = 7.6, 1.4 Hz, 2H), 3.50 (d, *J* = 13.6 Hz, 1H), 3.46 (d, *J* = 13.6 Hz, 1H), 2.72 (s, 1H); $^{13}C\{^{1}H\}$ NMR (100 MHz, $CDCl_3$): δ 137.5, 133.7, 130.7, 129.0 128.4 (q, $^{2}J_{CF}$ = 12.8 Hz), 127.8, 127.3, 125.4 (q, $^{1}J_{CF}$ = 283.0 Hz), 125.2 (q, $^{3}J_{CF}$ = 3.5 Hz), 123.8 (q, $^{1}J_{CF}$ = 270.9 Hz), 78.8, 78.3 (q, $^{3}J_{CF}$ = 1.9 Hz), 53.0 (q, $^{2}J_{CF}$ = 26.8 Hz), 40.7; ^{19}F NMR (376 MHz, $CDCl_3$): δ –64.3 (s), –73.3 (s); HRMS (FAB+) (*m*/*z*): $[M]^+$ calcd. for $C_{18}H_{12}F_6$, 342.0843; found, 342.0847. The enantiomeric excess of **7ha** was determined by HPLC analysis; DAICEL Chiralpak OD, hexane/^{i}PrOH = 99/1, flow rate = 0.5 mL/min, λ = 220 nm, retention time: 9.7 min (major) and 10.7 min (minor), 93% ee.

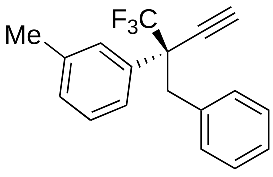

7ia

(*R*)-1-(2-Benzyl-1,1,1-trifluorobut-3-yn-2-yl)-3-methylbenzene (7ia). A colorless oil (19.3 mg, 0.067 mmol, 67% yield based on the amount of **3i**). $[\alpha]_D^{20}$ = –49.4 (0.5 M in $CHCl_3$); ^{1}H NMR (400

MHz, $CDCl_3$): δ 7.47–7.44 (br, 2H), 7.25 (pseudo t, J = 8.0 Hz, 1H), 7.21–7.10 (m, 4H), 6.99 (dd, J = 8.0, 1.6 Hz, 2H), 3.51 (d, J = 13.6 Hz, 1H), 3.40 (d, J = 13.6 Hz, 1H), 2.64 (s, 1H), 2.36 (s, 3H); $^{13}C\{^1H\}$ NMR (100 MHz, $CDCl_3$): δ 137.9, 134.5, 133.2, 130.8, 129.3, 129.2, 128.1, 127.5, 126.9, 125.7 (q, $^1J_{CF}$ = 282.7 Hz), 125.4, 79.1 (q, $^3J_{CF}$ = 2.0 Hz), 78.0, 52.9 (q, $^2J_{CF}$ = 26.2 Hz), 40.7, 21.6; ^{19}F NMR (376 MHz, $CDCl_3$): δ –73.4 (s); HRMS (FAB+) (m/z): $[M+H]^+$ calcd. for $C_{18}H_{16}F_3$, 289.1204; found, 289.1203. The enantiomeric excess of **7ia** was determined by HPLC analysis; DAICEL Chiralpak OJ-H, hexane/^{i}PrOH = 99/1, flow rate = 0.5 mL/min, λ = 220 nm, retention time: 13.9 min (major) and 15.4 min (minor), 90% ee.

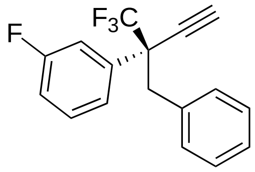

7ja

(*R*)-1-(2-Benzyl-1,1,1-trifluorobut-3-yn-2-yl)-3-fluorobenzene (7ja). A colorless oil (21.0 mg, 0.072 mmol, 72% yield based on the amount of **3j**). $[\alpha]_D^{20}$ = –18.5 (0.5 M in $CHCl_3$); ^1H NMR (400 MHz, $CDCl_3$): δ 7.44 (br d, J = 8.0 Hz, 1H), 7.40–7.30 (m, 2H), 7.20–7.10 (m, 3H), 7.05 (pseudo t of dd, J = 8.3, 2.8, 0.9 Hz, 1H), 6.98 (d, J = 8.0, 1.6 Hz, 2H), 3.47 (d, J = 13.4 Hz, 1H, 3.42 (d, J = 13.4 Hz, 1H), 2.68 (s, 1H); $^{13}C\{^1H\}$ NMR (100 MHz, $CDCl_3$): δ 162.5 (d, $^1J_{CF}$ = 245.3 Hz), 136.0 (d, $^3J_{CF}$ = 7.7 Hz), 133.9, 130.7, 129.7 (d, $^3J_{CF}$ = 7.7 Hz), 127.7, 127.2, 125.0 (q, $^1J_{CF}$ = 282.7 Hz), 124.1, 116.1 (d, $^2J_{CF}$ = 24.0 Hz), 115.6 (d, $^2J_{CF}$ = 21.1 Hz), 78.4, 77.2, 52.9 (q, $^2J_{CF}$ = 27.1 Hz), 40.8; ^{19}F NMR (376 MHz, $CDCl_3$): δ –73.4 (s), –113.8 (s); HRMS (FAB+) (m/z): $[M]^+$ calcd. for $C_{17}H_{12}F_4$, 292.0875; found, 292.0878. The enantiomeric excess of **7ja** was determined by HPLC analysis; DAICEL Chiralpak OJ-H, hexane/^{i}PrOH = 99/1, flow rate = 0.5 mL/min, λ = 220 nm, retention time: 11.9 min (major) and 16.0 min (minor), 90% ee.

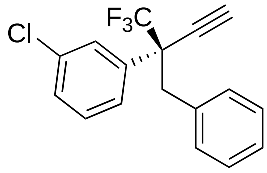

7ka

(*R*)-1-(2-Benzyl-1,1,1-trifluorobut-3-yn-2-yl)-3-chlorobenzene (7ka). A colorless oil (20.1 mg, 0.065 mmol, 65% yield based on the amount of **3k**). $[\alpha]_D^{20}$ = –50.0 (0.5 M in $CHCl_3$); ^1H NMR (400 MHz, $CDCl_3$): δ 7.64 (br, 1H), 7.54 (d, J = 7.2 Hz, 1H), 7.33 (dt, J = 8.0, 1.8 Hz, 1H), 7.29 (pseudo t, J = 7.4 Hz, 1H), 7.21–7.11 (m, 3H), 6.99 (dd, J = 7.6, 1.2 Hz, 1H), 3.47 (d, J = 13.6 Hz, 1H), 3.42 (d, J = 13.6 Hz, 1H), 2.69 (s, 1H); $^{13}C\{^1H\}$ NMR (100 MHz, $CDCl_3$): δ 135.5, 134.3, 133.9, 130.7, 129.4, 128.9, 128.8, 127.7, 127.2, 126.6, 125.4 (q, $^1J_{CF}$ = 283.0 Hz,), 78.6, 78.4 (q, $^3J_{CF}$ = 2.0 Hz), 52.9 (q, $^2J_{CF}$ = 24.8 Hz), 40.7; ^{19}F NMR (376 MHz, $CDCl_3$): δ –73.4 (s); HRMS (FAB+) (m/z): $[M]^+$ calcd. for $C_{17}H_{12}ClF_3$, 308.0580; found: 308.0576. The enantiomeric excess of **7ka** was determined by HPLC analysis; DAICEL Chiralpak OJ-H, hexane/^{i}PrOH = 99/1, flow rate = 0.5 mL/min, λ = 220 nm, retention time: 27.9 min (major) and 35.2 min (minor), 95% ee.

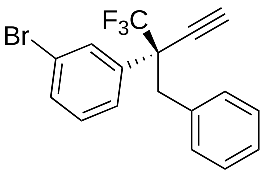

7la

(*R*)-1-(2-Benzyl-1,1,1-trifluorobut-3-yn-2-yl)-3-bromobenzene (7la). A colorless oil (24.7 mg, 0.070 mmol, 70% yield based on the amount of **3l**). $[\alpha]_D^{20}$ = –45.9 (0.5 M in $CHCl_3$); ^{1}H NMR (400 MHz, $CDCl_3$): δ 7.79 (br, 1H), 7.58(d of pseudo quint, *J* = 8.0, 0.9 Hz, 1H), 7.49 (ddd, *J* = 8.0, 1.8, 0.9 Hz, 1H,), 7.23 (t, *J* = 8.0 Hz, 1H), 7.19–7.11 (m, 3H), 6.99 (dd, *J* = 8.0, 1.6 Hz, 1H), 3.46 (d, *J* = 13.4 Hz, 1H), 3.41 (d, *J* = 13.4 Hz, 1H), 2.69 (s, 1H); ^{13}C{^{1}H} NMR (100 MHz, $CDCl_3$): δ 135.7, 133.8, 131.8, 131.7, 130.7, 129.7, 127.7, 127.2, 127.1, 125.4 (q, $^1J_{CF}$ = 283.0 Hz,), 122.5, 78.7, 78.3 (q, $^3J_{CF}$ = 1.9 Hz), 52.8 (q, $^2J_{CF}$ = 26.5 Hz), 40.7; ^{19}F NMR (376 MHz, $CDCl_3$): δ –73.4 (s); HRMS (FAB+) (*m*/*z*): $[M]^+$ calcd. for $C_{17}H_{12}BrF_3$, 352.0074; found, 352.0070. The enantiomeric excess of **7la** was determined by HPLC analysis; DAICEL Chiralpak OJ-H, hexane/^{i}PrOH = 99/1, flow rate = 0.5 mL/min, λ = 220 nm, retention time: 14.1 min (minor) and 18.4 min (major), 90% ee.

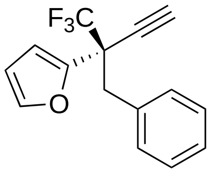

7ma

(*R*)-2-(2-Benzyl-1,1,1-trifluorobut-3-yn-2-yl)furan (7ma). A colorless oil (20.1 mg, 0.076 mmol, 76% yield based on the amount of **3m**). $[\alpha]_D^{20}$ = –11.0 (0.5 M in $CHCl_3$); ^{1}H NMR (400 MHz, $CDCl_3$): δ 7.50 (dd, *J* = 2.0, 0.8 Hz, 1H), 7.21–7.15 (m, 3H,), 6.97 (dd, *J* = 7.6, 1.8 Hz, 2H), 6.40 (dd, *J* = 3.6, 0.8, Hz, 1H), 6.32 (dd, *J* = 3.6 Hz, 2.0 Hz, 1H), 3.61 (d, *J* = 12.8 Hz, 1H), 3.22 (d, *J* = 12.8 Hz, 1H), 2.47 (s, 1H); ^{13}C{^{1}H} NMR (100 MHz, CD_3CN): δ 147.2, 144.4, 134.6, 130.9, 128.2, 127.7, 125.2 (q, $^1J_{CF}$ = 282.7 Hz), 112.8, 111.2, 77.5, 76.9 (q, $^3J_{CF}$ = 1.9 Hz), 49.7 (q, $^2J_{CF}$ = 28.1 Hz), 38.7; ^{19}F NMR (376 MHz, $CDCl_3$): δ –74.7 (s). HRMS (FAB+) (*m*/*z*): $[M]^+$ calcd. for $C_{15}H_{11}F_3O$, 264.0762; found, 264.0758. The enantiomeric excess of **7ma** was determined by HPLC analysis; DAICEL Chiralpak OJ-H, hexane/^{i}PrOH = 99/1, flow rate = 0.5 mL/min, λ = 220 nm, retention time: 13.2 min (minor) and 16.0 min (major), 96% ee.

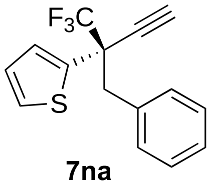

7na

(*S*)-2-(2-Benzyl-1,1,1-trifluorobut-3-yn-2-yl)thiophene (7na). A colorless oil (18.2 mg, 0.065 mmol, 65% yield based on the amount of **3n**). $[\alpha]_D^{20}$ = –51.6 (0.5 M in $CHCl_3$); ^{1}H NMR (400 MHz, $CDCl_3$): δ 7.30 (dd, *J* = 4.9, 1.4 Hz, 1H), 7.22–7.14 (m, 4H,), 7.04 (dd, *J* = 8.0 1.6 Hz, 1H), 6.98 (dd, *J* = 4.9, 3.6 Hz, 1H), 3.41 (d, *J* = 12.8 Hz, 1H), 3.36 (d, *J* = 12.8 Hz, 1H), 2.60 (s, 1H); ^{13}C{^{1}H} NMR (100 MHz, CD_3CN): δ 137.3, 134.0, 130.6, 128.3, 127.6, 127.2, 126.8, 126.3, 125.1 (q, $^1J_{CF}$ = 282.7 Hz), 78.5, 76.8, 50.5 (q, $^2J_{CF}$ = 28.1 Hz), 42.6 (^{13}C NMR spectrum of this compound was measured in acetonitrile-d_6 because some peaks of this compound overlapped with those of $CDCl_3$); ^{19}F NMR (376 MHz, $CDCl_3$): δ –75.1 (s); HRMS (FAB+) (*m*/*z*): $[M]^+$ calcd. for $C_{15}H_{11}F_3S$, 280.0534; found, 280.0527. The enantiomeric excess of **7na** was determined by HPLC analysis; DAICEL Chiralpak

OJ-H, hexane/^{i}PrOH = 99/1, flow rate = 0.5 mL/min, λ = 220 nm, retention time: 18.4 min (minor) and 23.6 min (major), 90% ee.

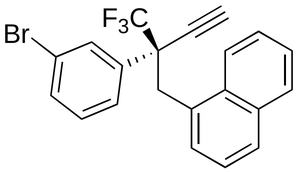

7lr

(*R*)-1-(2-(3-Bromophenyl)-2-(trifluoromethyl)but-3-yn-1-yl)naphthalene (7lr). A white solid (24.6 mg, 0.061 mmol, 61% yield based on the amount of **3l**). mp: 95.2–96.4 °C; $[\alpha]_D^{20}$ = +36.9 (0.5 M in $CHCl_3$); ^{1}H NMR (400 MHz, $CDCl_3$): δ 8.08–8.02 (m, 1H), 7.85–7.79 (m, 2H), 7.71 (d, *J* = 8.0 Hz, 1H), 7.59 (d, *J* = 7.6 Hz, 1H), 7.51–7.41 (m, 3H), 7.22 (pseudo t, *J* = 7.0 Hz, 1H), 7.20 (pseudo t, *J* = 8.0 Hz, 1H), 6.97 (d, *J* = 7.2 Hz, 1H), 4.14 (d, *J* = 14.4 Hz, 1H), 3.85 (d, *J* = 14.4 Hz, 1H), 2.45 (s, 1H); ^{13}C{^{1}H} NMR (100 MHz, $CDCl_3$): δ 136.3, 133.6, 132.9, 131.8, 131.6, 130.0, 129.8, 128.6, 128.4, 127.9, 126.9, 125.6 (q, $^1J_{CF}$ = 283.0 Hz), 125.5, 125.4, 124.7, 124.1, 122.6, 78.7, 78.5 (q, $^3J_{CF}$ = 1.9 Hz), 52.3 (q, $^2J_{CF}$ = 26.8 Hz), 36.2; ^{19}F NMR (376 MHz, $CDCl_3$): δ –71.6 (s); HRMS (FAB+) (*m*/*z*): $[M]^+$ calcd. for $C_{21}H_{14}BrF_3$, 402.0231; found, 402.0240. The enantiomeric excess of **7lr** was determined by HPLC analysis; DAICEL Chiralpak OD, hexane/^{i}PrOH = 99/1, flow rate = 0.5 mL/min, λ = 220 nm, retention time: 13.6 min (minor) and 18.9 min (major), 91% ee. Colorless needle crystals suitable for X-ray analysis were obtained by recrystallization from Et_2O, after additional purification of (*R*)-isomer by HPLC.

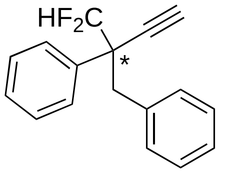

9

(*R*)-(2-(Difluoromethyl)but-3-yne-1,2-diyl)dibenzene (9). A colorless oil (19.2 mg, 0.075 mmol, 75% yield based on the amount of **8**). $[\alpha]_D^{20}$ = –32.7 (0.5 M in $CHCl_3$); ^{1}H NMR (400 MHz, $CDCl_3$): δ 7.61 (dd, *J* = 8.0, 1.6 Hz, 2H), 7.40–7.31 (m, 3H), 7.22–7.15 (m, 3H), 7.07 (dd, *J* = 7.2, 2.4 Hz, 2H), 5.95 (t, *J* = 56.2 Hz, 1H), 3.39 (d, *J* = 13.2 Hz, 1H), 3.31 (dd, *J* = 13.2, 0.8 Hz, 1H), 2.62 (s, 1H); ^{13}C{^{1}H} NMR (100 MHz, $CDCl_3$): δ 135.2, 134.9, 130.7, 128.3, 128.2, 128.1, 127.7, 126.9, 116.5 (t, $^1J_{CF}$ = 250.1 Hz), 80.8 (dd, $^3J_{CF}$ = 5.7, 3.8 Hz), 77.5, 51.1 (t, $^2J_{CF}$ = 20.1 Hz), 41.2 (t, $^3J_{CF}$ = 2.9 Hz); ^{19}F NMR (376 MHz, $CDCl_3$): δ –123.3 (dd, $^2J_{FF}$ = 268 Hz, $^2J_{HF}$ = 54 Hz), –126.5 (dd, dd, $^2J_{FF}$ = 268 Hz, $^2J_{HF}$ = 54 Hz); HRMS (FAB+) (*m*/*z*): $[M+H]^+$ calcd. for $C_{17}H_{15}F_2$, 257.1142; found, 257.1137. The enantiomeric excess of **9** was determined by HPLC analysis; DAICEL Chiralpak OJ-H, hexane/^{i}PrOH = 99/1, flow rate = 1.0 mL/min, λ = 220 nm, retention time: 19.4 min (major) and 30.4 min (minor), 63% ee.

1.5. Large-Scale Preparation of 7aa

In an oven dried 100 mL Schlenk flask were placed **[Ru]-2** (63.0 mg, 0.050 mmol) and NH_4BF_4 (11.0 mg, 0.10 mmol) under N_2. Anhydrous $ClCH_2CCH_2Cl$ (20 mL) was added, and then the mixture was magnetically stirred at room temperature for 60 min. Then, 1,1,1-trifluoro-2-phenylbut-3-yn-2-

ol (**3a**) (200 mg. 1.0 mmol), diethyl 4-benzyl-2,6-dimethyl-1,4-dihydropyridine-3,5-dicarboxylate (**4a**) (412.1 mg, 1.2 mmol), *fac*-[Ir(ppy)$_3$] (6.4 mg, 0.01 mmol), and $BF_3 \cdot Et_2O$ (148 μL, 170 mg, 1.20 mmol) were added under N_2 at room temperature. The reaction flask was placed in an As One LTB-125 constant low temperature water bath set at 25 °C, and was illuminated from the bottom of the bath with an Aitech System TMN100×120–22WD 12 W white LED lamp (400 nm to 750 nm) at a distance of approximately 2 cm from the light source for 48 h. The volatiles were removed *in vacuo*, and the residue was purified by column chromatography (SiO_2) with *n*-hexane as an eluent to afford (*R*)-(2-(trifluoromethyl)but-3-yne-1,2-diyl)dibenzene (**7aa**) as a colorless oil (205.5 mg, 0.75 mmol, 75% yield based on the amount of **3a**) with 93% ee.

1.6. Preparation of (*S*)-(4-bromo-2-(trifluoromethyl)but-3-yne-1,2-diyl)dibenzene (10)

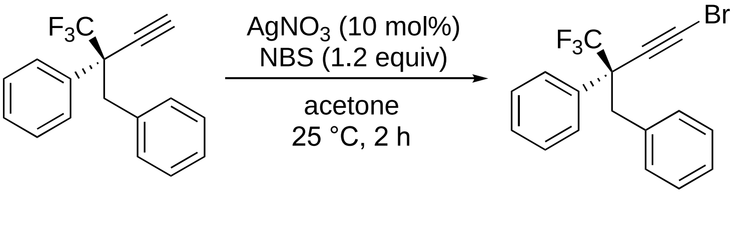

7aa
93% ee

10
95%, 93% ee

In a 20 mL Schlenk flask were placed **(*R*)-7aa** (27.4 mg. 0.10 mmol, 93% ee), *N*-bromosuccinimide (NBS) (21.4 mg, 0.12 mmol) and $AgNO_3$ (1.7 mg, 0.010 mmol) under N_2, where acetone (5.0 mL) was added at room temperature. And the mixture was stirred at room temperature for 2 h. The volatiles were then removed *in vacuo,* and the residue was purified by column chromatography (SiO_2) with *n*-hexane as an eluent to afford (*S*)-(4-bromo-2-(trifluoromethyl)but-3-yne-1,2-diyl)dibenzene (**10**) as a colorless oil (33.6 mg, 0.95 mmol, 95% yield based on the amount of **7aa**). $[\alpha]_D^{20}$ = –41.3 (0.5 M in $CHCl_3$); ^{1}H NMR (400 MHz, $CDCl_3$): δ 7.60–7.56 (m, 2H), 7.38–7.33 (m, 3H), 7.19–7.11 (m, 3H), 6.95 (dd, *J* = 7.8, 1.4 Hz, 2H), 3.49 (d, *J* = 13.4 Hz, 1H), 3.42 (d, *J* = 13.4 Hz, 1H); $^{13}C\{^1H\}$ NMR (100 MHz, $CDCl_3$): δ 134.3, 133.5, 130.7, 128.6, 128.4, 128.3, 127.6, 127.0, 125.5 (q, $^1J_{CF}$ = 283.0 Hz), 75.5, 54.2 (q, $^2J_{CF}$ = 26.5 Hz), 49.6, 41.0; ^{19}F NMR (376 MHz, $CDCl_3$): δ –73.2 (s); HRMS (FAB+) (*m*/*z*): $[M]^+$ calcd. for $C_{17}H_{12}BrF_3$, 352.0074; found: 352.0080. The enantiomeric excess of **10** was determined by HPLC analysis; DAICEL Chiralpak OJ-H, hexane/^{i}PrOH = 100/0, flow rate = 0.5 mL/min, λ = 220 nm, retention time: 16.0 min (major) and 19.4 min (minor), 93% ee.

1.7. Preparation of (*S*)-(2-(trifluoromethyl)but-3-yne-1,2,4-triyl)tribenzene (11)

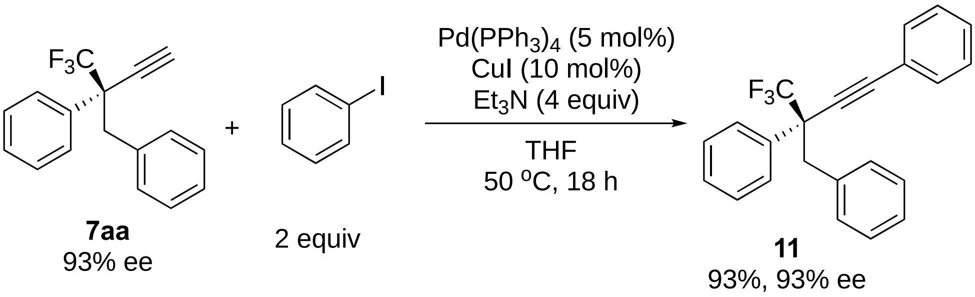

7aa
93% ee

2 equiv

11
93%, 93% ee

In a 20 mL Schlenk flask were placed **(*R*)-7aa** (27.4 mg. 0.10 mmol, 93% ee), iodobenzene (40.8 mg, 0.20 mmol), Pd(PPh3)4 (5.8 mg, 0.0050 mmol), CuI (1.9 mg, 0.010 mmol), and Et_3N (56 μL, 0.40 mmol) under N_2, where THF (2.0 mL) was added at room temperature. And the mixture was stirred at 50 °C for 18 h. The volatiles were then removed *in vacuo,* and the residue was purified by column

chromatography (SiO_2) with *n*-hexane as an eluent to afford (*S*)-(2-(trifluoromethyl)but-3-yne-1,2,4-triyl)tribenzene (**11**) as a white solid. (32.6 mg, 0.93 mmol, 93% yield based on the amount of **7aa**). mp: 57.0–58.5 °C; $[\alpha]_D^{20}$: –53.5 (0.5 M in $CHCl_3$); ^1H NMR (400 MHz, $CDCl_3$): δ 7.70 (br d, J = 8.0 Hz,2H), 7.45 (dd, J = 7.6, 2.0 Hz, 2H), 7.40–7.33 (m, 6H), 7.19–7.10 (m, 3H), 7.04 (dd, J = 7.6, 1.6 Hz, 2H), 3.58 (d, J = 13.2 Hz, 1H), 3.50 (d, J = 13.2 Hz, 1H); $^{13}C\{^1H\}$ NMR (100 MHz, $CDCl_3$): δ 134.8, 134.3, 131.7, 130.8, 128.7, 128.6, 128.4, 128.3, 128.3, 127.5, 126.9, 125.9 (q, $^1J_{CF}$ = 283.3 Hz), 122.2, 89.6, 84.5, 53.6 (q, $^2J_{CF}$ = 26.5 Hz), 41.2; ^{19}F NMR (376 MHz, $CDCl_3$): δ –73.2; HRMS (FAB+) (*m*/*z*): $[M]^+$ calcd. for $C_{23}H_{17}F_3$, 350.1282; found: 350.1289. The enantiomeric excess of **11** was determined by HPLC analysis; DAICEL Chiralpak OJ-H, hexane/^{i}PrOH = 99/1, flow rate = 0.5 mL/min, λ = 220 nm, retention time: 8.0 min (major) and 9.5 min (minor), 93% ee.

1.8. Preparation of (*S*)-(2-(trifluoromethyl)butane-1,2-diyl)dibenzene (12).

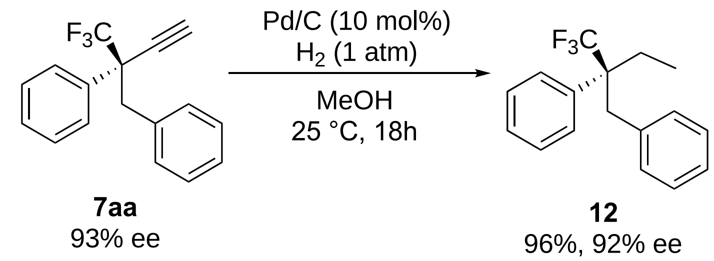

In a 20 mL Schlenk flask were placed **(*R*)-7aa** (27.4 mg. 0.10 mmol, 93% ee), and Pd/C (10.6 mg, 0.01 mmol, Palladium 10% on Carbon) under N_2, where MeOH (4.0 mL) was added at room temperature. After substitution of N_2 atmosphere to H_2 atmosphere by a hydrogen balloon, the mixture was stirred at room temperature for 18 h. The volatiles were then removed *in vacuo*, and the residue was purified by column chromatography (SiO_2) with *n*-hexane as an eluent to afford (*S*)-(2-(trifluoromethyl)butane-1,2-diyl)dibenzene (**12**) as a colorless oil. (26.7 mg, 0.96 mmol, 96% yield based on the amount of **7aa**). $[\alpha]_D^{20}$ = –50.3 (0.5 M in $CHCl_3$); ^1H NMR (400 MHz, $CDCl_3$) δ 7.41–7.29 (m, 5H), 7.20–7.09 (m, 3H), 6.80 (dd, J = 7.6, 1.6 Hz, 2H), 3.22 (s, 2H), 2.01 (qq, J = 7.5, 0.7, Hz, 2H), 0.96 (tq, J = 7.5, 1.4 Hz, 3H); $^{13}C\{^1H\}$ NMR (100 MHz, $CDCl_3$): δ 137.1, 135.7, 130.7, 128.6 (q, $^1J_{C\text{-}F}$ = 284.6 Hz), 128.2 (overlapping), 127.7, 127.4, 126.6, 52.6 (q, $^2J_{CF}$ = 21.7 Hz), 42.0, 25.0, 8.9; ^{19}F NMR (376 MHz, $CDCl_3$): δ –68.4; HRMS (FAB+) (*m*/*z*): $[M]^+$ calcd. for $C_{17}H_{17}F_3$, 278.1282; found, 278.1293. The enantiomeric excess of **12** was determined by HPLC analysis; DAICEL Chiralpak OJ-H, hexane/^{i}PrOH = 99/1, flow rate = 0.5 mL/min, λ = 220 nm, retention time: 11.5 min (minor) and 36.1 min (major), 92% ee.

1.9. Preparation of (*R*)-(2-(trifluoromethyl)pent-3-ene-1,2,5-triyl)tribenzene (13)

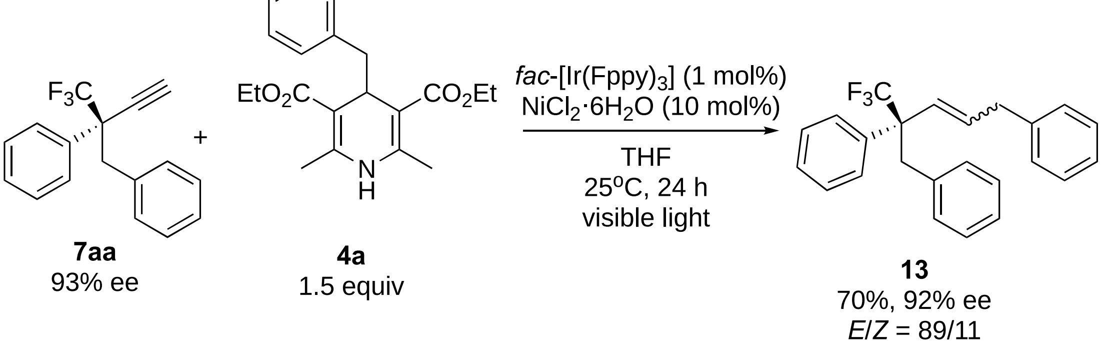

In a 20 mL Schlenk flask were placed **(*R*)-7aa** (27.4 mg. 0.10 mmol, 93% ee), **4a** (51.5 mg, 0.375mmol), *fac*-[Ir(Fppy)$_3$] (0.8 mg, 0.0011 mmol), and $NiCl_2 \cdot 6H_2O$ (2.4 mg, 0.020 mmol) under N_2,

where THF (2.0 mL) was added at room temperature. The reaction flask was placed in an As One LTB-125 constant low temperature water bath set at 25 °C, and was illuminated from the bottom of the bath with an Aitech System TMN100×120–22WD 12 W white LED lamp (400 nm to 750 nm) at a distance of approximately 2 cm from the light source for 24 h. *E*/*Z* ratio of (*R*)-(2-(trifluoromethyl)pent-3-ene-1,2,5-triyl)tribenzene in the crude mixture was determined to be 55/45 by quantitative measurement of GC-MS. The volatiles were then removed *in vacuo*, and the residue was purified by column chromatography (SiO_2) with *n*-hexane as an eluent to afford a mixture of (*R*,*E*)-(2-(trifluoromethyl)pent-3-ene-1,2,5-triyl)tribenzene ((*E*)-**13**) and (*R*,*Z*)-(2-(trifluoromethyl)pent-3-ene-1,2,5-triyl)tribenzene ((*Z*)-**13**) as a colorless oil (*E*/*Z* = 89/11, 25.6 mg, 0.70 mmol, 70% yield based on the amount of **7aa**). $[\alpha]_D^{20}$ = –26.5 (0.5 M in $CHCl_3$); ^{1}H NMR (400 MHz, $CDCl_3$): (*E*)-**13**: δ 7.48–7.44 (m, 2H), 7.36–7.27 (m, 5H), 7.22 (t of pseudo t, *J* = 7.2, 1.7Hz, 1H), 7.18–7.07(m, 5H), 6.86 (dd, *J* = 8.0, 1.6 Hz, 2H), 5.93 (dtq, *J* = 16.0, 6.9, 1.6 Hz, 1H), 5.65 (dq, *J* = 16.0, 1.3 Hz,1H), 3.50 (d, *J* = 14.2 Hz, 1H), 3.47 (d, *J* = 6.9 Hz, 2H), 3.37 (d, *J* = 14.2 Hz, 1H); (*Z*)-**13** (^{1}H NMR resonances due to 11H of aromatic CH, 1H of CH=C*H*CH_2, and 3H of CH_2 overlapping with those of (*E*)-**13** (δ 7.48–7.06, 5.97–5.88, and 3.53–3.49, respectively): 6.95 (dd, *J* = 7.6, 1.6Hz, 2H), 6.90 (dd, *J* = 8.2, 1.4 Hz, 2H), 5.83 (dq, *J* = 12.0, 1.8 Hz, 1H), (m, 3H, 3H of two CH_2 resonances overlapping with those of (*E*)-**13**), 3.33 (d, *J* = 13.6 Hz, 1H); $^{13}C\{^1H\}$ NMR (100 MHz, $CDCl_3$, 100 MHz): (*E*)-**13**: δ 139.6, 137.4, 135.6, 133.3, 131.0, 129.2, 129.1, 128.6, 128.5, 128.0, 127.7, 127.6, 127.3 (q, $^1J_{CF}$ = 283.3 Hz), 126.5, 126.2, 55.2 (q, $^2J_{CF}$ = 23.0 Hz), 40.7, 39.5; (*Z*)-**13**: δ 139.7, 137.9, 136.2, 136.0, 131.3, 129.3, 128.8, 128.3, 128.1, 126.8, 126.6, 126.6 (q, $^1J_{CF}$ = 277.9 Hz), 126.0, 55.1 (q, $^2J_{CF}$ = 23.0 Hz), 42.9, 35.6; ^{19}F NMR (376 MHz, $CDCl_3$): δ –69.9; HRMS (FAB+) (*m*/*z*): $[M]^+$ calcd. for $C_{24}H_{21}F_3$, 366.1595; found, 366.1605. The enantiomeric excess of **13** was determined by HPLC analysis; DAICEL Chiralpak OJ-H, hexane/^{i}PrOH = 99/1, flow rate = 0.5 mL/min, λ = 220 nm, retention time: (*E*)-**13**: 13.8 min (minor) and 15.8 min (major), 92% ee; (*Z*)-**13**: 11.9 min (minor) and 18.3 min (major), 92% ee.

1.10. Preparation of 14

In an oven dried 20 mL Schlenk flask were placed **[Ru]-1** (30.0 mg, 0.026 mmol), NH_4BF_4 (5.5 mg, 0.052 mmol) under N_2. Anhydrous $ClCH_2CCH_2Cl$ (3.0 mL) was added, and then the mixture was magnetically stirred at room temperature. After the addition of **3a** (52.0 mg, 0.26 mmol) and $BF_3\cdot Et_2O$ (8 μL, 9 mg, 0.065 mmol), the mixture was stirred at 25 °C for 24 h. Then, the solvent was filtered to remove residual inorganic salts. The filtrate was concentrated under reduced pressure, where conversion of **[Ru]-1** into **14** (98% NMR yield) was confirmed by ^{1}H NMR by using 1,1,2,2-tetrachloroethane as an internal standard. The residue was washed with hexane (5 mL × 3) and Et_2O (5 mL × 3) to give reddish brown solid (**14**) in 71% yield (25.8 mg, 0.018 mmol) (Fig. 6a).[5]

1.11. Stoichiometric reaction of 14 with 4a

In an oven dried 20 mL Schlenk flask were placed **14** (138 mg, 0.10 mmol) and under N_2. Anhydrous $ClCH_2CCH_2Cl$ (2.0 mL) was added and then, 1,1,1-trifluoro-2-(*p*-tolyl)but-3-yn-2-ol (**3b**) (21.4 mg, 0.10 mmol), diethyl 4-benzyl-2,6-dimethyl-1,4-dihydropyridine-3,5-dicarboxylate (**4a**) (41.2 mg, 0.12 mmol), *fac*-[Ir(ppy)$_3$] (0.7 mg, 0.0011 mmol) were added under N_2 at room temperature. The reaction flask was placed in an As One LTB-125 constant low temperature water bath set at 25 °C, and was illuminated from the bottom of the bath with an Aitech System TMN100×120–22WD 12 W white LED lamp (400 nm to 750 nm) at a distance of approximately 2 cm from the light source for 48 h. The volatiles were removed *in vacuo*, and the residue was purified by column chromatography

(SiO_2) with *n*-hexane as an eluent to afford (*R*)-(2-(trifluoromethyl)but-3-yne-1,2-diyl)dibenzene (**7aa**) as a colorless oil (18.1 mg, 0.066 mmol, 66% yield based on the amount of **3a**) with 88% ee (Fig. 6b).

1.12. Catalytic reaction of 3a with 4a by using 14 as a catalyst

In an oven dried 20 mL Schlenk flask were placed **14** (6.9 mg, 0.0050 mmol) and under N_2. Anhydrous $ClCH_2CCH_2Cl$ (2.0 mL) was added, and then, 1,1,1-trifluoro-2-phenylbut-3-yn-2-ol (**3a**) (20.0 mg. 0.10 mmol), diethyl 4-benzyl-2,6-dimethyl-1,4-dihydropyridine-3,5-dicarboxylate (**4a**) (41.2 mg, 0.12 mmol), *fac*-[Ir(ppy)$_3$] (0.7 mg, 0.0011 mmol), and $BF_3{\cdot}Et_2O$ (15 µL, 17 mg, 0.12 mmol) were added under N_2 at room temperature. The reaction flask was placed in an As One LTB-125 constant low temperature water bath set at 25 °C, and was illuminated from the bottom of the bath with an Aitech System TMN100×120–22WD 12 W white LED lamp (400 nm to 750 nm) at a distance of approximately 2 cm from the light source for 48 h. The volatiles were removed *in vacuo*, and the residue was purified by column chromatography (SiO_2) with *n*-hexane as an eluent to afford (*R*)-(2-(trifluoromethyl)but-3-yne-1,2-diyl)dibenzene (**7aa**) as a colorless oil (23.3 mg, 0.085 mmol, 85% yield based on the amount of **3a**) with 85% ee (Fig. 6c).

1.13. Reactions in the presence of TEMPO

In an oven dried 20 mL Schlenk flask were placed **[Ru]-2** (6.3 mg, 0.0050 mmol) and NH_4BF_4 (1.1 mg, 0.010 mmol) under N_2. Anhydrous $ClCH_2CCH_2Cl$ (2.0 mL) was added, and then the mixture was magnetically stirred at room temperature for 30 min. Then, 1,1,1-trifluoro-2-phenylbut-3-yn-2-ol (**3a**) (20.0 mg. 0.10 mmol), diethyl 4-benzyl-2,6-dimethyl-1,4-dihydropyridine-3,5-dicarboxylate (**4a**) (41.2 mg, 0.12 mmol), *fac*-[Ir(ppy)$_3$] (0.7 mg, 0.0011 mmol), and $BF_3{\cdot}Et_2O$ (15 µL, 17 mg, 0.12 mmol) were added under N_2 at room temperature. The reaction flask was placed in an As One LTB-125 constant low temperature water bath set at 25 °C, and was illuminated from the bottom of the bath with an Aitech System TMN100×120–22WD 12 W white LED lamp (400 nm to 750 nm) at a distance of approximately 2 cm from the light source for 48 h. The volatiles were removed in vacuo, and the crude yield of 1-(benzyloxy)-2,2,6,6-tetramethylpiperidine (**15**)[20] (34% NMR yield) was determined by ^1H NMR in $CDCl_3$, where 1,1,2,2-tetrachloroethane (16.8 mg, 0.100 mmol) was added as an internal standard (Fig. 6d).

1.14. Deuterium labeling reaction

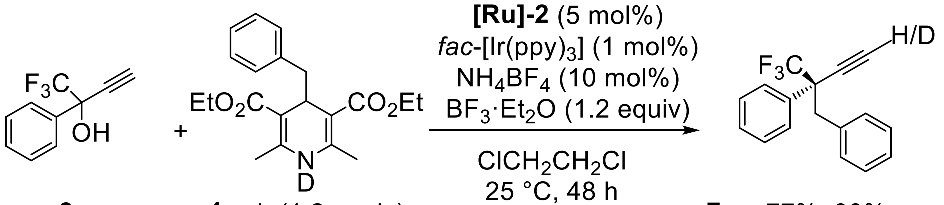

3a + **4a**-d_1 (1.2 equiv) → **7aa**, 77%, 93% ee, H/D = 70/30

In an oven dried 20 mL Schlenk flask were placed **[Ru]-2** (6.3 mg, 0.0050 mmol) and NH_4BF_4 (1.1 mg, 0.010 mmol) under N_2. Anhydrous $ClCH_2CCH_2Cl$ (2.0 mL) was added, and then the mixture was magnetically stirred at room temperature for 30 min. Then, 1,1,1-trifluoro-2-phenylbut-3-yn-2-ol (**3a**) (20.0 mg. 0.10 mmol), diethyl 4-benzyl-2,6-dimethyl-1-deuterium-4-hydropyridine-3,5-dicarboxylate (**4a**-d_1) (41.2 mg, 0.12 mmol), *fac*-[Ir(ppy)$_3$] (0.7 mg, 0.0011 mmol), and $BF_3{\cdot}Et_2O$ (15 µL, 17 mg, 0.12 mmol) were added under N_2 at room temperature. The reaction flask was

placed in an As One LTB-125 constant low temperature water bath set at 25 °C, and was illuminated from the bottom of the bath with an Aitech System TMN100×120–22WD 12 W white LED lamp (400 nm to 750 nm) at a distance of approximately 2 cm from the light source for 48 h. The volatiles were removed *in vacuo*, and the residue was purified by column chromatography (SiO_2) with *n*-hexane as an eluent to afford (*R*)-(2-(trifluoromethyl)but-3-yne-1,2-diyl)dibenzene (**7aa**) as a colorless oil (21.1 mg, 0.077 mmol, 77% yield based on the amount of **3a**) with 93% ee. The ratio of H/D is determined by ^{1}H NMR to be 70/30 at δ 2.66.

1.15. Stern–Volmer analysis

Luminescence quenching experiments for the THF solution of *fac*-[Ir(ppy)$_3$] (2.0 μmol/L, prepared by stepwise dilutions of *fac*-[Ir(ppy)$_3$] (1.3 mg, 2.0 μmol) with $ClCH_2CH_2Cl$) with **3a**, **4a** or **16** in selected concentrations were performed on a Shimadzu RF-5300PC spectrophotometer, where the solutions containing *fac*-[Ir(ppy)$_3$] and **3a**, **4a** or **16** were excited at λ_{max} = 440 nm, and emissions were measured at λ = 494 nm.

From the slopes (100.5 for **3a**, 208.3 for **4a** and 32.6 for **16**,) obtained by the plot and the excited-state lifetime of *fac*-[Ir(ppy)$_3$] (τ = 1.9 μs),[21] the rate constants were calculated to be at, $k_{3a} = (5.3 \pm 0.2) \times 10^7$ M^{-1} s^{-1} (**3a**), $k_{4a} = (1.10 \pm 0.05) \times 10^8$ M^{-1} s^{-1} (**4a**) and $k_{16} = (1.72 \pm 0.07) \times 10^7$ M^{-1} s^{-1} (**16**), respectively, by using the Stern–Volmer linear correlation relationship (Fig. 7a).[22]

1.16. Light on/off experiments

In an oven dried 20 mL Schlenk flask were placed **[Ru]-2** (6.3 mg, 0.0050 mmol) and NH_4BF_4 (1.1 mg, 0.010 mmol) under N_2. Anhydrous $ClCH_2CCH_2Cl$ (2.0 mL) was added, and then the mixture was magnetically stirred at room temperature for 30 min. Then, 1,1,1-trifluoro-2-phenylbut-3-yn-2-ol (**3a**) (20.0 mg. 0.10 mmol), diethyl 4-benzyl-2,6-dimethyl-1,4-dihydropyridine-3,5-dicarboxylate (**4a**) (41.2 mg, 0.12 mmol), *fac*-[Ir(ppy)$_3$] (0.7 mg, 0.0011 mmol), and $BF_3 \cdot Et_2O$ (15 μL, 17 mg, 0.12 mmol) were added under N_2 at room temperature. The reaction flask was placed in an As One LTB-125 constant low temperature water bath set at 25 °C. The reaction was conducted for 32 h under alternating periods of (1) irradiation from the bottom of the bath with an Aitech System TMN100×120–22WD 12 W white LED lamp (400 nm to 750 nm) at a distance of approximately 2 cm from the light source, and (2) darkness with the reaction vessel wrapped with aluminum foil, where yields of **7aa** were determined every 4 hours by quantitative measurements of gas chromatography–mass spectroscopy (GC-MS) recorded on a Shimadzu GCMS-QP2010 PLUS instrument, where *n*-octane was added as an internal standard (Fig. 7b).

1.17. Cyclic voltammetric studies

Cyclic voltammograms were recorded on an ALS/Chi model 610C electrochemical analyzer in $ClCH_2CH_2Cl$ containing 1 mM of sample and 0.1 M of $^{n}Bu_4NPF_6$ as a supporting electrolyte using glassy carbon working electrode and platinum wire counter electrode at a scan rate of 0.1 V/s at room temperature. All potentials were measured against $Ag/AgNO_3$ reference electrode (0.01 M $AgNO_3$, 0.1 M $^{n}NBu_4ClO_4$, MeCN) and converted to the values vs $FeCp_2^{+/0}$ (Cp = η^5-C_5H_5). The cyclic voltammograms of *fac*-[Ir(ppy)$_3$], **4a**, and **3a** in $ClCH_2CH_2Cl$ are shown in Supplementary Fig. 1.

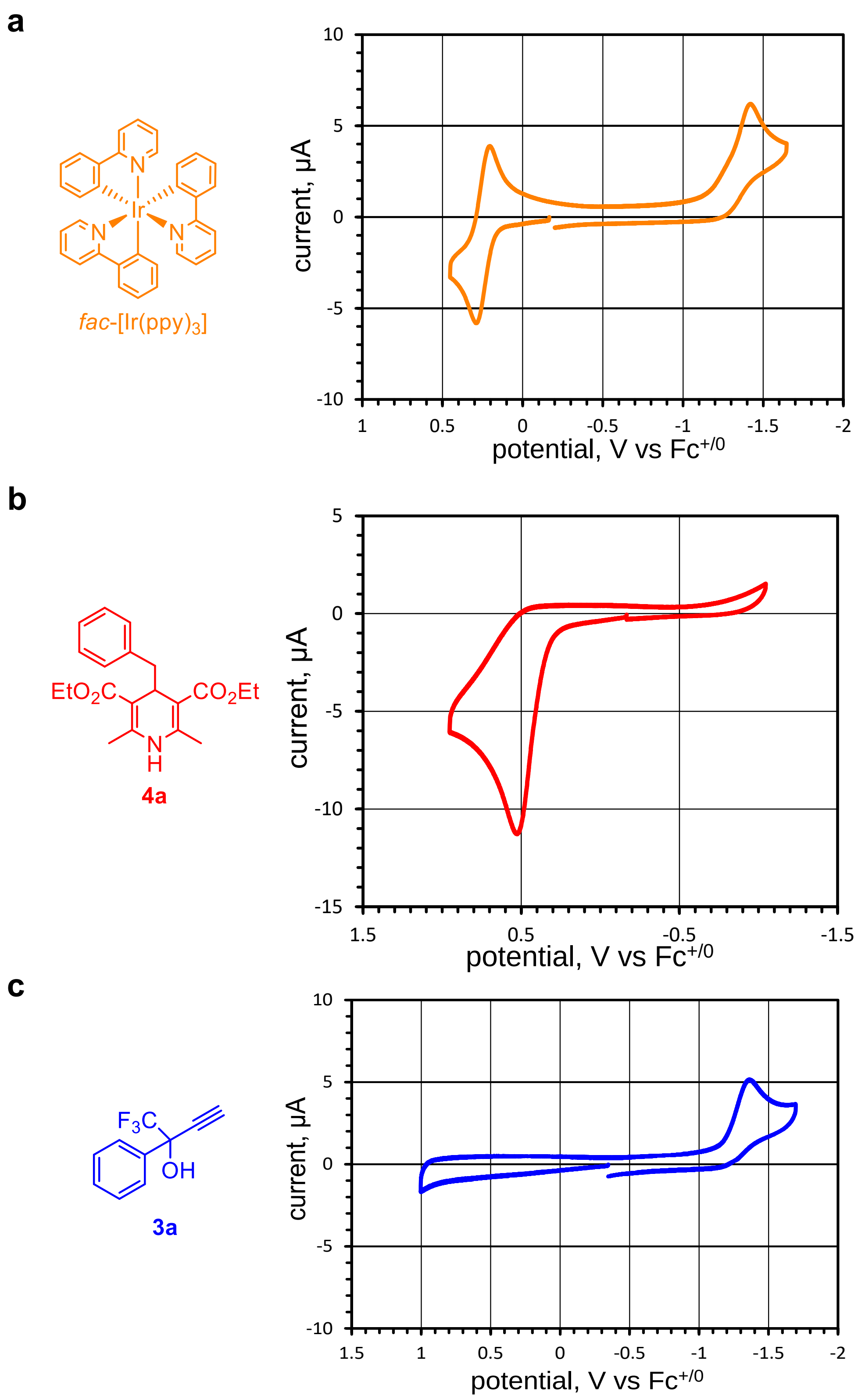

Supplementary Fig. 1: Cyclic voltammetric studies. **a** Cyclic voltammogram of *fac*-[Ir(ppy)$_3$] in $ClCH_2CH_2Cl$. $E_{1/2}(Ir/Ir^{-}) = -1.60$ V vs $FeCp_2^{+/0}$, $E_{pa}(Ir/Ir^{+}) = +0.25$ V vs $FeCp_2^{+/0}$. Therefore, $E_{pc}(Ir^{*}/Ir^{-}) = +0.90$ V vs $FeCp_2^{+/0}$, $E_{1/2}(Ir^{*/+}) = -2.25$ V vs $FeCp_2^{+/0}$, based on the emission energy of *fac*-[Ir(ppy)$_3$] at 2.50 eV.[23] **b** Cyclic voltammogram of **4a** in THF. E_{pa}(**4a**/**4a**$^{+\bullet}$) = +0.53 V vs $FeCp_2^{+/0}$. **c** Cyclic voltammogram of **3a** in $ClCH_2CH_2Cl$. E_{pc}(**3a**/**3a**$^{-\bullet}$) = –1.36 V vs $FeCp_2^{+/0}$.

1.18. Time profile experiment

In an oven dried 20 mL Schlenk flask were placed **[Ru]-2** (6.3 mg, 0.0050 mmol) and NH_4BF_4 (1.1 mg, 0.010 mmol) under N_2. Anhydrous $ClCH_2CCH_2Cl$ (2.0 mL) was added, and then the mixture was magnetically stirred at room temperature for 30 min. Then, 1,1,1-trifluoro-2-phenylbut-3-yn-2-ol (**3a**) (20.0 mg. 0.10 mmol), diethyl 4-benzyl-2,6-dimethyl-1,4-dihydropyridine-3,5-dicarboxylate (**4a**) (41.2 mg, 0.12 mmol), *fac*-[Ir(ppy)$_3$] (0.7 mg, 0.0011 mmol), and $BF_3{\cdot}Et_2O$ (15 µL, 17 mg, 0.12 mmol) were added under N_2 at room temperature. The reaction flask was placed in an As One LTB-125 constant low temperature water bath set at 25 °C, and was illuminated from the bottom of the bath with an Aitech System TMN100×120–22WD 12 W white LED lamp (400 nm to 750 nm) at a distance of approximately 2 cm from the light source. Yields of **7aa** at selected times (2, 4, 8, 12, 24 and 48 h) were determined by quantitative measurements of gas chromatography–mass spectroscopy (GC-MS) recorded on a Shimadzu GCMS-QP2010 PLUS instrument, where *n*-octane was added as an internal standard (Supplementary Fig. 2). The reaction rate was calculated as $k_{\mathbf{7aa}} = (2.33 \pm 0.04) \times 10^{-7}\ M^{-1}\ s^{-1}$.

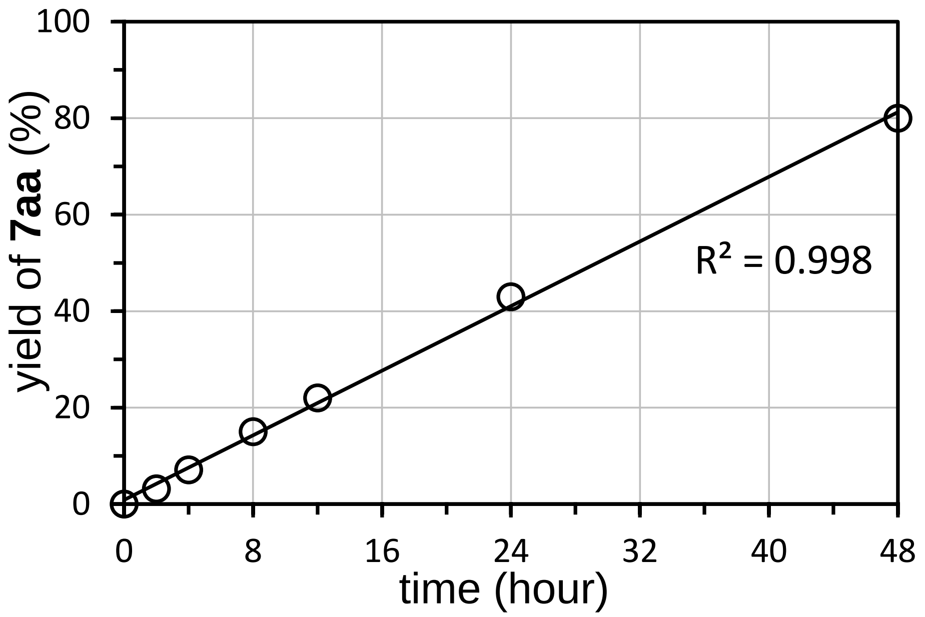

Supplementary Fig. 2: Time profile experiment.

1.19. Determination of quantum yields

In an oven dried 20 mL Schlenk flask were placed **[Ru]-2** (6.3 mg, 0.0050 mmol) and NH_4BF_4 (1.1 mg, 0.010 mmol) under N_2. Anhydrous $ClCH_2CCH_2Cl$ (2.0 mL) was added, and then the mixture was magnetically stirred at room temperature for 30 min. Then, 1,1,1-trifluoro-2-phenylbut-3-yn-2-ol (**3a**) (20.0 mg. 0.10 mmol), diethyl 4-benzyl-2,6-dimethyl-1,4-dihydropyridine-3,5-dicarboxylate (**4a**) (41.2 mg, 0.12 mmol), *fac*-[Ir(ppy)$_3$] (0.7 mg, 0.0011 mmol), and $BF_3{\cdot}Et_2O$ (15 µL, 17 mg, 0.12 mmol) were added under N_2 at room temperature. The reaction flask was illuminated from the side with an Ushio SX-U1251HQ ultrahigh pressure 250 W Hg lamp equipped with a 440-nm band pass filter (Kenko B440) at a distance of approximately 2 cm from the light source for 6 h. The volatiles were removed in vacuo, and the crude yield of **7aa** (0.00920 mmol, 9.2% NMR yield based on the amount of **3a** was determined by ^{1}H NMR in $CDCl_3$, where 1,1,2,2-tetrachloroethane (16.8 mg, 0.100 mmol) was added as an internal standard. Independently, the yields of **7aa** were

determined by terminating the reactions at 2 and 4 h, which clarified a zero-order reaction rate at 4.26 × 10^{-8} mol s^{-1}. The irradiated light intensity to a 2.5 mL solution in 50-mL Schlenk was estimated to be 8.50 × 10^{-7} E s^{-1} at 440 nm by using $K_3[Fe(C_2O_4)_3]$ as a chemical actinometer.[24] Thus, the quantum yield of the photoredox- and ruthenium-catalyzed reaction of **3a** with **4a** to afford propargylic alkylated product **7aa** is given as $\Phi = 0.0502 \pm 0.0003$ (Supplementary Fig. 3).

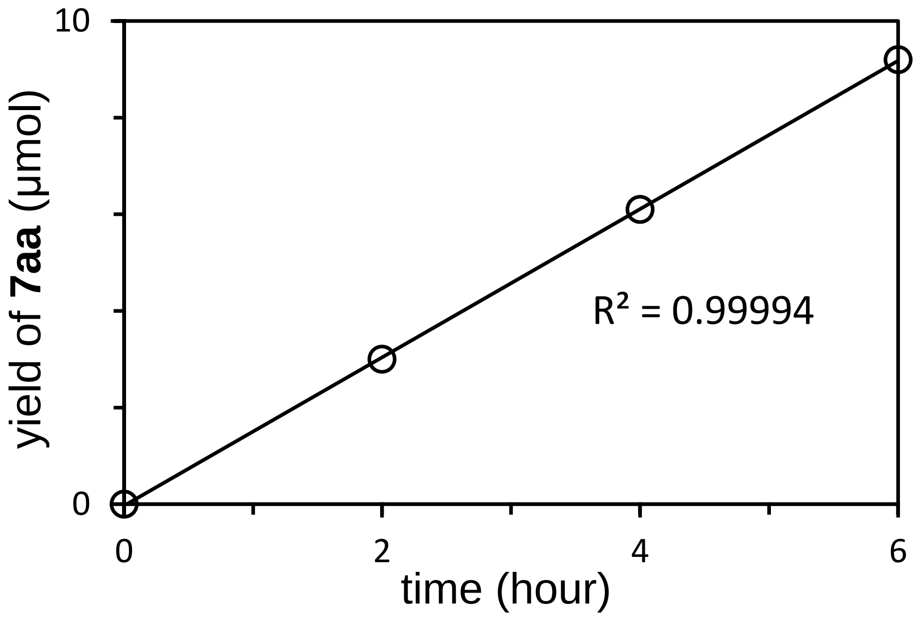

Supplementary Fig. 3: Quantum yield determination.

1.20. X-ray crystallographic study of (*R*)-1-(2-(3-bromophenyl)-1,1,1-trifluorobut-3-yn-2-yl)naphthalene (7lr)

Diffraction data for a crystal of **7lr** were collected for the 2θ range of 4.5° to 62.2° at –180 °C on a Rigaku XtaLAB Synergy-S diffractometer equipped with a HyPix-6000HE Hybrid Photon Counting (HPC) detector and VariMax optics using multi-layer mirror monochromated Mo-Kα (λ = 0.71073 Å) radiation, and VariMax optics. Intensity data were corrected for Lorentz and polarization effect and for empirical absorptions (CrysAlisPro),[25] while structure solutions and refinements were carried out by using CrystalStructure package.[26] Positions of non-hydrogen atoms were determined by direct methods (SHELXT Version 2014/5),[27] and subsequent Fourier syntheses (SHELXL Version 2016/6),[28] and were refined on F_o^2 with all the unique reflections by full-matrix least squares with anisotropic thermal parameters. All the hydrogen atoms were placed at the calculated positions with fixed isotropic parameters. Anomalous dispersion effects were included in F_c,[29] and mass attenuation coefficients, values for $\Delta f'$ and $\Delta f''$, and neutral atom scattering factors were taken from references.[30-32] The absolute configuration of **7lr** with a chiral center at the C(1) atom was determined to be (*R*), with the Flack parameter refined to be –0.005(5). Details of the crystal and data collection parameters of (*R*)-**7lr** are summarized in Supplementary Table 1. An ORTEP drawing of (*R*)-**7lr** is shown in Supplementary Fig. 4.

Supplementary Table 1 Crystallographic data for (*R*)-**7lr**

**compound**	(*R*)-**7lr**
chemical formula	$C_{21}H_{14}BrF_3$
CCDC number	2172478
formula weight	403.24
crystal size, mm^3	0.131 × 0.066 × 0.037
crystal color, habit	colorless, needle
temperature, °C	–180
crystal system	monoclinic
space group	$P2_1$ (no. 4)
a, Å	9.1554(4)
b, Å	7.1034(3)
c, Å	13.5438(6)
α, deg	90
β, deg	103.113(4)
γ, deg	90
V, Å^3	857.85(7)
Z	2
d_{calcd}, g, cm^{-3}	1.561
F(000)	404
μ, cm^{-1}	24.327
transmission factors range	0.847 – 0.914
number of measured reflections	13715
number of unique reflections	4344
number of refined parameters	226
R_{int}	0.0457
*R*1 ($I > 2\sigma(I)$) [a]	0.0297
*wR*2 (all data) [b]	0.0564
GOF [c]	1.000
maximum residual peak / hole, e Å^{-3}	+0.30 / –0.33
Flack parameter	–0.005(5)

[a] $R1 = \Sigma||F_o| - |F_c||/\Sigma|F_o|$. [b] $wR2 = [\Sigma\{w(F_o^2 - F_c^2)^2\}/\Sigma w(F_o^2)^2]^{1/2}$, $w = 1/[\sigma^2(F_o^2) + rP]$, $P = (\mathrm{Max}(F_o^2, 0) + 2F_c^2)/3$ [$r = 3.65$]. [c] $\mathrm{GOF} = [\Sigma w(F_o^2 - F_c^2)^2/(N_o - N_{params})]^{1/2}$.

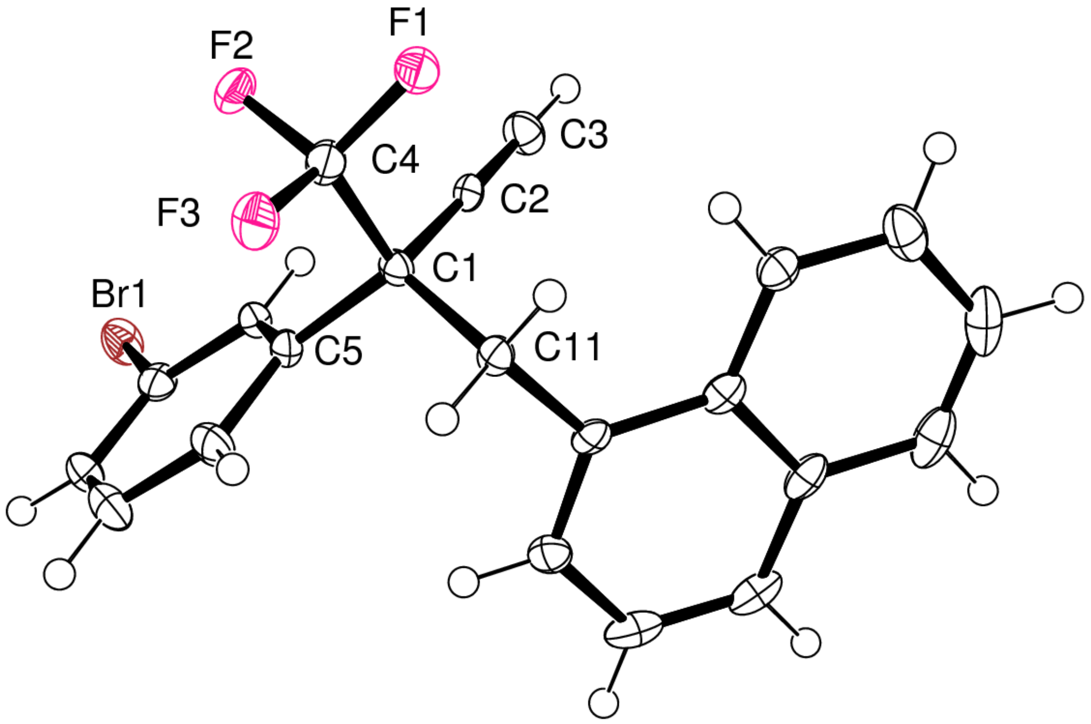

Supplementary Fig. 4: ORTEP drawing of (*R*)-**7lr**. Thermal ellipsoids are drawn at the 50% probability level. Selected bond lengths (Å) and angles (°): C(1)–C(2): 1.468(4), C(1)–C(4): 1.535(4), C(1)–C(5): 1.548(4), C(1)–C(11): 1.556(4), C(2)–C(3): 1.188(5); C(2)–C(1)–C(4):106.6(2), C(2)–C(1)–C(5): 111.3(2), C(4)–C1)–C(5): 106.9(2), C(2)–C1)–C(11): 110.4(3), C(4)–C1)–C(11): 108.1(2), C(5)–C1)–C(11): 113.2(2), C(3)–C2)–C(1): 178.4(3).

1.21. DFT calculations

DFT calculations were carried out with the Gaussian16 (Revision A.03) program package.[33] Geometry optimization and analytical vibrational frequency analysis were performed by ωB97XD Kohn-Sham DFT [34,35] (restricted KS method for singlet state and unrestricted KS method for doublet and triplet states).[35] In the numerical integration, a larger grid (*superfinegrid*) was used.[33] Pople's 6-311G** basis set[36] for C, H, S, and Cl atoms and the SDD basis set[37] with the effective core potential for Ru atom were used for the Gaussian basis functions (5d-type). The solvent effects of dichloroethane were estimated by the IEF-PCM method[38,39] for the gas phase optimized structures. For the IEF-PCM calculations, the ωB97XD functional was used with the larger basis set (Pople's 6-311++G** basis set[36] for C, H, N, O, S, and Cl atoms (5d-type) and the SDD basis set for Ru atom; ωB97XD(IEFPCM)/(SDD, 6-311++G**)//ωB97XD(IEFPCM)/(SDD, 6-311G**)). The Gibbs free energy at 298K was estimated by the IEF-PCM energy and the gas-phase thermal correction term (*T*). All the results are summarized in Supplementary Table 2 and Supplementary Figs. 5–8.

Supplementary Table 2 Total electronic energy *E* and Gibbs free energy $G^{298.15K}$ (a. u.)

	ωB97XD/(SDD, 6-311G**)		ωB97XD(IEFPCM)/(SDD, 6-311++G**) //ωB97XD/(SDD, 6-311G**)	
	E	*T*	*E*	$G^{298.15K}$
**I**	-2652.931215	0.577572	-2652.987526	-2652.409954
Bn·	-270.878749	0.085445	-270.884482	-270.799037
**II**	-2923.827178	0.683276	-2923.885964	-2923.202688
**TS$_{II-III}$**	-2923.824805	0.685582	-2923.882584	-2923.197002
**III**	-2923.871077	0.688734	-2923.937231	-2923.248497
**IV**	-2924.089301	0.688499	-2924.116165	-2923.427666
**V**	-2924.502562	0.703703	-2924.566060	-2923.862357
**VI**	-2924.163164	0.701287	-2924.351071	-2923.649784
$^2[Ir(ppy)_3]^-$	-1540.585524	0.417328	-1540.672281	-1540.254953
$^1[Ir(ppy)_3]$	-1540.580980	0.424514	-1540.608779	-1540.184265
PyH^+	-861.641565	0.252167	-861.717414	-861.465247
Py	-861.257404	0.238104	-861.277723	-861.039619

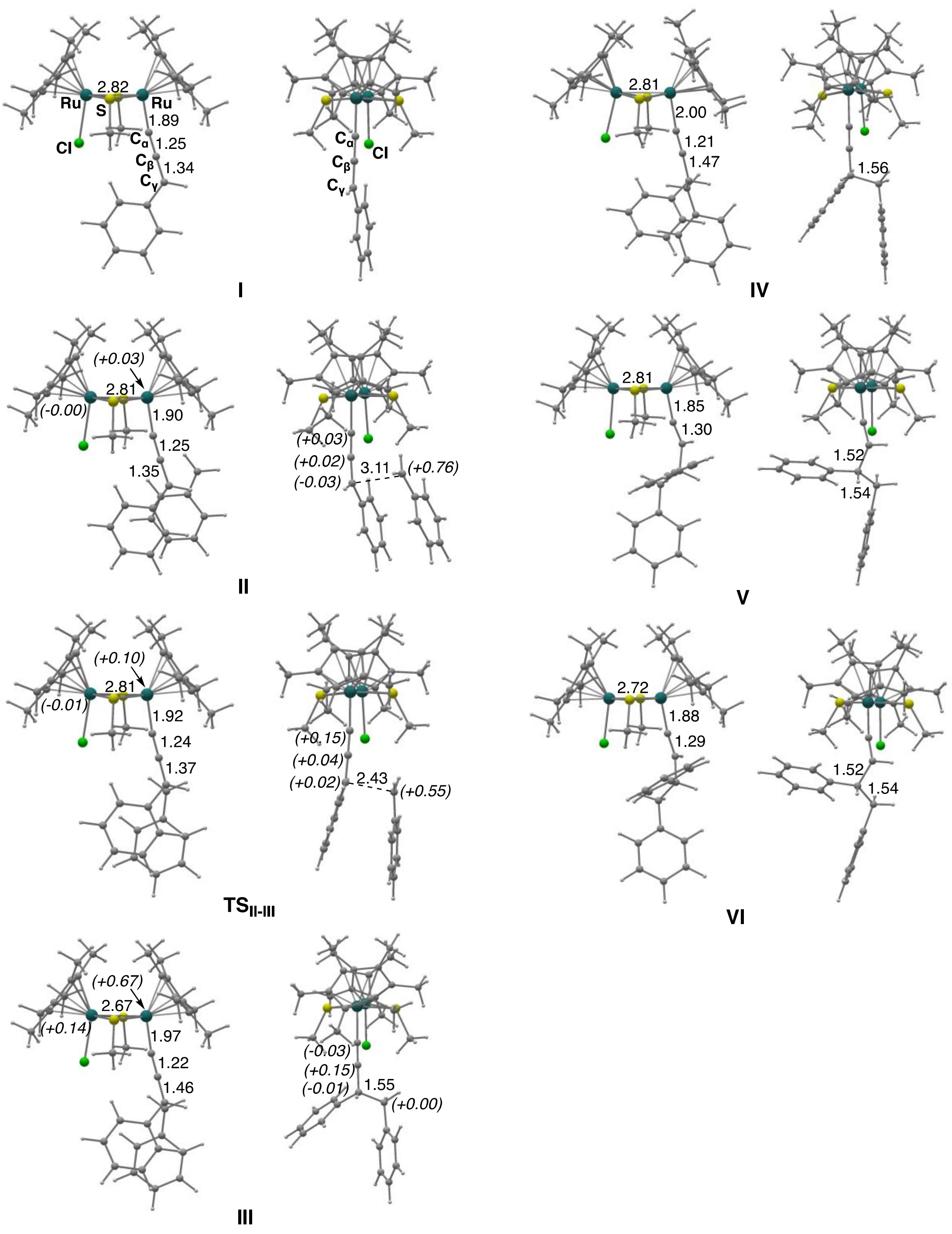

Supplementary Fig. 5: Structures optimized at ωB97X-D/(SDD, 6-311G**) level of theory. Bond lengths are given in Å.

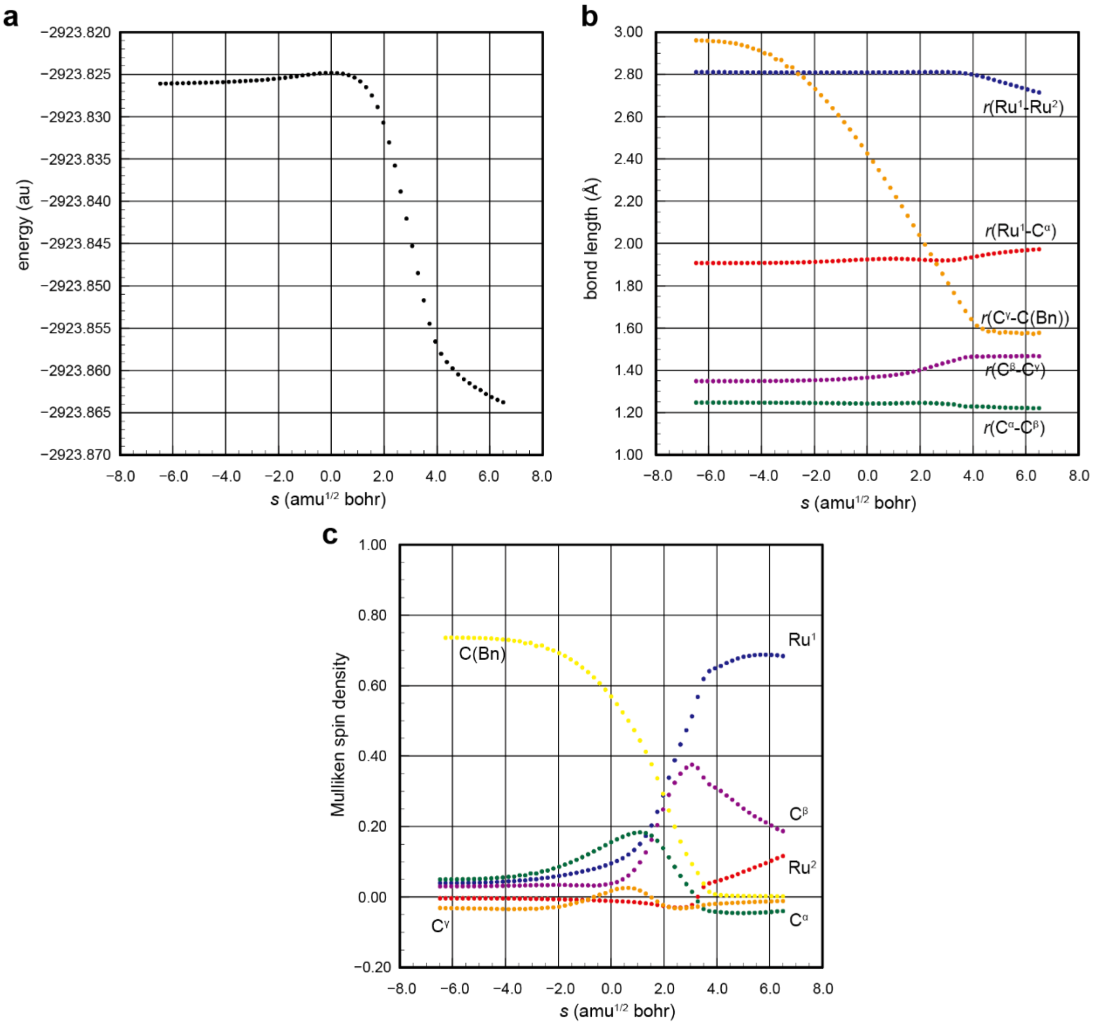

Supplementary Fig. 6: Profiles for $\mathbf{TS_{II\text{-}III}}$. **a** Potential energy profile along IRC for $\mathbf{TS_{II\text{-}III}}$. **b** Changes in bond lengths along the IRC for $\mathbf{TS_{II\text{-}III}}$. **c** Changes in Mulliken spin density along the IRC for $\mathbf{TS_{II\text{-}III}}$.

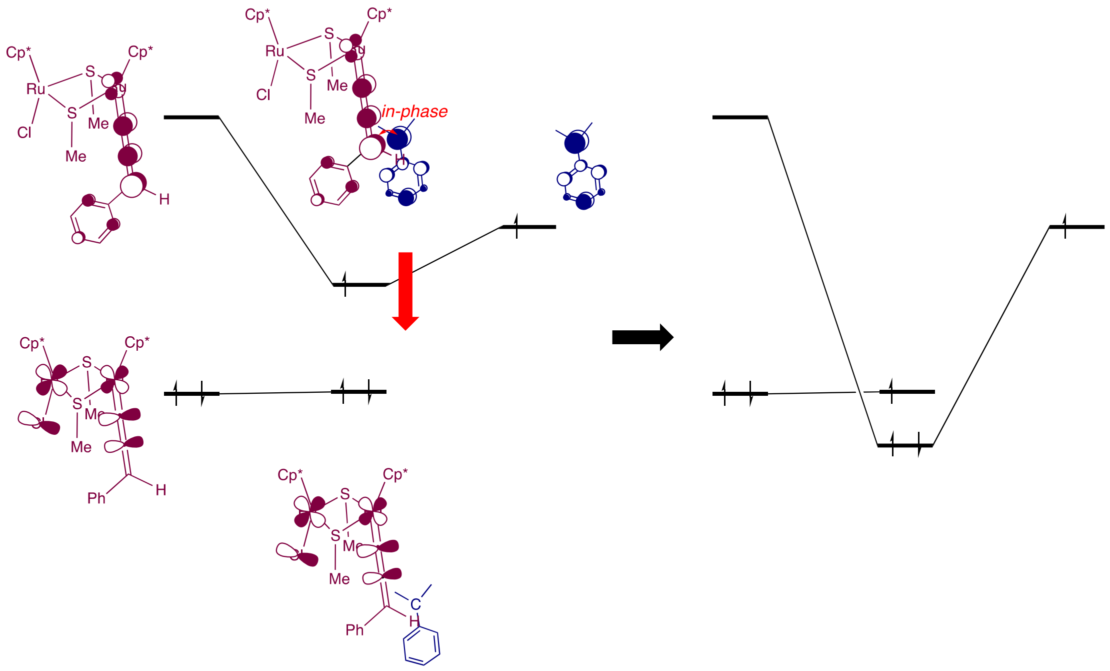

Supplementary Fig. 7: Change in SOMO along the reaction.

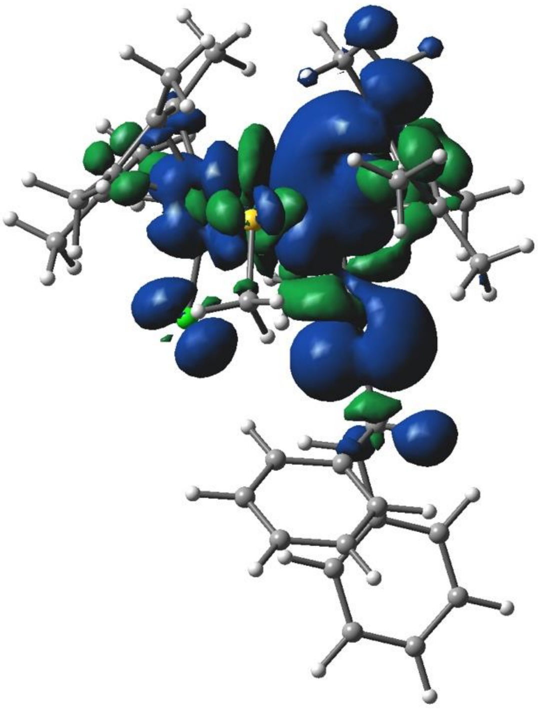

Supplementary Fig. 8: Spin density in **III**.

1.22. NMR charts

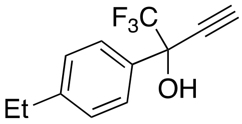

3c

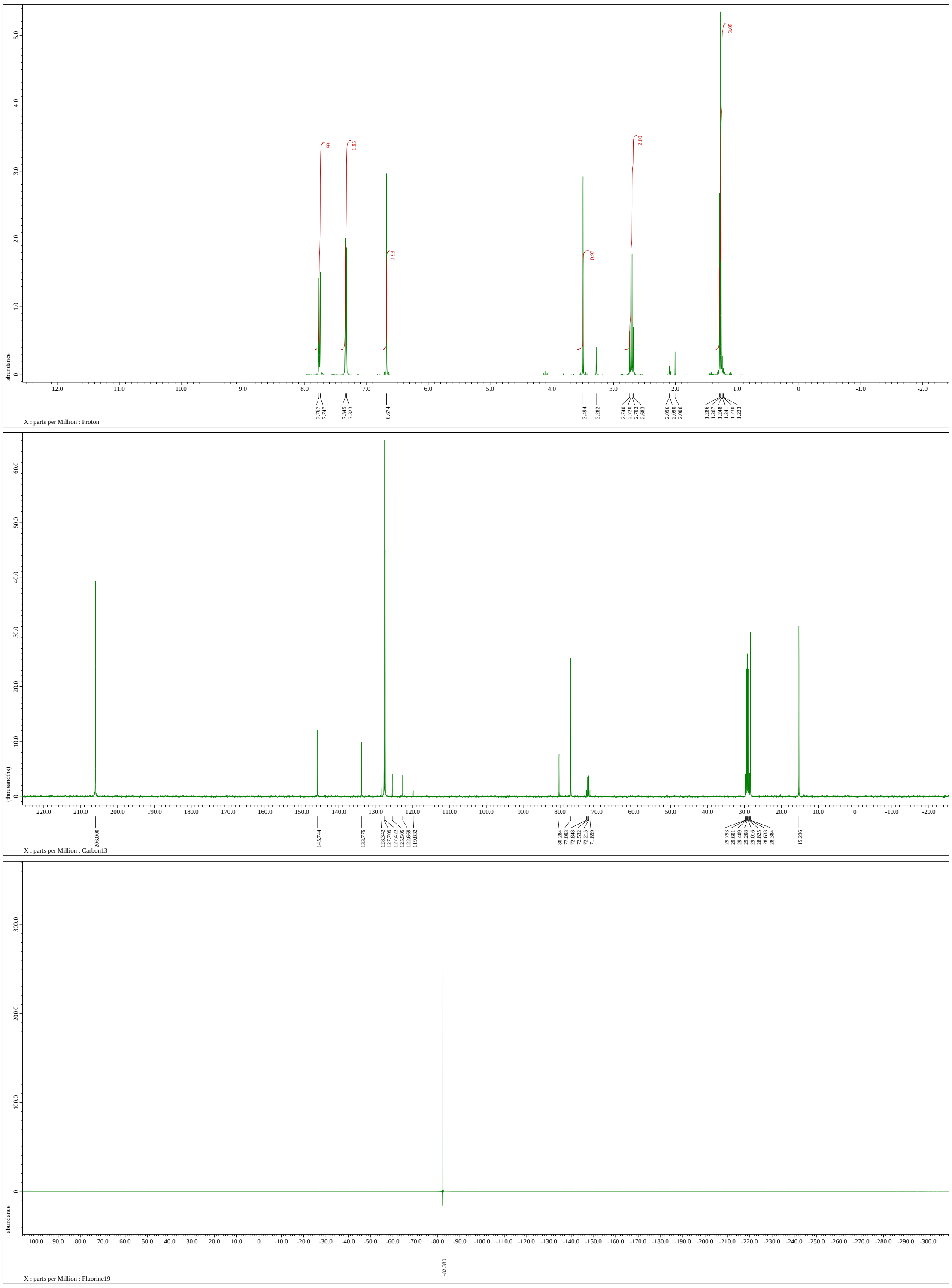

Supplementary Fig. 9: ^{1}H, ^{13}C, and ^{19}F NMR charts of **3c**.

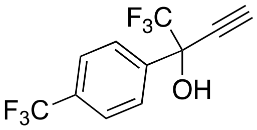

3h

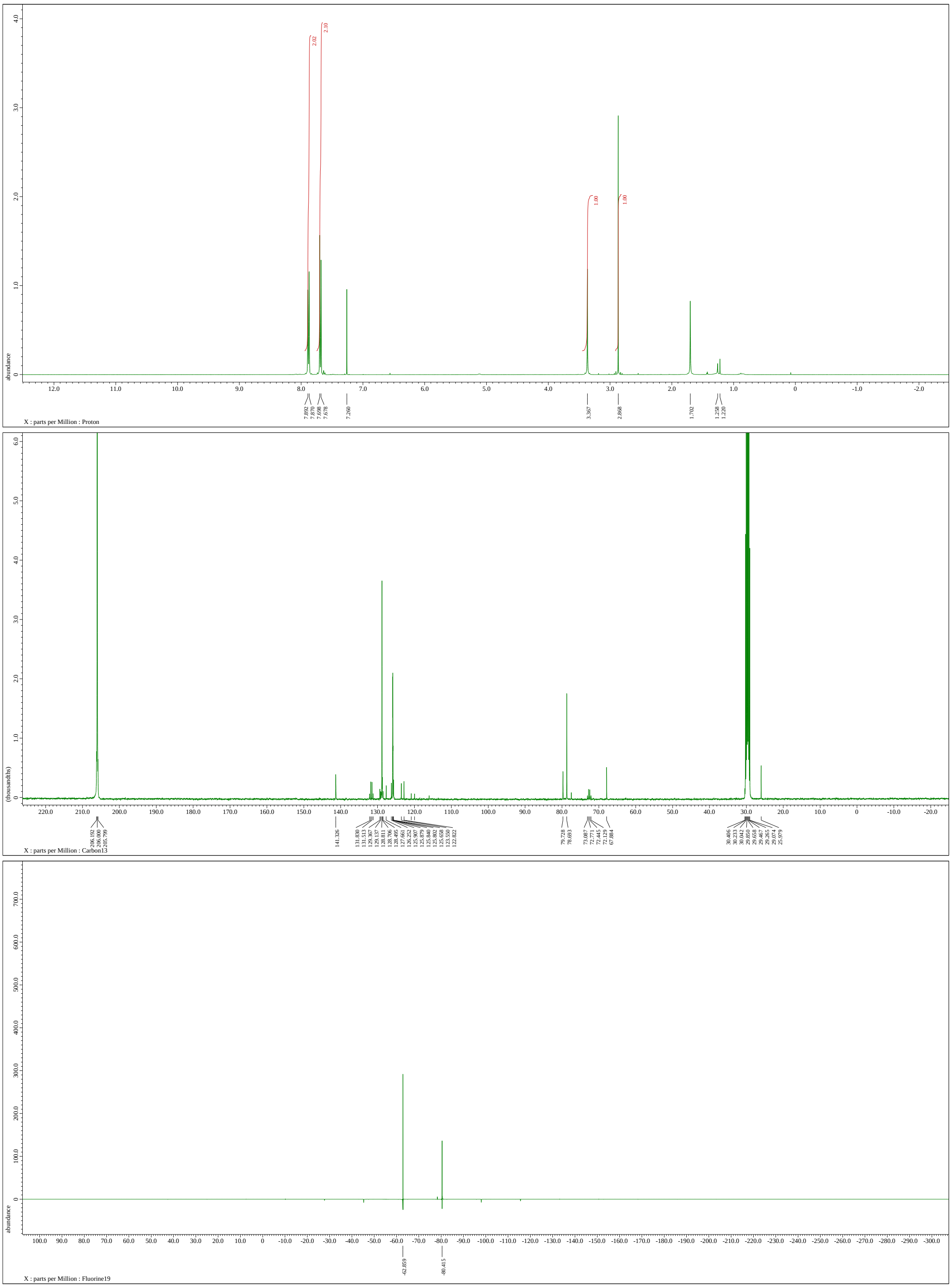

Supplementary Fig. 10: ^{1}H, ^{13}C, and ^{19}F NMR charts of **3h**.

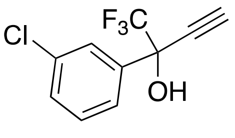

3k

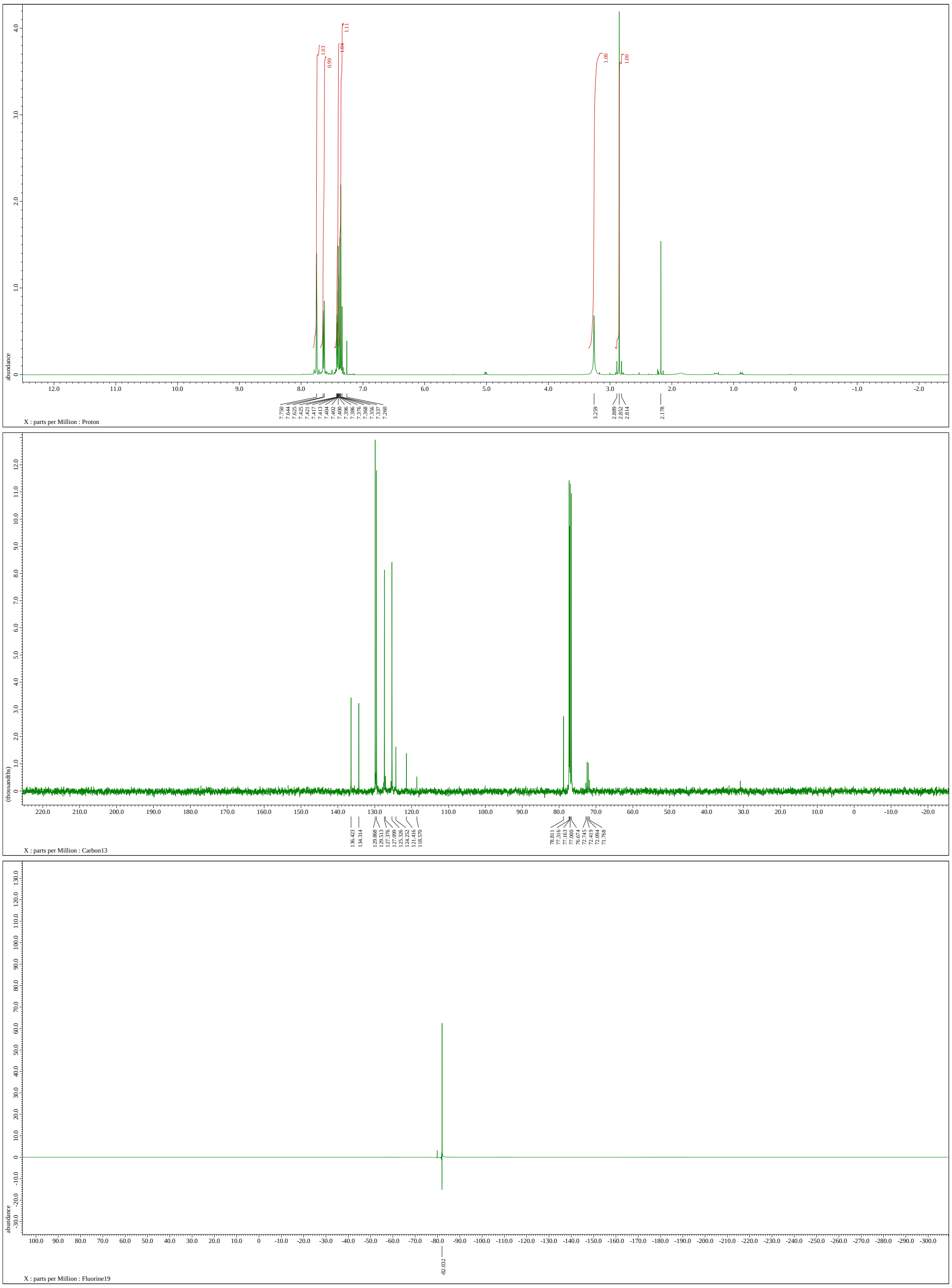

Supplementary Fig. 11: ^{1}H, ^{13}C, and ^{19}F NMR charts of **3k**.

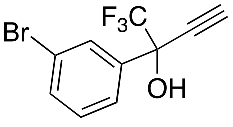

3l

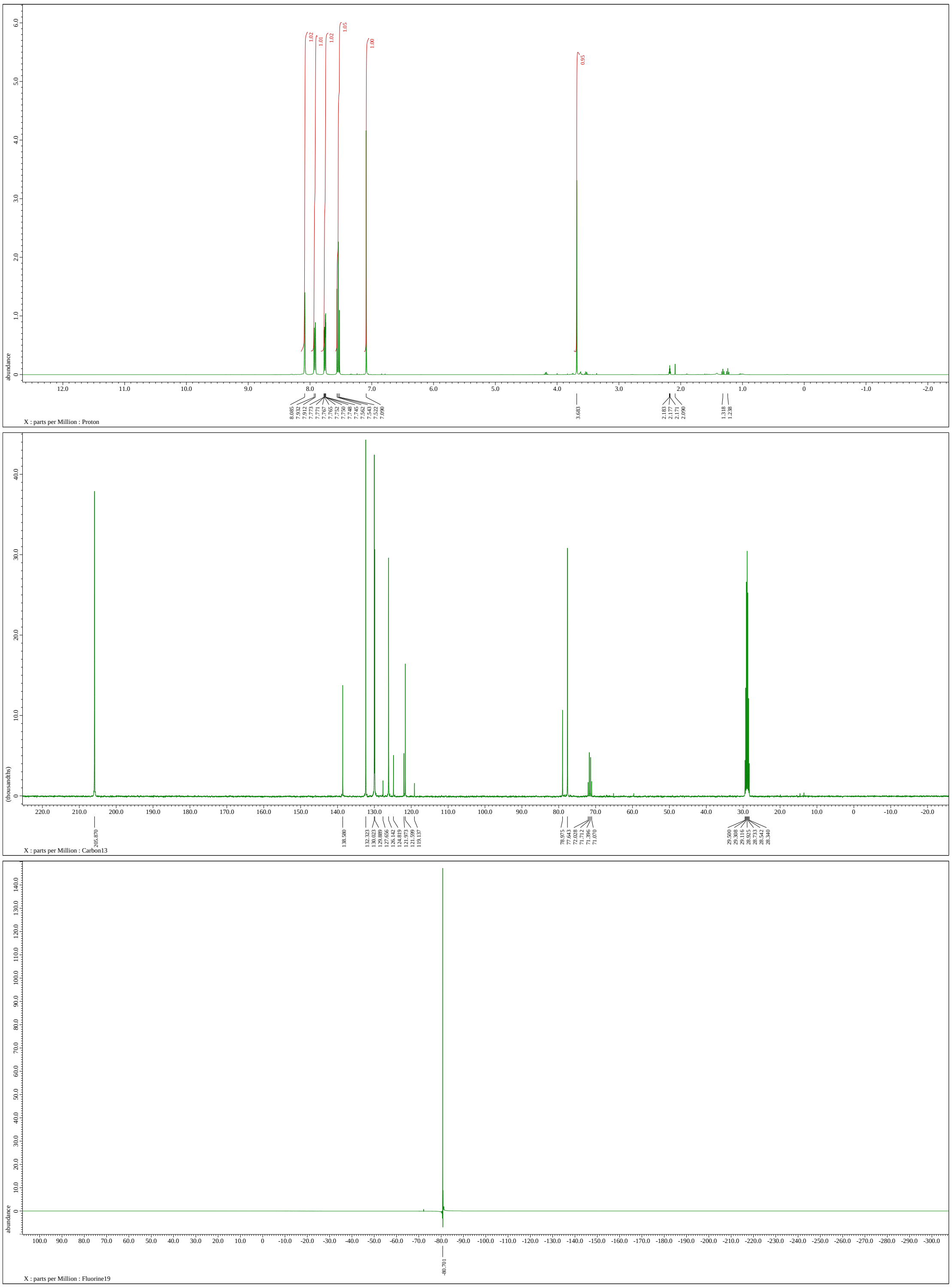

Supplementary Fig. 12: ^{1}H, ^{13}C, and ^{19}F NMR charts of **3l**.

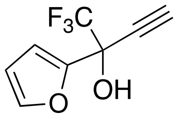

3m

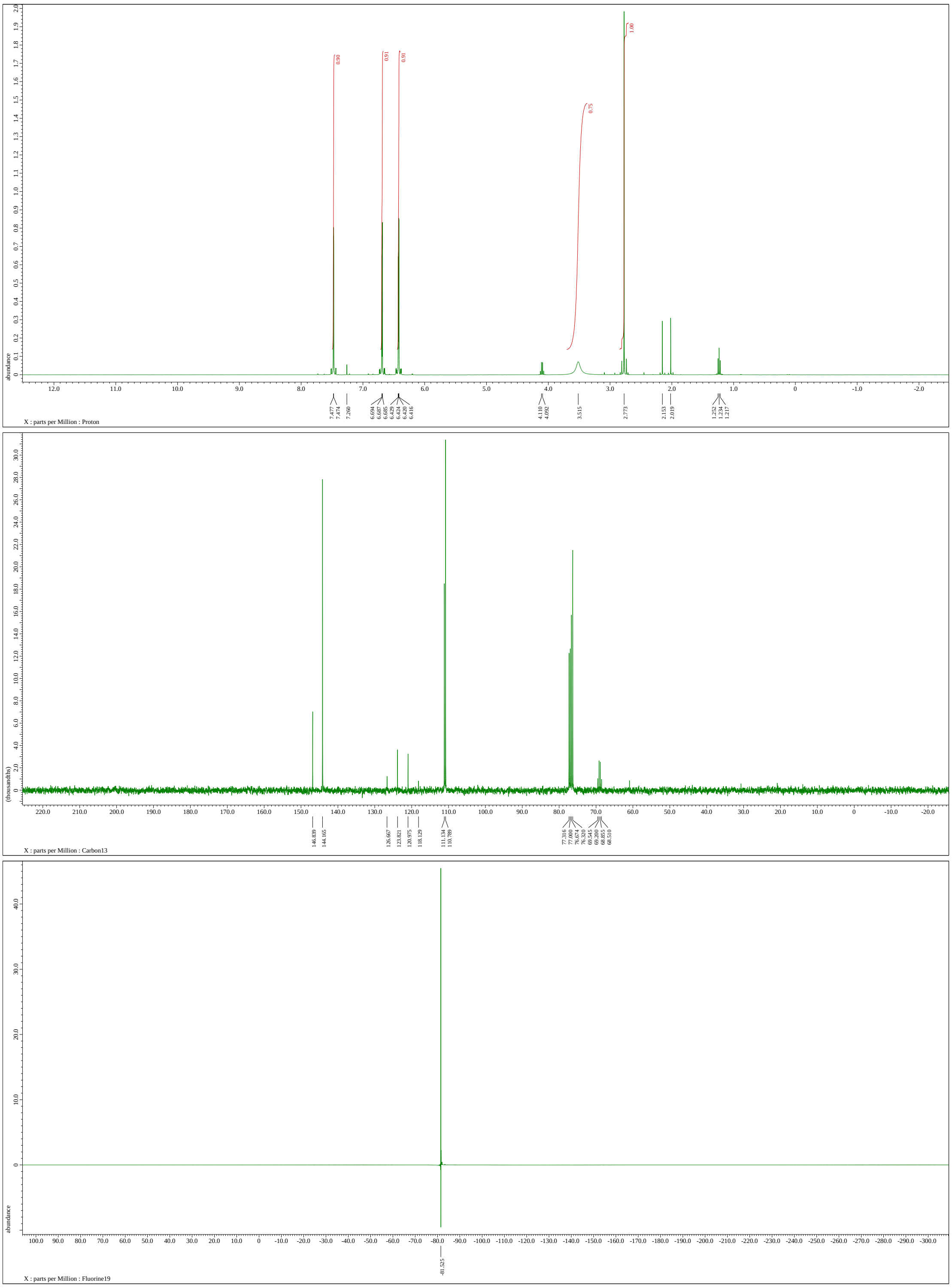

Supplementary Fig. 13: ^{1}H, ^{13}C, and ^{19}F NMR charts of **3m**.

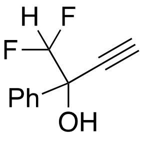

8

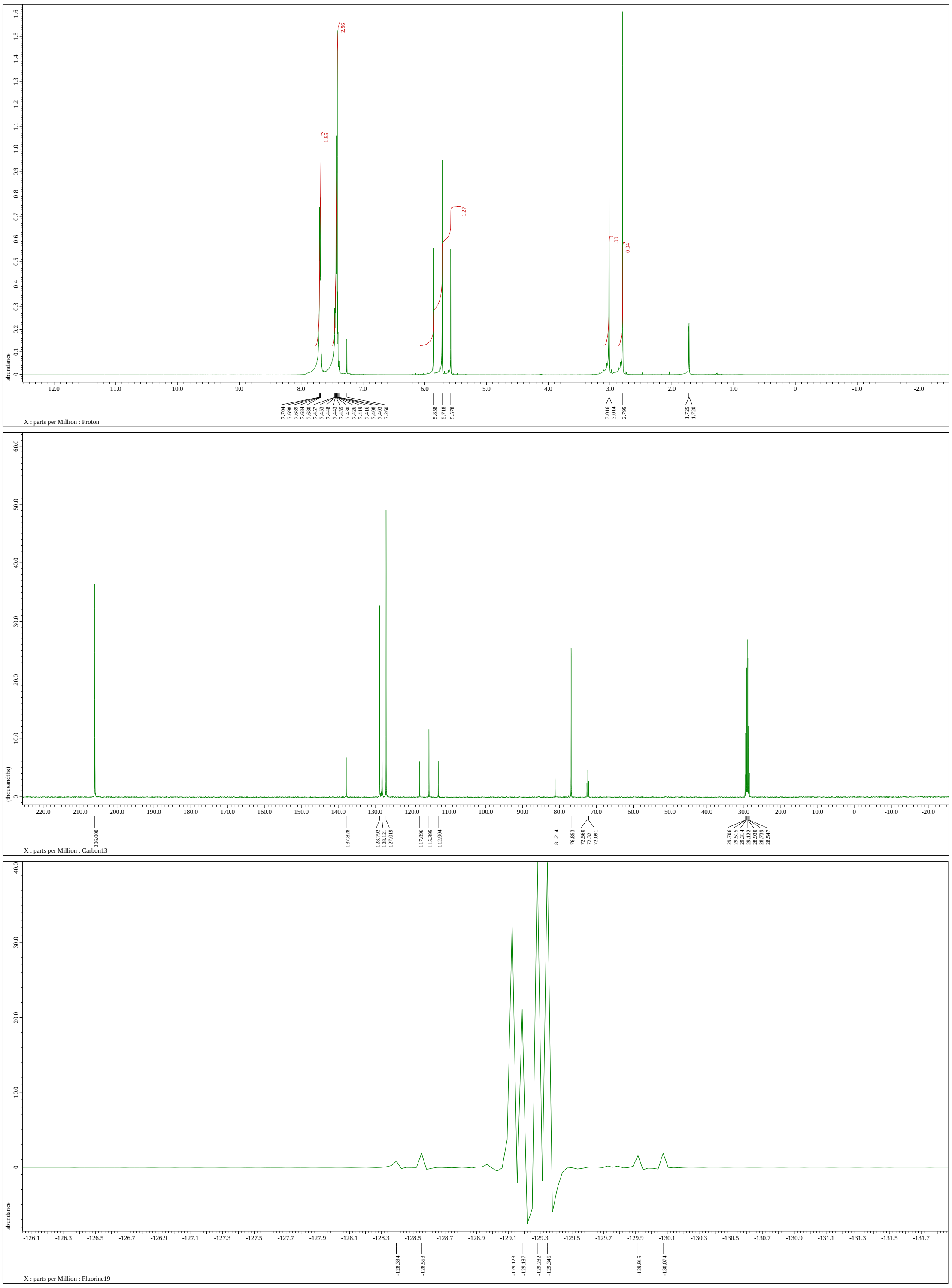

Supplementary Fig. 14: ^{1}H, ^{13}C, and ^{19}F NMR charts of **8**.

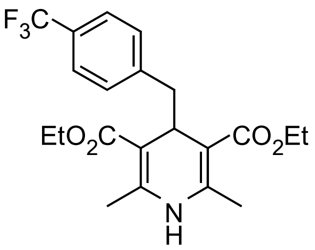

4h

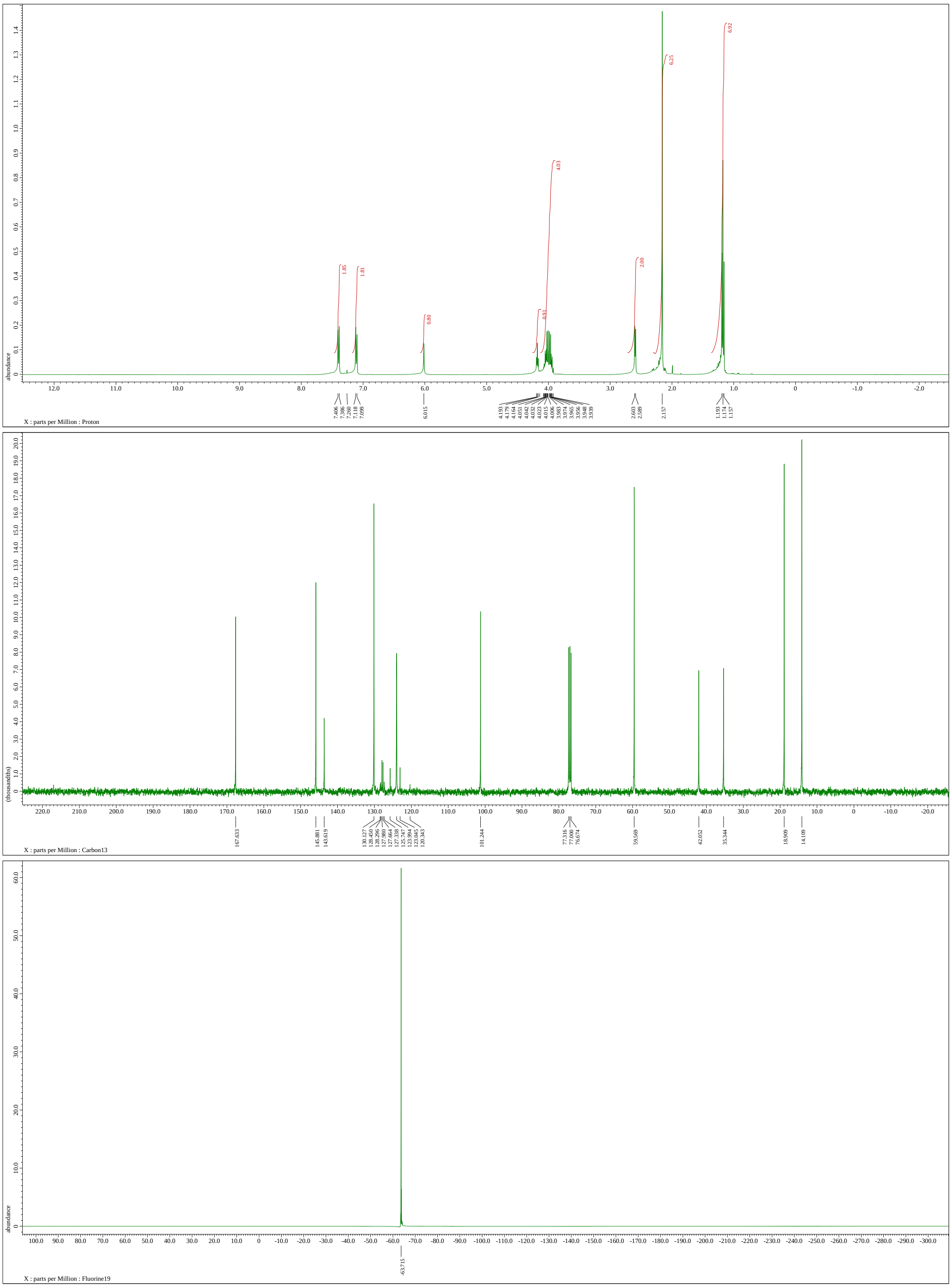

Supplementary Fig. 15: ^{1}H, ^{13}C, and ^{19}F NMR charts of **4h.**

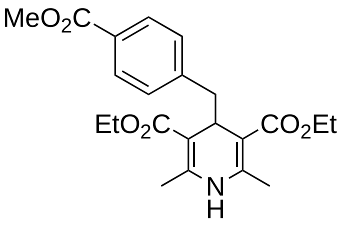

4i

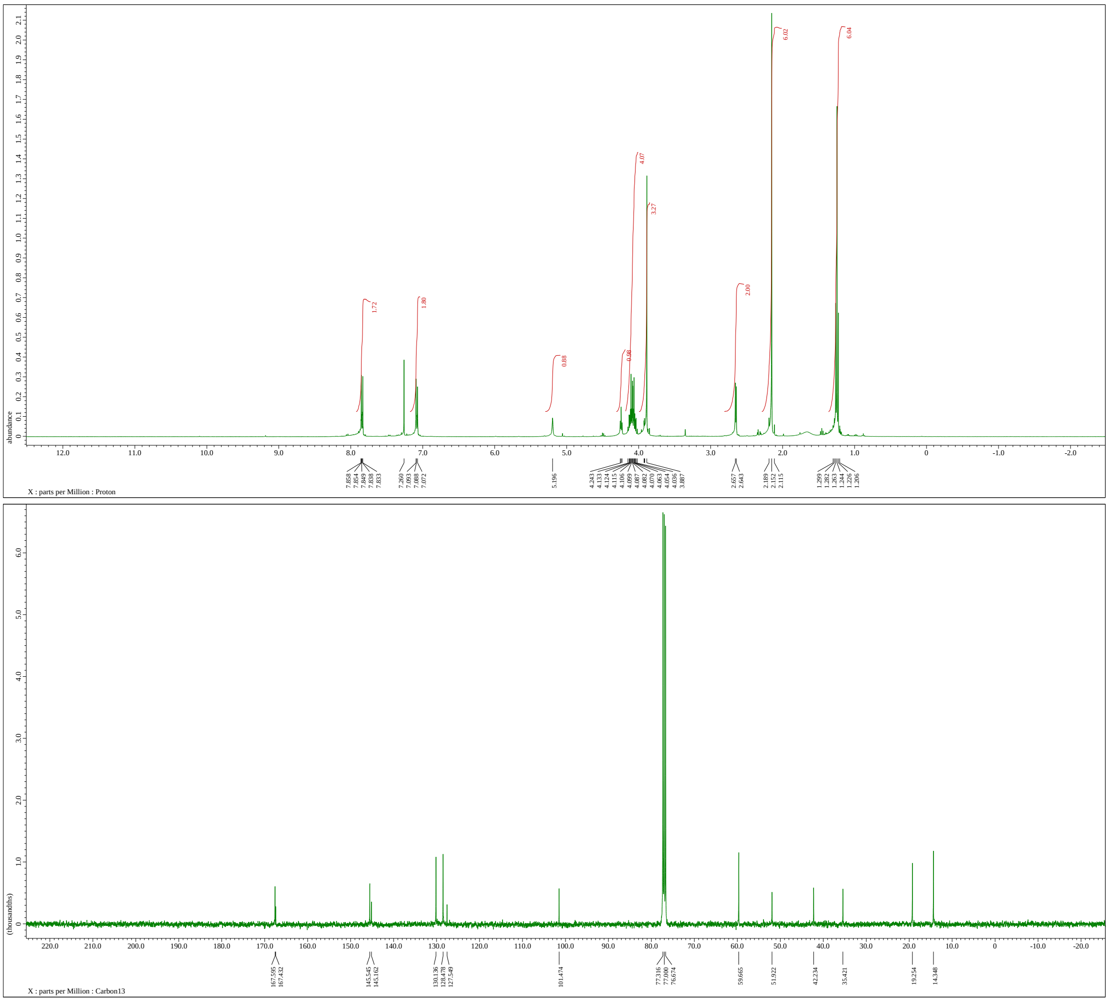

Supplementary Fig. 16: ^{1}H and ^{13}C NMR charts of **4i**.

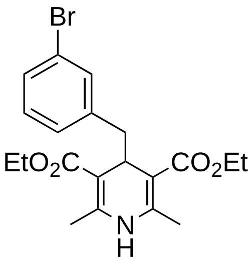

4o

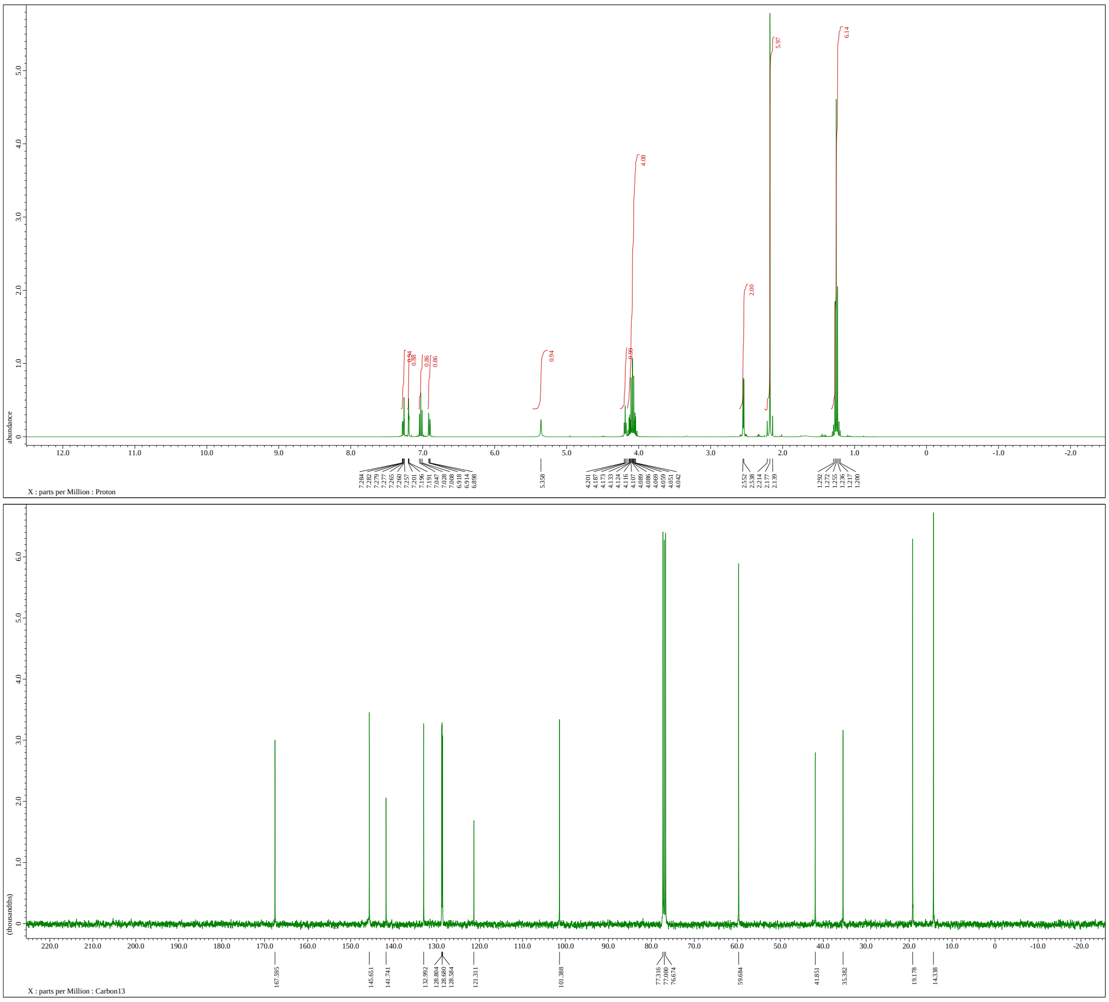

Supplementary Fig. 17: ^{1}H and ^{13}C NMR charts of **4o**.

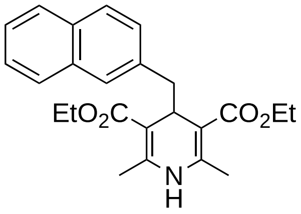

4s

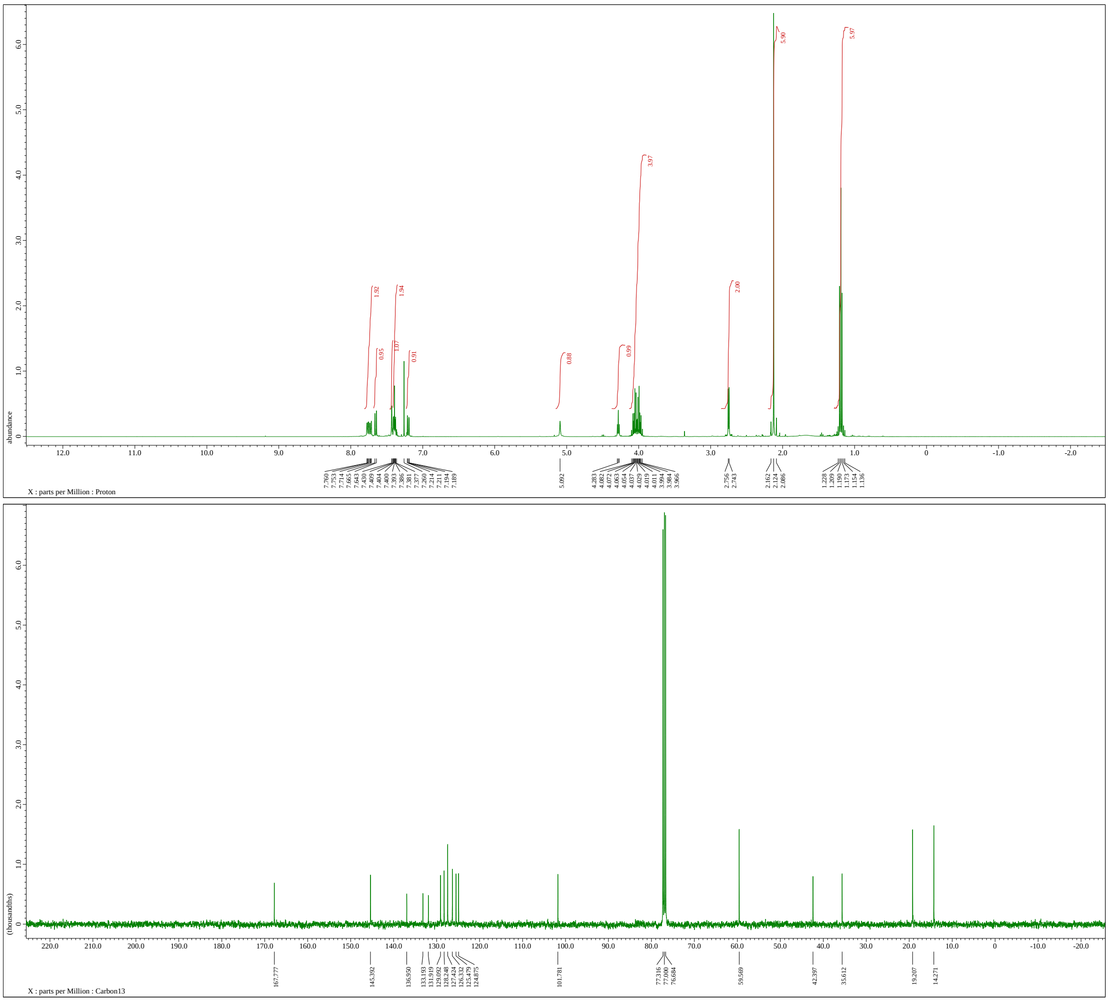

Supplementary Fig. 18: ^{1}H and ^{13}C NMR charts of **4s**.

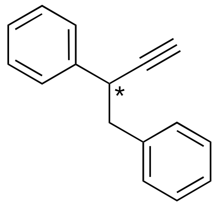

5

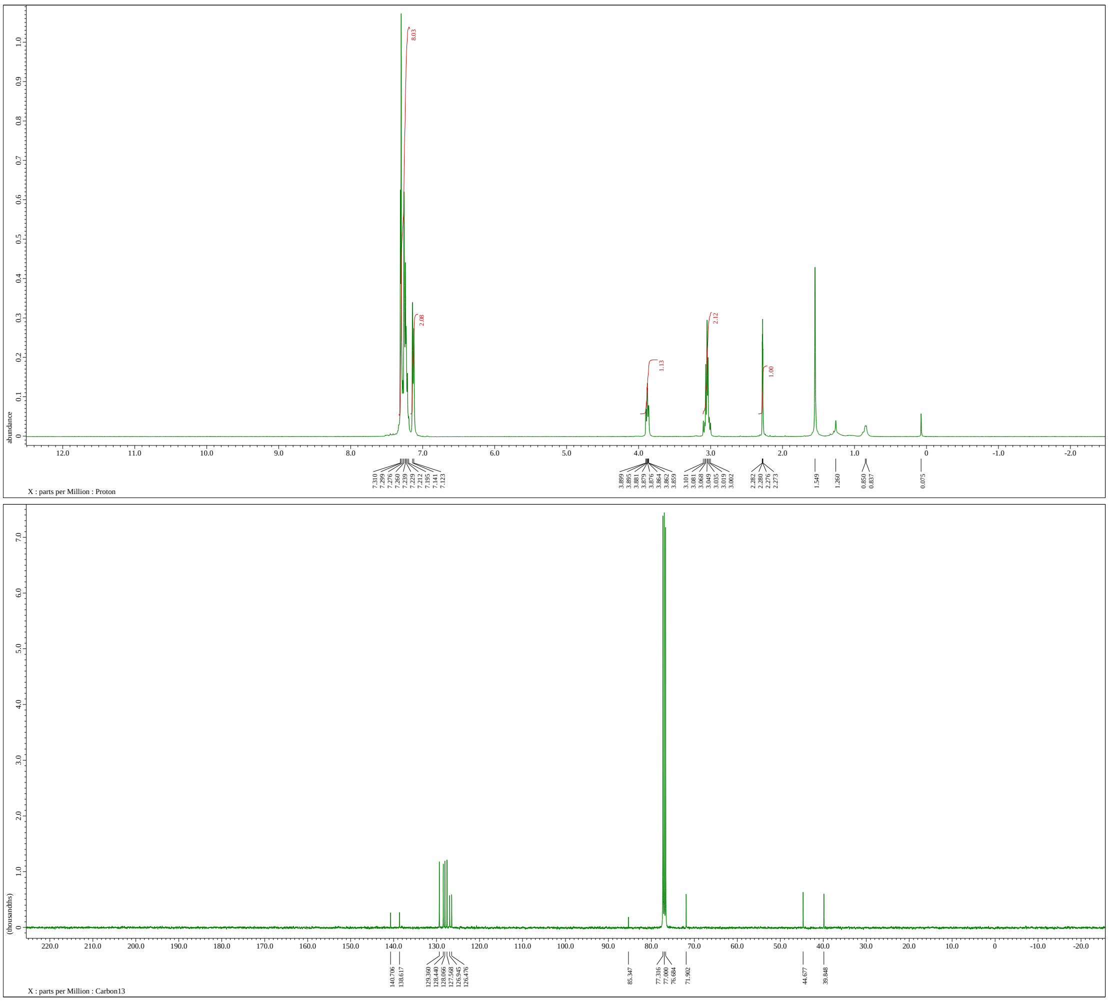

Supplementary Fig. 19: ^{1}H and ^{13}C NMR charts of **5**.

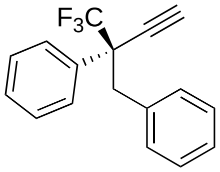

7aa

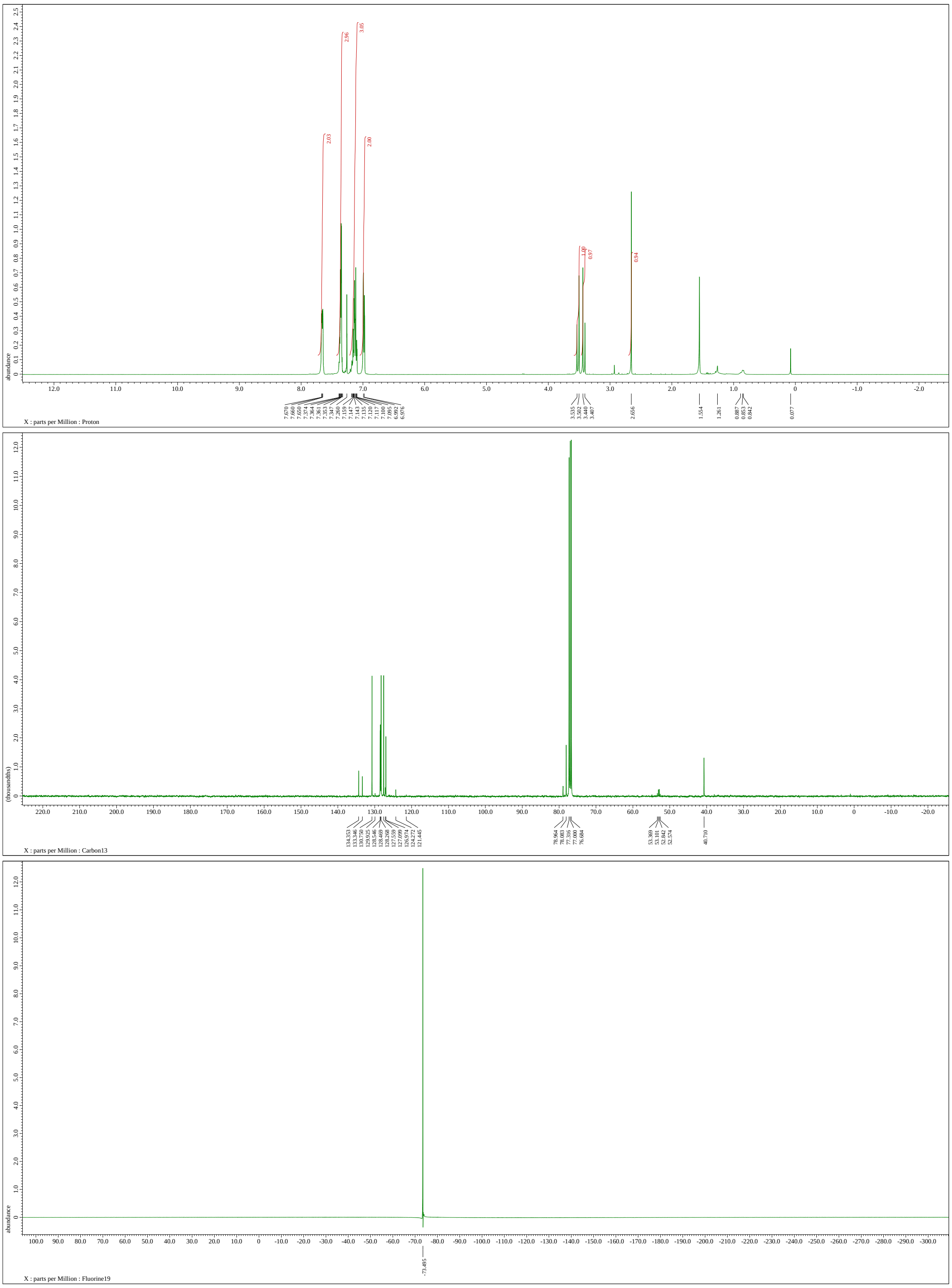

Supplementary Fig. 20: ^{1}H, ^{13}C, and ^{19}F NMR charts of **7aa**.

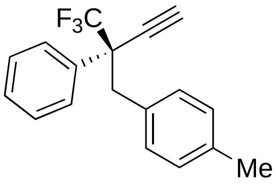

7ab

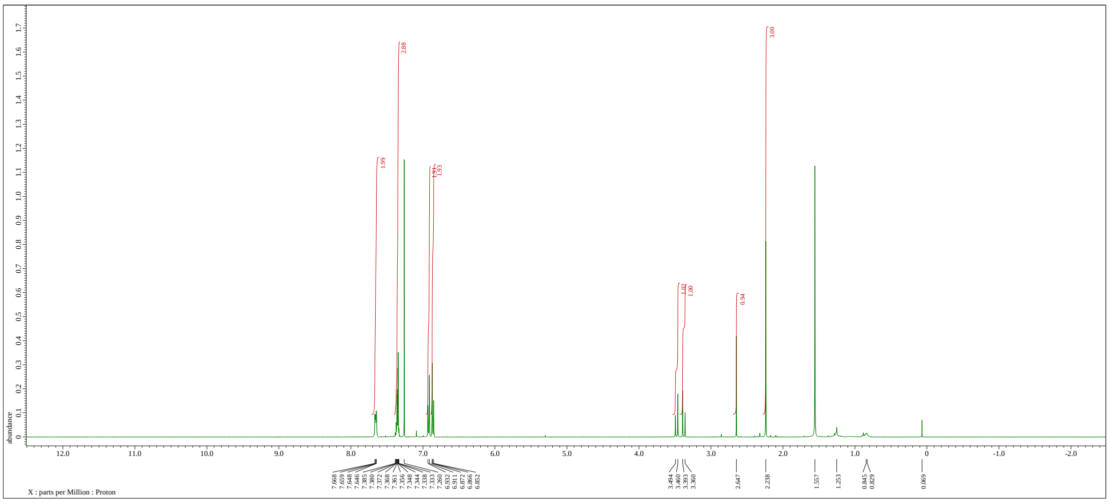

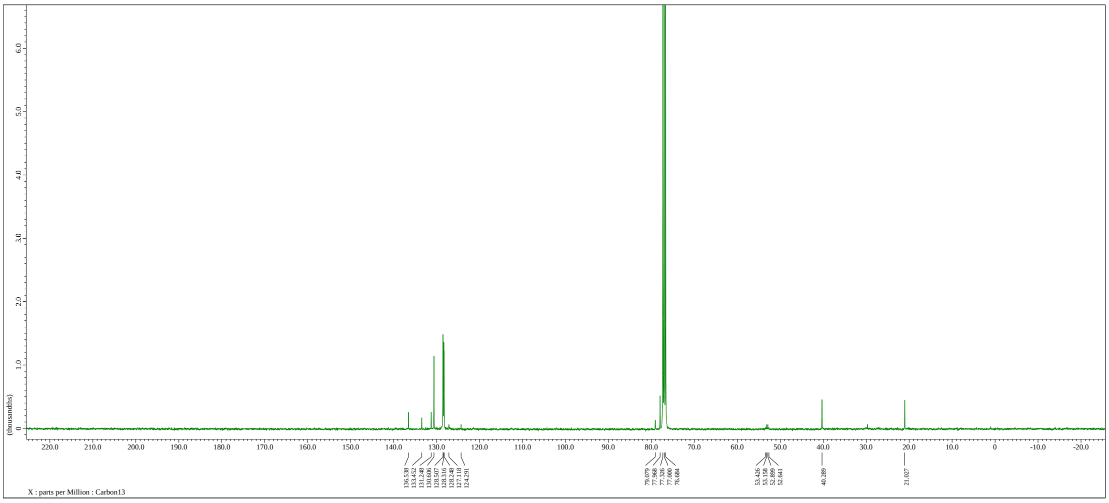

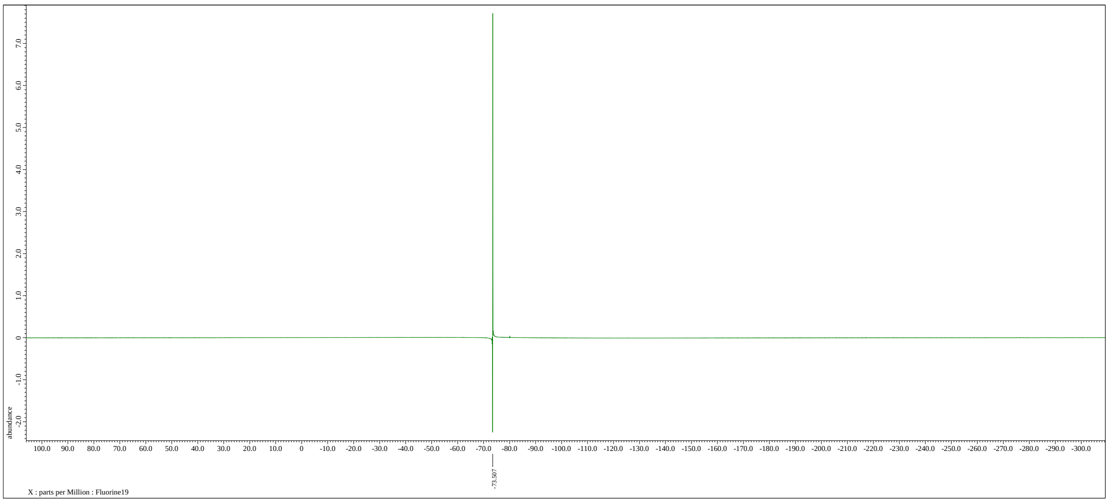

Supplementary Fig. 21: ^{1}H, ^{13}C, and ^{19}F NMR charts of **7ab**.

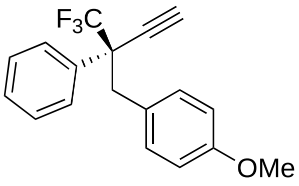

7ac

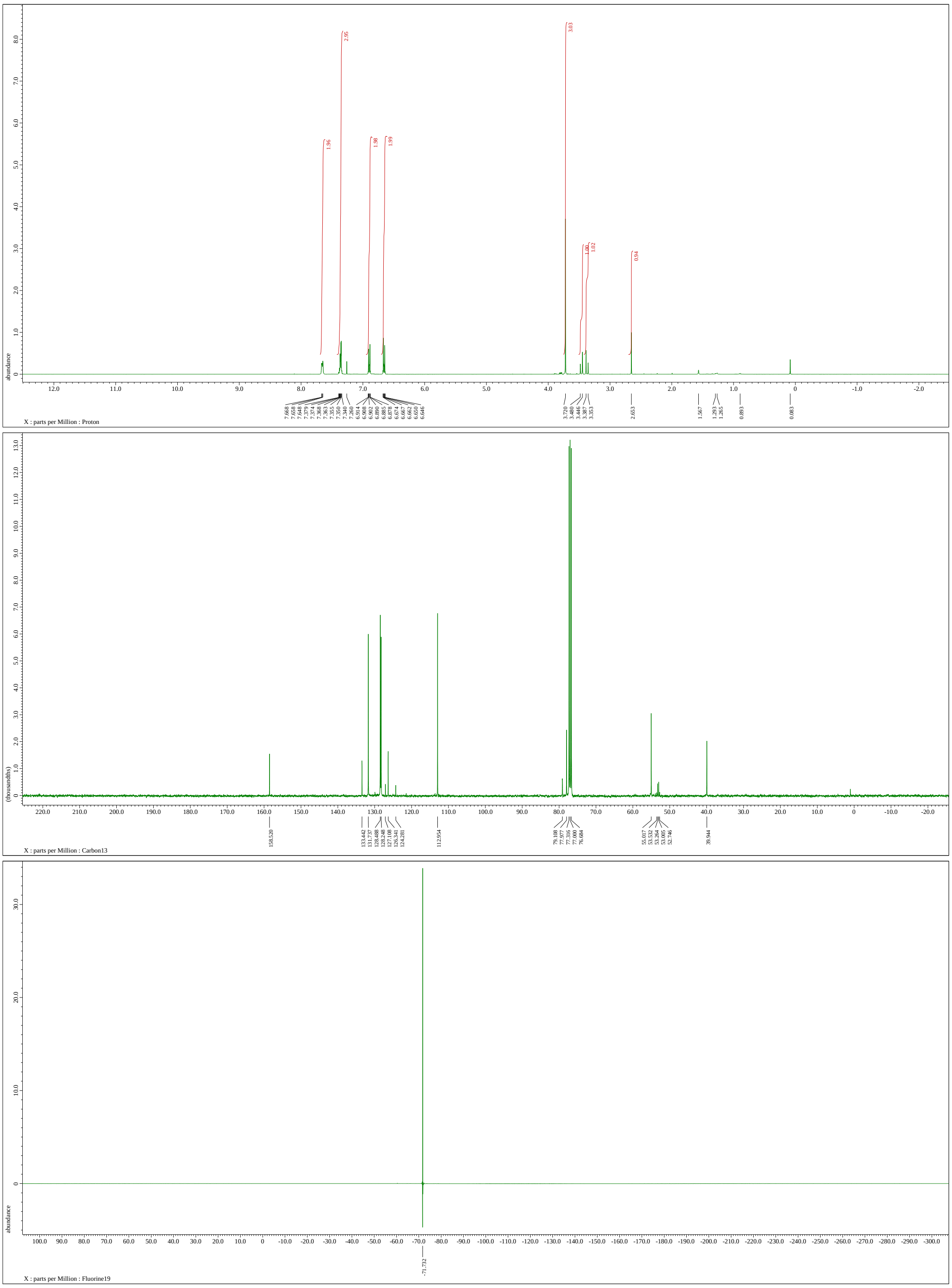

Supplementary Fig. 22: ^{1}H, ^{13}C, and ^{19}F NMR charts of **7ac**.

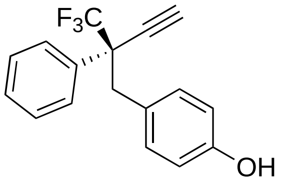

7ad

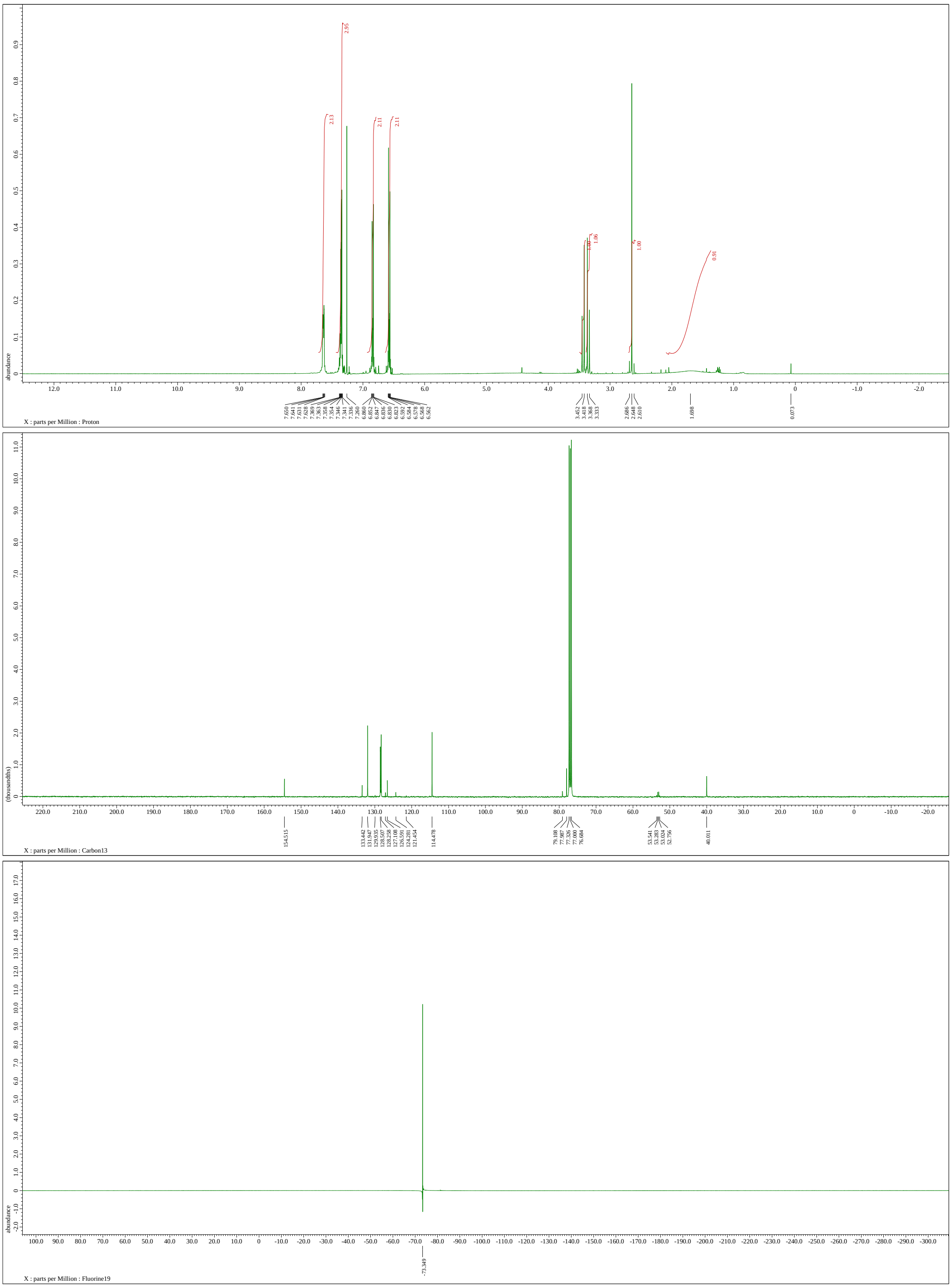

Supplementary Fig. 23: ^{1}H, ^{13}C, and ^{19}F NMR charts of **7ad**.

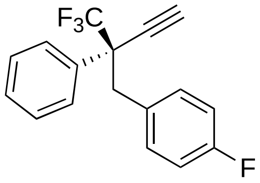

7ae

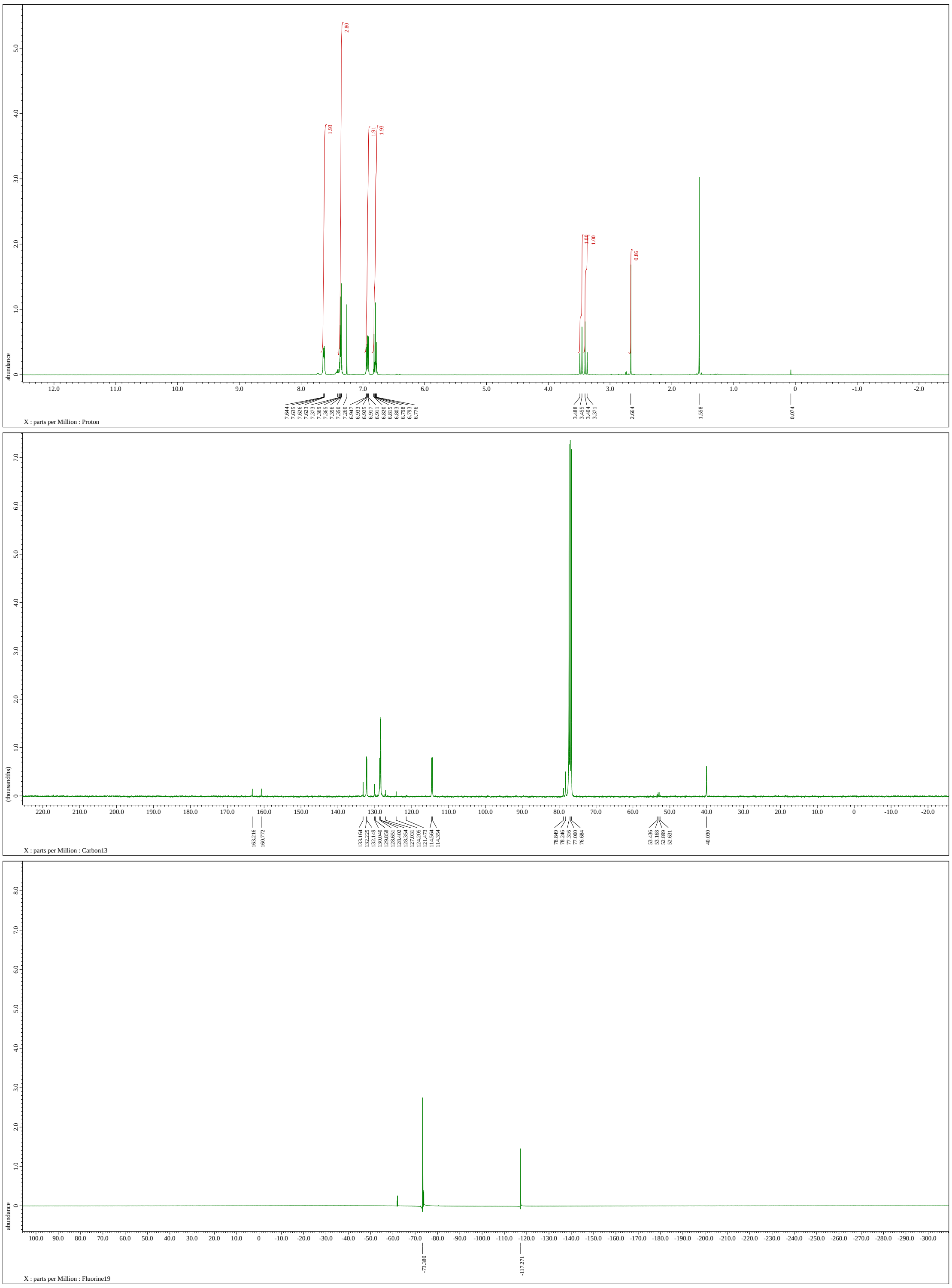

Supplementary Fig. 24: ^{1}H, ^{13}C, and ^{19}F NMR charts of **7ae**.

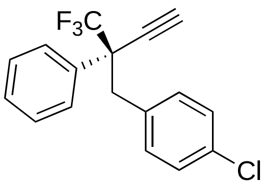

7af

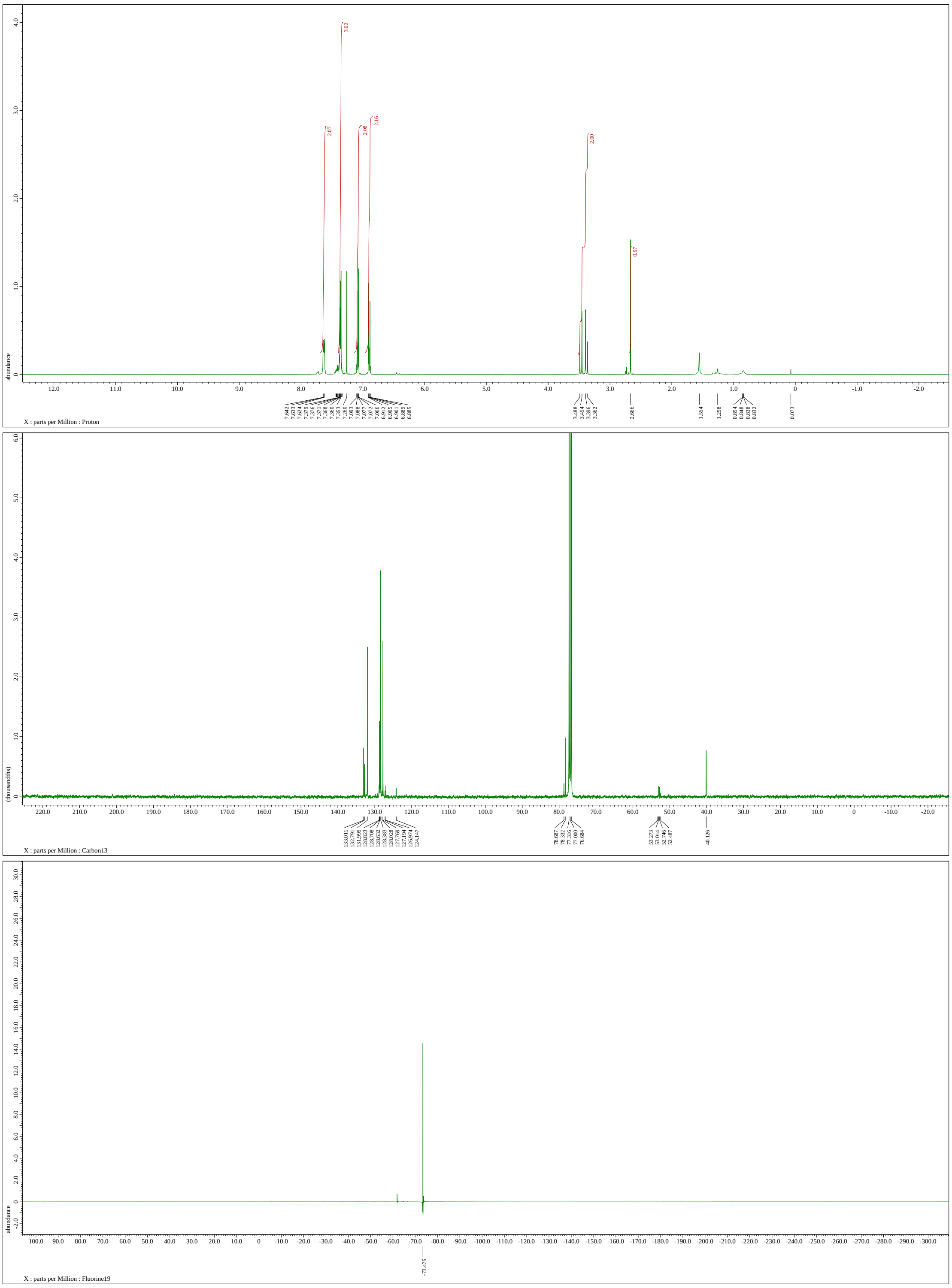

Supplementary Fig. 25: ^{1}H, ^{13}C, and ^{19}F NMR charts of **7af**.

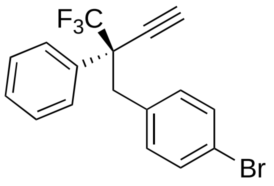

7ag

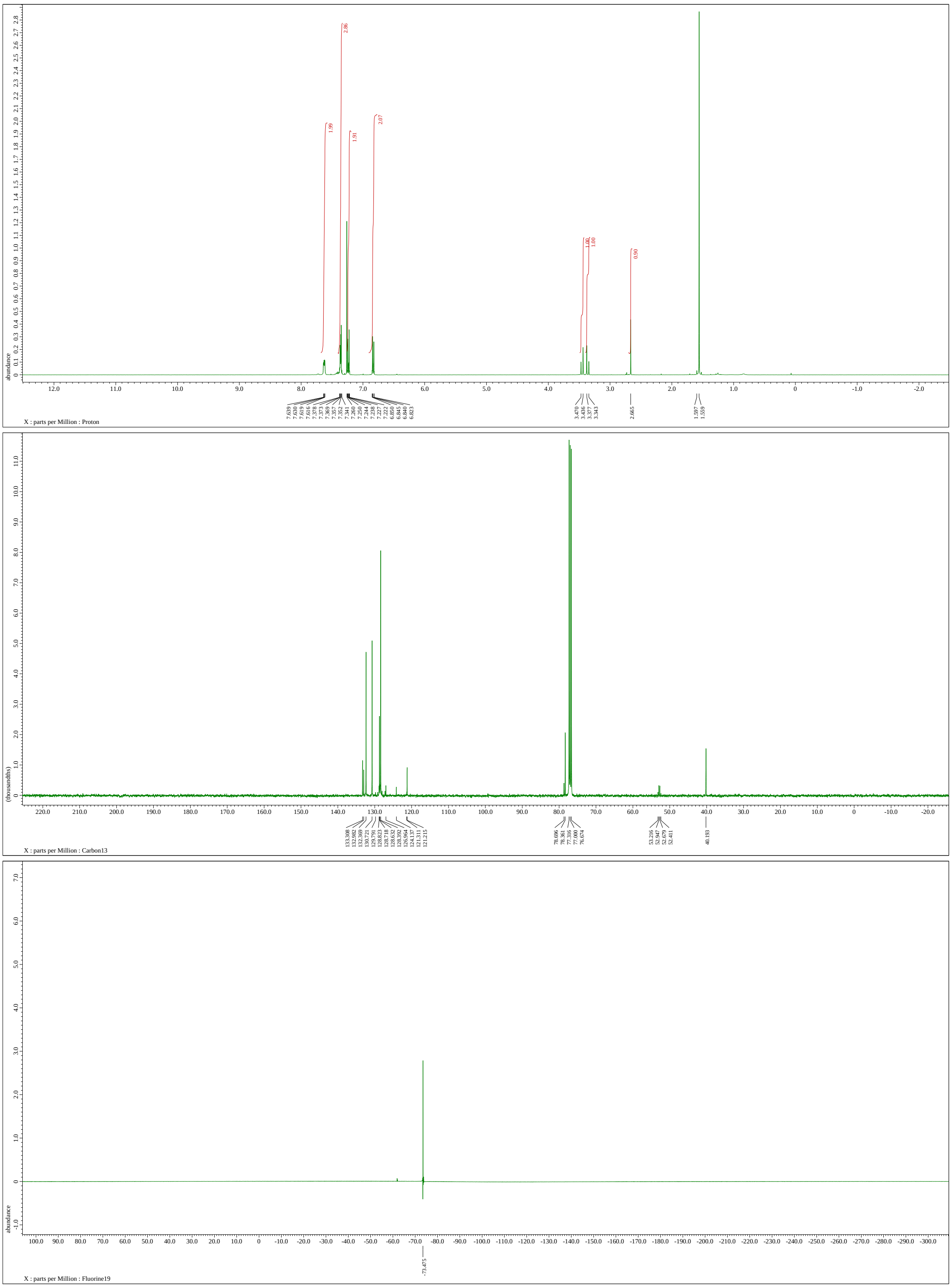

Supplementary Fig. 26: ^{1}H, ^{13}C, and ^{19}F NMR charts of **7ag**.

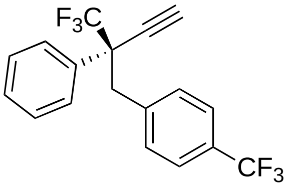

7ah

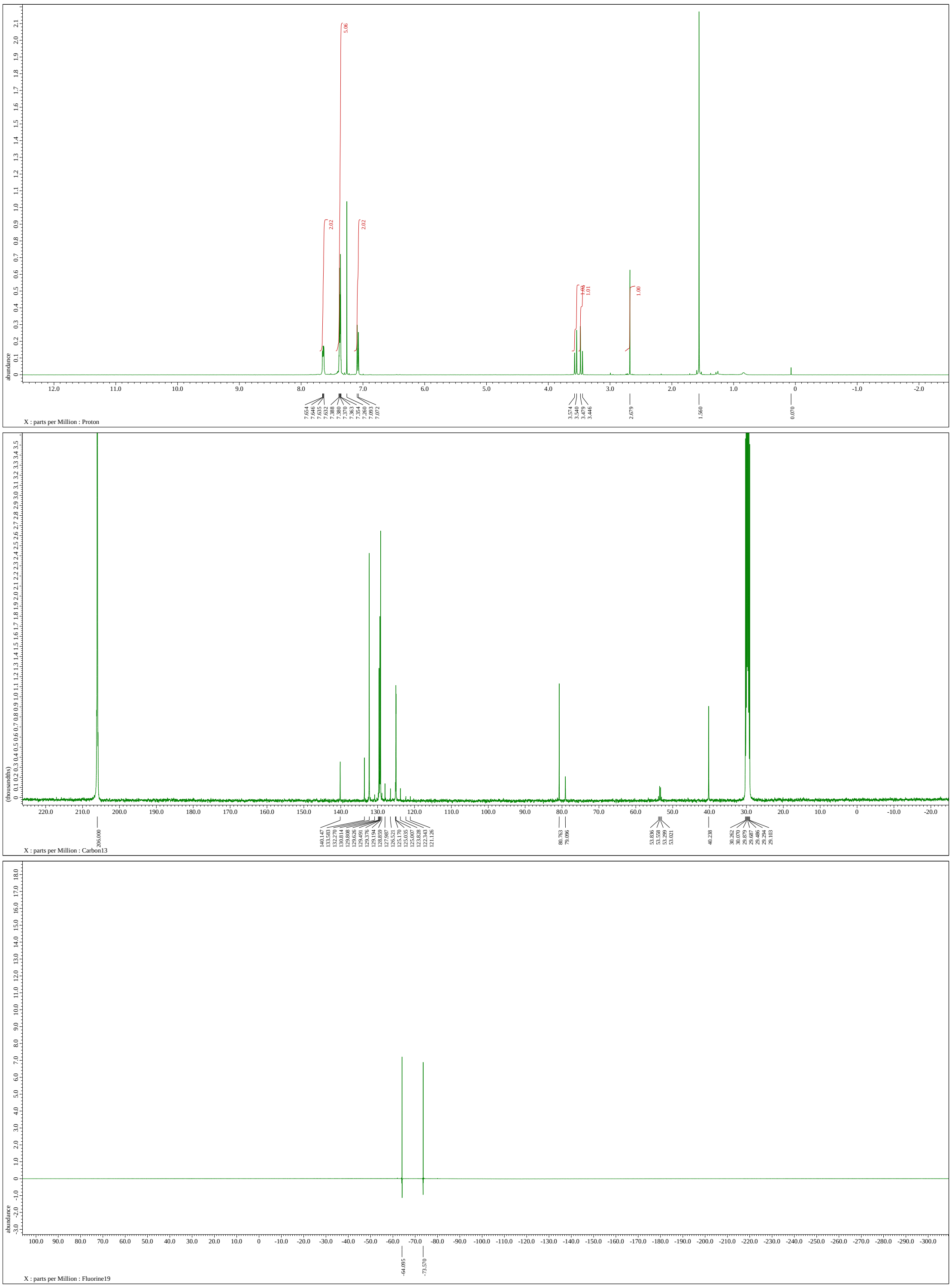

Supplementary Fig. 27: ^{1}H, ^{13}C, and ^{19}F NMR charts of **7ah**.

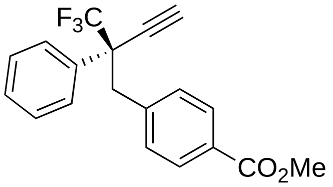

7ai

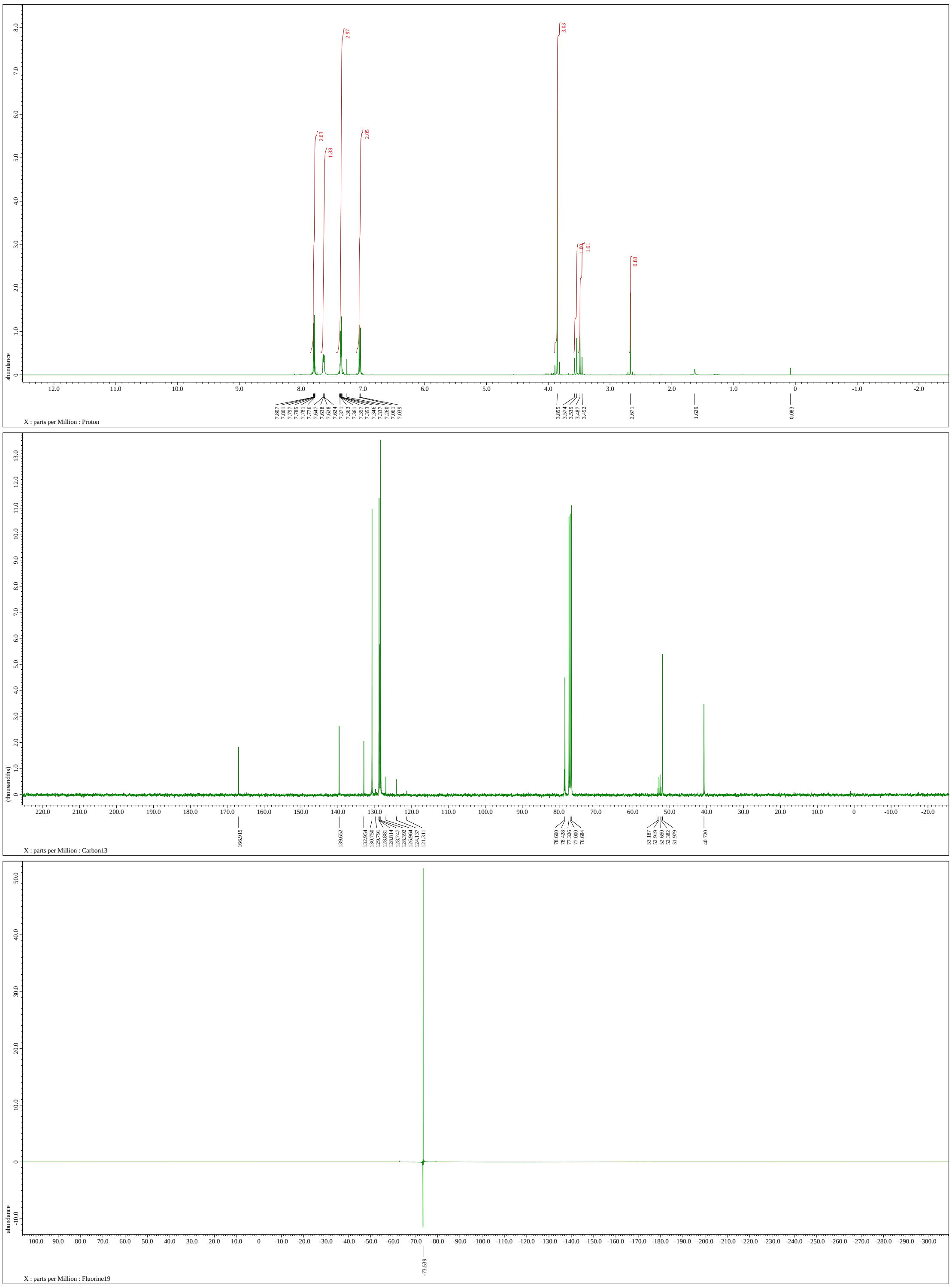

Supplementary Fig. 28: ^{1}H, ^{13}C, and ^{19}F NMR charts of **7ai**.

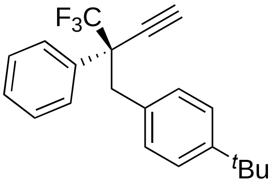

7aj

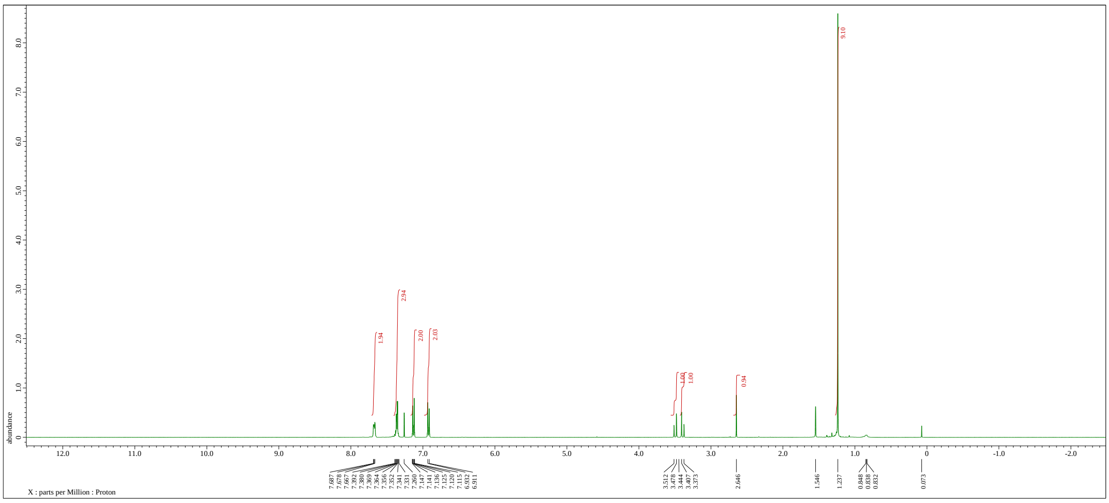

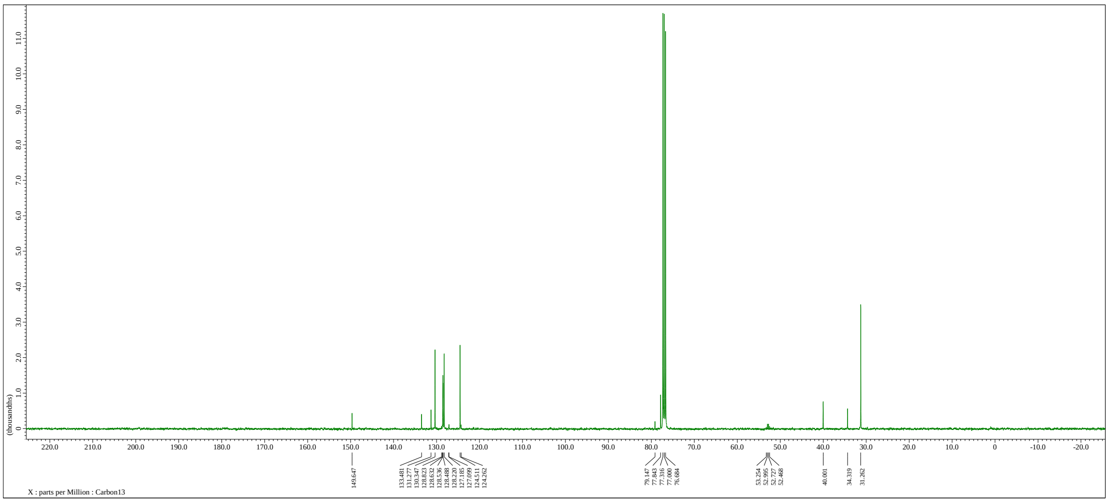

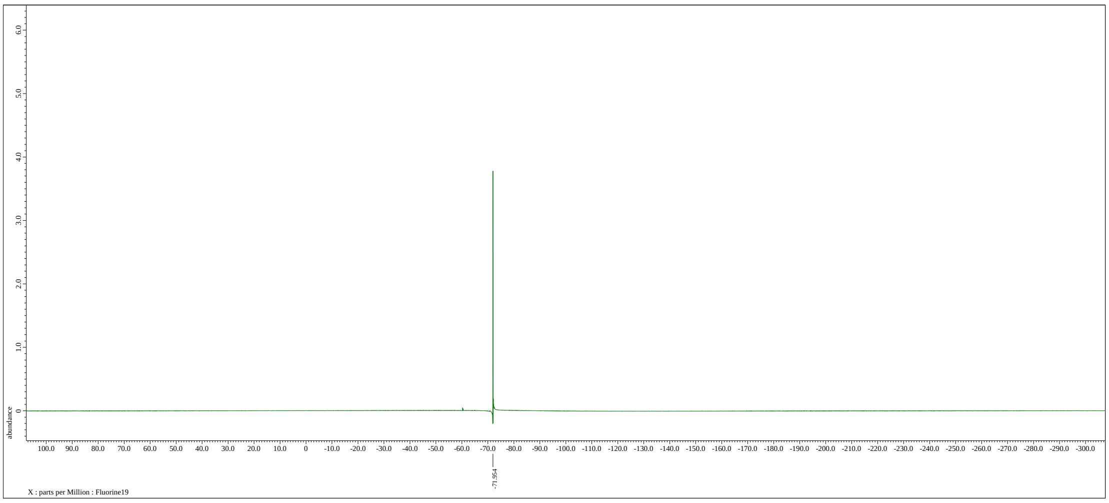

Supplementary Fig. 29: ^{1}H, ^{13}C, and ^{19}F NMR charts of **7aj**.

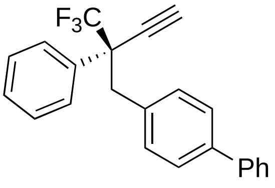

7ak

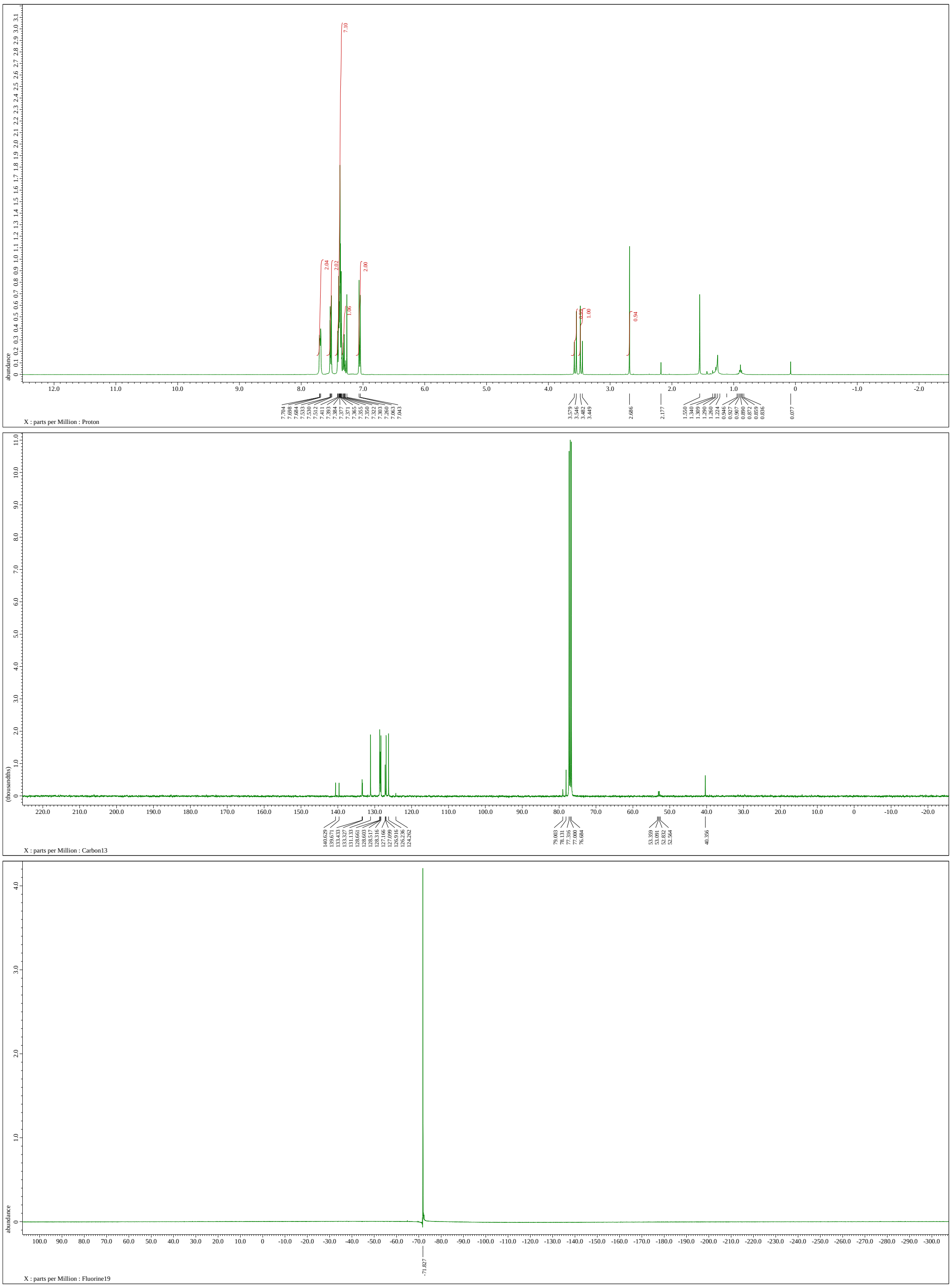

Supplementary Fig. 30: ^{1}H, ^{13}C, and ^{19}F NMR charts of **7ak**.

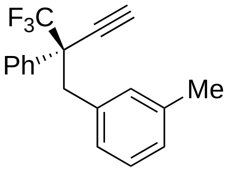

7al

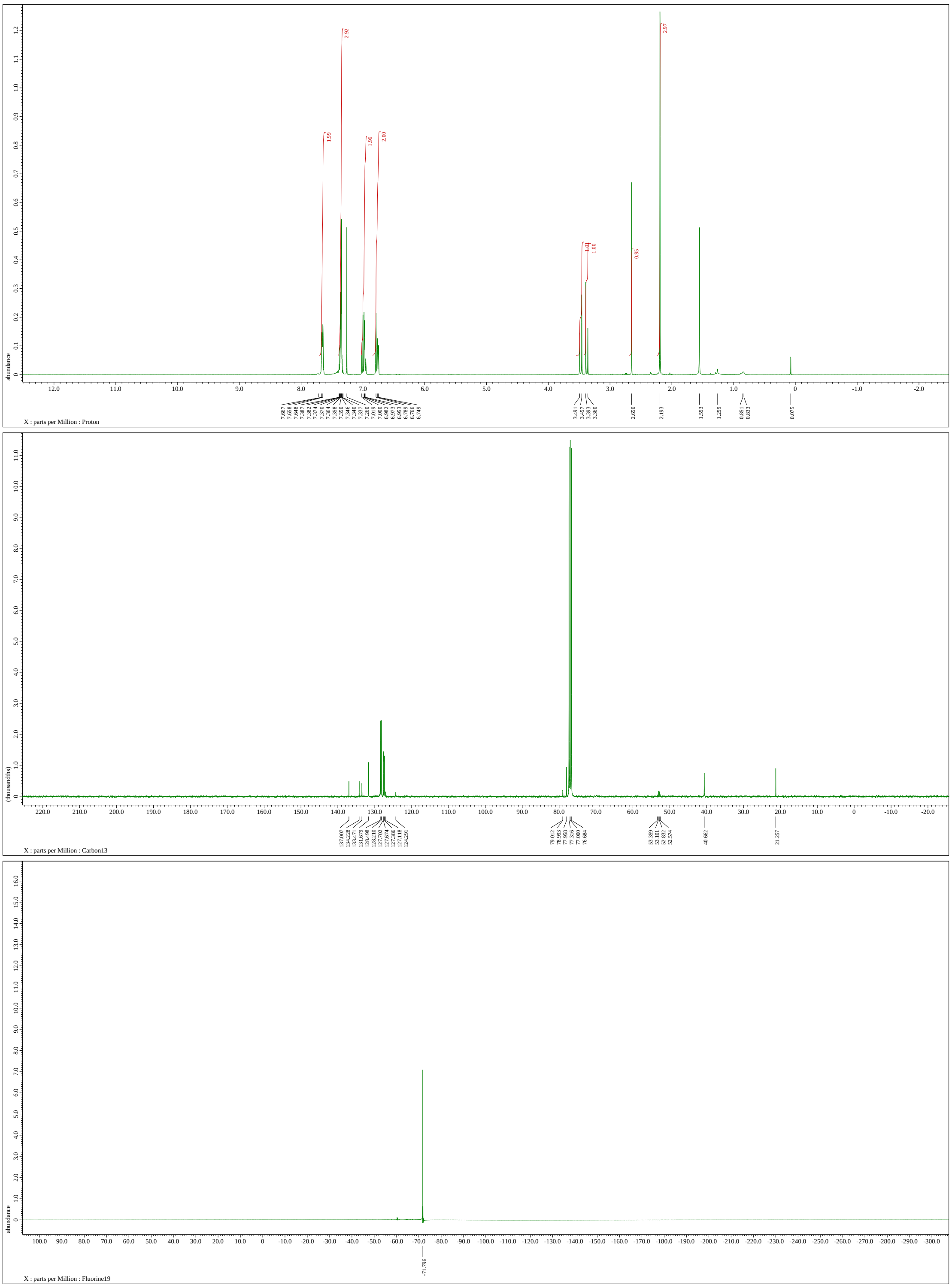

Supplementary Fig. 31: ^{1}H, ^{13}C, and ^{19}F NMR charts of **7al**.

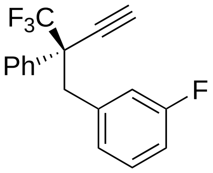

7am

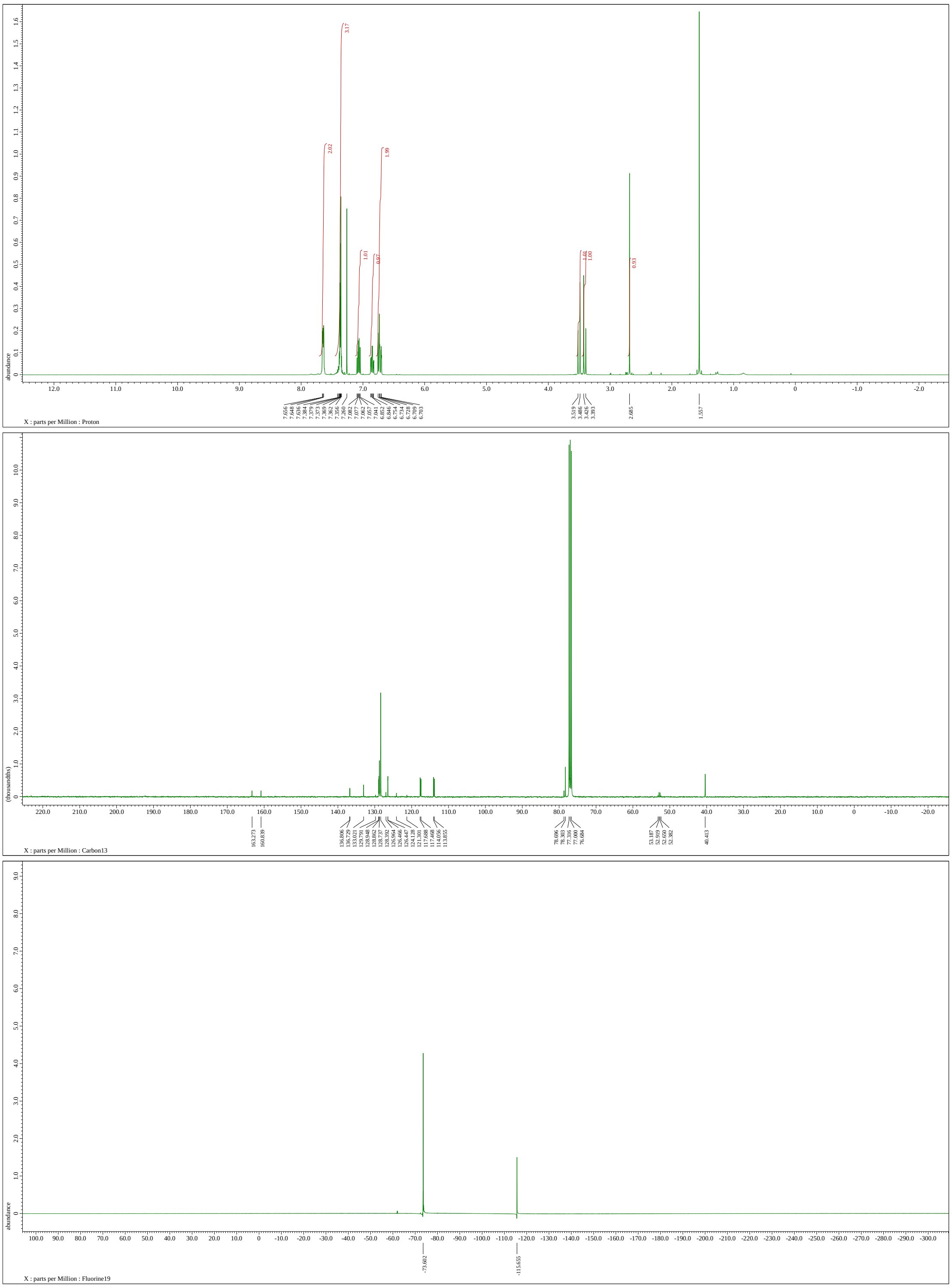

Supplementary Fig. 32: ^{1}H, ^{13}C, and ^{19}F NMR charts of **7am**.

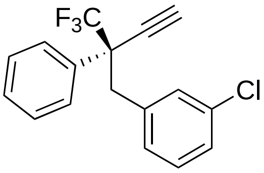

7an

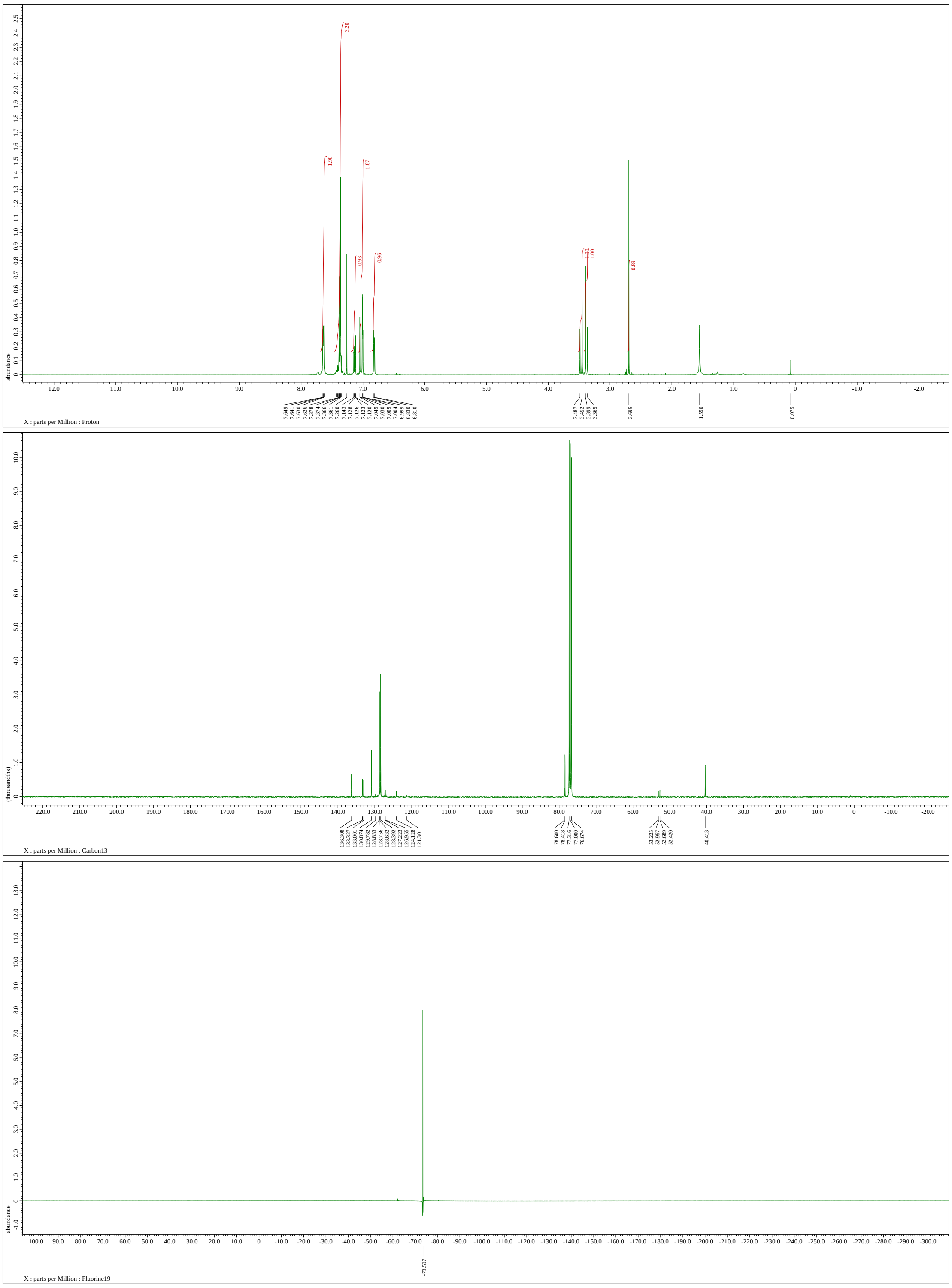

Supplementary Fig. 33: ^{1}H, ^{13}C, and ^{19}F NMR charts of **7an**.

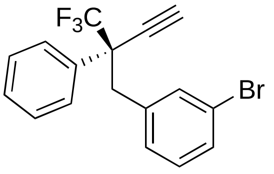

7ao

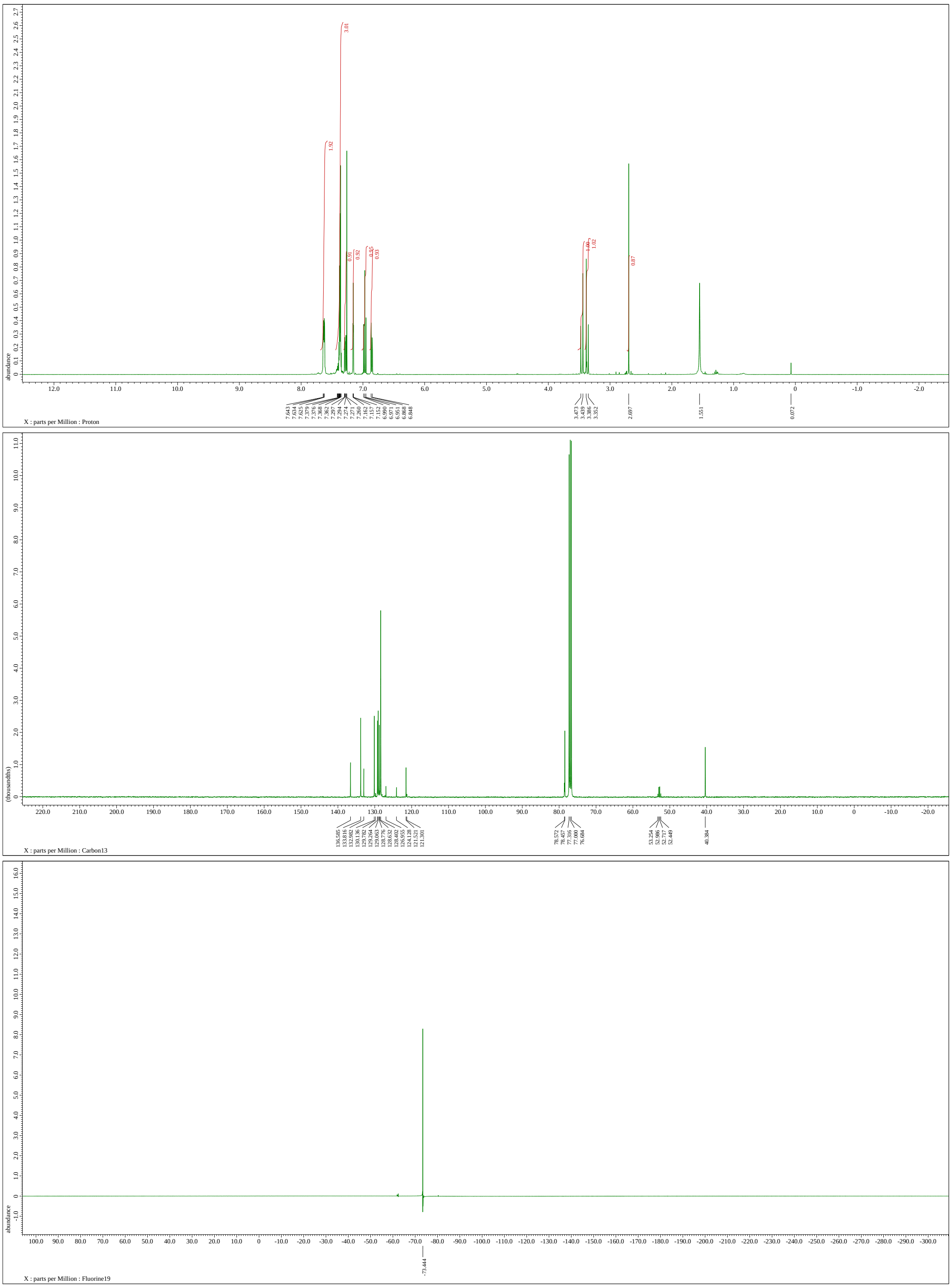

Supplementary Fig. 34: ^{1}H, ^{13}C, and ^{19}F NMR charts of **7ao**.

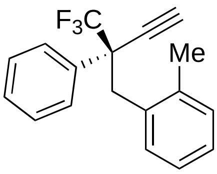

7ap

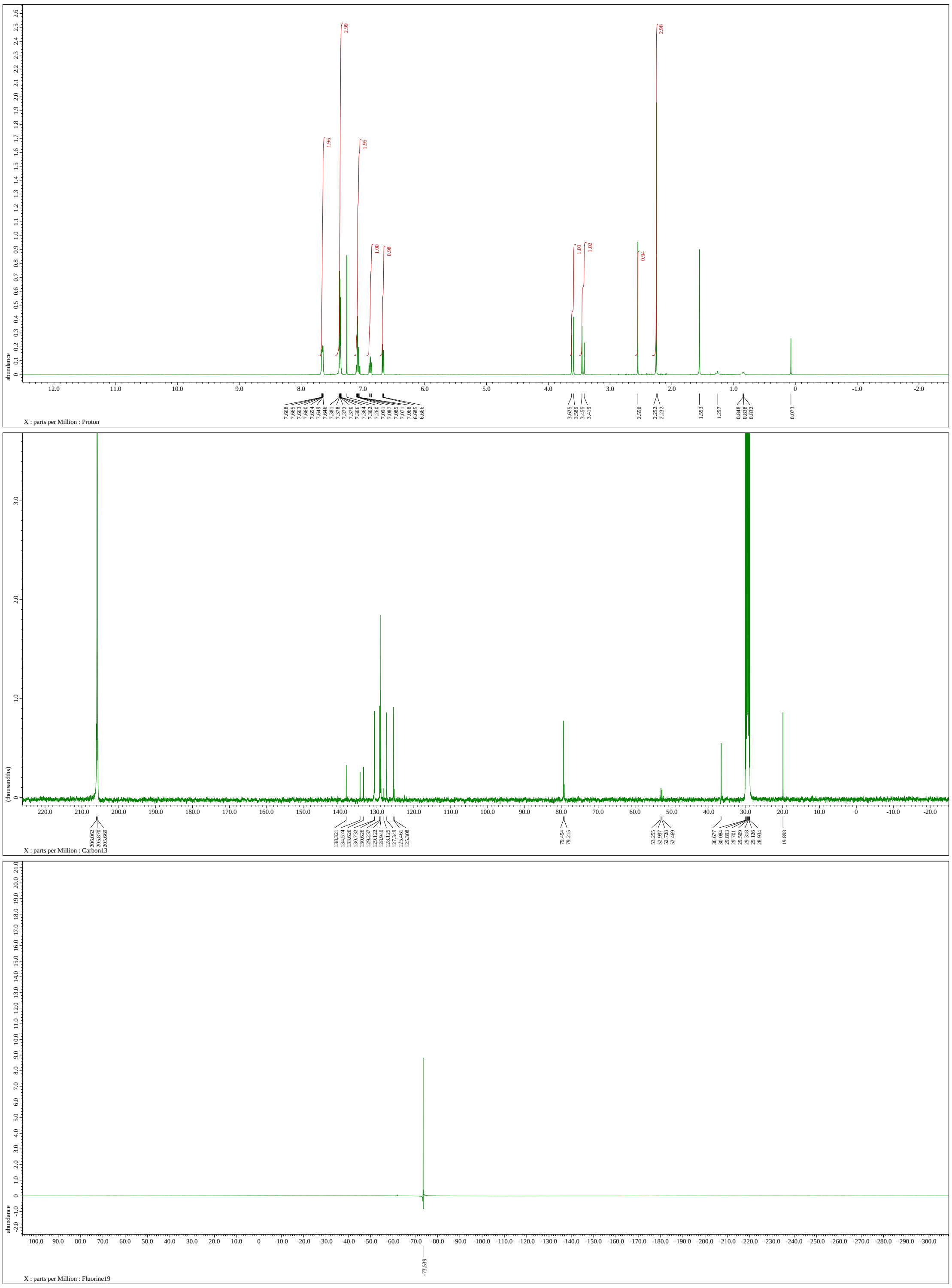

Supplementary Fig. 35: ^{1}H, ^{13}C, and ^{19}F NMR charts of **7ap**.

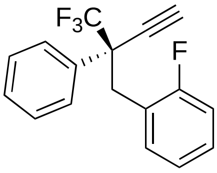

7aq

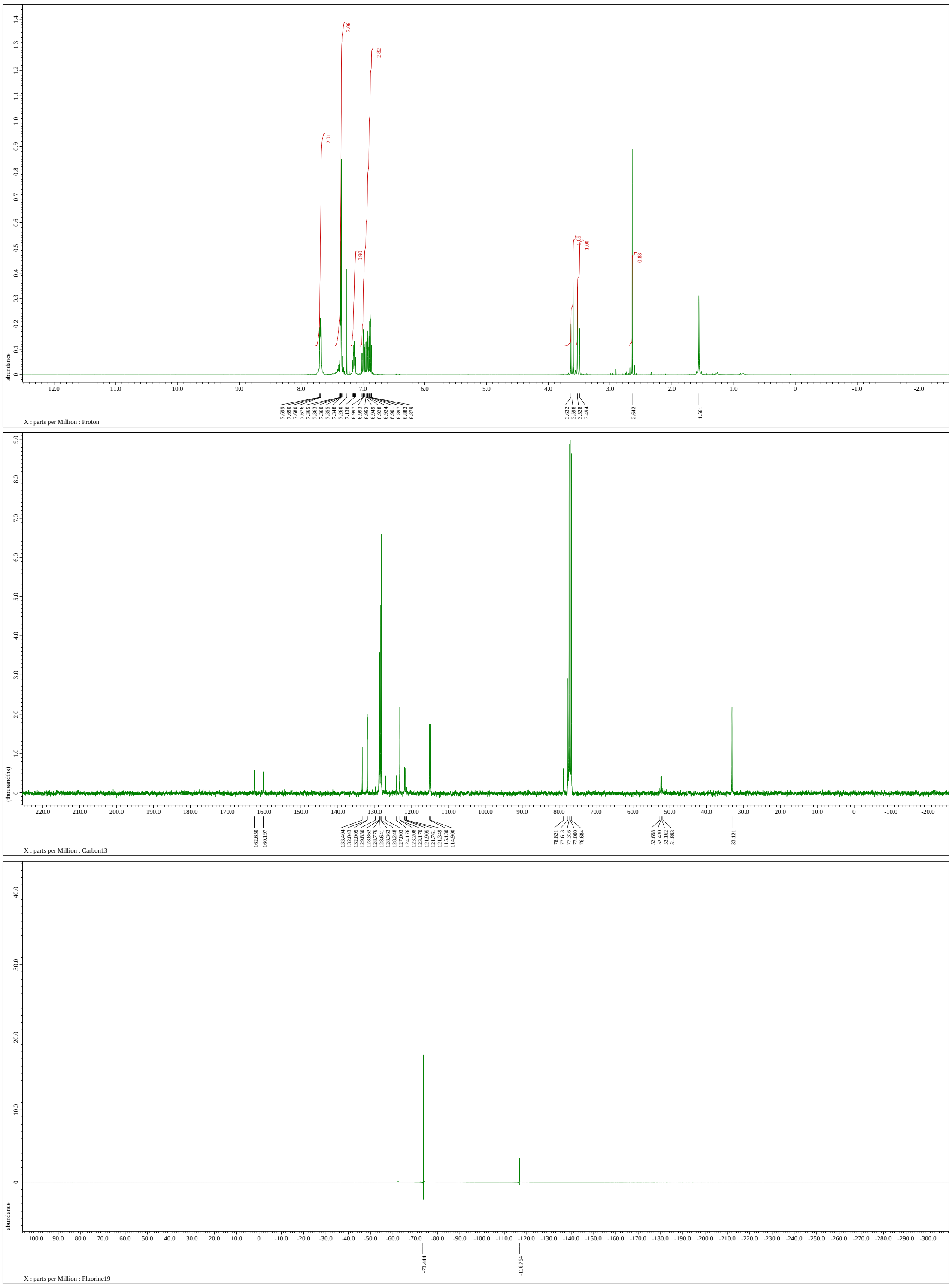

Supplementary Fig. 36: ^{1}H, ^{13}C, and ^{19}F NMR charts of **7aq**.

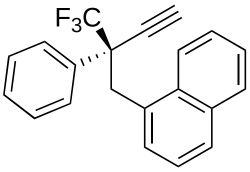

7ar

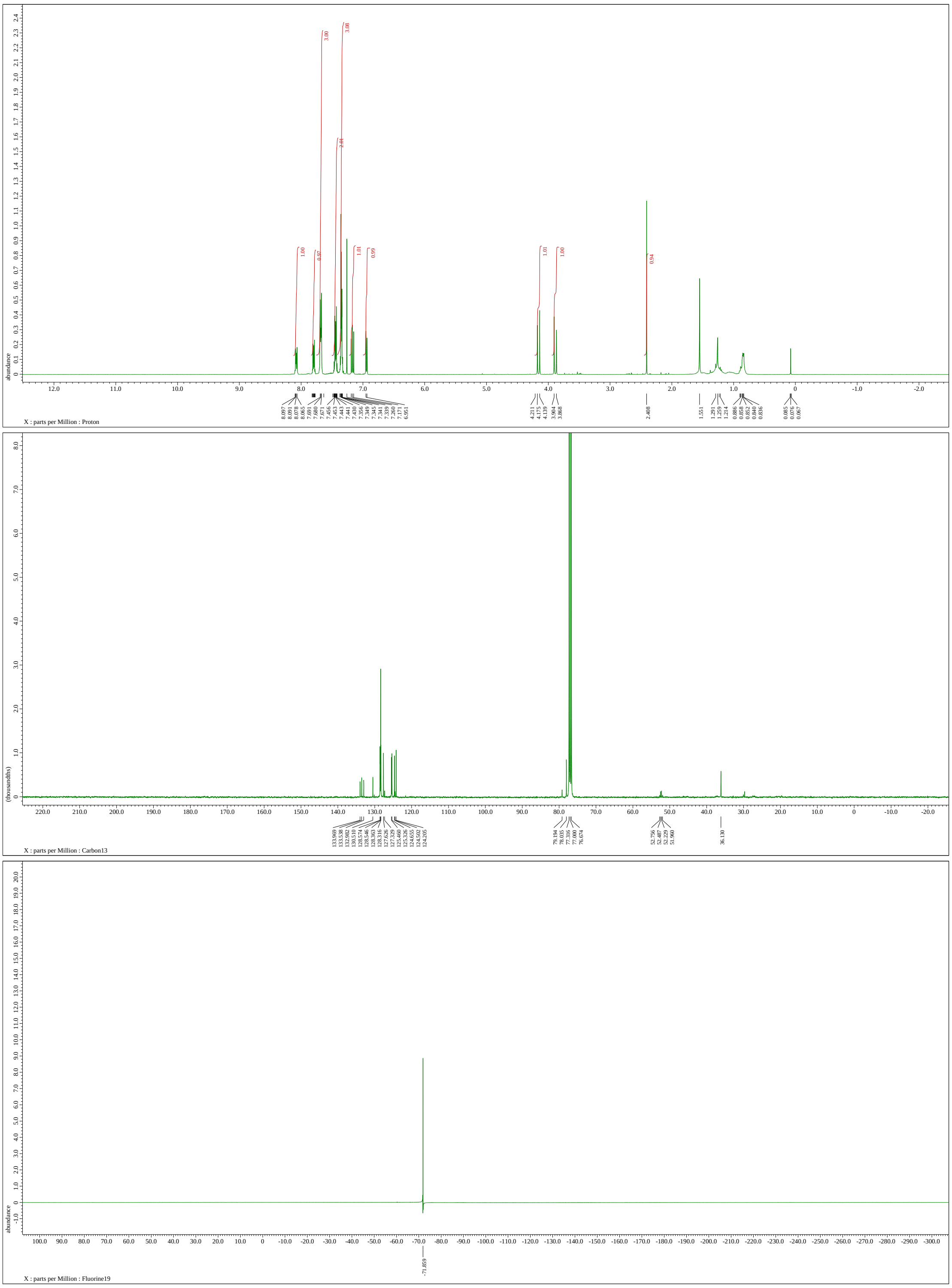

Supplementary Fig. 37: ^{1}H, ^{13}C, and ^{19}F NMR charts of **7ar**.

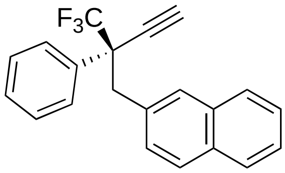

7as

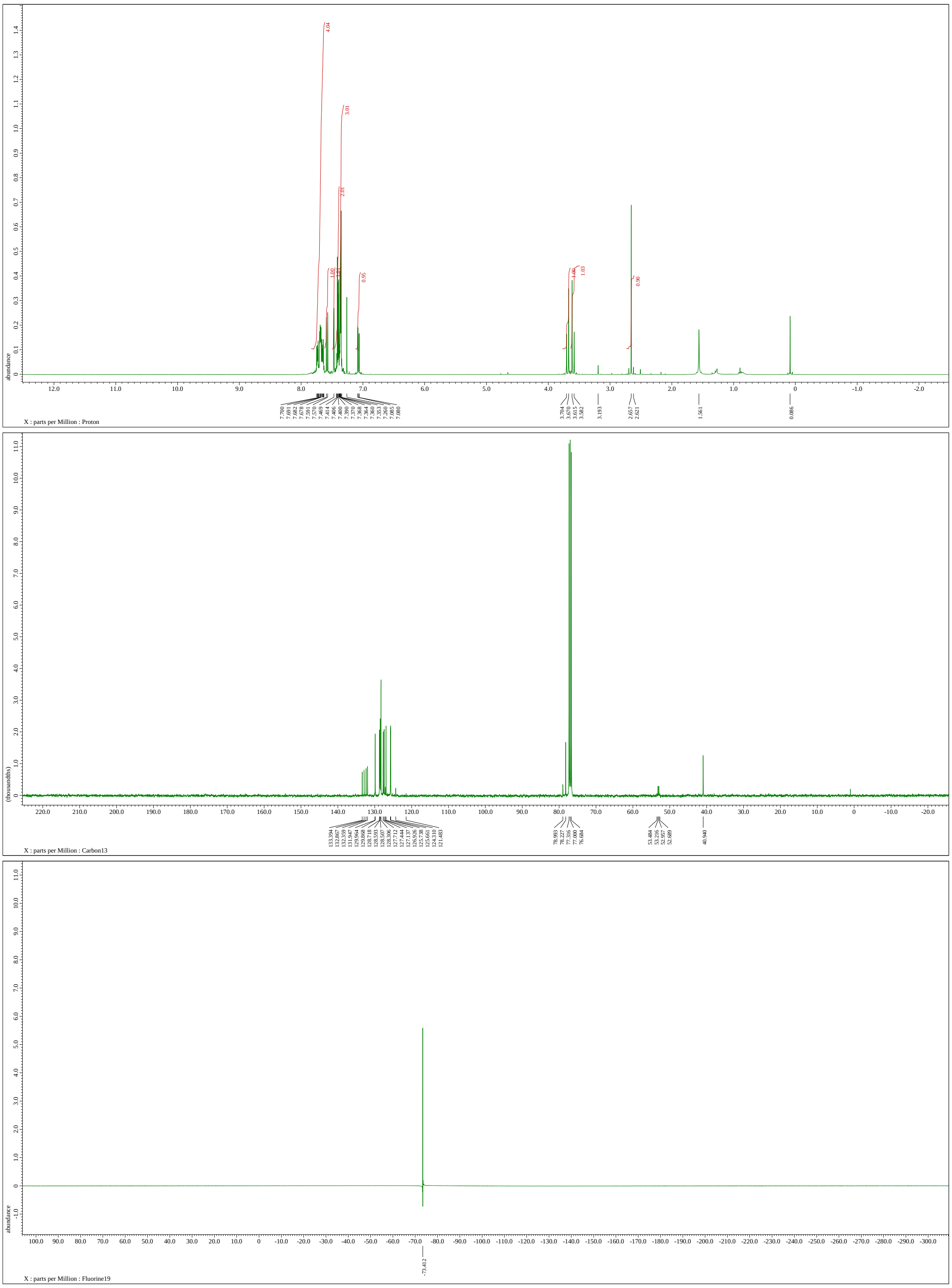

Supplementary Fig. 38: ^{1}H, ^{13}C, and ^{19}F NMR charts of **7as**.

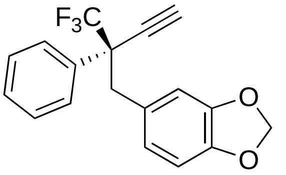

7at

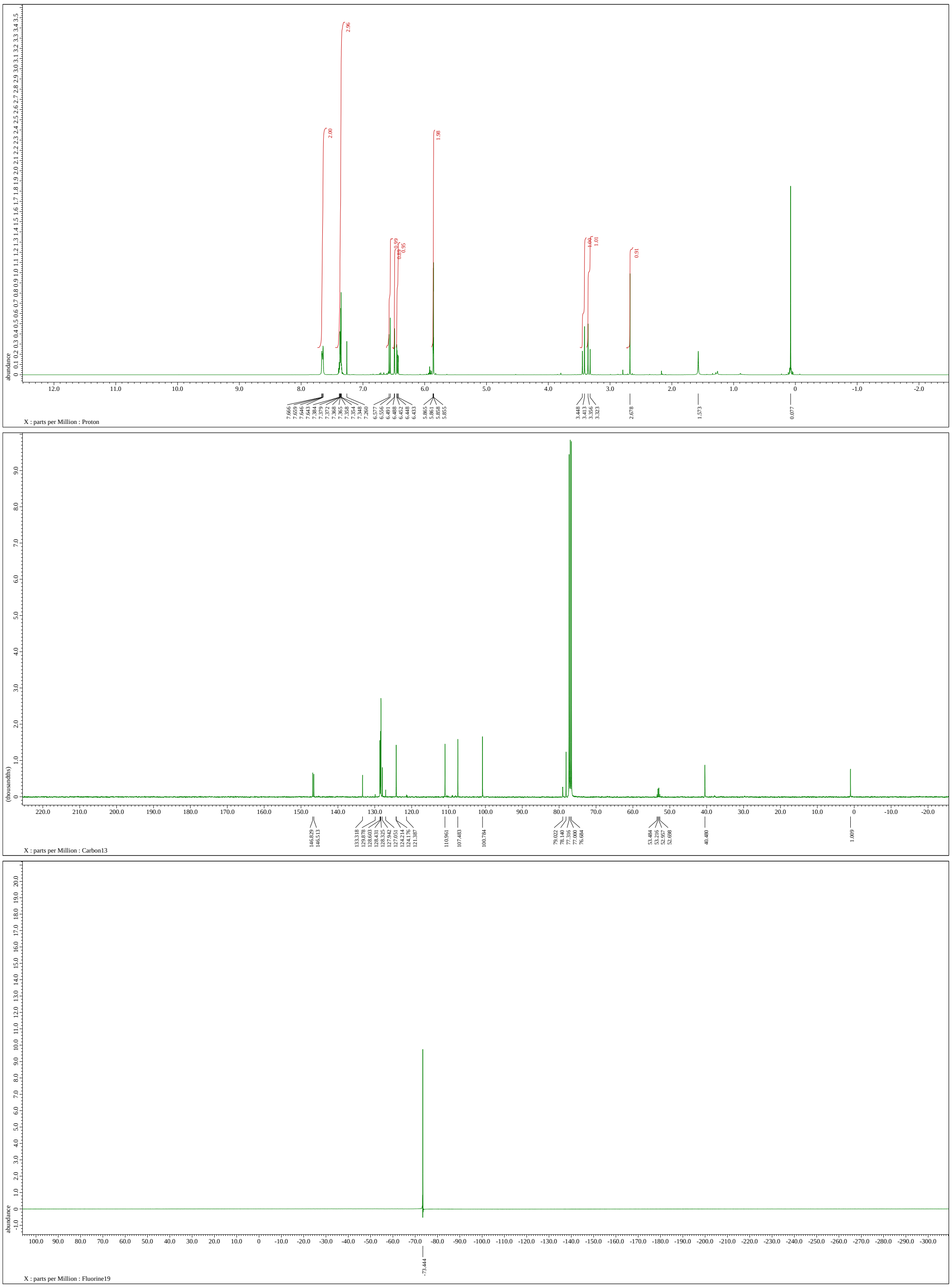

Supplementary Fig. 39: ^{1}H, ^{13}C, and ^{19}F NMR charts of **7at**.

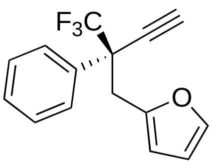

7au

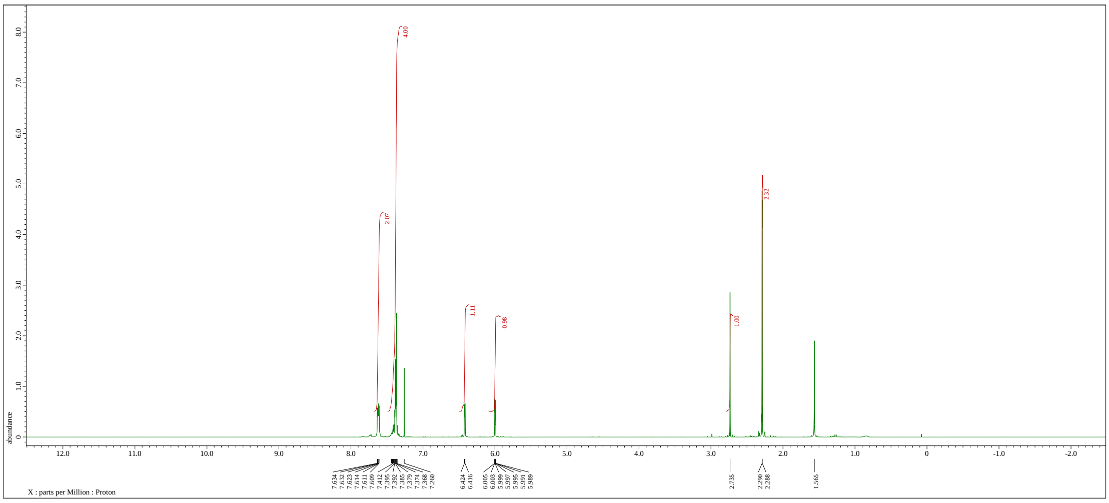

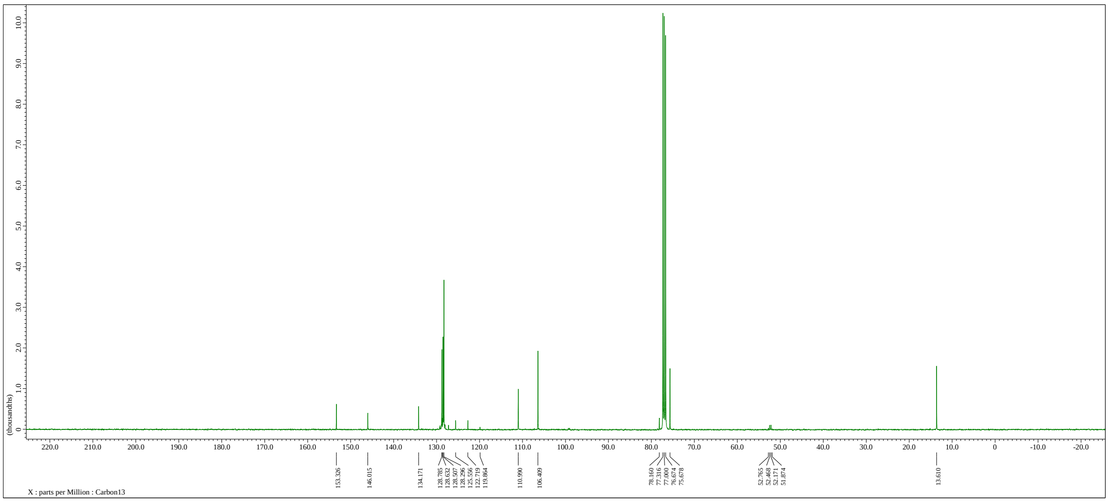

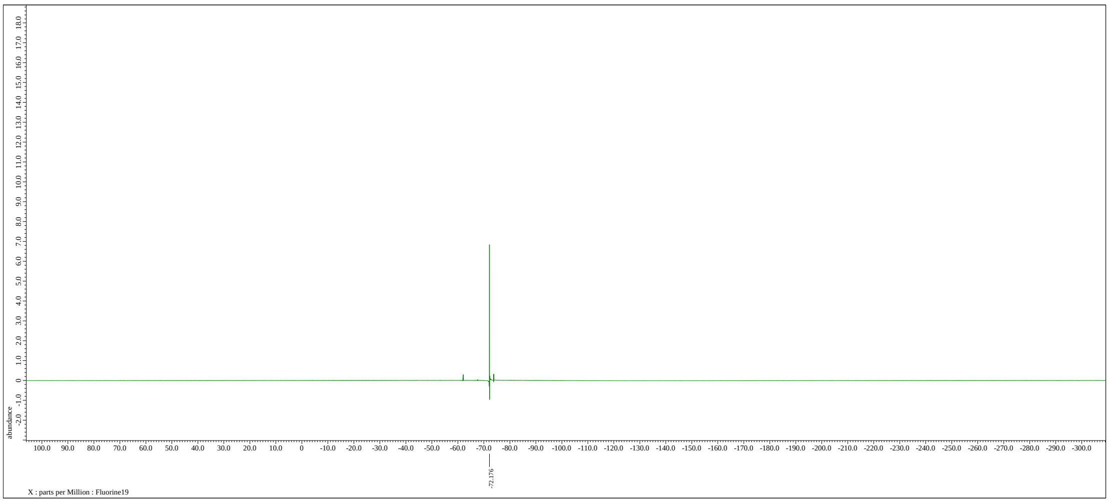

Supplementary Fig. 40: ^{1}H, ^{13}C, and ^{19}F NMR charts of **7au**.

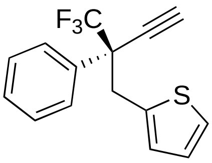

7av

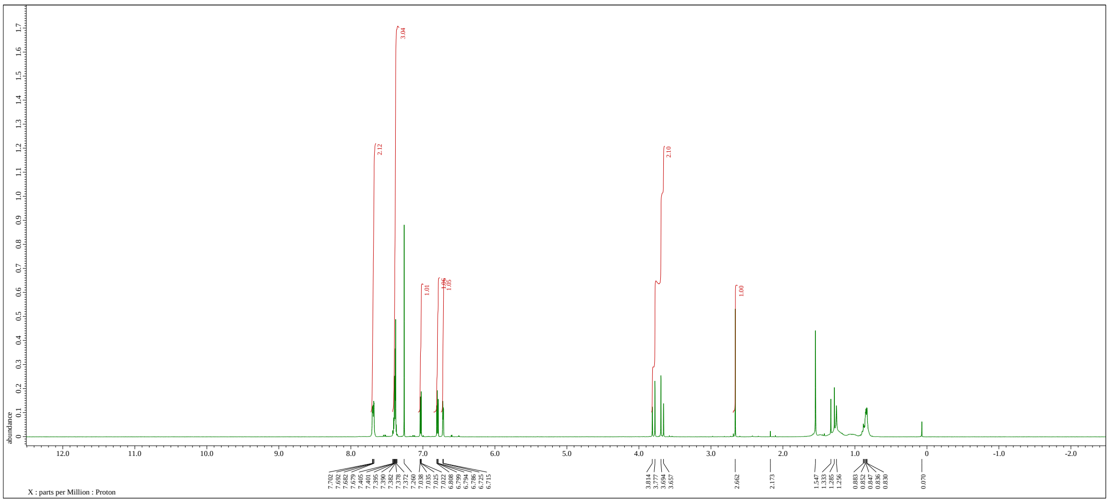

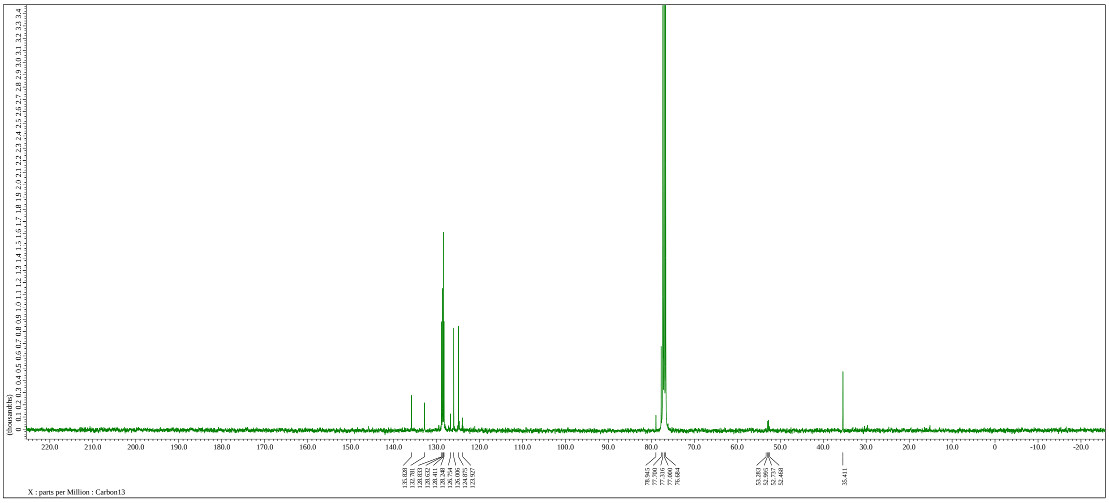

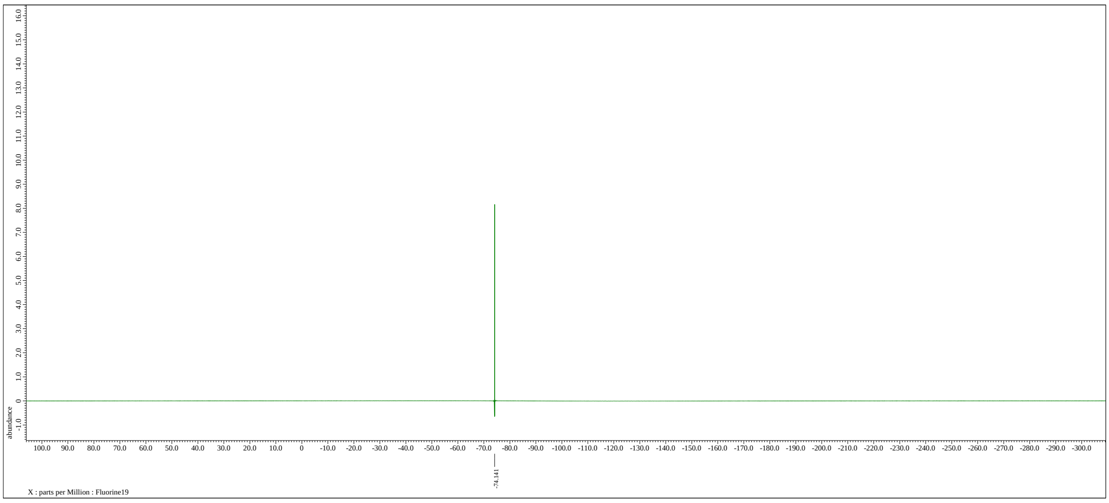

Supplementary Fig. 41: ^{1}H, ^{13}C, and ^{19}F NMR charts of **7av**.

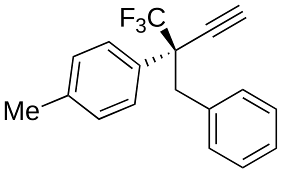

7ba

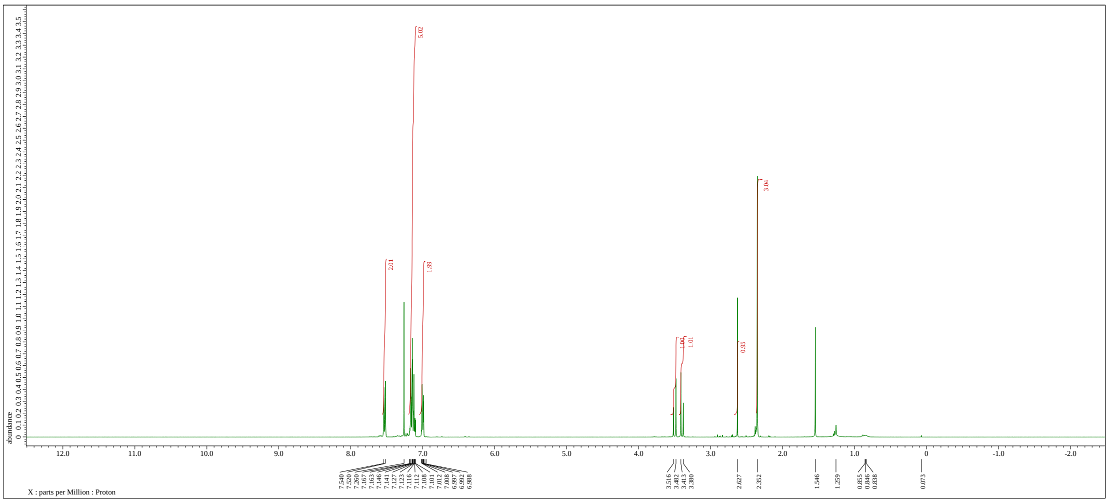

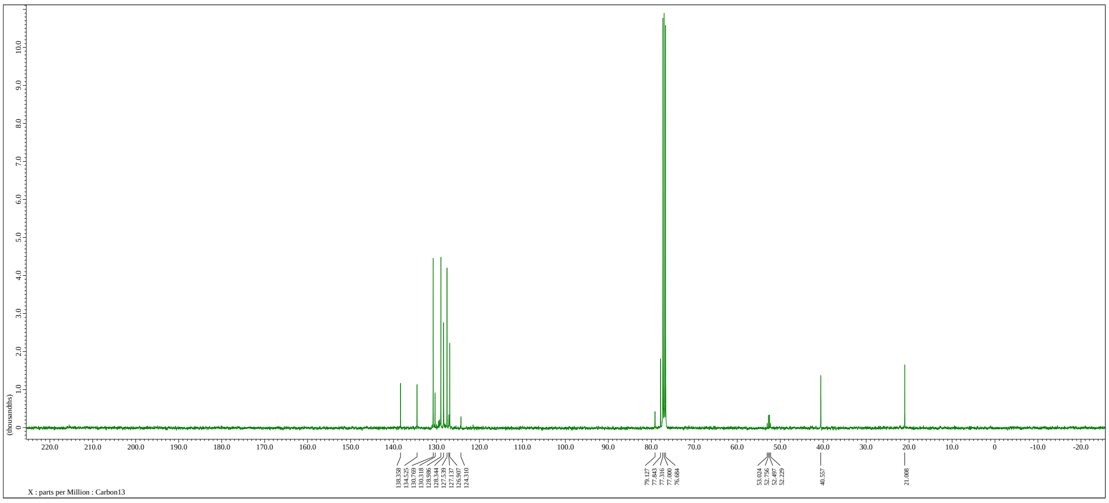

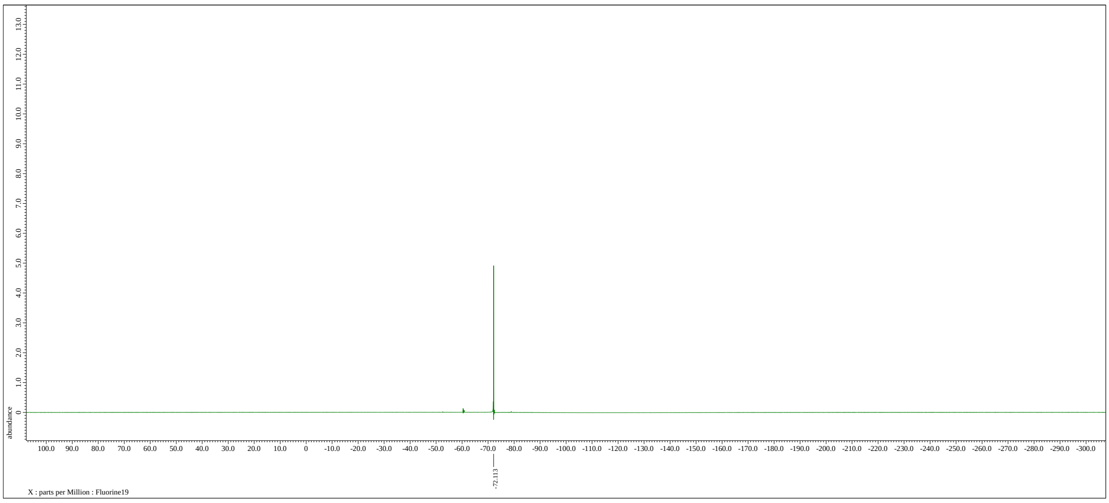

Supplementary Fig. 42: ^{1}H, ^{13}C, and ^{19}F NMR charts of **7ba**.

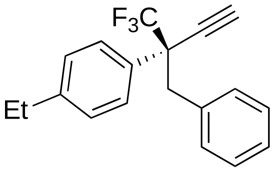

7ca

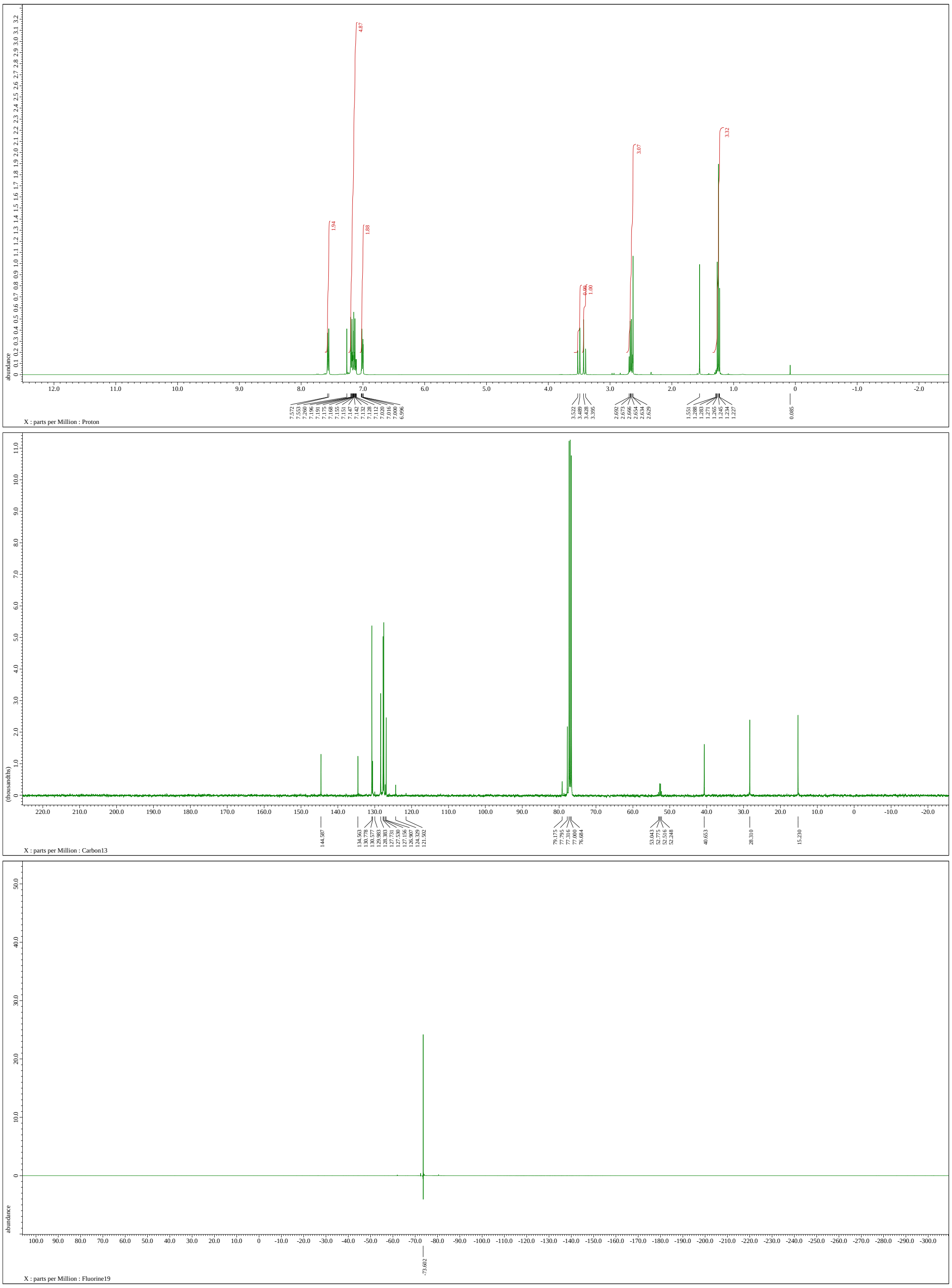

Supplementary Fig. 43: ^{1}H, ^{13}C, and ^{19}F NMR charts of **7ca**.

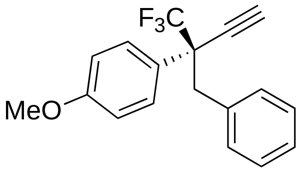

7da

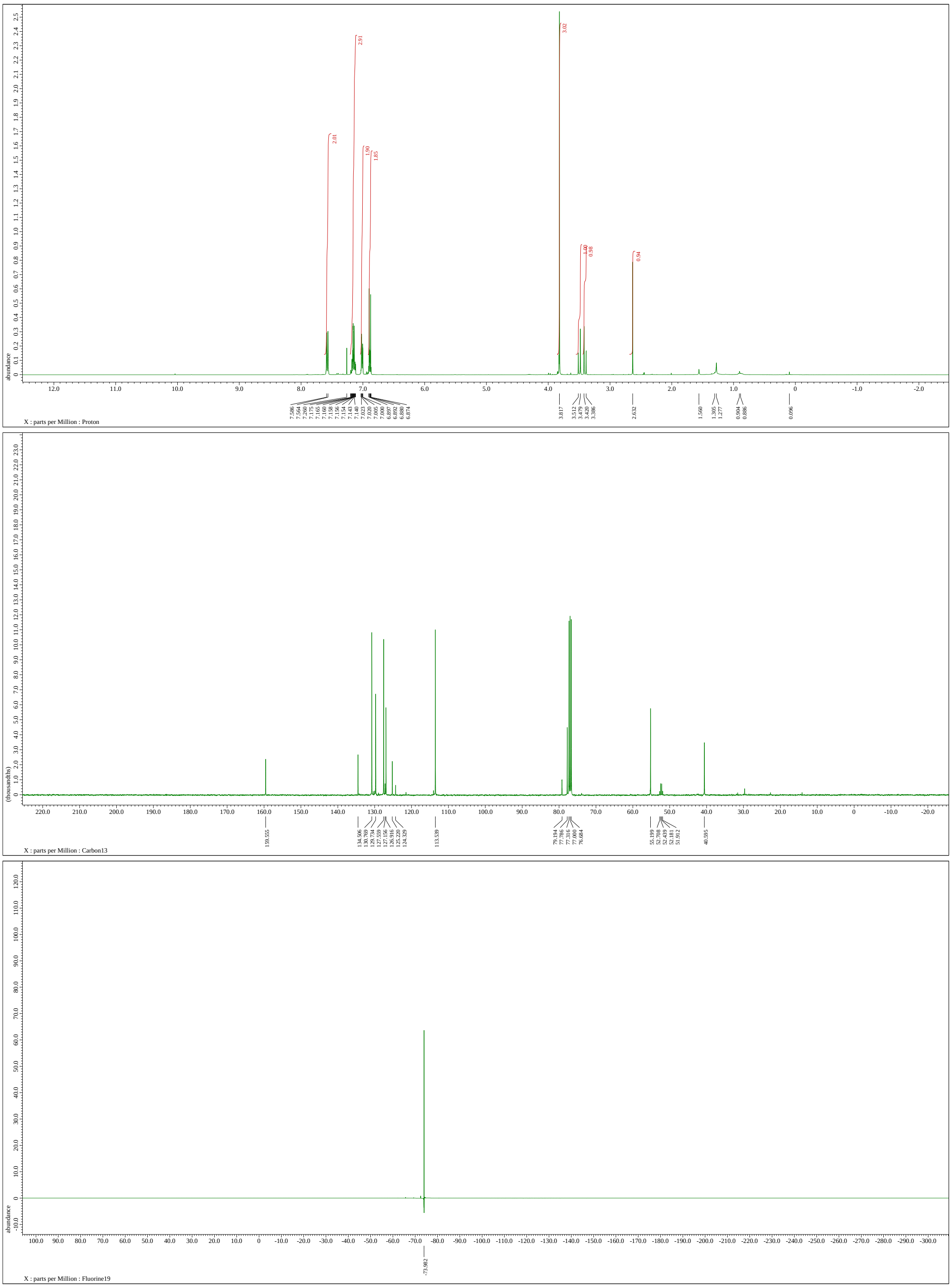

Supplementary Fig. 44: ^{1}H, ^{13}C, and ^{19}F NMR charts of **7da**.

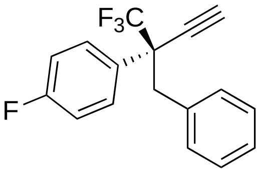

7ea

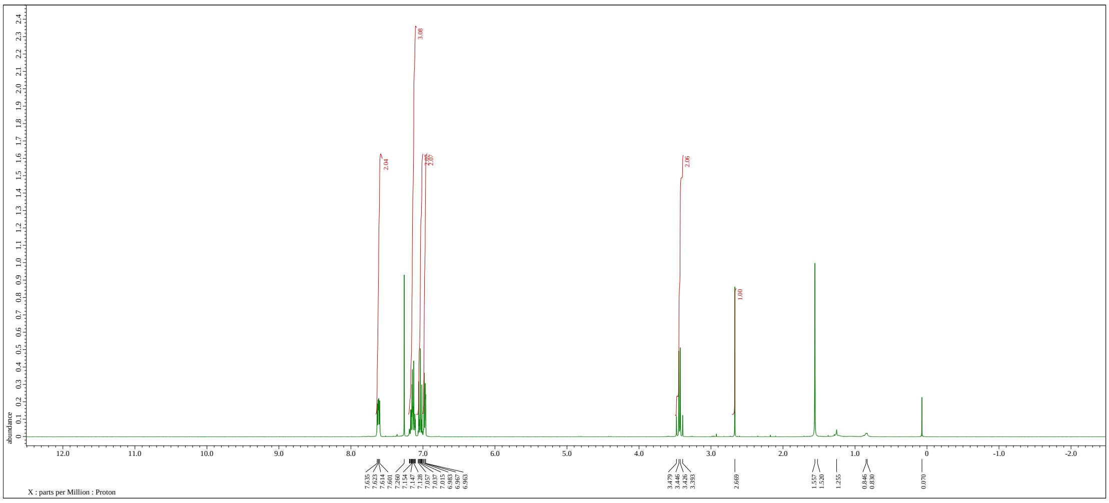

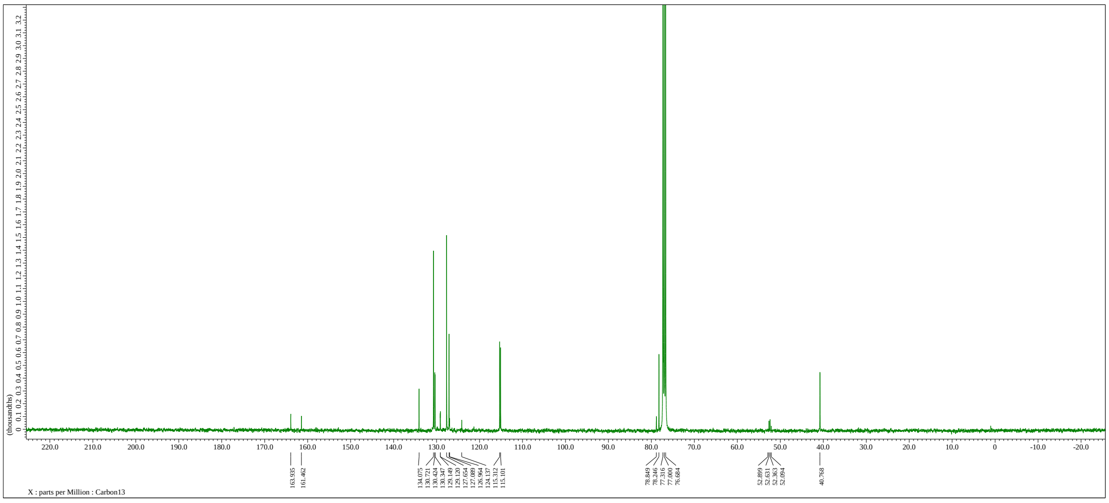

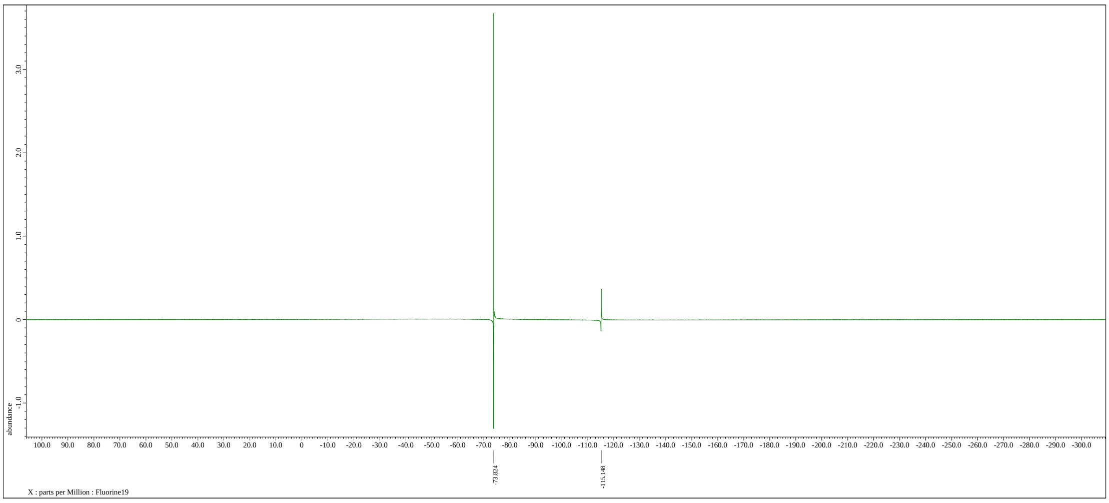

Supplementary Fig. 45: ^{1}H, ^{13}C, and ^{19}F NMR charts of **7ea**.

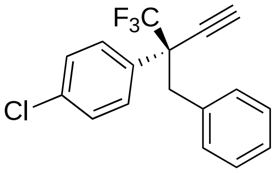

7fa

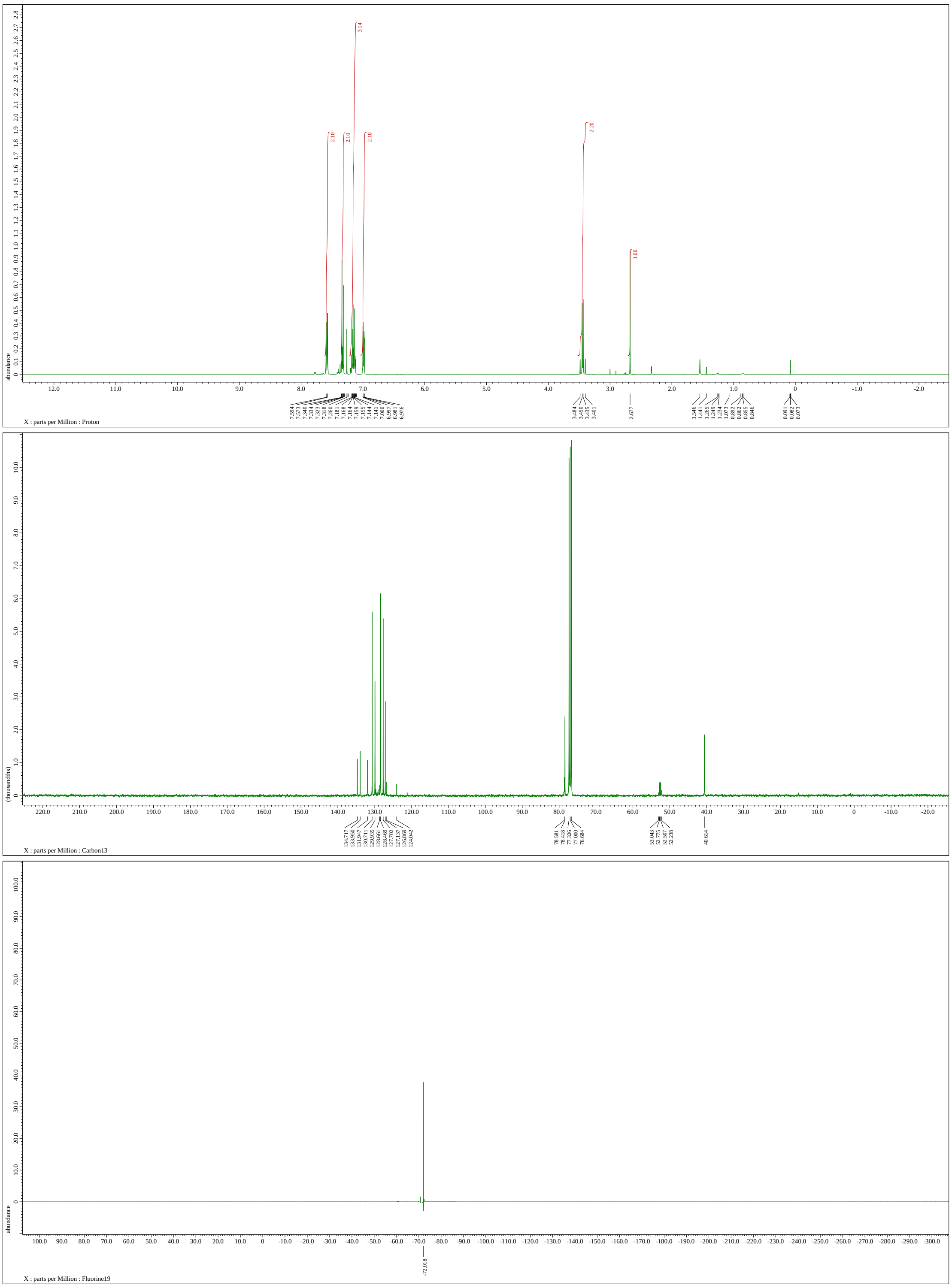

Supplementary Fig. 46: ^{1}H, ^{13}C, and ^{19}F NMR charts of **7fa**.

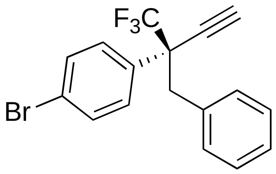

7ga

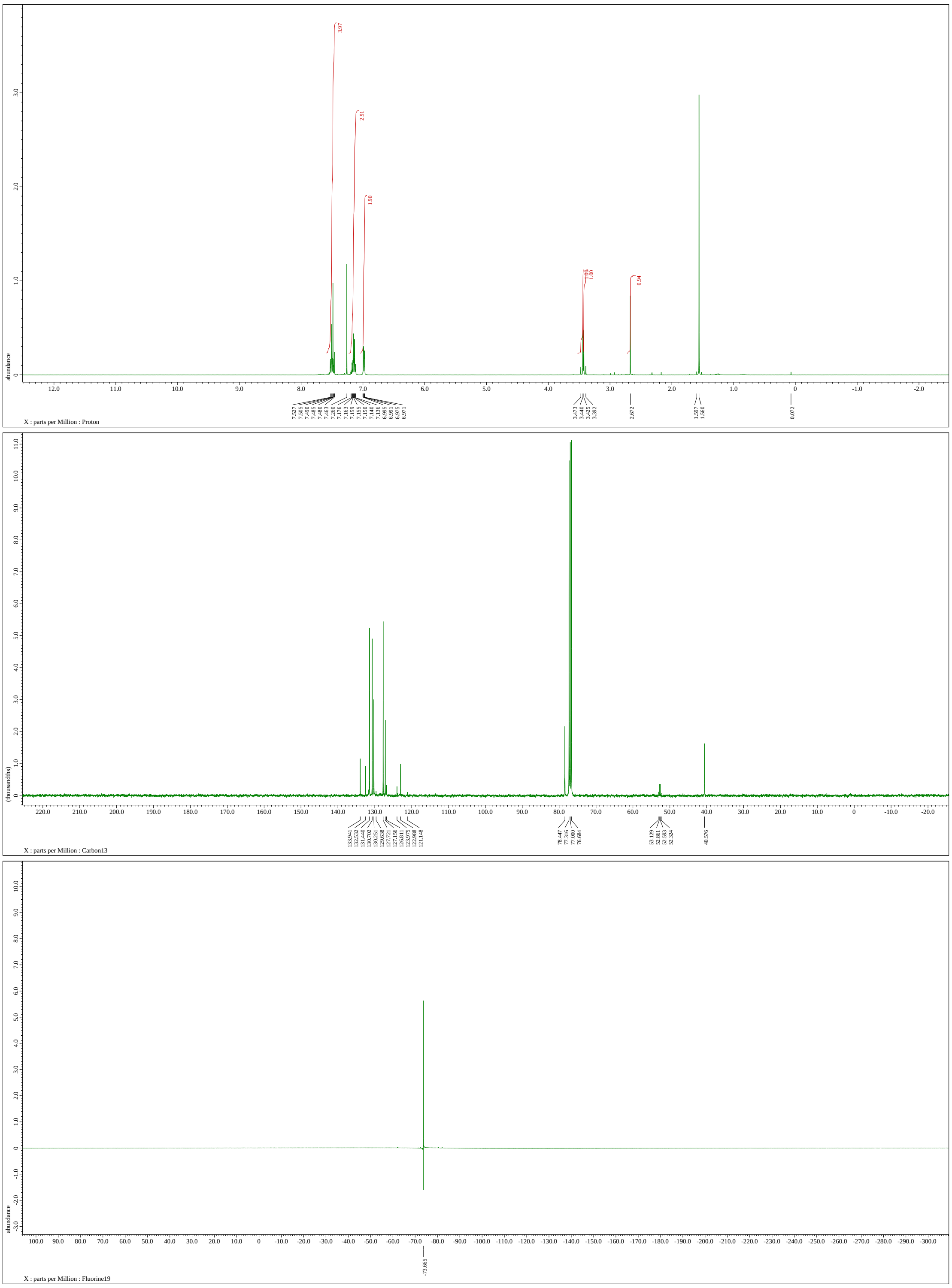

Supplementary Fig. 47: ^{1}H, ^{13}C, and ^{19}F NMR charts of **7ga**.

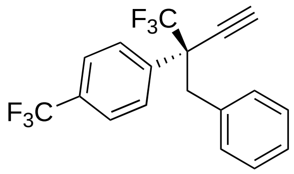

7ha

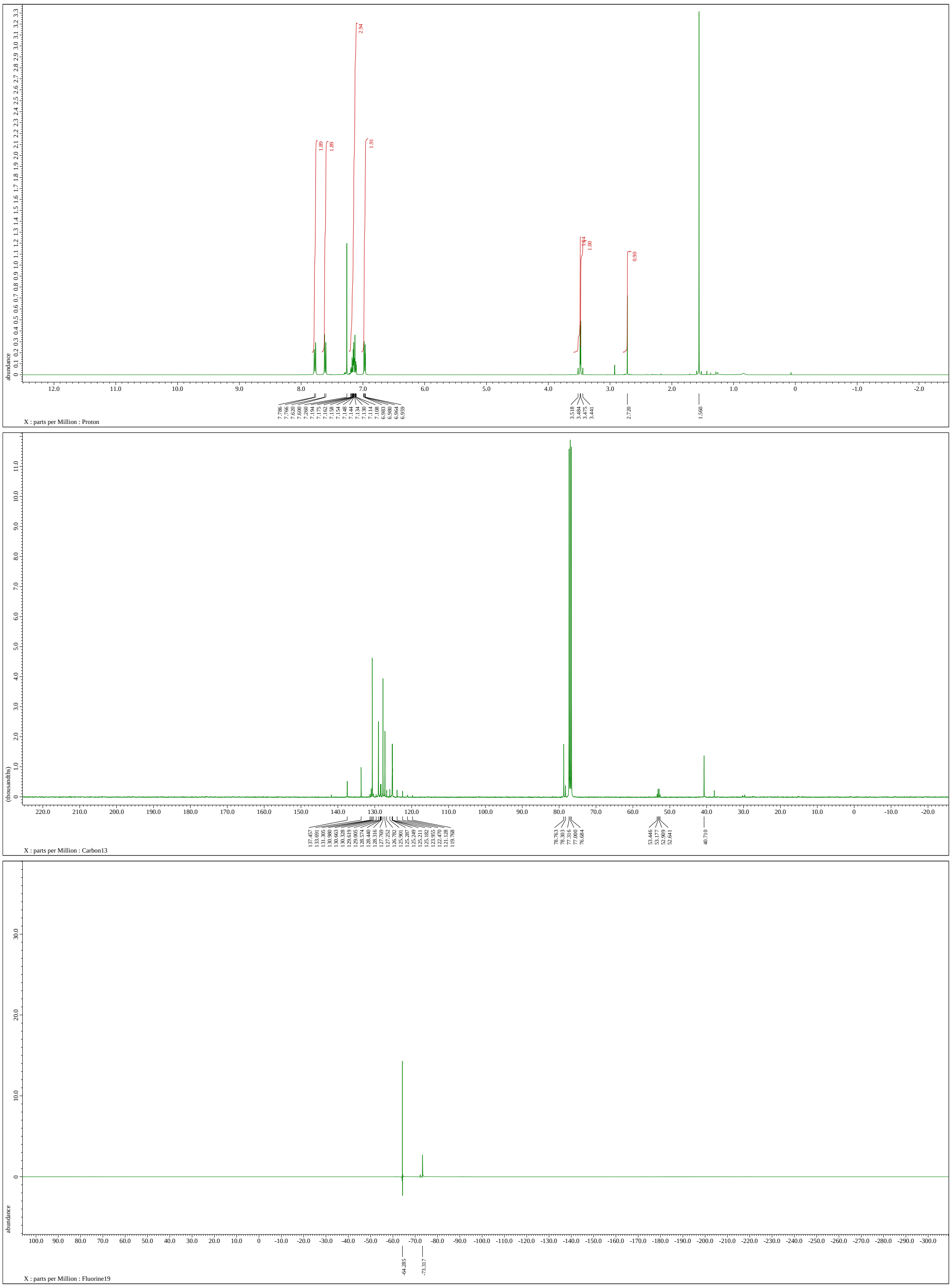

Supplementary Fig. 48: ^{1}H, ^{13}C, and ^{19}F NMR charts of **7ha**.

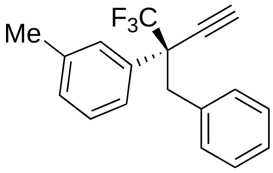

7ia

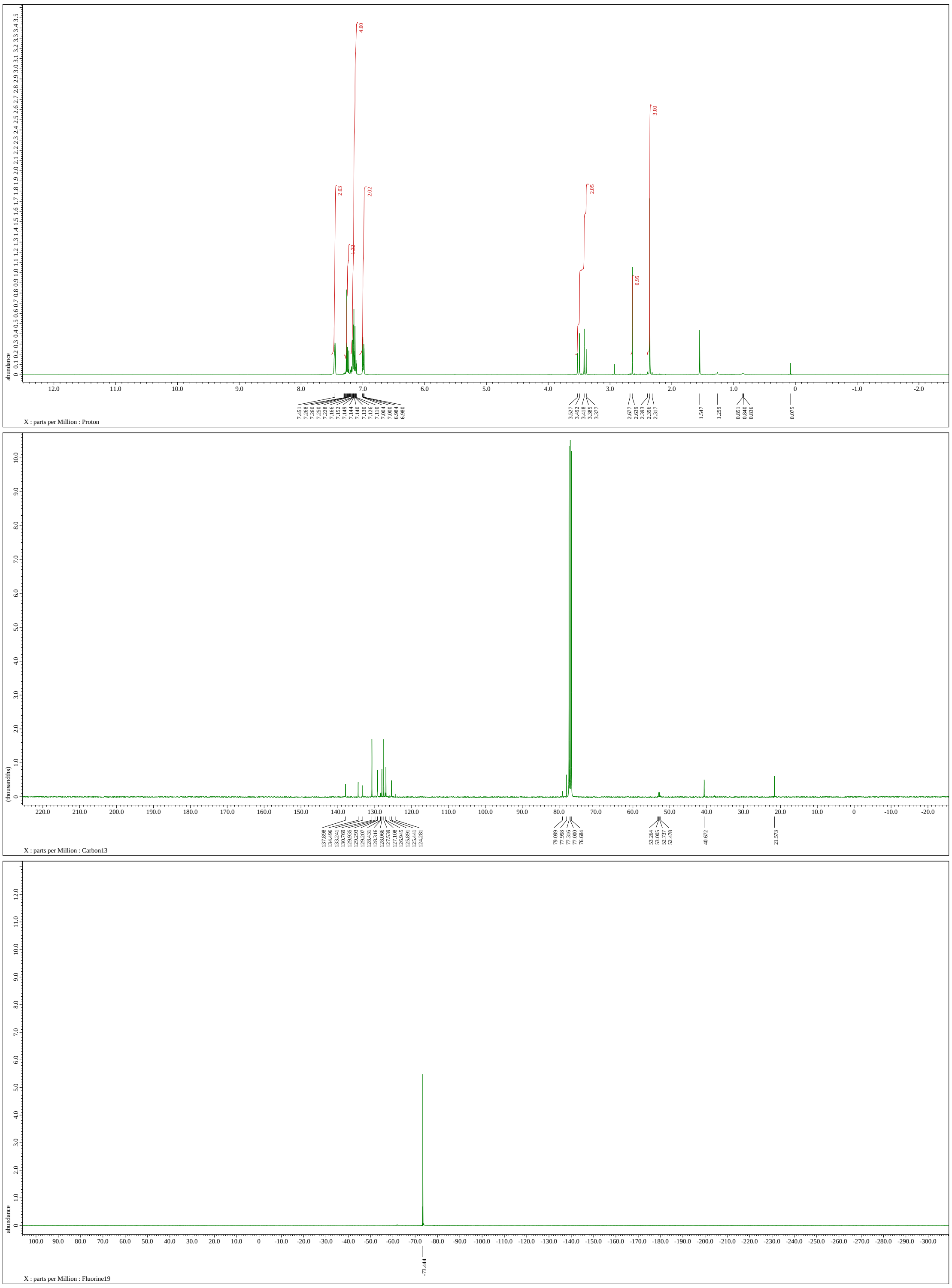

Supplementary Fig. 49: ^{1}H, ^{13}C, and ^{19}F NMR charts of **7ia**.

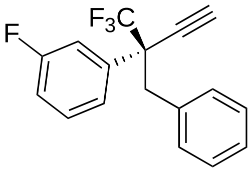

7ja

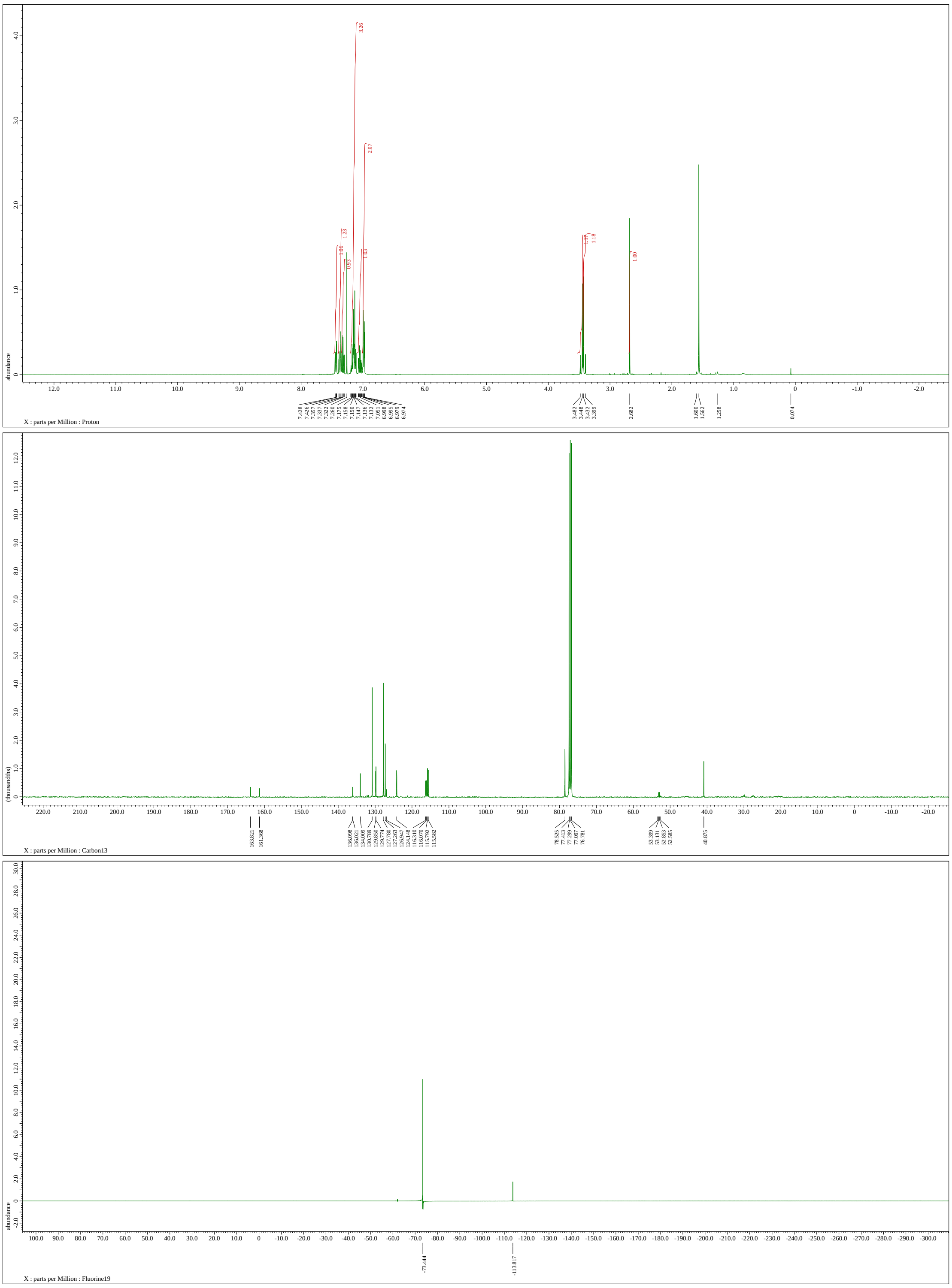

Supplementary Fig. 50: ^{1}H, ^{13}C, and ^{19}F NMR charts of **7ja**.

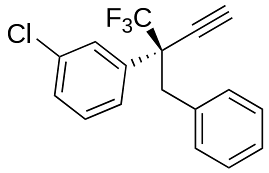

7ka

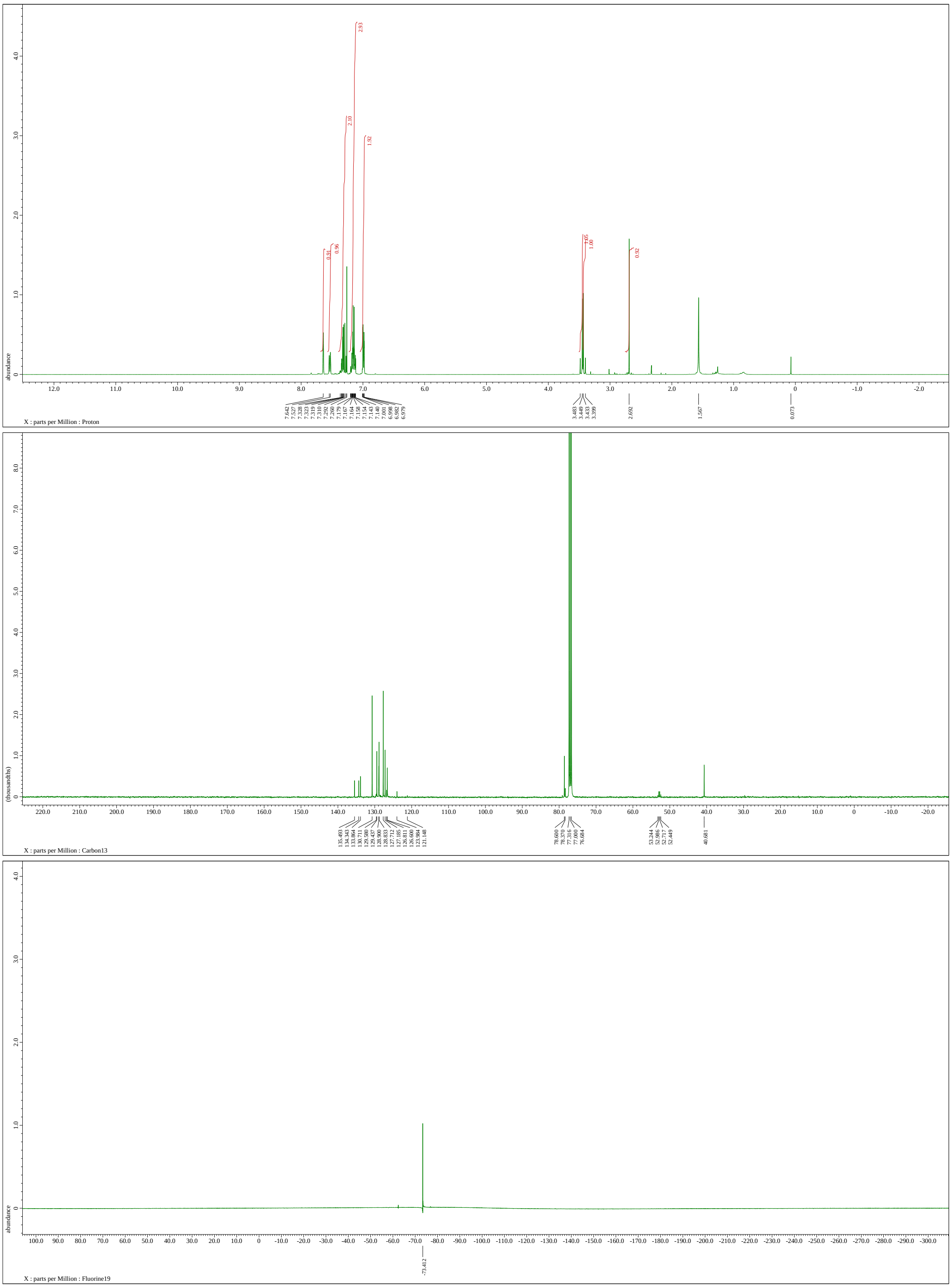

Supplementary Fig. 51: ^{1}H, ^{13}C, and ^{19}F NMR charts of **7ka**.

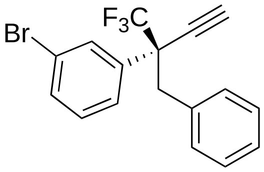

7la

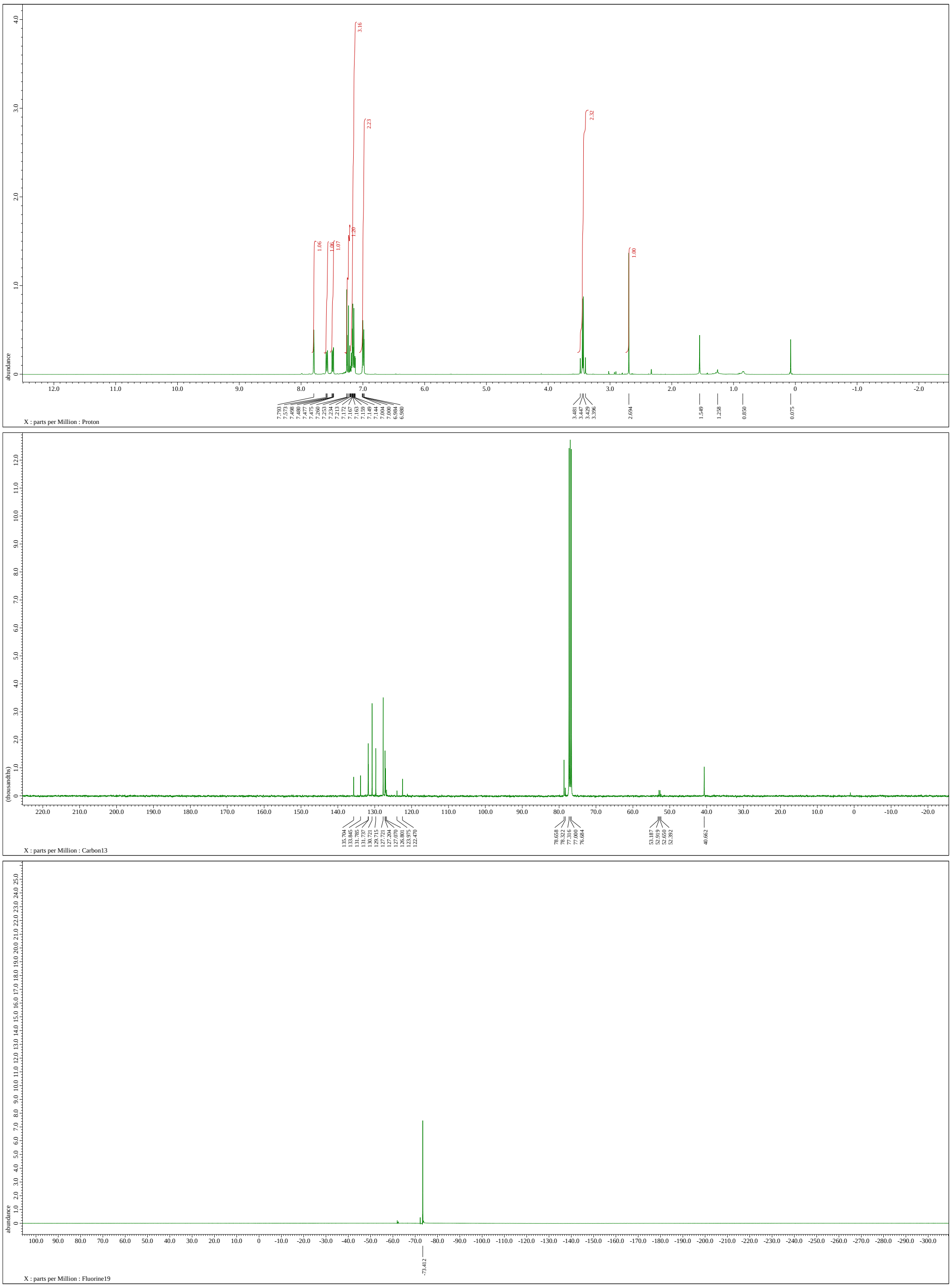

Supplementary Fig. 52: ^{1}H, ^{13}C, and ^{19}F NMR charts of **7la**.

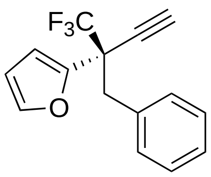

7ma

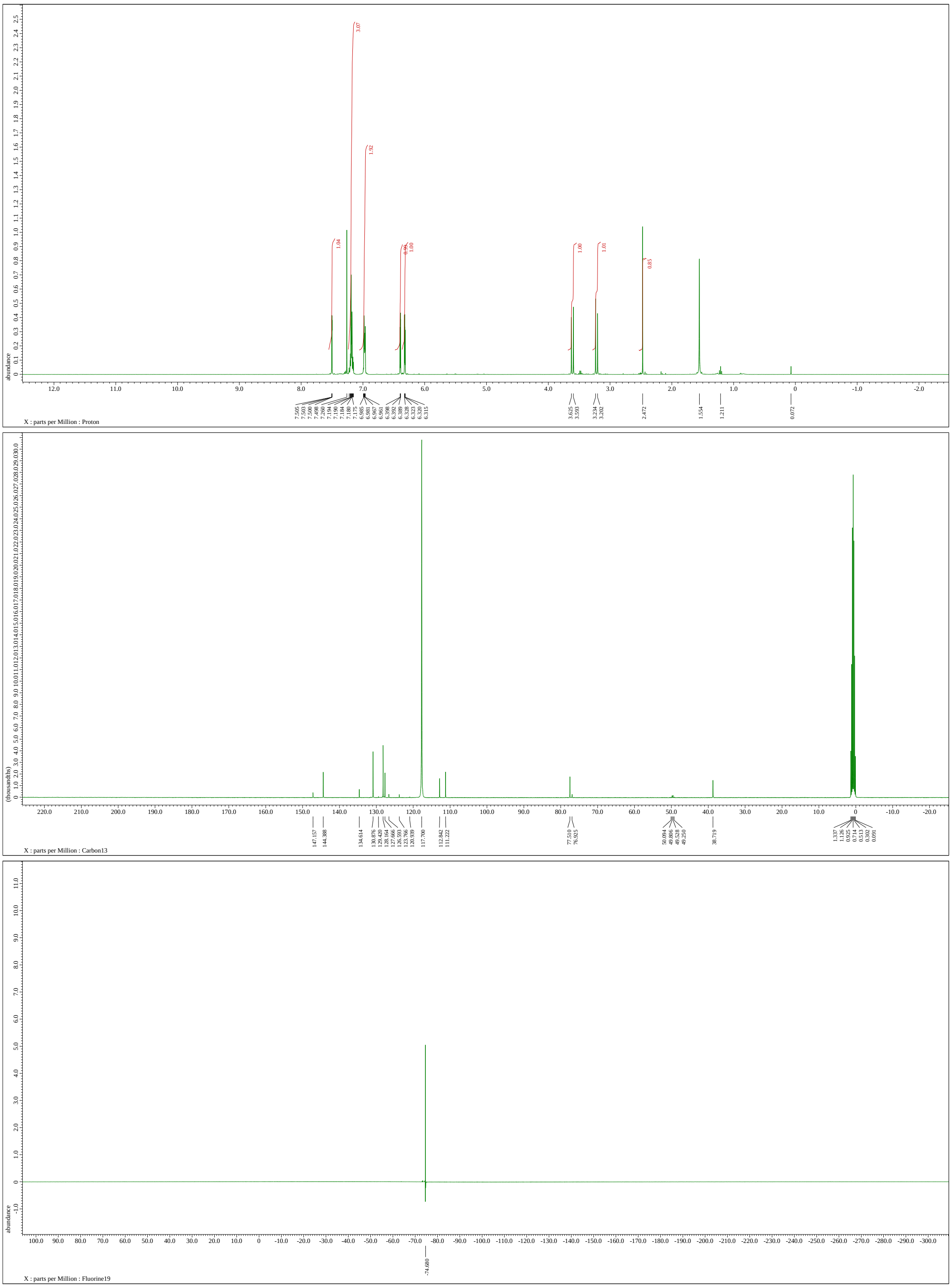

Supplementary Fig. 53: ^{1}H, ^{13}C, and ^{19}F NMR charts of **7ma**.

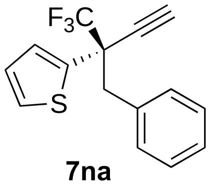

7na

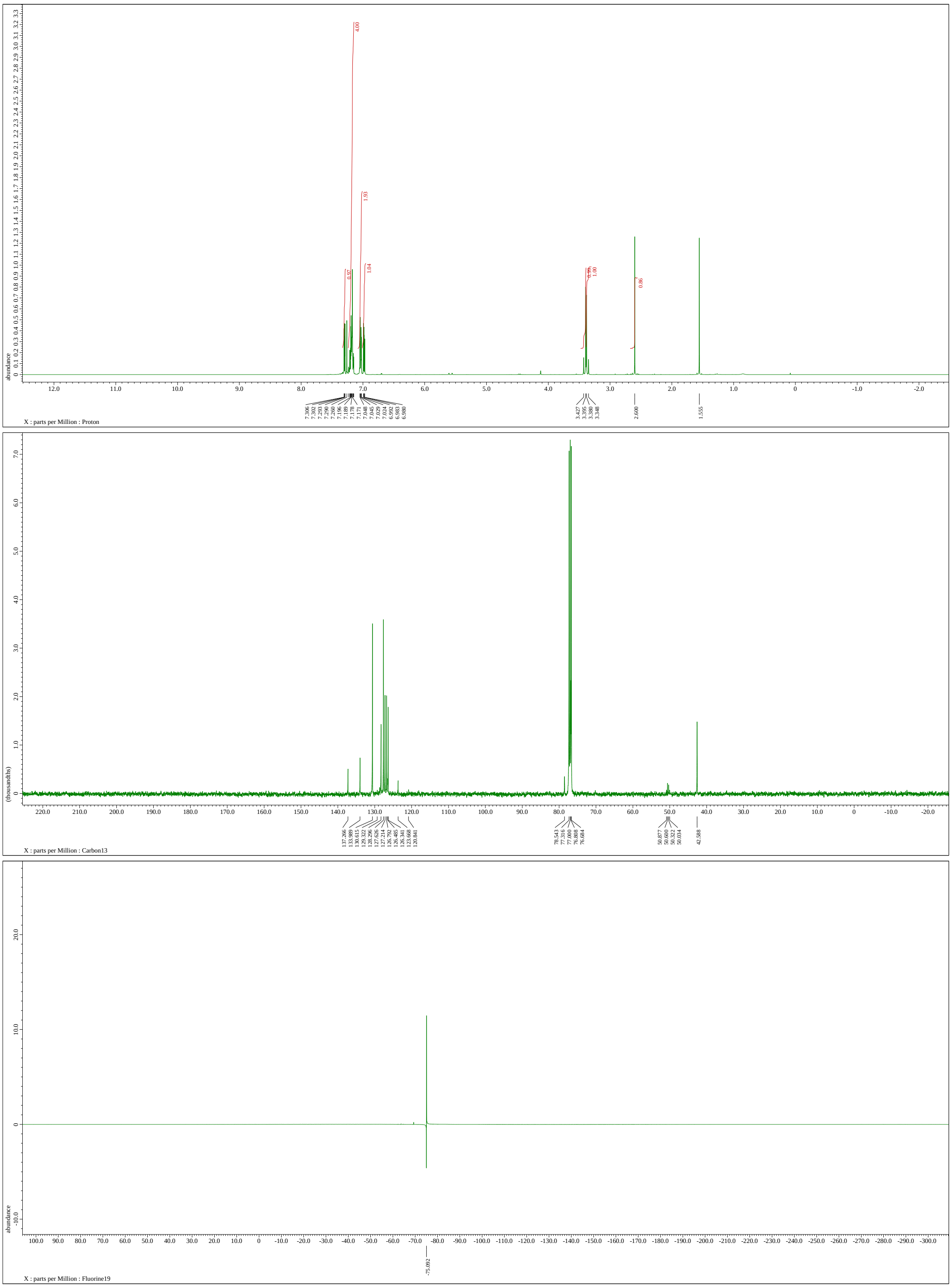

Supplementary Fig. 54: ^{1}H, ^{13}C, and ^{19}F NMR charts of **7na**.

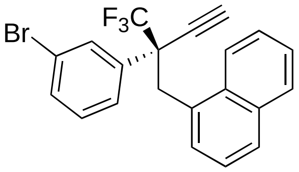

7lr

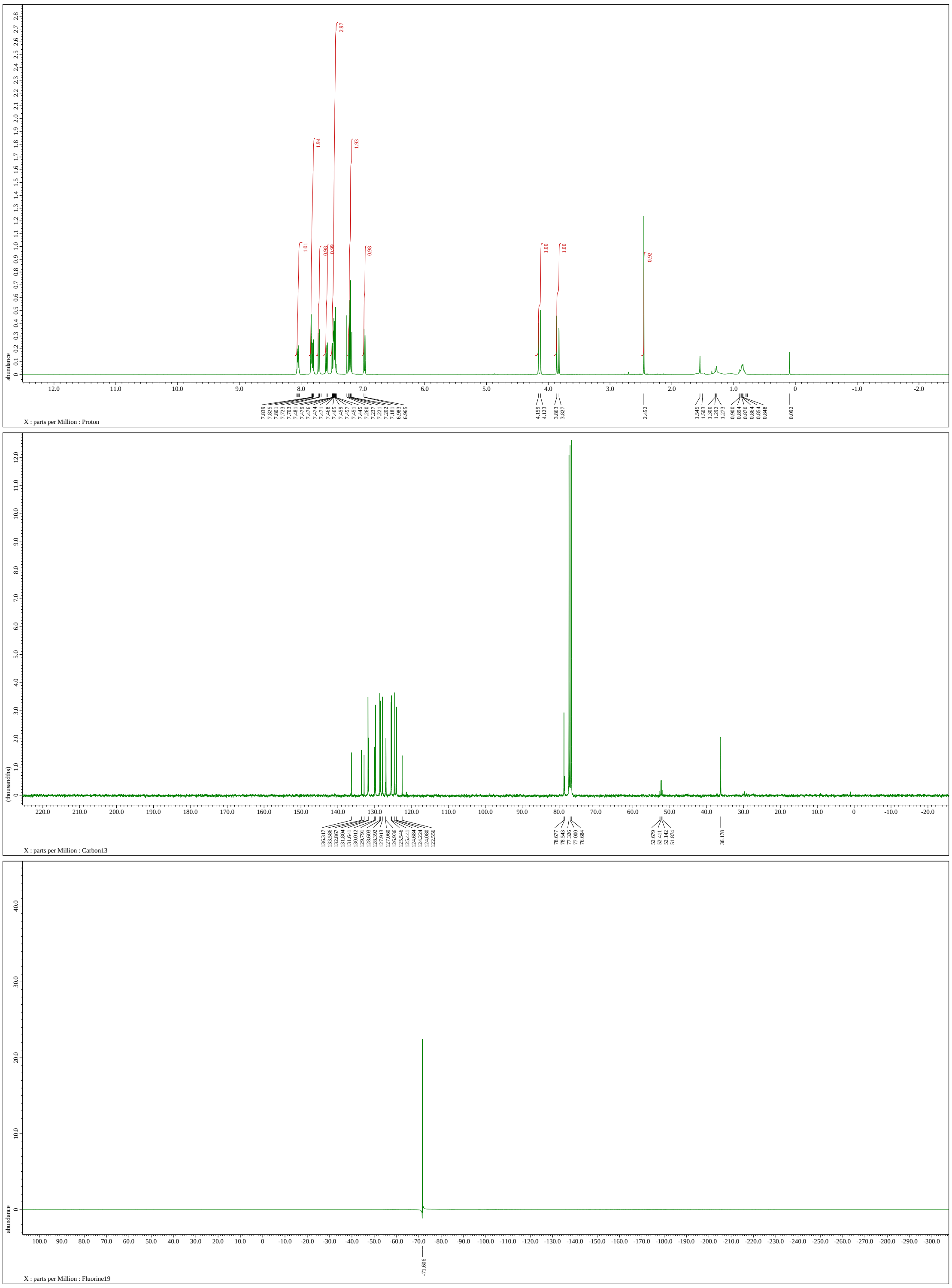

Supplementary Fig. 55: ^{1}H, ^{13}C, and ^{19}F NMR charts of **7lr**.

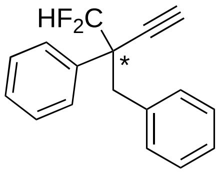

9

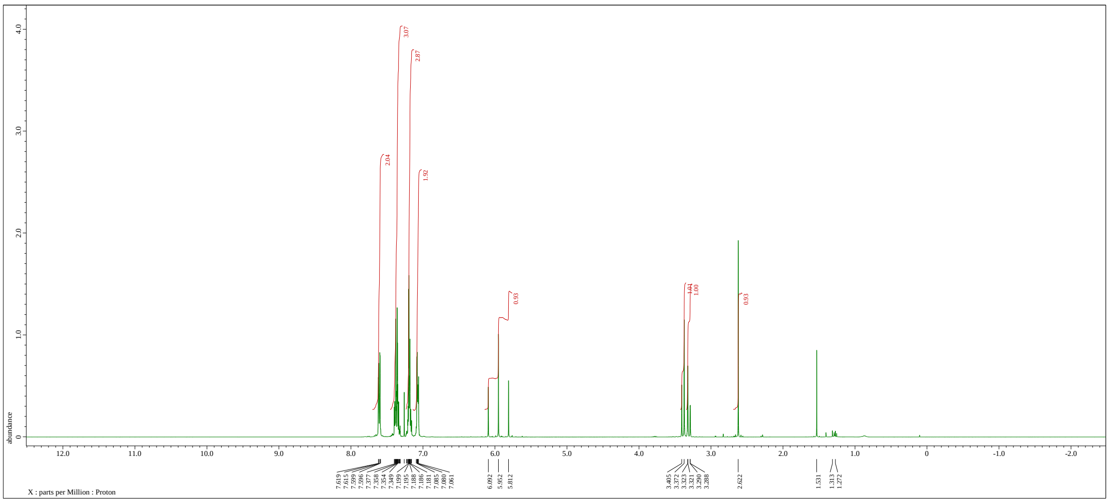

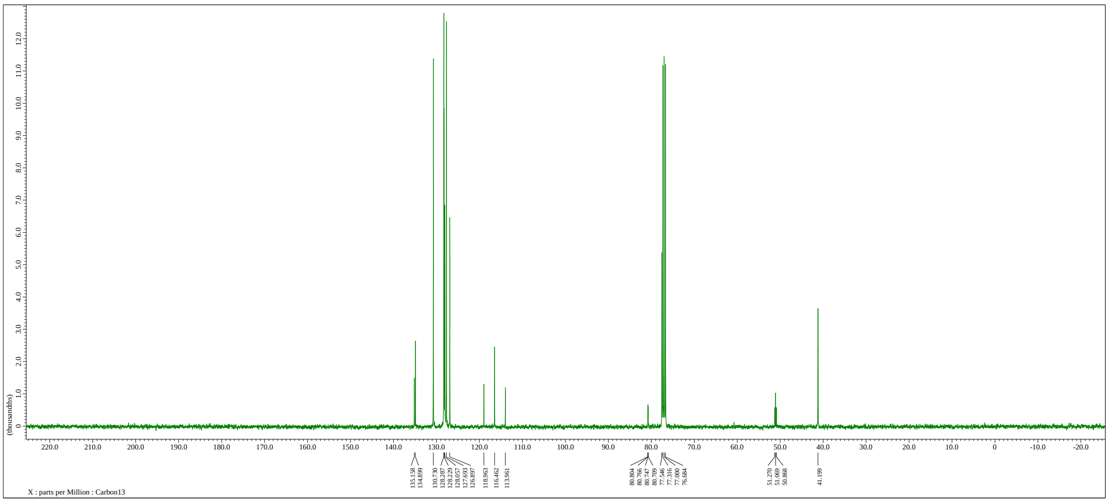

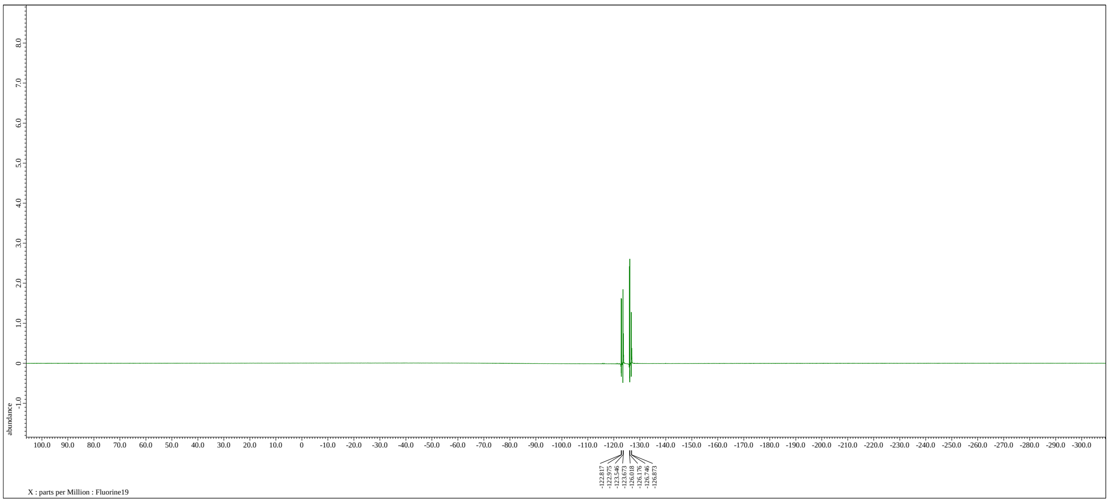

Supplementary Fig. 56: ^{1}H, ^{13}C, and ^{19}F NMR charts of **9**.

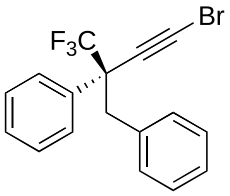

10

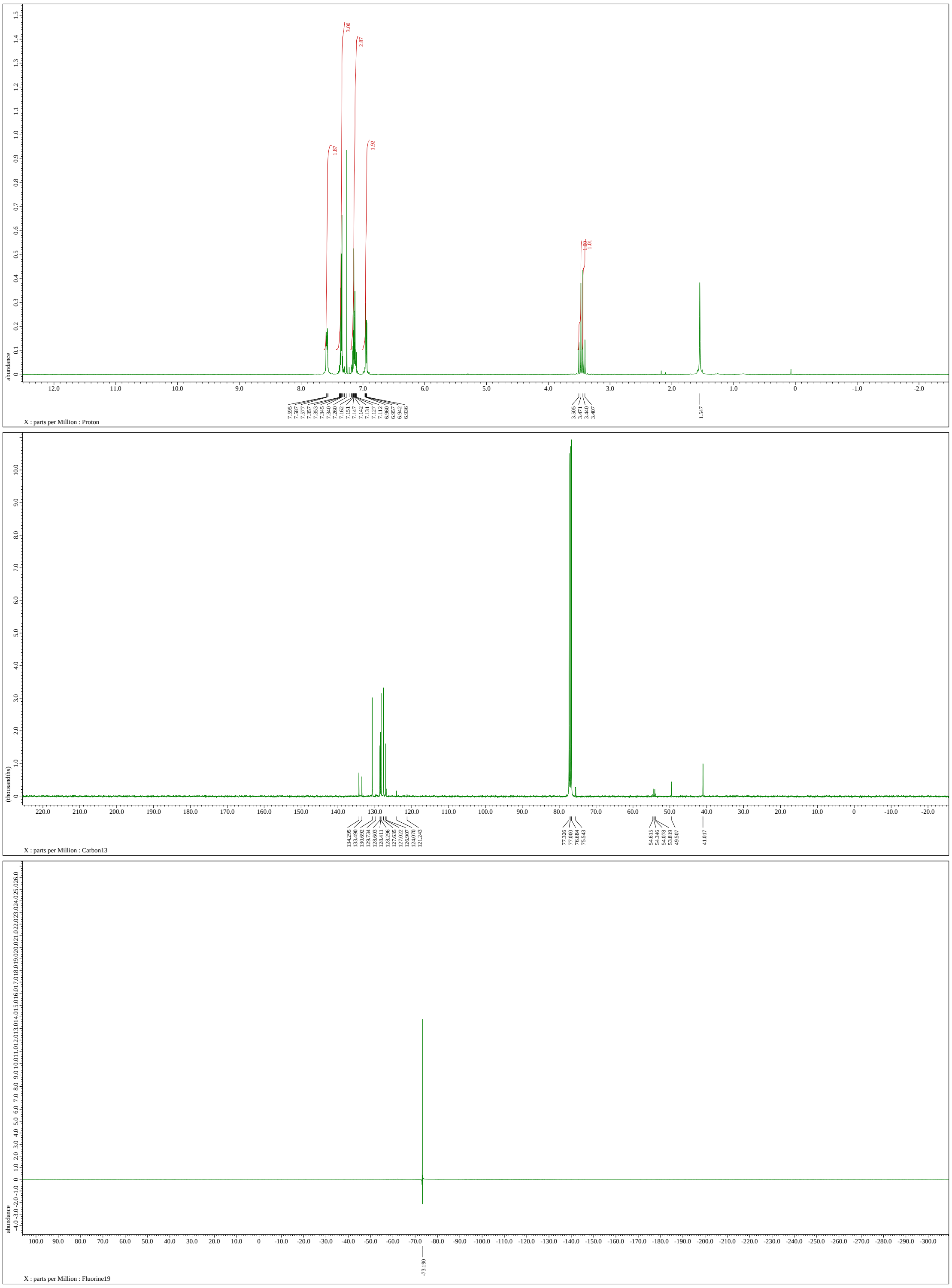

Supplementary Fig. 57: ^{1}H, ^{13}C, and ^{19}F NMR charts of **10**.

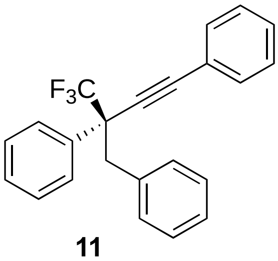

11

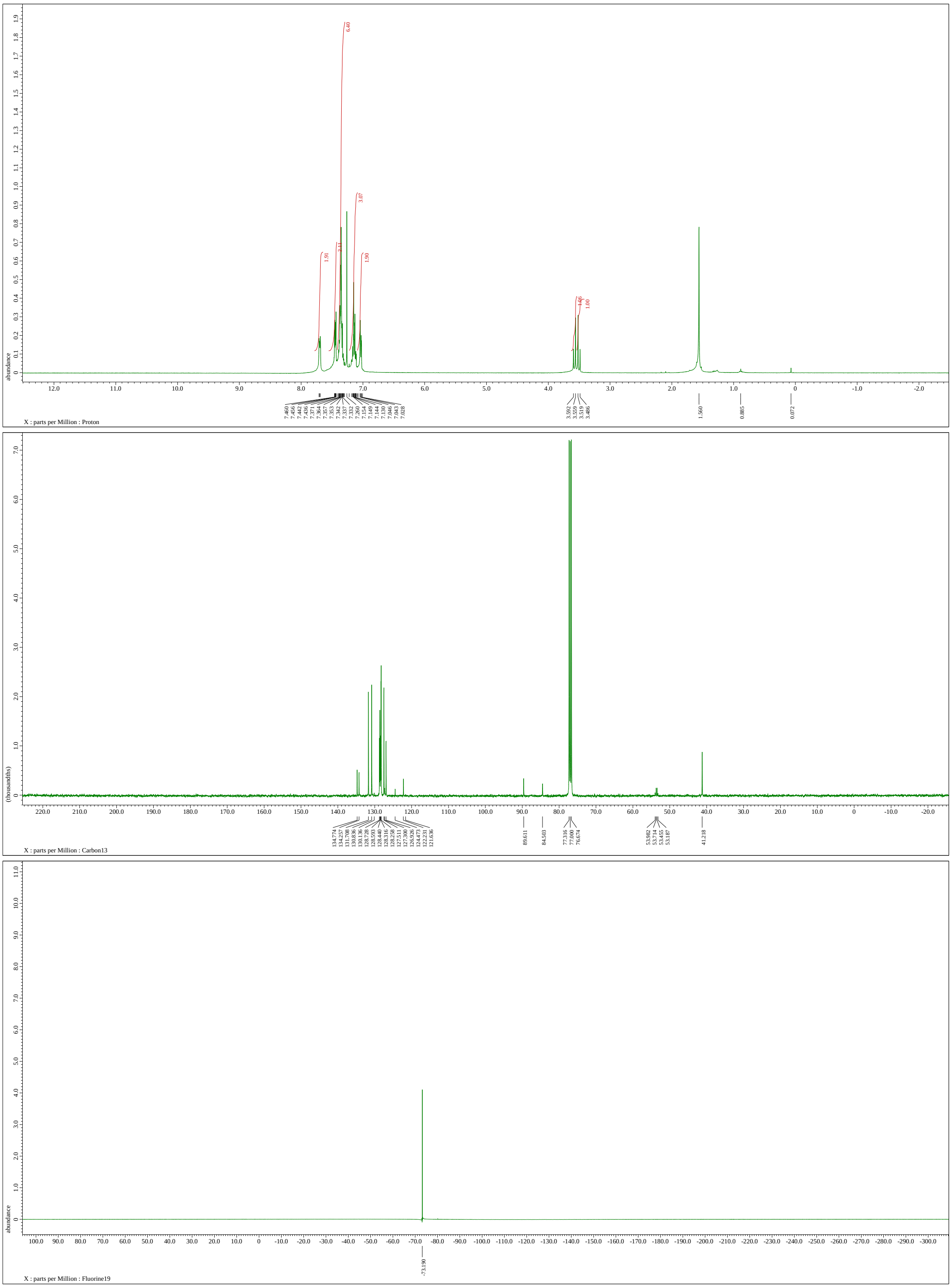

Supplementary Fig. 58: ^{1}H, ^{13}C, and ^{19}F NMR charts of **11**.

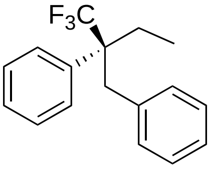

12

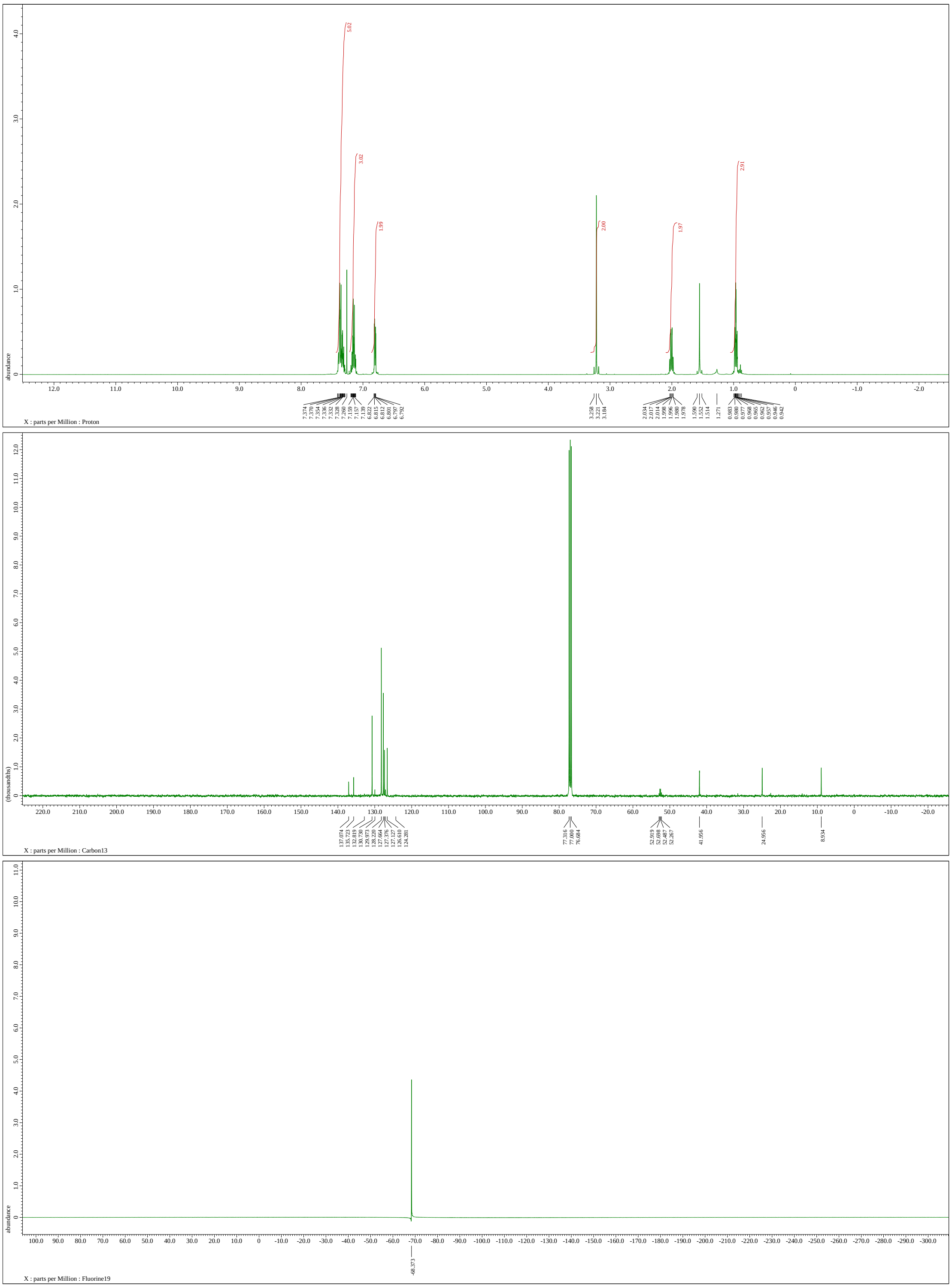

Supplementary Fig. 59: ^{1}H, ^{13}C, and ^{19}F NMR charts of **12**.

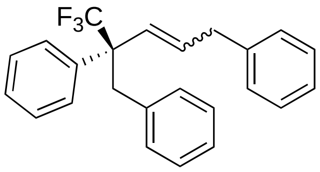

13

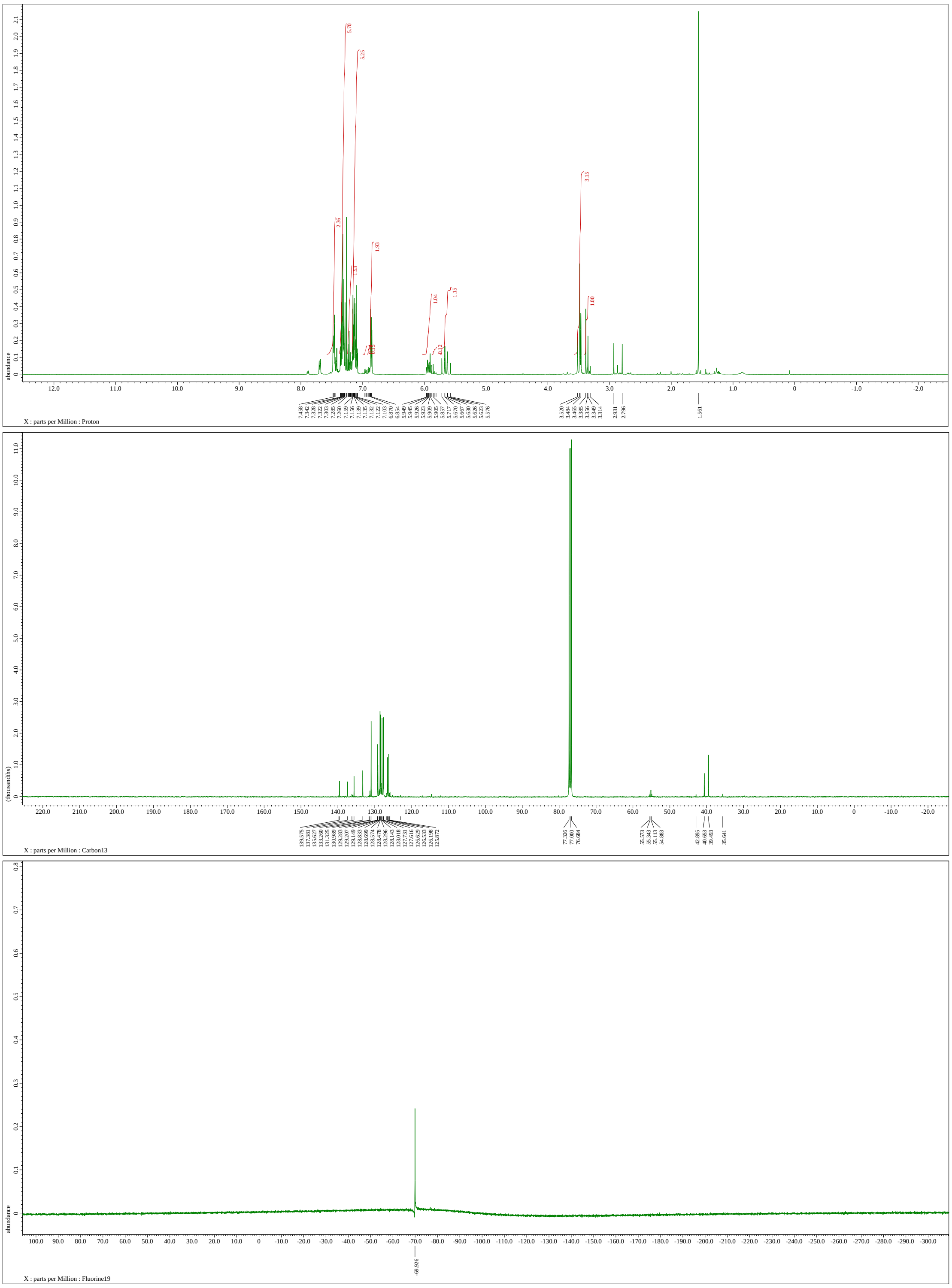

Supplementary Fig. 60: ^{1}H, ^{13}C, and ^{19}F NMR charts of **13**.

1.23: HPLC charts

5 (racemate)

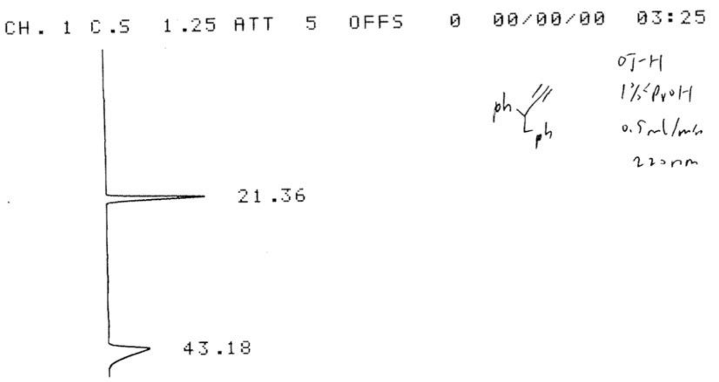

D-2500 00/00/00 03:25

METHOD: TAG: 3 CH: 1

FILE: 0 CALC-METHOD: AREA% TABLE: 0 CONC: AREA

NO.	RT	AREA	CONC	BC
1	21.36	111322	46.050	BB
2	43.18	130422	53.950	BB
TOTAL		241744	100.000	

PEAK REJ : 0

5 (chiral)

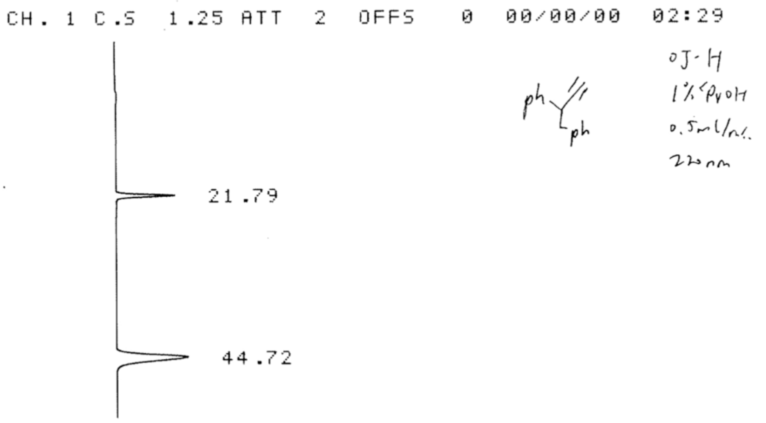

D-2500 00/00/00 02:29

METHOD: TAG: 2 CH: 1

FILE: 0 CALC-METHOD: AREA% TABLE: 0 CONC: AREA

NO.	RT	AREA	CONC	BC
1	21.79	6518	26.234	BB
2	44.72	18328	73.766	BB
TOTAL		24846	100.000	

PEAK REJ : 0

Supplementary Fig. 61: HPLC charts of **5**.

7aa (racemate)

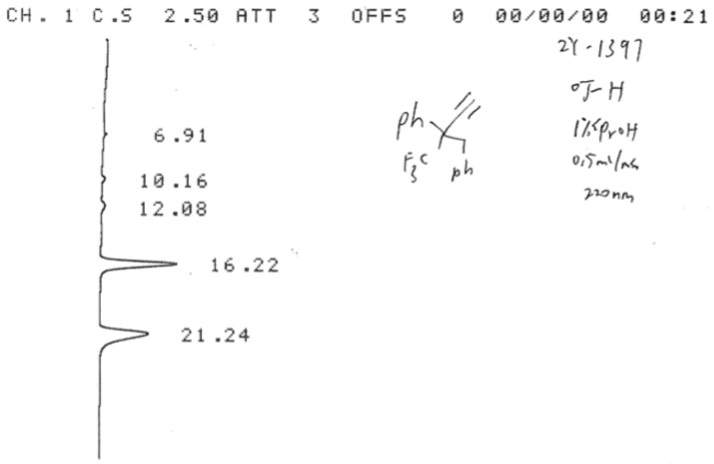

D-2500 00/00/00 00:21

METHOD: TAG: 1 CH: 1

FILE: 0 CALC-METHOD: AREA% TABLE: 0 CONC: AREA

NO.	RT	AREA	CONC	BC
4	16.22	20010	49.260	BB
5	21.24	20611	50.740	BB
TOTAL				
		40621	100.000	

PEAK REJ : 1200

7aa (chiral)

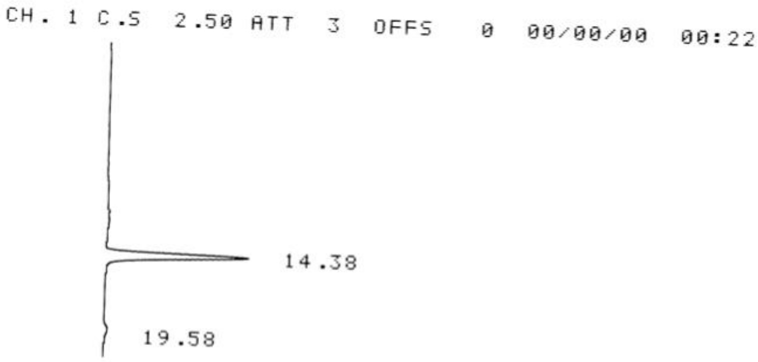

D-2500 00/00/00 00:22

METHOD: TAG: 2 CH: 1

FILE: 0 CALC-METHOD: AREA% TABLE: 0 CONC: AREA

NO.	RT	AREA	CONC	BC
1	14.38	29864	97.002	BB
2	19.58	923	2.998	BB
TOTAL				
		30787	100.000	

PEAK REJ : 0

Supplementary Fig. 62: HPLC charts of **7aa**.

7ab (racemate)

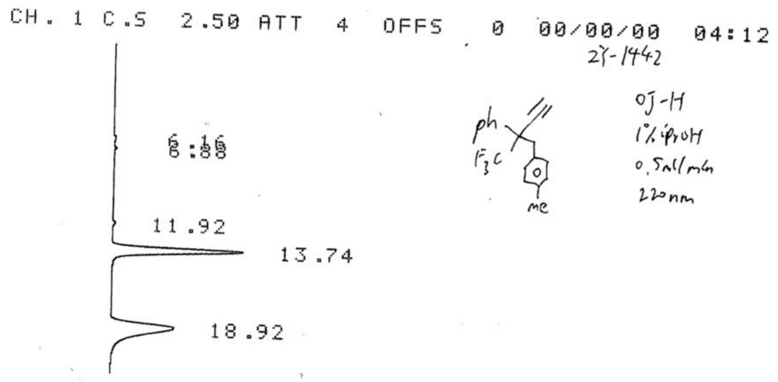

D-2500

00/00/00 04:12

METHOD: TAG: 3 CH: 1

FILE: 0 CALC-METHOD: AREA% TABLE: 0 CONC: AREA

NO.	RT	AREA	CONC	BC
4	13.74	54065	50.596	BB
5	18.92	52792	49.404	BB
TOTAL		106857	100.000	

PEAK REJ : 700

7ab (chiral)

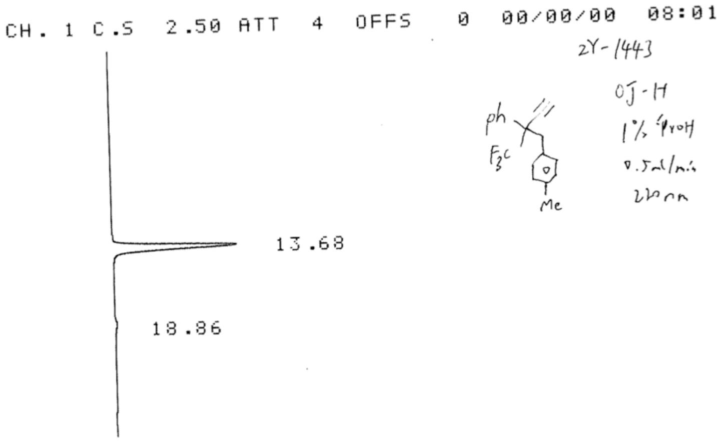

00/00/00 08:01

D-2500

METHOD: TAG: 6 CH: 1

FILE: 0 CALC-METHOD: AREA% TABLE: 0 CONC: AREA

NO.	RT	AREA	CONC	BC
1	13.68	48405	97.242	BB
2	18.86	1373	2.758	BB
TOTAL		49778	100.000	

PEAK REJ : 0

Supplementary Fig. 63: HPLC charts of **7ab**.

7ac (racemate)

CH. 1 C.S 2.50 ATT 4 OFFS 0 00/00/00 01:19

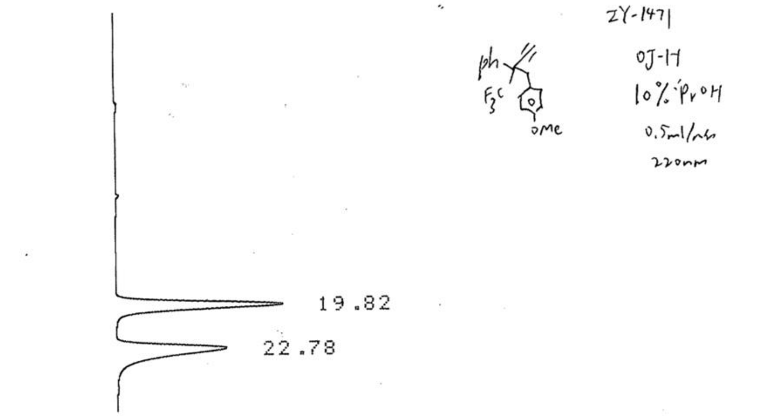

D-2500 00/00/00 01:19

METHOD: TAG: 3 CH: 1

FILE: 0 CALC-METHOD: AREA% TABLE: 0 CONC: AREA

NO.	RT	AREA	CONC	BC
1	19.82	101021	50.795	BB
2	22.78	97857	49.205	BB
TOTAL		198878	100.000	

PEAK REJ : 0

7ac (chiral)

CH. 1 C.S 2.50 ATT 3 OFFS 0 00/00/00 03:10

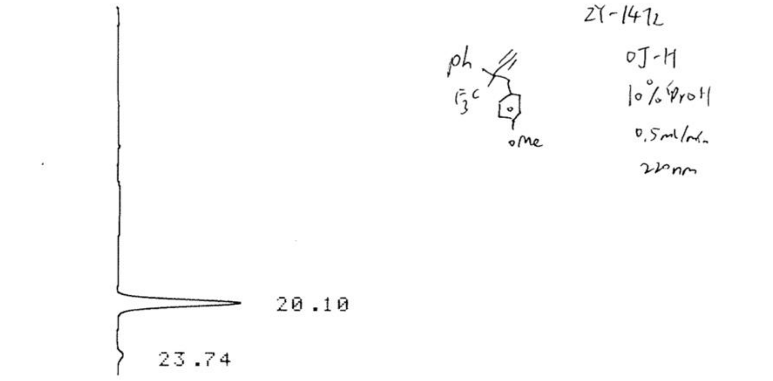

D-2500 00/00/00 03:10

METHOD: TAG: 1 CH: 1

FILE: 0 CALC-METHOD: AREA% TABLE: 0 CONC: AREA

NO.	RT	AREA	CONC	BC
1	20.10	35227	96.136	BB
2	23.74	1416	3.864	BB
TOTAL		36643	100.000	

PEAK REJ : 0

Supplementary Fig. 64: HPLC charts of **7ac**.

7ad (racemate)

CH. 1 C.S 1.25 ATT 3 OFFS 0 00/00/00 04:12

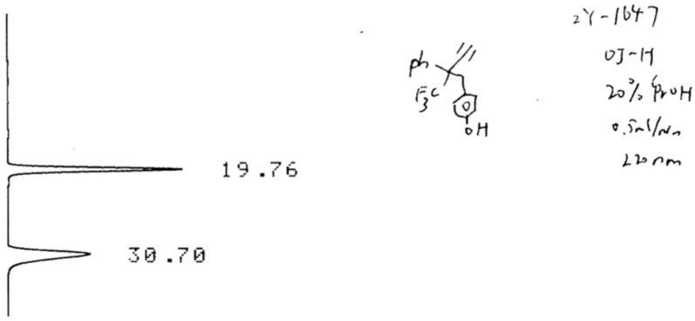

D-2500 00/00/00 04:12

METHOD: TAG: 3 CH: 1

FILE: 0 CALC-METHOD: AREA% TABLE: 0 CONC: AREA

NO.	RT	AREA	CONC	BC
1	19.76	47549	50.141	BB
2	30.70	47281	49.859	BB
TOTAL		94830	100.000	

PEAK REJ : 0

7ad (chiral)

CH. 1 C.S 1.25 ATT 4 OFFS 0 00/00/00 00:01

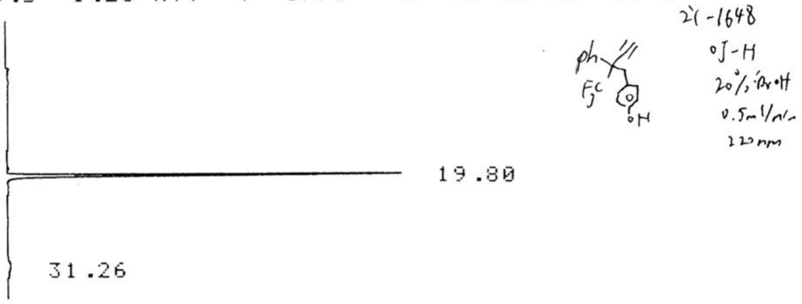

D-2500 00/00/00 00:01

METHOD: TAG: 1 CH: 1

FILE: 0 CALC-METHOD: AREA% TABLE: 0 CONC: AREA

NO.	RT	AREA	CONC	BC
1	19.80	71668	95.999	BB
2	31.26	2987	4.001	BB
TOTAL		74655	100.000	

PEAK REJ : 0

Supplementary Fig. 65: HPLC charts of **7ad**.

7ae (racemate)

CH. 1 C.S 1.25 ATT 3 OFFS 0 00/00/00 00:56

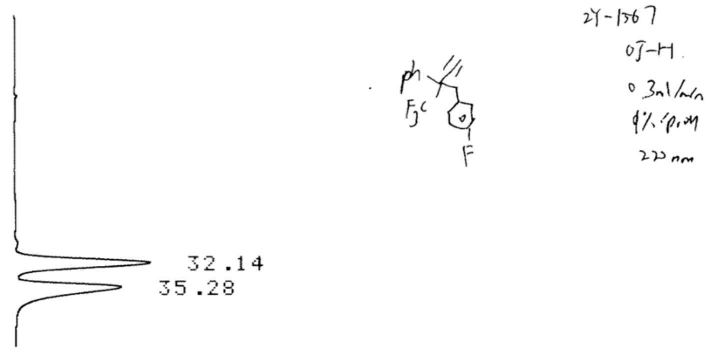

D-2500 00/00/00 00:56

METHOD: TAG: 2 CH: 1

FILE: 0 CALC-METHOD: AREA% TABLE: 0 CONC: AREA

NO.	RT	AREA	CONC	BC
1	32.14	82872	50.214	BB
2	35.28	82166	49.786	BB
TOTAL				
		165038	100.000	

PEAK REJ : 0

7ae (chiral)

CH. 1 C.S 1.25 ATT 2 OFFS 0 00/00/00 00:42

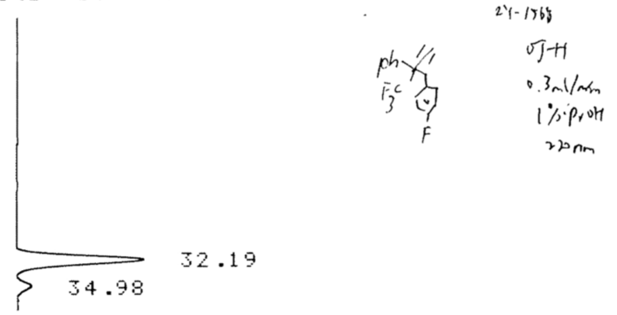

D-2500 00/00/00 00:42

METHOD: TAG: 2 CH: 1

FILE: 0 CALC-METHOD: AREA% TABLE: 0 CONC: AREA

NO.	RT	AREA	CONC	BC
1	32.19	36704	95.833	BB
2	34.98	1596	4.167	BB
TOTAL				
		38300	100.000	

PEAK REJ : 0

Supplementary Fig. 66: HPLC charts of **7ae**.

7af (racemate)

CH. 1 C.S 2.50 ATT 3 OFFS 0 00/00/00 00:00

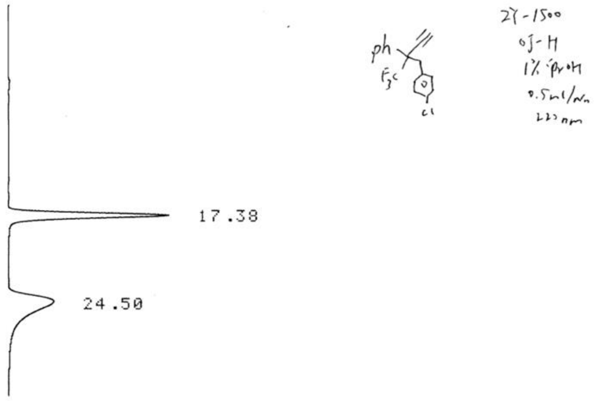

D-2500 00/00/00 00:00

METHOD: TAG: 1 CH: 1

FILE: 0 CALC-METHOD: AREA% TABLE: 0 CONC: AREA

NO.	RT	AREA	CONC	BC
1	17.38	45384	51.277	BB
2	24.50	43123	48.723	BB
TOTAL		88507	100.000	

PEAK REJ : 0

7af (chiral)

CH. 1 C.S 2.50 ATT 2 OFFS 0 00/00/00 01:28

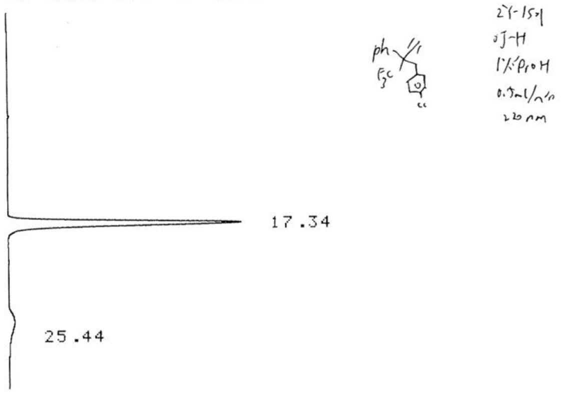

D-2500 00/00/00 01:28

METHOD: TAG: 2 CH: 1

FILE: 0 CALC-METHOD: AREA% TABLE: 0 CONC: AREA

NO.	RT	AREA	CONC	BC
1	17.34	35915	95.094	BB
2	25.44	1853	4.906	BB
TOTAL		37768	100.000	

PEAK REJ : 0

Supplementary Fig. 67: HPLC charts of **7af**.

7ag (racemate)

CH. 1 C.S 2.50 ATT 6 OFFS 0 00/00/00 01:57

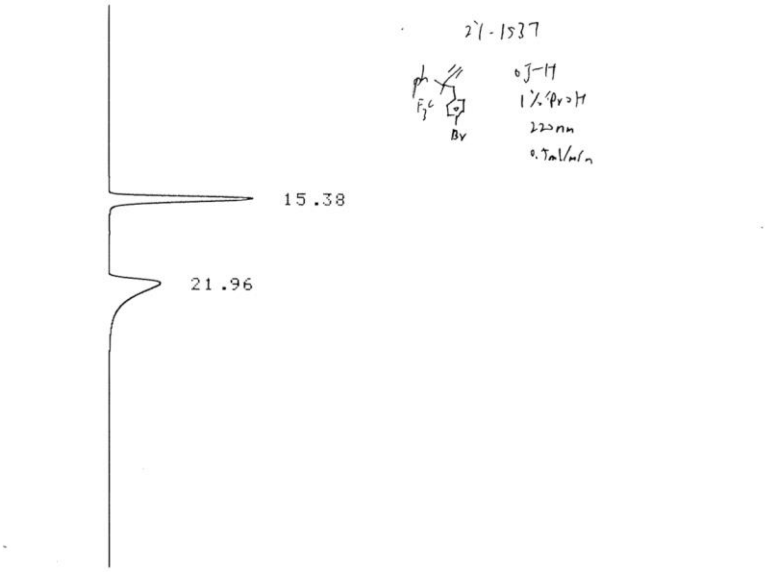

D-2500 00/00/00 01:57

METHOD: TAG: 2 CH: 1

FILE: 0 CALC-METHOD: AREA% TABLE: 0 CONC: AREA

NO.	RT	AREA	CONC	BC
1	15.38	343806	47.005	BB
2	21.96	387618	52.995	BB
TOTAL				
		731424	100.000	

PEAK REJ : 0

7ag (chiral)

CH. 1 C.S 2.50 ATT 4 OFFS 0 00/00/00 03:47

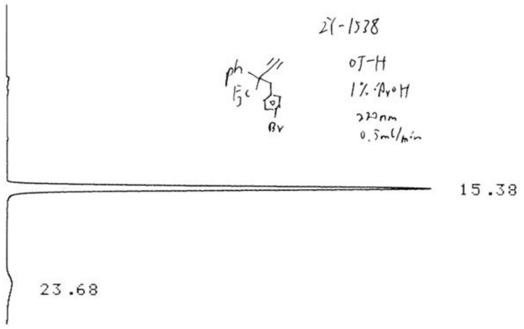

D-2500 00/00/00 03:47

METHOD: TAG: 4 CH: 1

FILE: 0 CALC-METHOD: AREA% TABLE: 0 CONC: AREA

NO.	RT	AREA	CONC	BC
1	15.38	227544	97.027	BB
2	23.68	6972	2.973	BB
TOTAL				
		234516	100.000	

PEAK REJ : 0

Supplementary Fig. 68: HPLC charts of **7ag**.

7ah (racemate)

CH. 1 C.S 2.50 ATT 4 OFFS 0 00/00/00 00:09

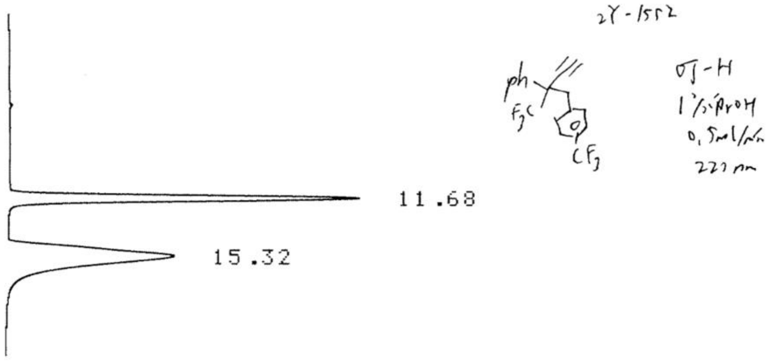

D-2500 00/00/00 00:09

METHOD: TAG: 1 CH: 1

FILE: 0 CALC-METHOD: AREA% TABLE: 0 CONC: AREA

NO.	RT	AREA	CONC	BC
1	11.68	144051	53.995	BB
2	15.32	122736	46.005	BB
TOTAL				
		266787	100.000	
PEAK REJ :		0		

7ah (chiral)

CH. 1 C.S 2.50 ATT 4 OFFS 0 00/00/00 00:34

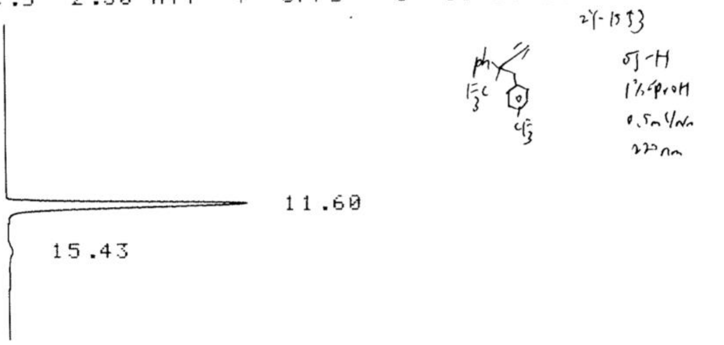

D-2500 00/00/00 00:34

METHOD: TAG: 1 CH: 1

FILE: 0 CALC-METHOD: AREA% TABLE: 0 CONC: AREA

NO.	RT	AREA	CONC	BC
1	11.60	91516	95.737	BB
2	15.43	4075	4.263	BB
TOTAL				
		95591	100.000	
PEAK REJ :		0		

Supplementary Fig. 69: HPLC charts of **7ah**.

7ai (racemate)

CH. 1 C.S 1.25 ATT 4 OFFS 0 00/00/00 00:44

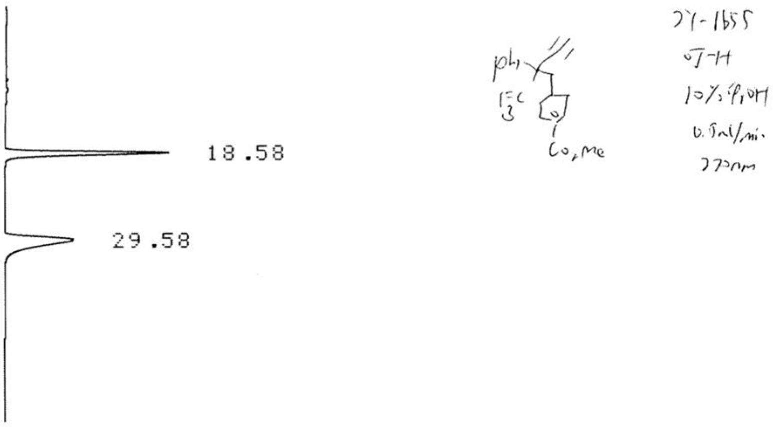

D-2500 00/00/00 00:44

METHOD: TAG: 2 CH: 1

FILE: 0 CALC-METHOD: AREA% TABLE: 0 CONC: AREA

NO.	RT	AREA	CONC	BC
1	18.58	87848	50.612	BB
2	29.58	85724	49.388	BB
TOTAL				
		173572	100.000	

PEAK REJ : 0

7ai (chiral)

CH. 1 C.S 1.25 ATT 4 OFFS 0 00/00/00 03:09

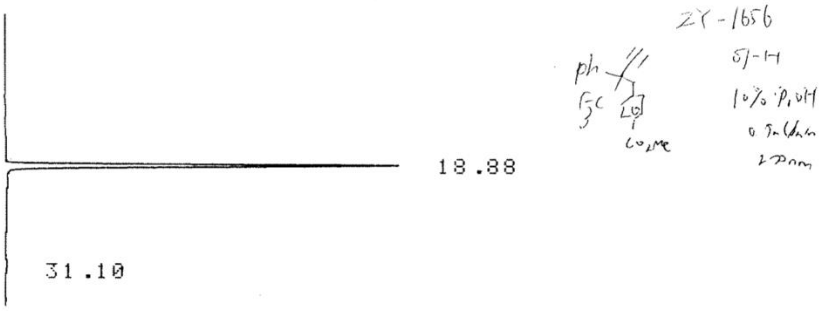

D-2500 00/00/00 03:09

METHOD: TAG: 3 CH: 1

FILE: 0 CALC-METHOD: AREA% TABLE: 0 CONC: AREA

NO.	RT	AREA	CONC	BC
1	18.88	133304	97.798	BB
2	31.10	3002	2.202	BB
TOTAL				
		136306	100.000	

PEAK REJ : 0

Supplementary Fig. 70: HPLC charts of **7ai**.

7aj (racemate)

CH. 1 C.S 2.50 ATT 4 OFFS 0 00/00/00 00:58

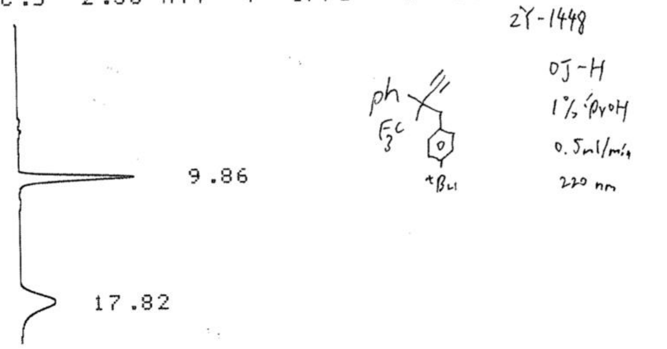

D-2500 00/00/00 00:58

METHOD: TAG: 2 CH: 1

FILE: 0 CALC-METHOD: AREA% TABLE: 0 CONC: AREA

NO.	RT	AREA	CONC	BC
1	9.86	42994	52.497	BB
2	17.82	38904	47.503	BB
TOTAL		81898	100.000	

PEAK REJ : 0

7aj (chiral)

CH. 1 C.S 2.50 ATT 3 OFFS 0 00/00/00 00:28

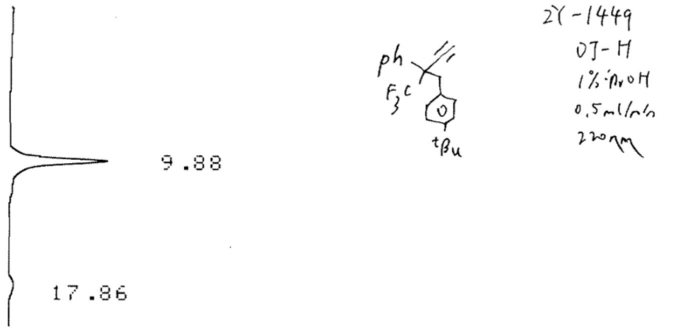

D-2500 00/00/00 00:28

METHOD: TAG: 1 CH: 1

FILE: 0 CALC-METHOD: AREA% TABLE: 0 CONC: AREA

NO.	RT	AREA	CONC	BC
1	9.88	29071	91.802	BB
2	17.86	2596	8.198	BB
TOTAL		31667	100.000	

PEAK REJ : 0

Supplementary Fig. 71: HPLC charts of **7aj**.

7ak (racemate)

CH. 1 C.S 5.00 ATT 5 OFFS 0 00/00/00 01:55

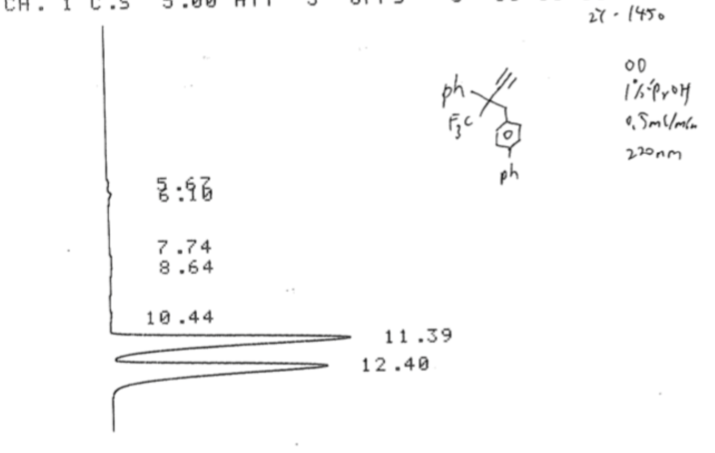

D-2500 00/00/00 01:55

METHOD: TAG: 3 CH: 1

FILE: 0 CALC-METHOD: AREA% TABLE: 0 CONC: AREA

NO.	RT	AREA	CONC	BC
6	11.39	205520	48.976	BV
7	12.40	214118	51.024	VB
TOTAL		419638	100.000	

PEAK REJ : 3500

7ak (chiral)

CH. 1 C.S 5.00 ATT 3 OFFS 0 00/00/00 02:23

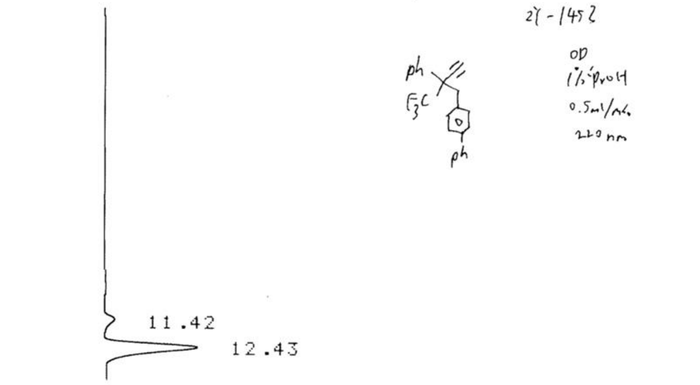

D-2500 00/00/00 02:23

METHOD: TAG: 2 CH: 1

FILE: 0 CALC-METHOD: AREA% TABLE: 0 CONC: AREA

NO.	RT	AREA	CONC	BC
1	11.42	1818	8.681	BB
2	12.43	19125	91.319	BB
TOTAL		20943	100.000	

PEAK REJ : 0

Supplementary Fig. 72: HPLC charts of **7ak**.

7al (racemate)

CH. 1 C.S 2.50 ATT 4 OFFS 0 00/00/00 07:31

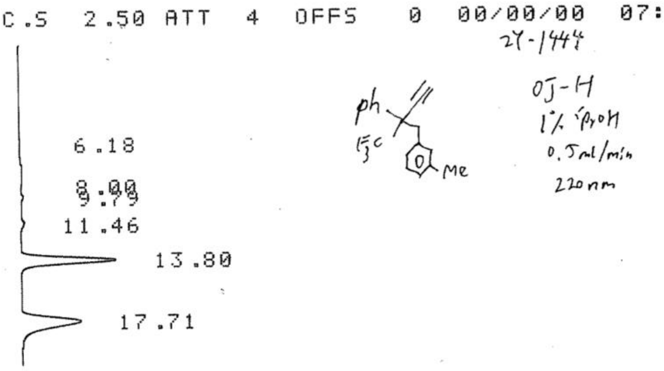

D-2500 00/00/00 07:31

METHOD: TAG: 5 CH: 1

FILE: 0 CALC-METHOD: AREA% TABLE: 0 CONC: AREA

NO.	RT	AREA	CONC	BC
5	13.80	41647	49.819	BB
6	17.71	41950	50.181	BB
TOTAL				
		83597	100.000	

PEAK REJ : 1600

7al (chiral)

CH. 1 C.S 2.50 ATT 3 OFFS 0 00/00/00 00:35

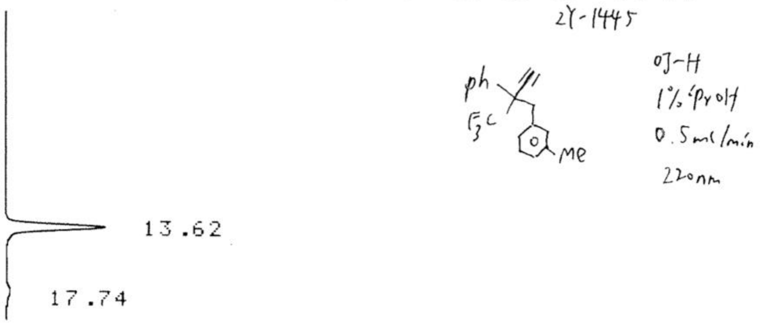

D-2500 00/00/00 00:35

METHOD: TAG: 2 CH: 1

FILE: 0 CALC-METHOD: AREA% TABLE: 0 CONC: AREA

NO.	RT	AREA	CONC	BC
1	13.62	20157	95.080	BB
2	17.74	1043	4.920	BB
TOTAL				
		21200	100.000	

PEAK REJ : 0

Supplementary Fig. 73: HPLC charts of **7al**.

7am (racemate)

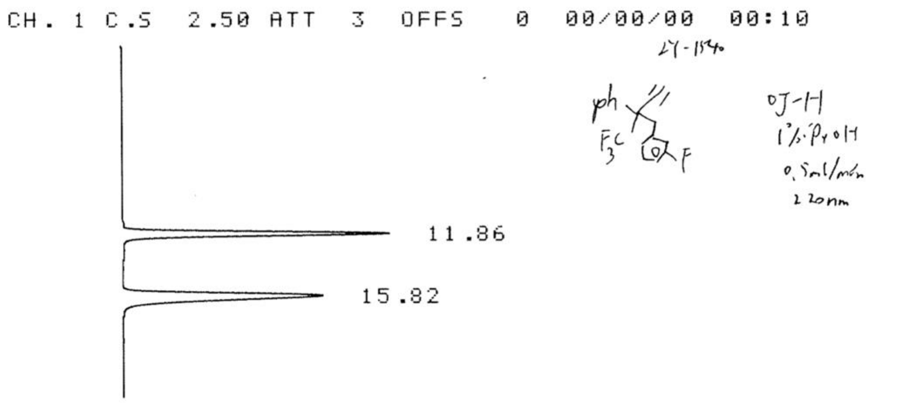

D-2500 00/00/00 00:10

METHOD: TAG: 1 CH: 1

FILE: 0 CALC-METHOD: AREA% TABLE: 0 CONC: AREA

NO.	RT	AREA	CONC	BC
1	11.86	40194	45.931	BB
2	15.82	47316	54.069	BB
TOTAL				
		87510	100.000	

PEAK REJ : 0

7am (chiral)

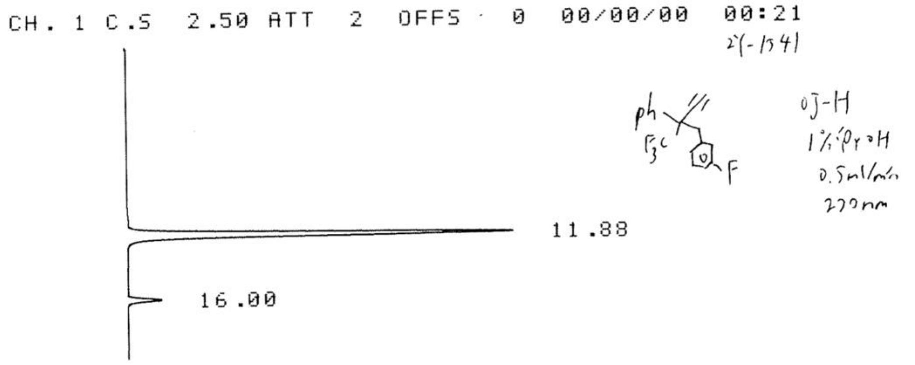

D-2500 00/00/00 00:21

METHOD: TAG: 2 CH: 1

FILE: 0 CALC-METHOD: AREA% TABLE: 0 CONC: AREA

NO.	RT	AREA	CONC	BC
1	11.88	26913	94.931	BB
2	16.00	1437	5.069	BB
TOTAL				
		28350	100.000	

PEAK REJ : 0

Supplementary Fig. 74: HPLC charts of **7am**.

7an (racemate)

CH. 1 C.S 1.25 ATT 3 OFFS 0 00/00/00 01:45

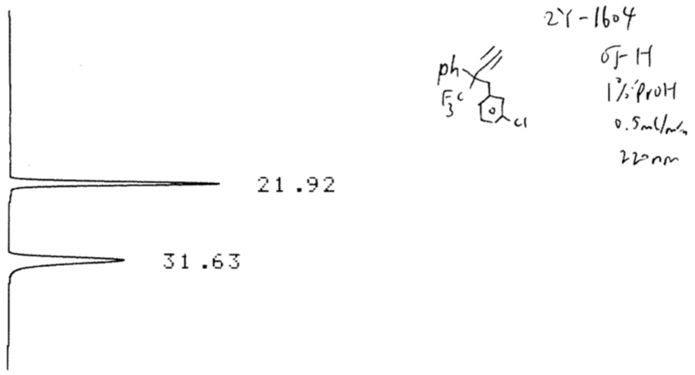

D-2500 00/00/00 01:45

METHOD: TAG: 2 CH: 1

FILE: 0 CALC-METHOD: AREA% TABLE: 0 CONC: AREA

NO.	RT	AREA	CONC	BC
1	21.92	54946	49.620	BB
2	31.63	55788	50.380	BB
TOTAL				
		110734	100.000	

PEAK REJ : 0

7an (chiral)

CH. 1 C.S 1.25 ATT 2 OFFS 0 00/00/00 00:04

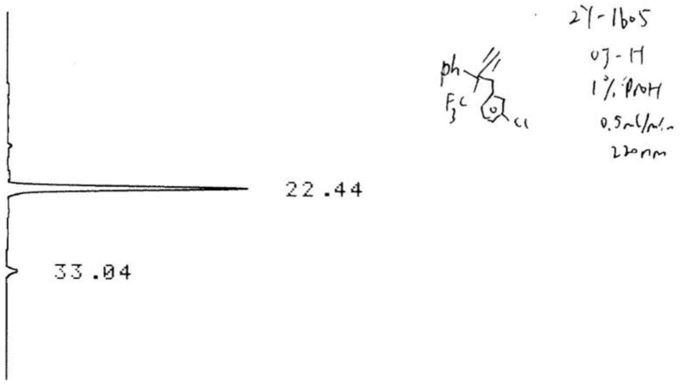

D-2500 00/00/00 00:04

METHOD: TAG: 1 CH: 1

FILE: 0 CALC-METHOD: AREA% TABLE: 0 CONC: AREA

NO.	RT	AREA	CONC	BC
1	22.44	31052	95.642	BB
2	33.04	1415	4.358	BB
TOTAL				
		32467	100.000	

PEAK REJ : 0

Supplementary Fig. 75: HPLC charts of **7an**.

7ao (racemate)

CH. 1 C.S 1.25 ATT 3 OFFS 0 00/00/00 02:55

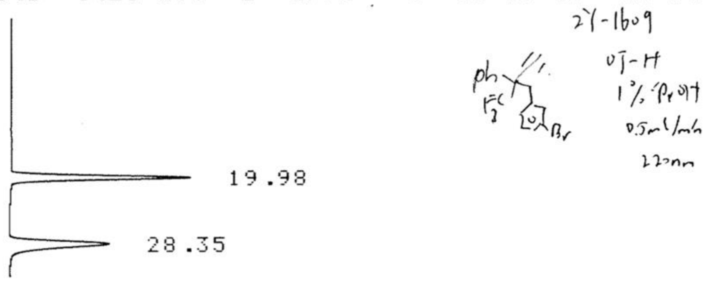

D-2500 00/00/00 02:55

METHOD: TAG: 2 CH: 1

FILE: 0 CALC-METHOD: AREA% TABLE: 0 CONC: AREA

NO.	RT	AREA	CONC	BC
1	19.98	48335	51.028	BB
2	28.35	46387	48.972	BB
TOTAL		94722	100.000	

PEAK REJ : 0

7ao (chiral)

CH. 1 C.S 1.25 ATT 2 OFFS 0 00/00/00 01:47

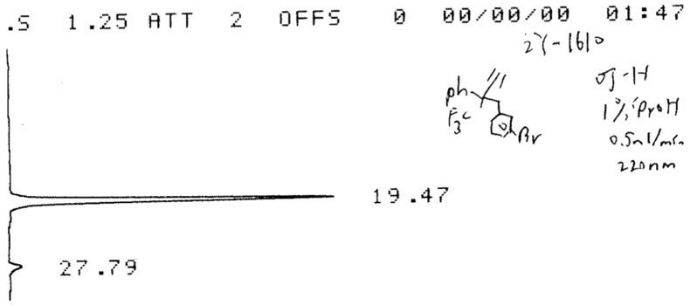

D-2500 00/00/00 01:47

METHOD: TAG: 4 CH: 1

FILE: 0 CALC-METHOD: AREA% TABLE: 0 CONC: AREA

NO.	RT	AREA	CONC	BC
1	19.47	41257	96.682	BB
2	27.79	1416	3.318	BB
TOTAL		42673	100.000	

PEAK REJ : 0

Supplementary Fig. 76: HPLC charts of **7ao**.

7ap (racemate)

CH. 1 C.S 2.50 ATT 4 OFFS 0 00/00/00 00:37

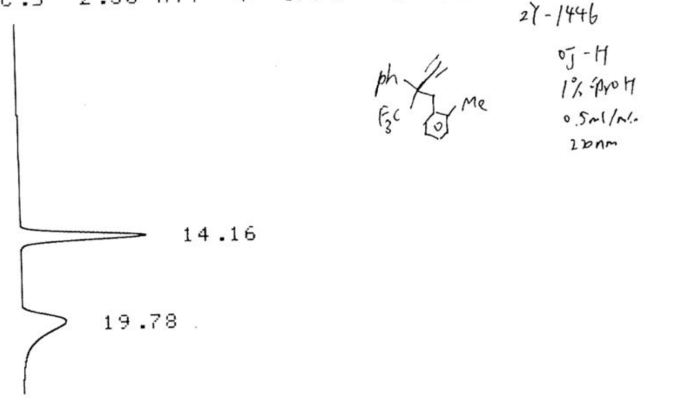

D-2500 00/00/00 00:37

METHOD: TAG: 2 CH: 1

FILE: 0 CALC-METHOD: AREA% TABLE: 0 CONC: AREA

NO.	RT	AREA	CONC	BC
1	14.16	67756	52.148	BB
2	19.78	62173	47.852	BB
TOTAL		129929	100.000	

PEAK REJ : 0

7ap (chiral)

CH. 1 C.S 2.50 ATT 3 OFFS 0 00/00/00 00:00

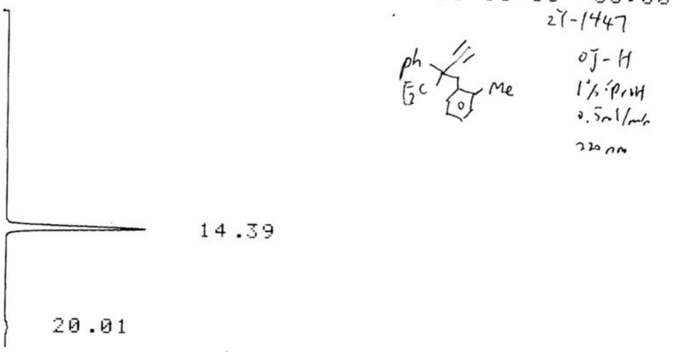

D-2500 00/00/00 00:00

METHOD: TAG: 1 CH: 1

FILE: 0 CALC-METHOD: AREA% TABLE: 0 CONC: AREA

NO.	RT	AREA	CONC	BC
1	14.39	20469	95.085	BB
2	20.01	1058	4.915	BB
TOTAL		21527	100.000	

PEAK REJ : 0

Supplementary Fig. 77: HPLC charts of **7ap**.

7aq (racemate)

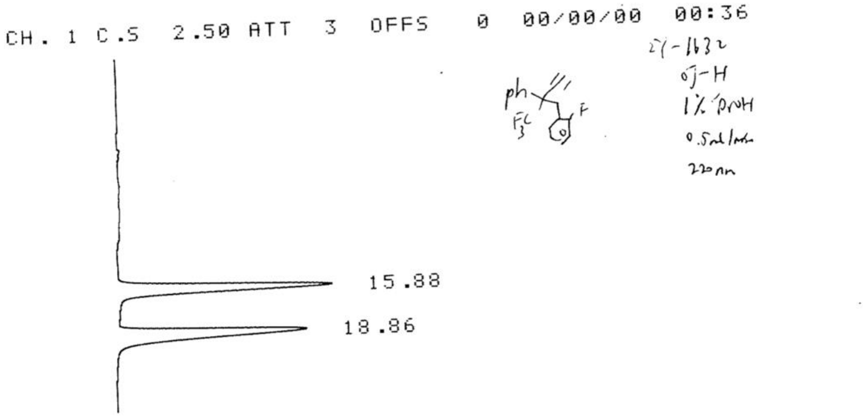

D-2500 00/00/00 00:36

METHOD: TAG: 2 CH: 1

FILE: 0 CALC-METHOD: AREA% TABLE: 0 CONC: AREA

NO.	RT	AREA	CONC	BC
1	15.88	59573	48.618	BB
2	18.86	62961	51.382	BB
TOTAL		122534	100.000	

PEAK REJ : 0

7aq (chiral)

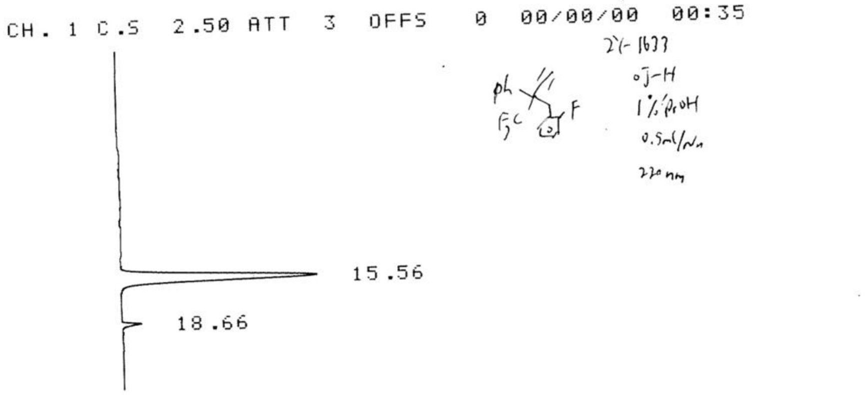

D-2500 00/00/00 00:35

METHOD: TAG: 2 CH: 1

FILE: 0 CALC-METHOD: AREA% TABLE: 0 CONC: AREA

NO.	RT	AREA	CONC	BC
1	15.56	51528	96.512	BB
2	18.66	1862	3.488	BB
TOTAL		53390	100.000	

PEAK REJ : 0

Supplementary Fig. 78: HPLC charts of **7aq**.

7ar (racemate)

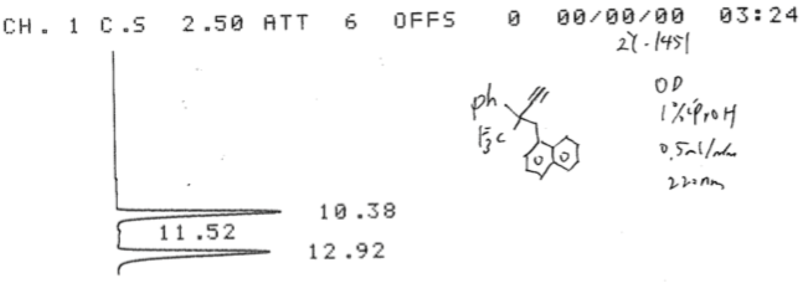

D-2500 00/00/00 03:24

METHOD: TAG: 5 CH: 1

FILE: 0 CALC-METHOD: AREA% TABLE: 0 CONC: AREA

NO.	RT	AREA	CONC	BC
1	10.38	220533	47.754	BB
3	12.92	241279	52.246	BB
TOTAL		461812	100.000	

PEAK REJ : 3500

7ar (chiral)

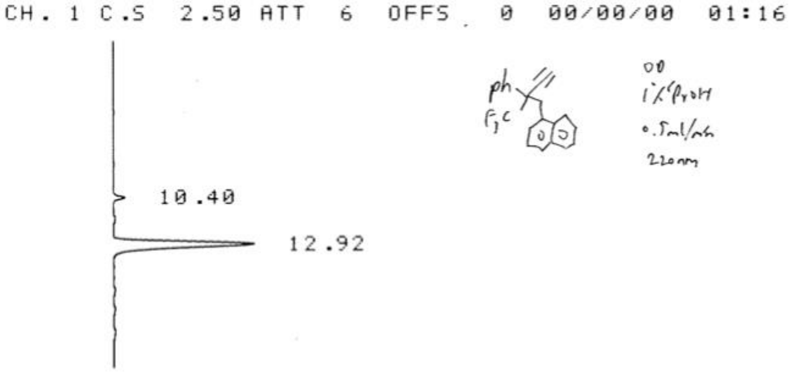

D-2500 00/00/00 01:16

METHOD: TAG: 2 CH: 1

FILE: 0 CALC-METHOD: AREA% TABLE: 0 CONC: AREA

NO.	RT	AREA	CONC	BC
1	10.40	12167	4.633	BB
2	12.92	250443	95.367	BB
TOTAL		262610	100.000	

PEAK REJ : 0

Supplementary Fig. 79: HPLC charts of **7ar**.

7as (racemate)

CH. 1 C.S 1.25 ATT 4 OFFS 0 00/00/00 02:24

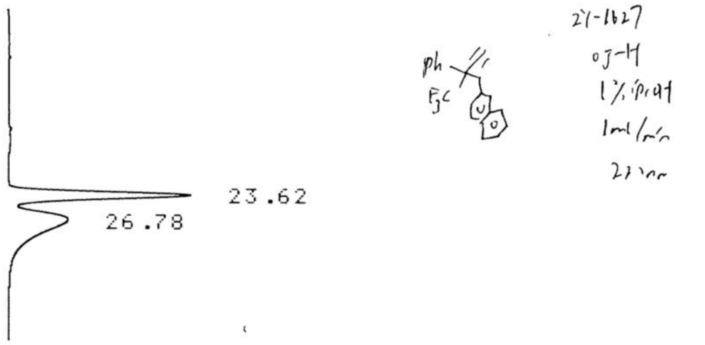

D-2500 00/00/00 02:24

METHOD: TAG: 3 CH: 1

FILE: 0 CALC-METHOD: AREA% TABLE: 0 CONC: AREA

NO.	RT	AREA	CONC	BC
1	23.62	183168	52.127	BV
2	26.78	168223	47.873	VB
TOTAL				
		351391	100.000	

PEAK REJ : 0

7as (chiral)

CH. 1 C.S 1.25 ATT 6 OFFS 0 00/00/00 01:06

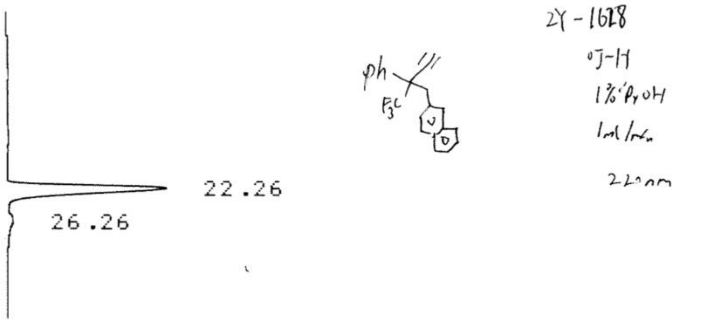

D-2500 00/00/00 01:06

METHOD: TAG: 2 CH: 1

FILE: 0 CALC-METHOD: AREA% TABLE: 0 CONC: AREA

NO.	RT	AREA	CONC	BC
1	22.26	670761	97.442	BB
2	26.26	17608	2.558	BB
TOTAL				
		688369	100.000	

PEAK REJ : 0

Supplementary Fig. 80: HPLC charts of **7as**.

7at (racemate)

CH. 1 C.S 1.25 ATT 2 OFFS 0 00/00/00 03:15

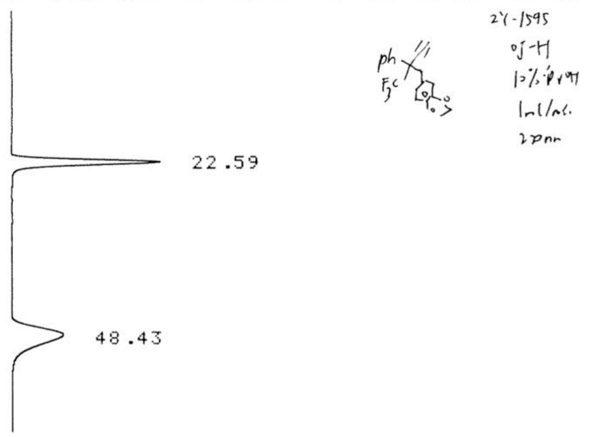

D-2500 00/00/00 03:15

METHOD: TAG: 4 CH: 1

FILE: 0 CALC-METHOD: AREA% TABLE: 0 CONC: AREA

NO.	RT	AREA	CONC	BC
1	22.59	37910	50.827	BB
2	48.43	36676	49.173	BB
TOTAL		74586	100.000	

PEAK REJ : 0

7at (chiral)

CH. 1 C.S 1.25 ATT 2 OFFS 0 00/00/00 00:01

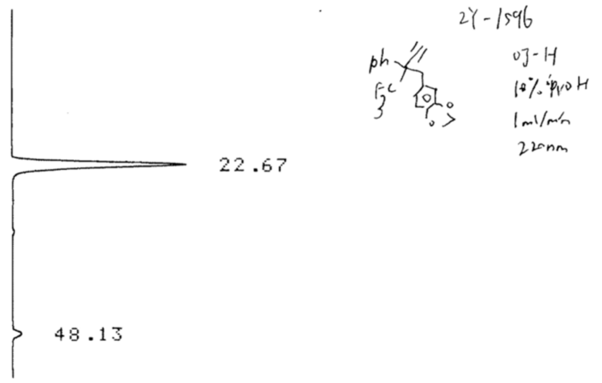

D-2500 00/00/00 00:01

METHOD: TAG: 1 CH 1

FILE: 0 CALC-M 'HOD: AREA% TABLE 0 CONC: AREA

NO.	RT	AREA	CONC	BC
1	22.67	34983	95.329	BB
2	48.13	1714	4.671	BB
TOTAL		36697	100.000	

PEAK REJ : 0

Supplementary Fig. 81: HPLC charts of **7at**.

7au (racemate)

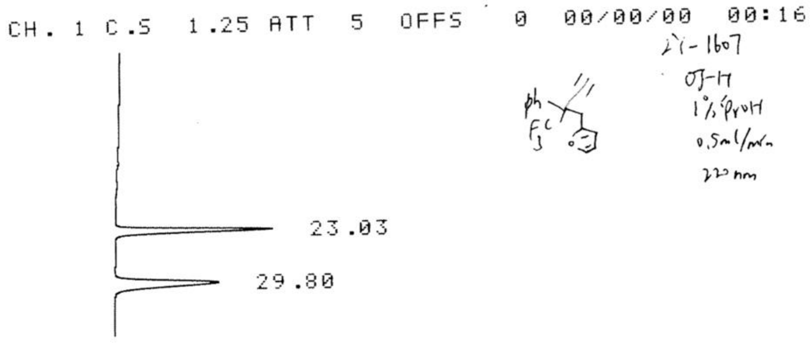

D-2500 00/00/00 00:16

METHOD: TAG: 1 CH: 1

FILE: 0 CALC-METHOD: AREA% TABLE: 0 CONC: AREA

NO.	RT	AREA	CONC	BC
1	23.03	183632	49.752	BB
2	29.80	185463	50.248	BB
TOTAL				
		369095	100.000	

PEAK REJ : 0

7au (chiral)

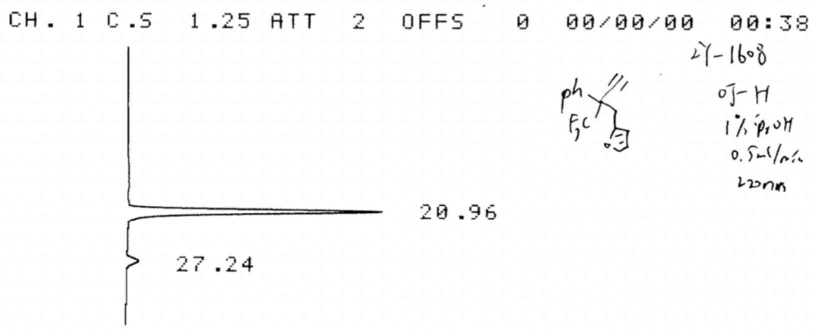

D-2500 00/00/00 00:38

METHOD: TAG: 2 CH: 1

FILE: 0 CALC-METHOD: AREA% TABLE: 0 CONC: AREA

NO.	RT	AREA	CONC	BC
1	20.96	39608	94.960	BB
2	27.24	2102	5.040	BB
TOTAL				
		41710	100.000	

PEAK REJ : 0

Supplementary Fig. 82: HPLC charts of **7au**.

7av (racemate)

CH. 1 C.S 2.50 ATT 4 OFFS 0 00/00/00 00:00

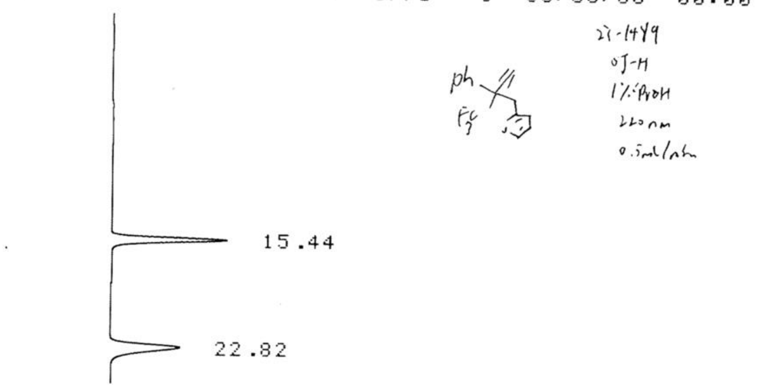

D-2500 00/00/00 00:00

METHOD: TAG: 1 CH: 1

FILE: 0 CALC-METHOD: AREA% TABLE: 0 CONC: AREA

NO.	RT	AREA	CONC	BC
1	15.44	44780	50.247	BB
2	22.82	44340	49.753	BB
TOTAL		89120	100.000	
PEAK REJ :		0		

7av (chiral)

CH. 1 C.S 2.50 ATT 3 OFFS 0 00/00/00 08:23

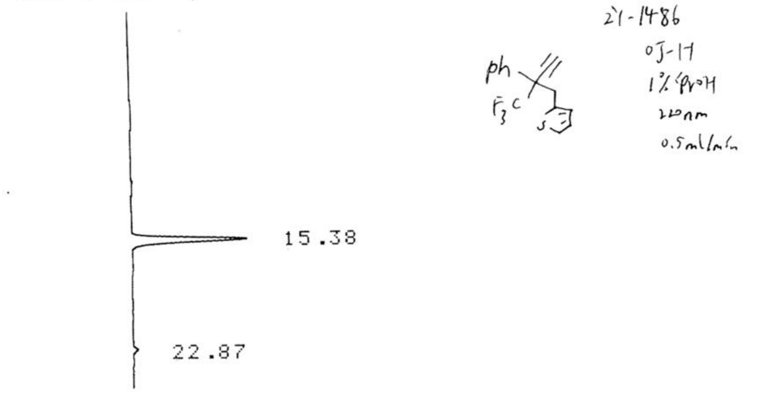

D-2500 00/00/00 08:23

METHOD: TAG: 6 CH: 1

FILE: 0 CALC-METHOD: AREA% TABLE: 0 CONC: AREA

NO.	RT	AREA	CONC	BC
1	15.38	22707	97.192	BB
2	22.87	656	2.808	BB
TOTAL		23363	100.000	
PEAK REJ :		0		

Supplementary Fig. 83: HPLC charts of **7av**.

7ba (racemate)

CH. 1 C.S 2.50 ATT 3 OFFS 0 00/00/00 00:59

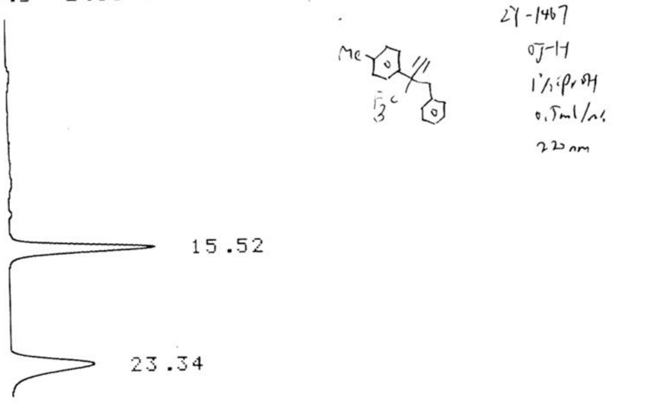

D-2500 00/00/00 00:59

METHOD: TAG: 2 CH: 1

FILE: 0 CALC-METHOD: AREA% TABLE: 0 CONC: AREA

NO.	RT	AREA	CONC	BC
1	15.52	40652	49.590	BB
2	23.34	41325	50.410	BB
TOTAL		81977	100.000	

PEAK REJ : 0

7ba (chiral)

CH. 1 C.S 2.50 ATT 3 OFFS 0 00/00/00 01:53

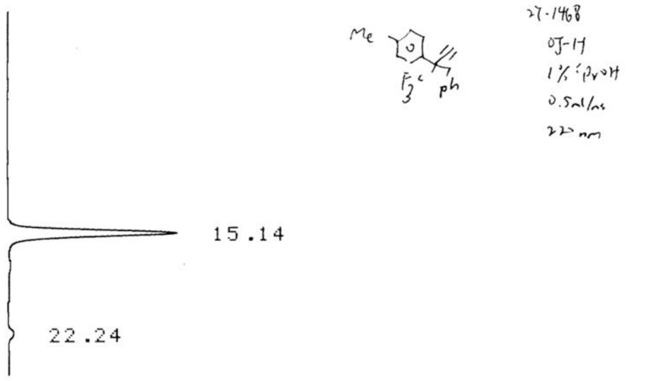

D-2500 00/00/00 01:53

METHOD: TAG: 3 CH: 1

FILE: 0 CALC-METHOD: AREA% TABLE: 0 CONC: AREA

NO.	RT	AREA	CONC	BC
1	15.14	44533	95.012	BB
2	22.24	2338	4.988	BB
TOTAL		46871	100.000	

PEAK REJ : 0

Supplementary Fig. 84: HPLC charts of **7ba**.

7ca (racemate)

CH. 1 C.S 2.50 ATT 5 OFFS 0 00/00/00 00:00

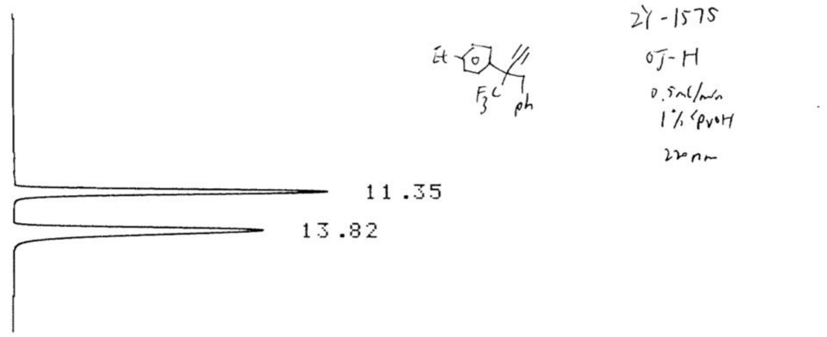

D-2500 00/00/00 00:00

METHOD: TAG: 1 CH: 1

FILE: 0 CALC-METHOD: AREA% TABLE: 0 CONC: AREA

NO.	RT	AREA	CONC	BC
1	11.35	234656	46.948	BB
2	13.82	265160	53.052	BB
TOTAL				
		499816	100.000	

PEAK REJ : 0

7ca (chiral)

CH. 1 C.S 2.50 ATT 2 OFFS 0 00/00/00 00:02

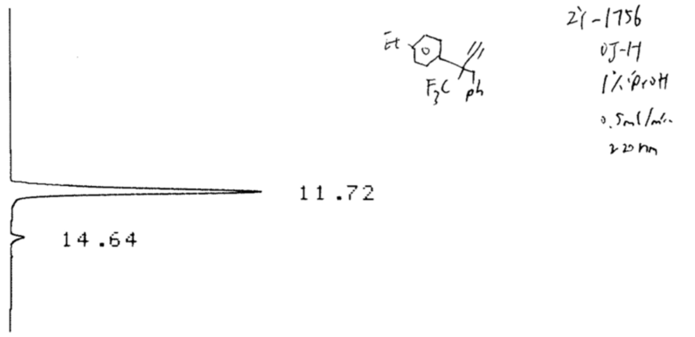

D-2500 00/00/00 00:02

METHOD: TAG: 1 CH: 1

FILE: 0 CALC-METHOD: AREA% TABLE: 0 CONC: AREA

NO.	RT	AREA	CONC	BC
1	11.72	17448	94.826	BB
2	14.64	952	5.174	BB
TOTAL				
		18400	100.000	

PEAK REJ : 0

Supplementary Fig. 85: HPLC charts of **7ca**.

7da (racemate)

CH. 1 C.S 1.25 ATT 3 OFFS 0 00/00/00 02:01

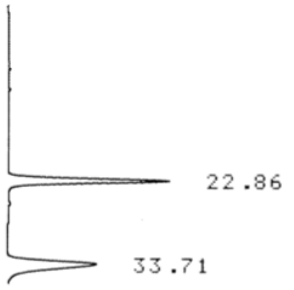

D-2500 00/00/00 02:01

METHOD: TAG: 3 CH: 1

FILE: 0 CALC-METHOD: AREA% TABLE: 0 CONC: AREA

NO.	RT	AREA	CONC	BC
1	22.86	52779	50.450	BB
2	33.71	51838	49.550	BB
TOTAL		104617	100.000	

PEAK REJ : 0

7da (chiral)

CH. 1 C.S 1.25 ATT 3 OFFS 0 00/00/00 01:25

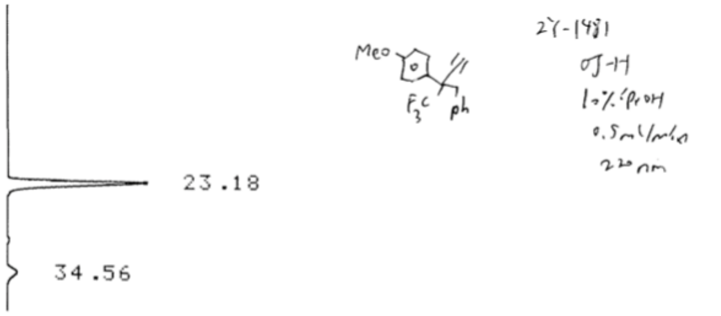

D-2500 00/00/00 01:25

METHOD: TAG: 3 CH: 1

FILE: 0 CALC-METHOD: AREA% TABLE: 0 CONC: AREA

NO.	RT	AREA	CONC	BC
1	23.18	53632	95.361	BB
2	34.56	2609	4.639	BB
TOTAL		56241	100.000	

PEAK REJ : 0

Supplementary Fig. 86: HPLC charts of **7da**.

7ea (racemate)

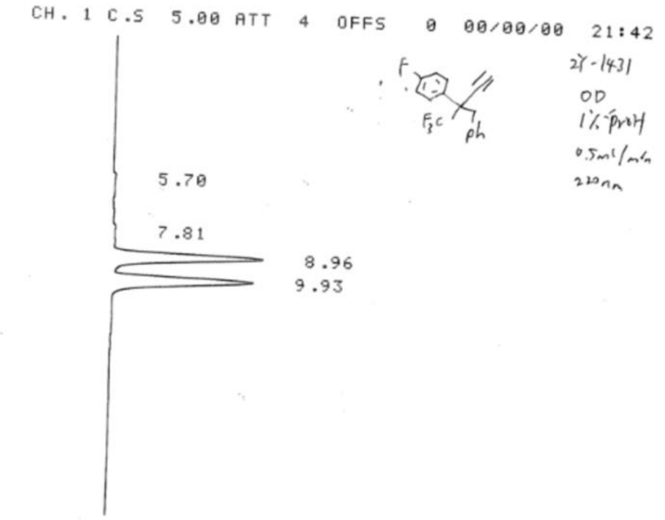

D-2500

00/00/00 21:42

METHOD: TAG: 4 CH: 1

FILE: 0 CALC-METHOD: AREA% TABLE: 0 CONC: AREA

NO.	RT	AREA	CONC	BC
3	8.96	62309	50.026	BV
4	9.93	62243	49.974	VB
TOTAL		124552	100.000	

PEAK REJ : 500

7ea (chiral)

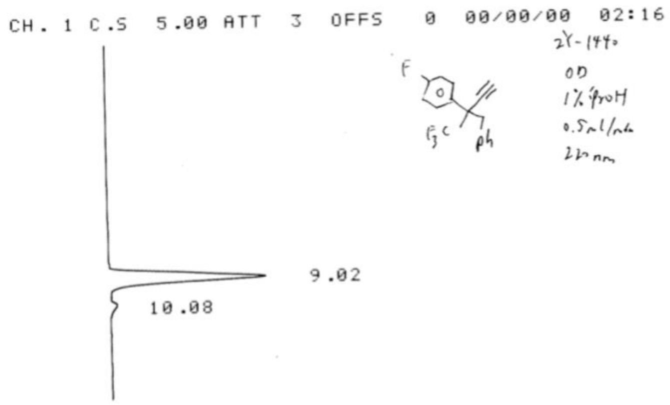

D-2500

00/00/00 02:16

METHOD: TAG: 3 CH: 1

FILE: 0 CALC-METHOD: AREA% TABLE: 0 CONC: AREA

NO.	RT	AREA	CONC	BC
1	9.02	33123	97.198	BB
2	10.08	955	2.802	TBB
TOTAL		34078	100.000	

PEAK REJ : 0

Supplementary Fig. 87: HPLC charts of **7ea**.

7fa (racemate)

CH. 1 C.S 2.50 ATT 3 OFFS 0 00/00/00 00:00

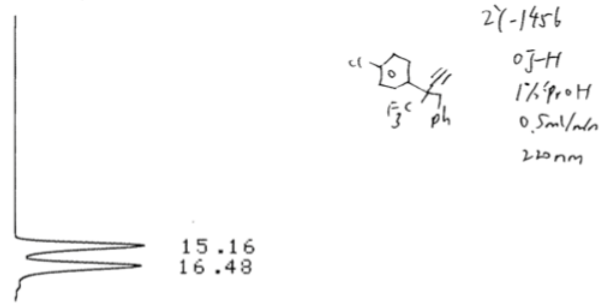

D-2500 00/00/00 00:00

METHOD: TAG: 1 CH: 1

FILE: 0 CALC-METHOD: AREA% TABLE: 0 CONC: AREA

NO.	RT	AREA	CONC	BC
1	15.16	35691	48.019	BV
2	16.48	38636	51.981	VB
TOTAL				
		74327	100.000	
PEAK REJ :		0		

7fa (chiral)

CH. 1 C.S 2.50 ATT 3 OFFS 0 00/00/00 00:25

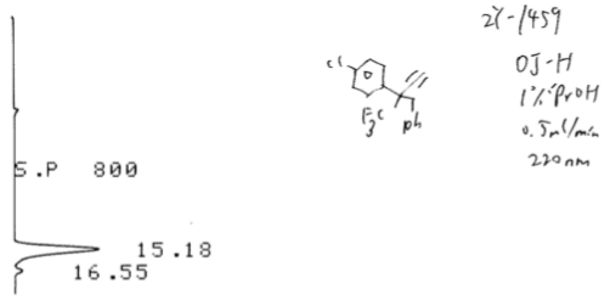

D-2500 00/00/00 00:25

METHOD: TAG: 2 CH: 1

FILE: 0 CALC-METHOD: AREA% TABLE: 0 CONC: AREA

NO.	RT	AREA	CONC	BC
1	15.18	23828	94.951	BV
2	16.55	1267	5.049	VB
TOTAL				
		25095	100.000	
PEAK REJ :		0		

Supplementary Fig. 88: HPLC charts of **7fa**.

7ga (racemate)

CH. 1 C.S 2.50 ATT 3 OFFS 0 00/00/00 01:43

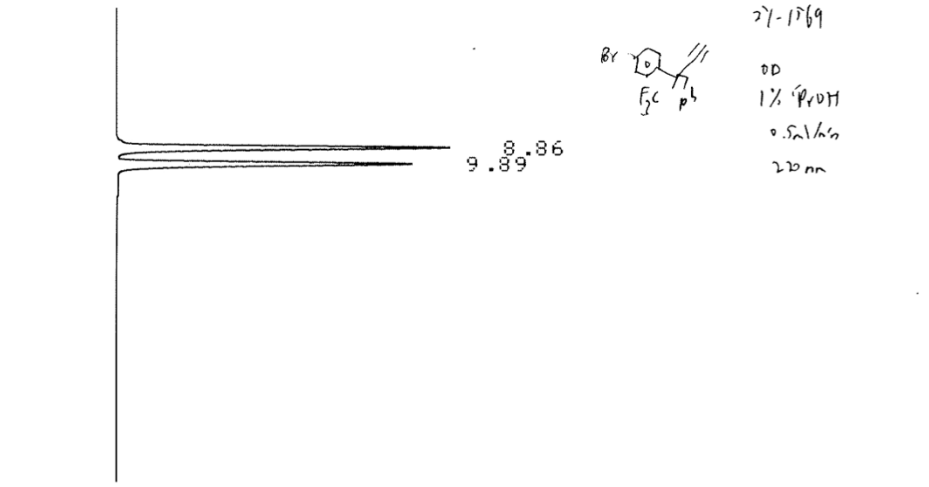

D-2500 00/00/00 01:43

METHOD: TAG: 2 CH: 1

FILE: 0 CALC-METHOD: AREA% TABLE: 0 CONC: AREA

NO.	RT	AREA	CONC	BC
1	8.86	43906	51.060	BB
2	9.89	42083	48.940	BB
TOTAL		85989	100.000	

PEAK REJ : 0

7ga (chiral)

CH. 1 C.S 2.50 ATT 3 OFFS 0 00/00/00 00:59

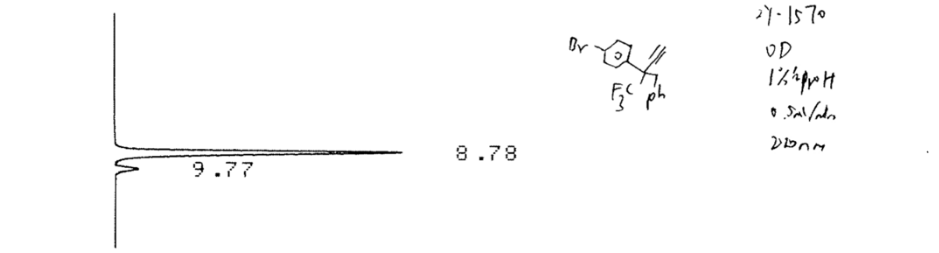

D-2500 00/00/00 00:59

METHOD: TAG: 1 CH: 1

FILE: 0 CALC-METHOD: AREA% TABLE: 0 CONC: AREA

NO.	RT	AREA	CONC	BC
1	8.78	36849	95.983	BB
2	9.77	1542	4.017	BB
TOTAL		38391	100.000	

PEAK REJ : 0

Supplementary Fig. 89: HPLC charts of **7ga**.

7ha (racemate)

CH. 1 C.S 2.50 ATT 5 OFFS 0 00/00/00 00:46

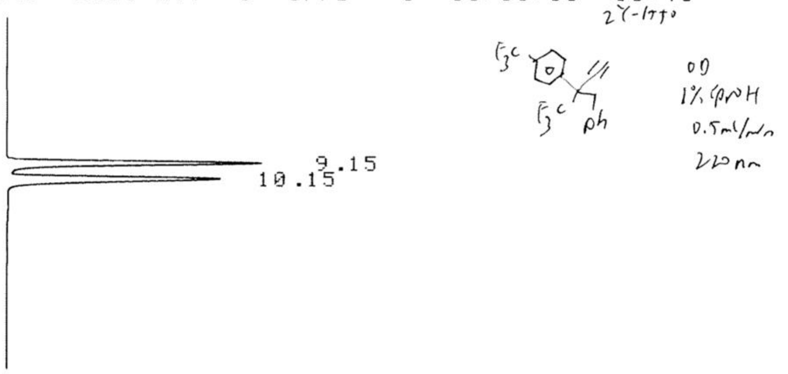

D-2500 00/00/00 00:46

METHOD: TAG: 1 CH: 1

FILE: 0 CALC-METHOD: AREA% TABLE: 0 CONC: AREA

NO.	RT	AREA	CONC	BC
1	9.15	152182	50.672	BV
2	10.15	148144	49.328	VB
TOTAL				
		300326	100.000	

PEAK REJ : 0

7ha (chiral)

CH. 1 C.S 2.50 ATT 3 OFFS 0 00/00/00 01:16

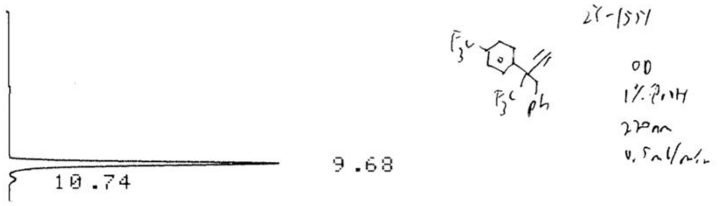

D-2500 00/00/00 01:16

METHOD: TAG: 1 CH: 1

FILE: 0 CALC-METHOD: AREA% TABLE: 0 CONC: AREA

NO.	RT	AREA	CONC	BC
1	9.68	32244	96.447	BB
2	10.74	1188	3.553	BB
TOTAL				
		33432	100.000	

PEAK REJ : 0

Supplementary Fig. 90: HPLC charts of **7ha**.

7ia (racemate)

CH. 1 C.S 2.50 ATT 3 OFFS 0 00/00/00 00:46

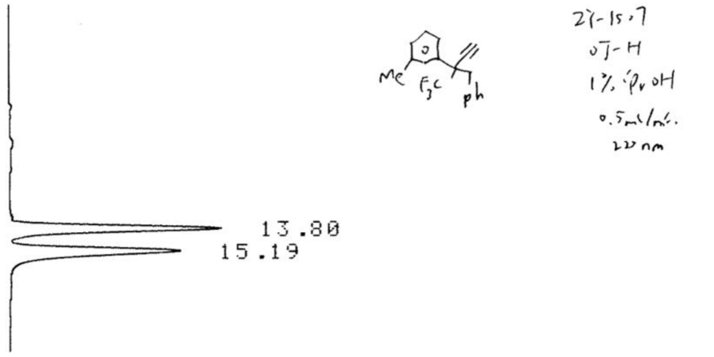

D-2500 00/00/00 00:46

METHOD: TAG: 2 CH: 1

FILE: 0 CALC-METHOD: AREA% TABLE: 0 CONC: AREA

NO.	RT	AREA	CONC	BC
1	13.80	47133	50.424	BV
2	15.19	46341	49.576	VB
TOTAL		93474	100.000	

PEAK REJ : 0

7ia (chiral)

CH. 1 C.S 2.50 ATT 3 OFFS 0 00/00/00 03:02

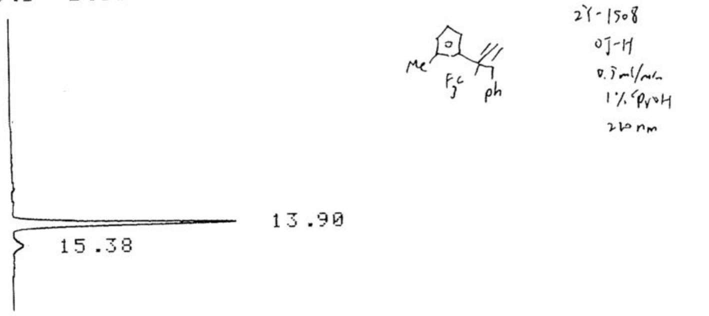

D-2500 00/00/00 03:02

METHOD: TAG: 3 CH: 1

FILE: 0 CALC-METHOD: AREA% TABLE: 0 CONC: AREA

NO.	RT	AREA	CONC	BC
1	13.90	23758	94.635	BB
2	15.38	1347	5.365	BB
TOTAL		25105	100.000	

PEAK REJ : 0

Supplementary Fig. 91: HPLC charts of **7ia**.

7ja (racemate)

CH. 1 C.S 2.50 ATT 4 OFFS 0 00/00/00 03:43

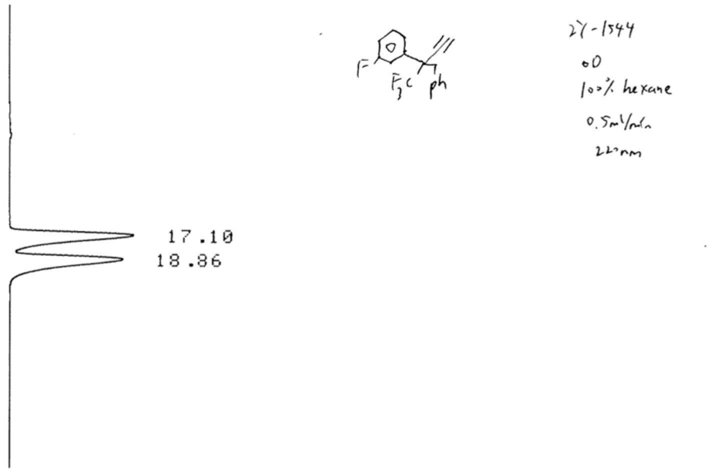

D-2500 00/00/00 03:43

METHOD: TAG: 4 CH: 1

FILE: 0 CALC-METHOD: AREA% TABLE: 0 CONC: AREA

NO.	RT	AREA	CONC	BC
1	17.10	106167	49.215	BV
2	18.86	109552	50.785	VB
TOTAL				
		215719	100.000	

PEAK REJ : 0

7ja (chiral)

CH. 1 C.S 2.50 ATT 3 OFFS 0 00/00/00 00:44

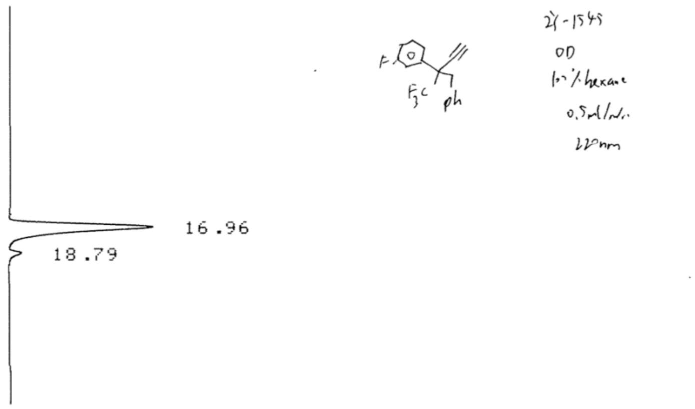

D-2500 00/00/00 00:44

METHOD: TAG: 2 CH: 1

FILE: 0 CALC-METHOD: AREA% TABLE: 0 CONC: AREA

NO.	RT	AREA	CONC	BC
1	16.96	55783	95.432	BB
2	18.79	2670	4.568	BB
TOTAL				
		58453	100.000	

PEAK REJ : 0

Supplementary Fig. 92: HPLC charts of **7ja**.

7ka (racemate)

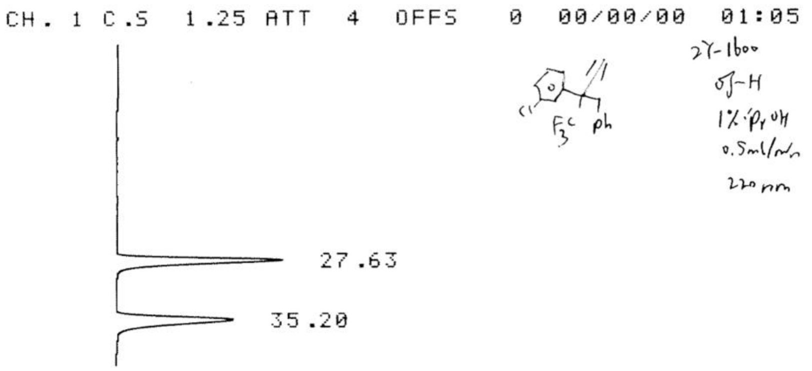

D-2500 00/00/00 01:05

METHOD: TAG: 2 CH: 1

FILE: 0 CALC-METHOD: AREA% TABLE: 0 CONC: AREA

NO.	RT	AREA	CONC	BC
1	27.63	134497	51.212	BB
2	35.20	128130	48.788	BB
TOTAL		262627	100.000	

PEAK REJ : 0

7ka (chiral)

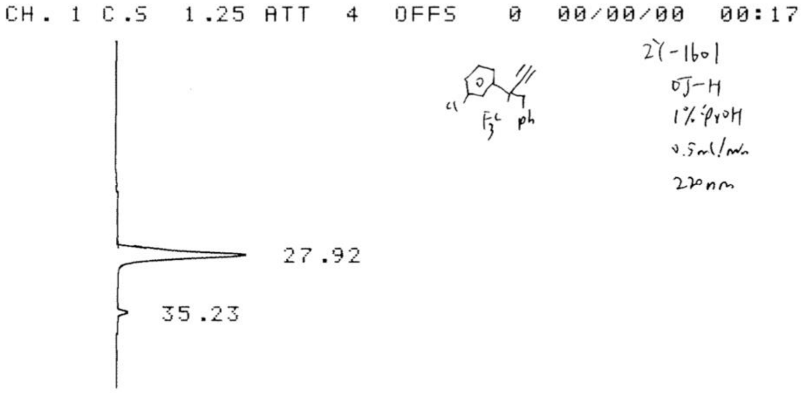

D-2500 00/00/00 00:17

METHOD: TAG: 1 CH: 1

FILE: 0 CALC-METHOD: AREA% TABLE: 0 CONC: AREA

NO.	RT	AREA	CONC	BC
1	27.92	147598	97.300	BB
2	35.23	4096	2.700	BB
TOTAL		151694	100.000	

PEAK REJ : 0

Supplementary Fig. 93: HPLC charts of **7ka**.

7la (racemate)

CH. 1 C.S 2.50 ATT 3 OFFS 0 00/00/00 01:02

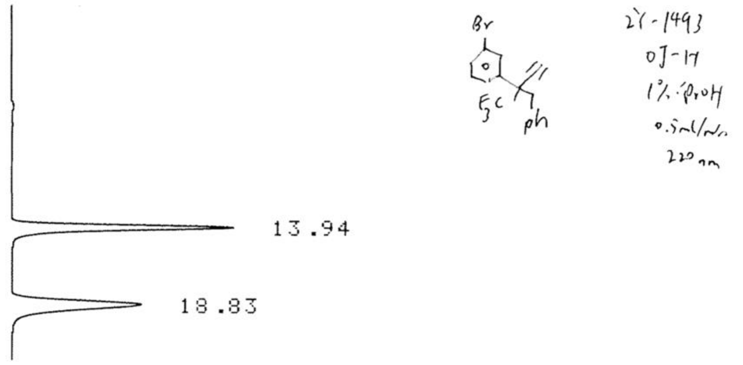

D-2500 00/00/00 01:02

METHOD: TAG: 2 CH: 1

FILE: 0 CALC-METHOD: AREA% TABLE: 0 CONC: AREA

NO.	RT	AREA	CONC	BC
1	13.94	43268	51.048	BB
2	18.83	41492	48.952	BB
TOTAL		84760	100.000	

PEAK REJ : 0

7la (chiral)

CH. 1 C.S 2.50 ATT 2 OFFS 0 00/00/00 02:28

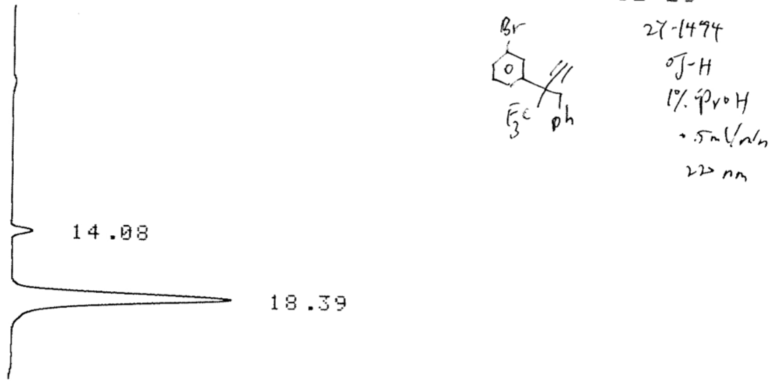

D-2500 00/00/00 02:28

METHOD: TAG: 2 CH: 1

FILE: 0 CALC-METHOD: AREA% TABLE: 0 CONC: AREA

NO.	RT	AREA	CONC	BC
1	14.08	2145	5.120	BB
2	18.39	39748	94.880	BB
TOTAL		41893	100.000	

PEAK REJ : 0

Supplementary Fig. 94: HPLC charts of **7la**.

7ma (racemate)

CH. 1 C.S 2.50 ATT 4 OFFS 0 00/00/00 01:28

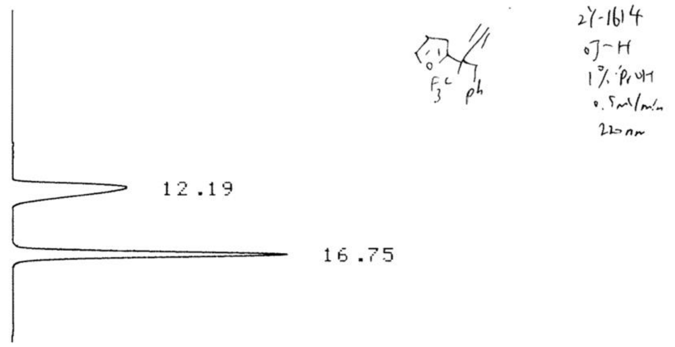

D-2500 00/00/00 01:28

METHOD: TAG: 1 CH: 1

FILE: 0 CALC-METHOD: AREA% TABLE: 0 CONC: AREA

NO.	RT	AREA	CONC	BC
1	12.19	111003	46.790	BB
2	16.75	126236	53.210	BB
TOTAL		237239	100.000	

PEAK REJ : 0

7ma (chiral)

CH. 1 C.S 2.50 ATT 2 OFFS 0 00/00/00 04:54

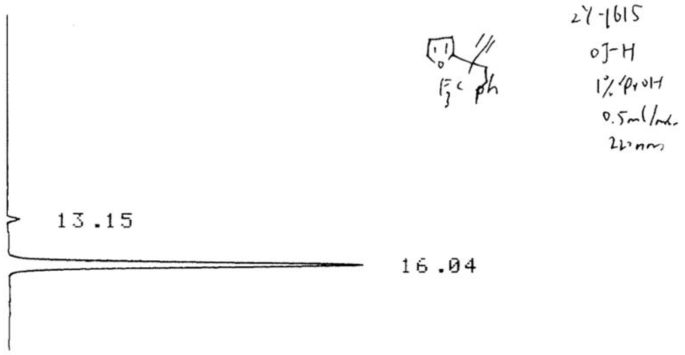

D-2500 00/00/00 04:54

METHOD: TAG: 3 CH: 1

FILE: 0 CALC-METHOD: AREA% TABLE: 0 CONC: AREA

NO.	RT	AREA	CONC	BC
1	13.15	616	1.882	BB
2	16.04	32107	98.118	BB
TOTAL		32723	100.000	

PEAK REJ : 0

Supplementary Fig. 95: HPLC charts of **7ma**.

7na (racemate)

CH. 1 C.S 1.25 ATT 4 OFFS 0 00/00/00 00:05

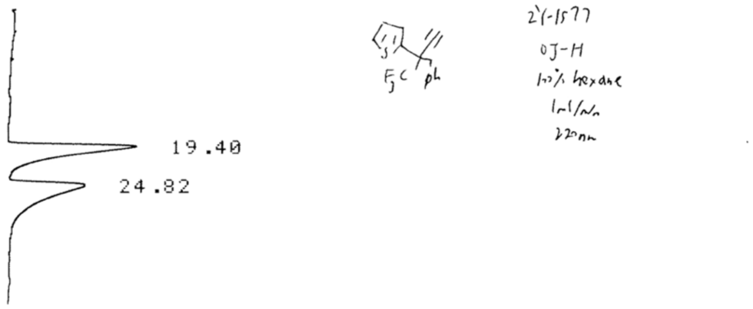

D-2500 00/00/00 00:05

METHOD: TAG: 1 CH: 1

FILE: 0 CALC-METHOD: AREA% TABLE: 0 CONC: AREA

NO.	RT	AREA	CONC	BC
1	19.40	230428	55.847	BB
2	24.82	182180	44.153	BB
TOTAL				
		412608	100.000	

PEAK REJ : 0

7na (chiral)

CH. 1 C.S 1.25 ATT 2 OFFS 0 00/00/00 02:43

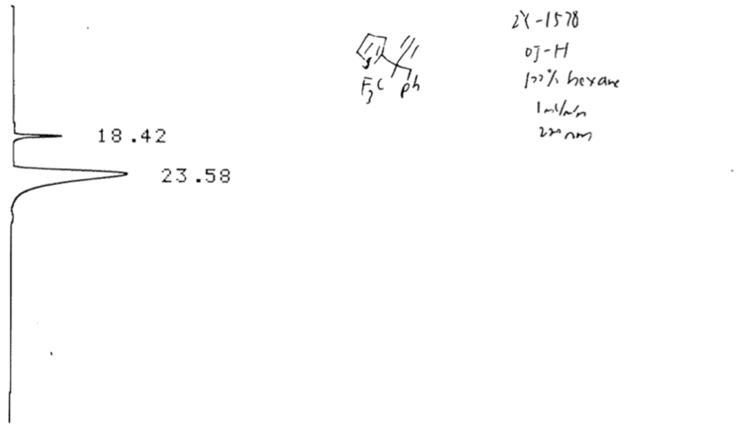

D-2500 00/00/00 02:43

METHOD: TAG: 2 CH: 1

FILE: 0 CALC-METHOD: AREA% TABLE: 0 CONC: AREA

NO.	RT	AREA	CONC	BC
1	18.42	2494	5.083	BB
2	23.58	46571	94.917	BB
TOTAL				
		49065	100.000	

PEAK REJ : 0

Supplementary Fig. 96: HPLC charts of **7na**.

7lr (racemate)

CH. 1 C.S 2.50 ATT 4 OFFS 0 00/00/00 00:43

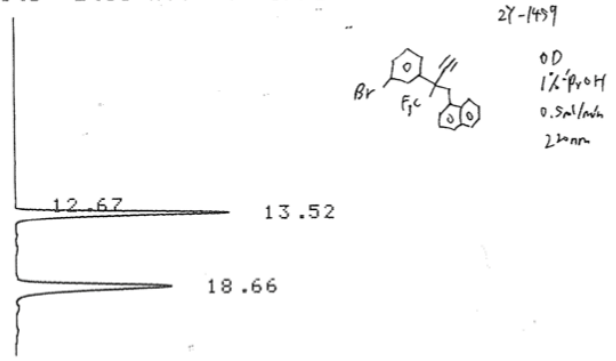

D-2500 00/00/00 00:43

METHOD: TAG: 2 CH: 1

FILE: 0 CALC-METHOD: AREA% TABLE: 0 CONC: AREA

NO.	RT	AREA	CONC	BC
2	13.52	84006	49.898	BB
3	18.66	84348	50.102	BB
TOTAL		168354	100.000	

PEAK REJ : 800

7lr (chiral)

CH. 1 C.S 2.50 ATT 4 OFFS 0 00/00/00 01:00

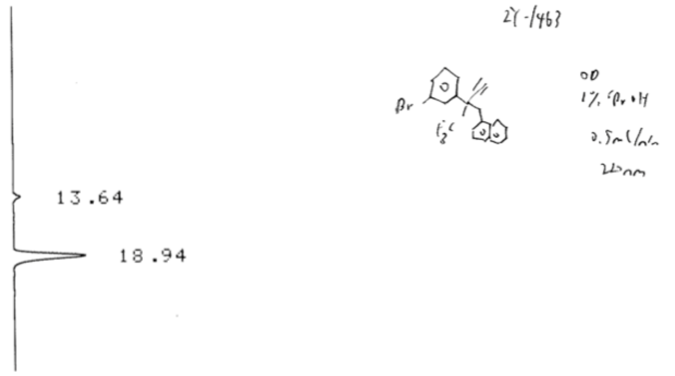

D-2500 00/00/00 01:00

METHOD: TAG: 1 CH: 1

FILE: 0 CALC-METHOD: AREA% TABLE: 0 CONC: AREA

NO.	RT	AREA	CONC	BC
1	13.64	1684	4.439	BB
2	18.94	36255	95.561	BB
TOTAL		37939	100.000	

PEAK REJ : 0

Supplementary Fig. 97: HPLC charts of **7lr**.

9 (racemate)

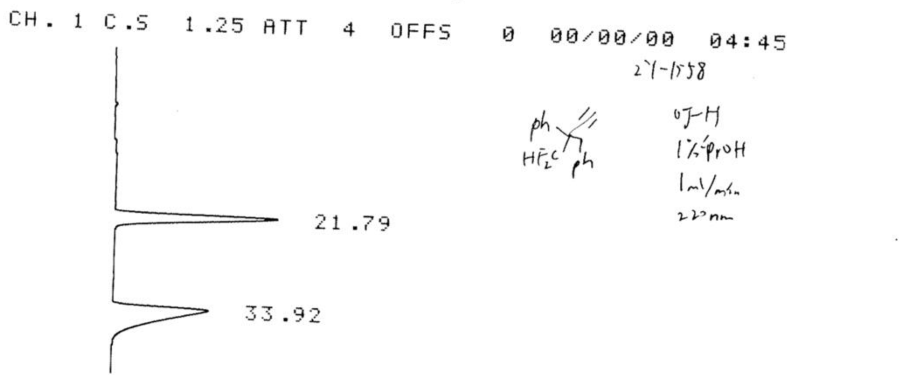

D-2500

00/00/00 04:45

METHOD: TAG: 5 CH: 1

FILE: 0 CALC-METHOD: AREA% TABLE: 0 CONC: AREA

NO.	RT	AREA	CONC	BC
1	21.79	156554	46.727	BB
2	33.92	178488	53.273	BB
TOTAL		335042	100.000	

PEAK REJ : 0

9 (chiral)

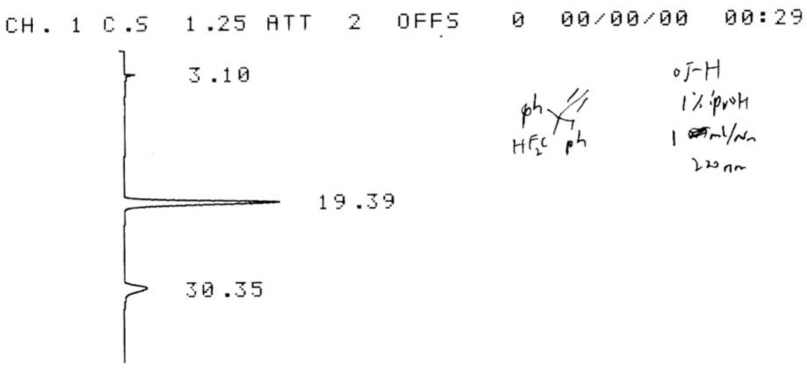

D-2500

00/00/00 00:29

METHOD: TAG: 1 CH: 1

FILE: 0 CALC-METHOD: AREA% TABLE: 0 CONC: AREA

NO.	RT	AREA	CONC	BC
2	19.39	19866	81.532	BB
3	30.35	4500	18.468	BB
TOTAL		24366	100.000	

PEAK REJ : 400

Supplementary Fig. 98: HPLC charts of **9**.

10 (racemate)

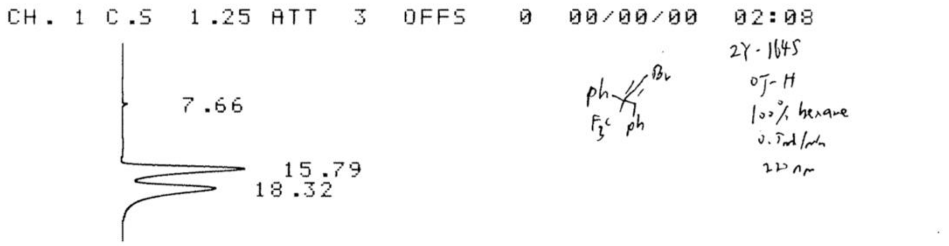

D-2500 00/00/00 02:08

METHOD: TAG: 3 CH: 1

FILE: 0 CALC-METHOD: AREA% TABLE: 0 CONC: AREA

NO.	RT	AREA	CONC	BC
2	15.79	59580	51.112	BV
3	18.32	56988	48.888	VB
TOTAL		116568	100.000	

PEAK REJ : 600

10 (chiral)

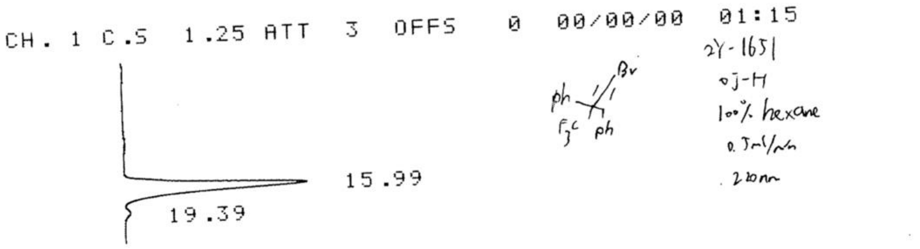

00/00/00 01:15

D-2500

METHOD: TAG: 3 CH: 1

FILE: 0 CALC-METHOD: AREA% TABLE: 0 CONC: AREA

NO.	RT	AREA	CONC	BC
1	15.99	122008	96.492	BB
2	19.39	4435	3.508	BB
TOTAL		126443	100.000	

PEAK REJ : 0

Supplementary Fig. 99: HPLC charts of **10**.

11 (racemate)

CH. 1 C.S 2.50 ATT 5 OFFS 0 00/00/00 01:56

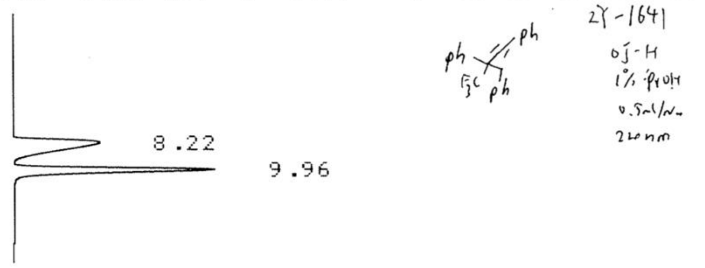

D-2500 00/00/00 01:56

METHOD: TAG: 5 CH: 1

FILE: 0 CALC-METHOD: AREA% TABLE: 0 CONC: AREA

NO.	RT	AREA	CONC	BC
1	8.22	123622	48.472	BB
2	9.96	131417	51.528	BB
TOTAL				
		255039	100.000	

PEAK REJ : 0

11 (chiral)

CH. 1 C.S 2.50 ATT 4 OFFS 0 00/00/00 07:27

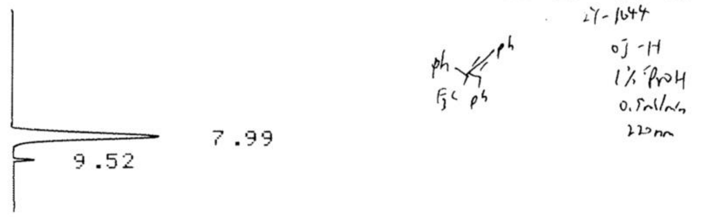

D-2500 00/00/00 07:27

METHOD: TAG: 13 CH: 1

FILE: 0 CALC-METHOD: AREA% TABLE: 0 CONC: AREA

NO.	RT	AREA	CONC	BC
1	7.99	82751	96.389	BB
2	9.52	3100	3.611	BB
TOTAL				
		85851	100.000	

PEAK REJ : 0

Supplementary Fig. 100: HPLC charts of **11**.

12 (racemate)

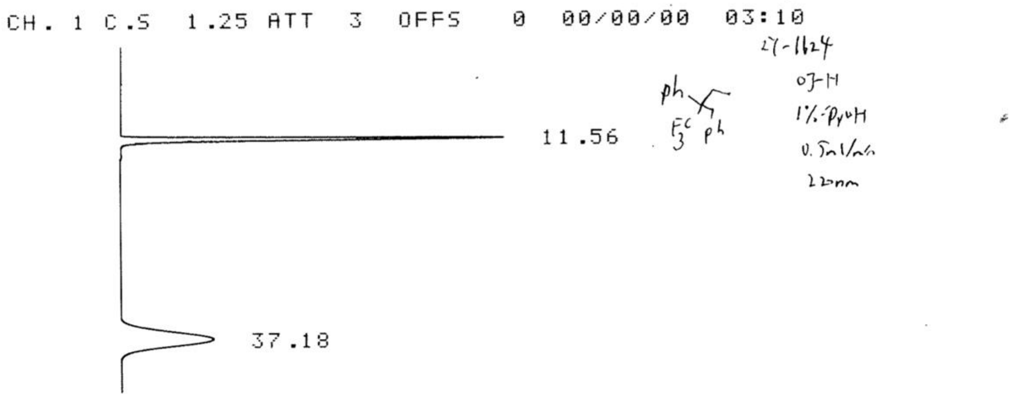

D-2500 00/00/00 03:10

METHOD: TAG: 7 CH: 1

FILE: 0 CALC-METHOD: AREA% TABLE: 0 CONC: AREA

NO.	RT	AREA	CONC	BC
1	11.56	73777	47.572	BB
2	37.18	81308	52.428	BB
TOTAL		155085	100.000	

PEAK REJ : 0

12 (chiral)

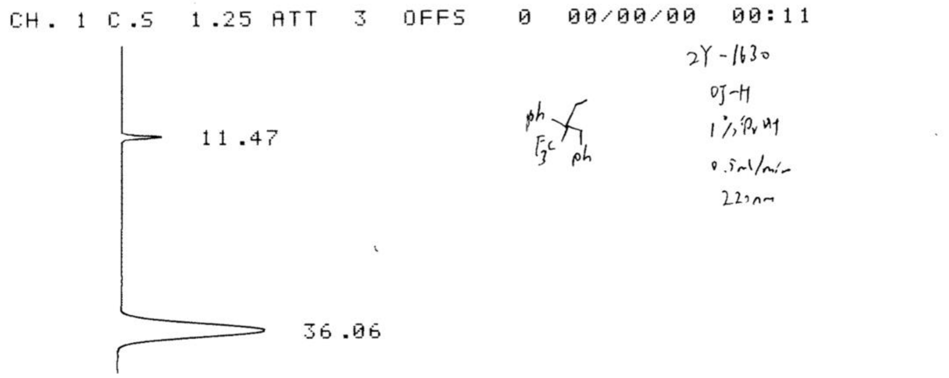

D-2500 00/00/00 00:11

METHOD: TAG: 1 CH: 1

FILE: 0 CALC-METHOD: AREA% TABLE: 0 CONC: AREA

NO.	RT	AREA	CONC	BC
1	11.47	5916	4.170	BB
2	36.06	135966	95.830	BB
TOTAL		141882	100.000	

PEAK REJ : 0

Supplementary Fig. 101: HPLC charts of **12**.

13 (racemate)

CH. 1 C.S 2.50 ATT 4 OFFS 0 00/00/00 00:11

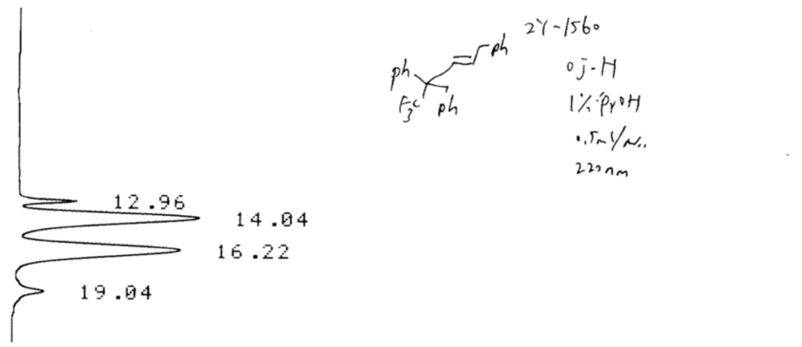

D-2500 00/00/00 00:11

METHOD: TAG: 1 CH: 1

FILE: 0 CALC-METHOD: AREA% TABLE: 0 CONC: AREA

NO.	RT	AREA	CONC	BC
1	12.96	17121	4.871	BV
2	14.04	159155	45.278	VV
3	16.22	156901	44.637	VB
4	19.04	18327	5.214	BB
TOTAL				
		351504	100.000	
PEAK REJ :		0		

13 (chiral)

CH. 1 C.S 2.50 ATT 3 OFFS 0 00/00/00 01:38

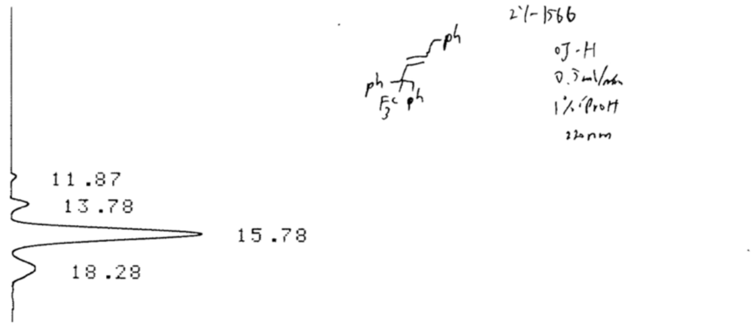

D-2500 00/00/00 01:38

METHOD: TAG: 1 CH: 1

FILE: 0 CALC-METHOD: AREA% TABLE: 0 CONC: AREA

NO.	RT	AREA	CONC	BC
2	11.87	481	0.409	BB
3	13.78	4356	3.706	BB
4	15.78	100761	85.710	BB
5	18.28	11962	10.175	BB
TOTAL				
		117560	100.000	
PEAK REJ :		0		

Supplementary Fig. 102: HPLC charts of **13**.

2. Supplementatry References

1 Sathyamoorthy, B., Axelrod, A., Farwell, V., Bennett, S. M., Calitree, B. D., Benedict, J. B., Sukumaran, D. K. & Detty, M. R. Novel 21,23-ditelluraporphyrins and the first 26,28-ditellurasapphyrin and 30,33-ditellurarubyrin. *Organometallics* **29**, 3431–3441 (2010).

2 Xu, C.-F., Xu, M., Yang, L.-Q. & Li, C.-Y. Synthesis of allenes via gold-catalyzed intermolecular reaction of propargylic alcohols and aromatic compounds. *J. Org. Chem.* **77**, 3010–3016 (2012).

3 McAdam, C. A., McLaughlin, M. G., Johnston, A. J. S., Chen, J., Walter, M. W. & Cook, M. J. Platinum catalysed hydrosilylation of propargylic alcohols. *Org. Biomol. Chem.* **11**, 4488–4502 (2013).

4 Kawai, H., Tachi, K., Tokunaga, E., Shiro, M. & Shibata, N. Cinchona alkaloid-catalyzed asymmetric trifluoromethylation of alkynyl ketones with trimethylsilyl trifluoromethane. *Org. Lett.* **12**, 5104–5107 (2010).

5 Liu, S., Tanabe, Y., Kuriyama, S., Sakata, K. & Nishibayashi, Y. Ruthenium-catalyzed enantioselective propargylic phosphinylation of propargylic alcohols with phosphine oxides. *Angew. Chem. Int. Ed.* **60**, 11231–11236 (2021).

6 Kourist, R., Bartsch, S. & Bornscheuer, U. T. Highly enantioselective synthesis of arylaliphatic tertiary alcohols using mutants of an esterase from Bacillus subtilis. *Adv. Synth. Catal.* **349**, 1393–1398 (2007).

7 Holmes, M., Nguyen, K. D., Schwartz, L. A., Luong, T. & Krische, M. J. Enantioselective formation of CF_3-bearing all-carbon quaternary stereocenters via C–H functionalization of methanol: iridium catalyzed allene hydrohydroxymethylation. *J. Am. Chem. Soc.* **139**, 8114–8117 (2017).

8 Tsuchida, K., Senda, Y., Nakajima, K. & Nishibayashi, Y. Construction of chiral tri- and tetra-arylmethanes bearing quaternary carbon centers: copper-catalyzed enantioselective propargylation of indoles with propargylic esters. *Angew. Chem. Int. Ed.* **55**, 9728–9732 (2016).

9 Li, G., Chen, R., Wu, L., Fu, Q., Zhang, X. & Tang, Z. Alkyl transfer from C–C cleavage. *Angew. Chem. Int. Ed.* **52**, 8432–8436 (2013).

10 Bai, Z., Zhang, H., Wang, H., Yu, H., Chen, G. & He, G. Enantioselective alkylamination of unactivated alkenes under copper catalysis. *J. Am. Chem. Soc.* **143**, 1195–1202 (2021).

11 Qu, Q.-Y., Min, Q.-Q., Ao, G.-Z. & Liu, F. Radical alkylation of para-quinone methides with 4-substituted Hantzsch esters/nitriles via organic photoredox catalysis. *Org. Biomol. Chem.* **16**, 6391–6394 (2018).

12 Nakajima, K., Nojima, S., Sakata, K. & Nishibayashi, Y. Visible-light-mediated aromatic substitution reactions of cyanoarenes with 4-alkyl-1,4-dihydropyridines through double carbon–carbon bond cleavage. *ChemCatChem* **8**, 1028–1032 (2016).

13 Nakajima, K., Nojima, S. & Nishibayashi, Y. Nickel- and photoredox-catalyzed cross-coupling reactions of aryl halides with 4-alkyl-1,4-dihydropyridines as formal nucleophilic alkylation reagents. *Angew. Chem. Int. Ed.* **55**, 14106–14110 (2016).

14 Zhang, H.-H., Zhao, J. -J. & Yu, S. Enantioselective allylic alkylation with 4-alkyl-1,4-dihydro-pyridines enabled by photoredox/palladium cocatalysis. *J. Am. Chem. Soc.* **140**, 16914–16919 (2018).

15 Nakajima, K., Guo, X. & Nishibayashi, Y. Cross-coupling reactions of alkenyl halides with 4-benzyl-1,4-dihydropyridines associated with E to Z isomerization under nickel and photoredox catalysis. *Chem. Asian J.* **13**, 3653–3657 (2018).

16 Kidwai, M., Saxena, S., Mohan, R. & Venkataramanan, R. A novel one pot synthesis of nitrogen containing heterocycles: an alternate methodology to the Biginelli and Hantzsch reactions. *J.*

Chem. Soc., Perkin Trans. 1 1845-1846 (2022).
17 Zhang, Y., Tanabe, Y., Kuriyama, S. & Nishibayashi, Y. Cooperative photoredox- and nickel-catalyzed alkylative cyclization reactions of alkynes with 4-alkyl-1,4-dihydropyridines. *J. Org. Chem.* **86**, 12577–12590 (2021).
18 Nishibayashi, Y., Wakiji, I. & Hidai, M. Novel propargylic substitution reactions catalyzed by thiolate-bridged diruthenium complexes via allenylidene intermediates. *J. Am. Chem. Soc.* **122**, 11019−11020 (2000).
19 Inada, Y., Nishibayashi, Y. & Uemura, S. Ruthenium-catalyzed asymmetric propargylic substitution reactions of propargylic alcohols with acetone. *Angew. Chem., Int. Ed.* **44**, 7715–7717 (2005).
20 Yasu, Y., Koike, T. & Akita, M. Visible light-induced selective generation of radicals from organoborates by photoredox catalysis. *Adv. Synth. Catal.* **354**, 3414–3420 (2012).
21 Dixon, I. M., Collin, J., Sauvage, J., Flamigni, L., Encinas, S. & Barigelletti, F. A family of luminescent coordination compounds: iridium(iii) polyimine complexes. *Chem. Soc. Rev.* **29**, 385–391 (2000).
22 Pitre, S. P., McTiernan, C. D. & Scaiano, J. C. Understanding the kinetics and spectroscopy of photoredox catalysis and transition-metal-free alternatives. *Acc. Chem. Res.* **49**, 1320–1330 (2016).
23 Flamigni, L., Barbieri, A., Sabatini, C., Ventura, B. & Barigelletti, F. Photochemistry and photophysics of coordination compounds: iridium. *Top. Curr. Chem.* **281**, 143–203 (2007).
24 Hatchard, C. G. & Parker, C. A. A new sensitive chemical actinometer - II. Potassium ferrioxalate as a standard chemical actinometer. *Proc. R. Soc. London Ser. A* **235**, 518– 526 (1956).
25 CrysAlisPro, version 1.171.41.99a, Data Collection and Processing Software, Rigaku Corporation, Tokyo, Japan (2021).
26 CrystalStructure, version 4.3, Crystal Structure Analysis Package, Rigaku Corporation, Tokyo, Japan, (2021).
27 Sheldrick, G. M. SHELXT: integrating space group determination and structure solution. *Acta Crystallogr.* **A70**, C1437 (2014).
28 Sheldrick, G. M. Crystal structure refinement wish SHELXL. *Acta Crystallogr.* **C71**, 3–8 (2015).
29 Ibers, J. A. & Hamilton, W. C. Dispersion corrections and crystal structure refinements. *Acta Crystallogr.* **17**, 781–782 (1964).
30 Creagh, D. C. & Hubbell, J. H. Mass attenuation coefficients (cm^2 g^{-1}). in *International Tables for Crystallography* (ed: Wilson, A. J. C.) Vol. C, Table 4.2.3.3., pp. 200–206 ((Kluwer Academic Publishers, 1992).
31 Creagh, D. C. & McAuley, W. J. Dispersion corrections for forward scattering. in *International Tables for Crystallography* (ed: Wilson, A. J. C.) Vol. C, Table 4.2.6.8., pp. 219–222 (Kluwer Academic Publishers, 1992).
32 Maslen, E. N., Fox, A. G. & O'Keefe, M. A. Coefficients for analytical approximation to the scattering factors of Tables 6.1.1.1 and 6.1.1.3. in *International Tables for Crystallography* (ed: Wilson, A. J. C.) Vol. C, Table 6.1.1.4., pp. 500–503 (Kluwer Academic Publishers, 1992).
33 Frisch, M. J. et al. Gaussian 16, Revision A.03. Gaussian, Inc., Wallingford CT (2016).
34 Chai, J.-D. & Head-Gordon, M., Long-range corrected hybrid density functionals with damped atom–atom dispersion correction. *Phys. Chem. Chem. Phys.* **10**, 6615−6620 (2008).
35 Kohn, W. & Sham, L. J. Self-consistent equations including exchange and correlation effects. *Phys. Rev.* **140**, A1133−A1138 (1965).
36 Hehre, W. J., Radom, L., Schleyer, P. v. R. & Pople, J. A. *Ab Initio Molecular Orbital Theory* (Wiley, 1986).

37 Dolg, M., Wedig, U. & Preuss, H. Energy-adjusted ab initio pseudopotentials for the first row transition elements. *J. Chem. Phys.* **86**, 866−872 (1987).
38 Miertuš, S., Scrocco, E. & Tomasi, J. Electrostatic interaction of a solute with a continuum. A direct utilization of ab initio molecular potentials for the prevision of solvent effects. *Chem. Phys.* **55**, 117−129 (1981).
39 Scalmani, G. & Frisch, M. Continuous surface charge polarizable continuum models of solvation. I. General formalism. *J. Chem. Phys.* **132**, 114110 (2010).
