# Supplementary material for: Interplay of diruthenium catalyst in controlling enantioselective propargylic substitution reactions with visible light-generated alkyl radicals
Source: Nat Commun. 2023 Feb 23;14:859. doi: 10.1038/s41467-023-36453-9 (PMC9950057; doi:10.1038/s41467-023-36453-9)
Supplement: Supplementary file 3 — Description of Additional Supplementary Files [file 41467_2023_36453_MOESM3_ESM.docx]

**Description of Additional Supplementary Files**

**File Name: Supplementary Data 1**

**Description:** Information of Cartesian Coordinates.
